# A Simple and Rapid Method for Quantitative HPLC MS/MS Determination of Selected Perfluorocarboxylic Acids and Perfluorosulfonates in Human Serum

**DOI:** 10.1155/2020/8878618

**Published:** 2020-10-16

**Authors:** Valentina Marra, Annalisa Abballe, Elena Dellatte, Nicola Iacovella, Anna Maria Ingelido, Elena De Felip

**Affiliations:** Istituto Superiore di Sanità, Dipartimento Ambiente e Salute, Viale Regina Elena 299, Roma 00161, Italy

## Abstract

Per- and polyfluorinated alkyl substances (PFASs) are ubiquitous environmental contaminants, present in the environment and in the human body. They have raised global concern because of their diffusion in the environment, particularly in water, causing cases of human overexposure due to consumption of contaminated drinking water. Human biomonitoring is the most effective way to characterize human exposure to PFASs, and it is important that as many labs as possible could easily perform this kind of analysis. Analytical methods for quantitation of PFAS mixtures in human serum have been developed, but most of them required materials that are not always easily available in all the laboratories. This paper describes a very simple and accessible HPLC MS/MS method of analysis and quantification of 13 perfluorocarboxylic acids and perfluorosulfonate compounds (belonging to the class of per- and polyfluorinated alkyl substances (PFASs)) in human serum. Method development data provide detailed descriptions of the optimization process in terms of sample preparation, laboratory analysis of human serum samples, determination of analytes by HPLC MS/MS, and describing the pump gradient time, working conditions, and acquisition.

## 1. Introduction

Perfluoroalkyl and polyfluoroalkyl substances (PFASs) are a class of synthetic organic chemicals containing fully (per) or partly (poly) fluorinated carbon chains with different functional groups used since 1950s in various industrial and commercial products (fire foams, clothes, protective coatings for carpets, pots, paints, and insecticides) [1]. For many years, they have spread in the environment (water, soil, and biota) and have also entered the food chain [2–6]. Their chemical-physical properties are thermal and chemical resistance, impermeability to oil and water, and surfactant properties. These chemical-physical properties combined with high industrial production and frequent and widespread use have made PFAS persistent contaminants capable of spreading easily in the environment without degrading but bioaccumulating and bioconcentrating in the food chain [7, 8]. Human exposure to these substances can pose a risk to public health [9–13]; for this reason, the scientific community in the last few years is implementing a series of monitoring measures for these substances in the environment and in humans. In particular, human biomonitoring is the most effective way to characterize human exposure to PFAS. We therefore developed a very simple method for the analysis of PFAS in human serum by liquid chromatography tandem mass spectrometry. The sample preparation is based on the denaturation of proteins with acetonitrile, volume reduction, and injection: no extraction or purification in the solid phase is necessary.

## 2. Materials and Methods

The Laboratory of the Italian National Institute of Health (ISS), Human Exposure to Environmental Contaminants Unit, has developed the method described in this paper.

We ordinarily use the method hereafter described for the analysis of serum samples within the framework of various national and international projects and in interlaboratory comparison exercises [10, 14–18], Ring Exercise Test for Persistent Organic Pollutants in Human Serum https://www.inspq.qc.ca/en/ctq/eqas/amap/description [19]. Results obtained by our laboratory in the interlaboratory comparison exercises are reported in detail in supplementary data 2. Reports of the interlaboratory comparison exercises are in supplementary data 3.

### 2.1. Sample Preparation

Sampling is usually performed by Italian local sanitary units in charge for blood withdrawal, which we provide with specific instructions to avoid PFAS contamination, particularly contaminations deriving from the presence of teflon-coated materials or similar (these are among the major causes of analytical interferences found in PFAS analysis). We recommend using polycarbonate or high-density polyethylene materials to collect or store the samples. Blood withdrawal (about 5 mL) must be performed with a serum tube containing no anticoagulant (may contain coagulation activator and separator gel) that must be placed in a suitable rack in a vertical position (avoiding as far as possible any solicitation of the contents) and left at room temperature until the clot is completely formed (about 30 min) and then centrifuged (centrifuge within 2 hours after collection) at about 3500 g for 15 minutes at 20°C.

**Figure 1:**
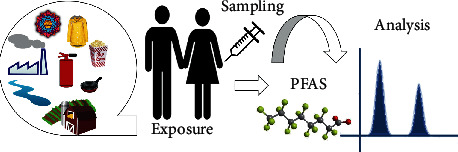
Assessment of human exposure to PFAS through biomonitoring.

After centrifugation, the tube must be removed from the centrifuge and placed in a rack, and the serum was removed from the tube with a pipette, transferred into a 15 mL polypropylene tube with a properly labelled Falcon-type screw cap, and immediately frozen keeping the tube in a vertical position inside a suitable test tube rack. 15 mL polypropylene test tubes used for storing and shipping the serum samples must be identified by indicating the identification code of the sample on the label. The tubes containing the serum samples should be stored at −20°C in a rack until shipment. Before analysis, the serum samples are allowed to warm up at room temperature and thoroughly mixed, and 250 *μ*L must be aspirated with a pipette, transferred to a 15 mL polypropylene centrifuge tube, weighed and spiked with 50 *μ*L of internal standard solution in acetonitrile (concentration: 210.40 ng/mL each PFAS), and allowed to rest overnight at 4°C. The following steps are performed after samples have been allowed to equilibrate to room temperature for one hour. The spiked samples are mixed with acetonitrile and centrifuged (Megafuge 1.0 R Heraeus Sepatech) at 3500 rpm for 15 min to precipitate proteins. After centrifugation and separation of the two phases, the volume of the supernatant is reduced in a multiple sample evaporator system (Zymark TurboVap LV concentration evaporator) to about 200 *μ*L, transferred to an autosampler vial, evaporated to dryness, and added with 300 *μ*L of the injection standard solution (13C4 PFHpA 50 ng/mL in acetonitrile) to undergo instrumental analysis. Extracts are stored at 4°C and analyzed within 1 week after conditioning at room temperature for one hour.  Figure 1 shows in graphic form how human exposure to PFAS usually occurs (indicating the major sources of exposure) and how these compounds are analyzed in human body through blood sampling and analysis.

### 2.2. Chemicals

High-purity chemical ^13^C-labelled internal standards of perfluorobutanoic acid (PFBA), perfluorohexanoic acid (PFHxA), perfluorooctanoic acid (PFOA), perfluorohexane sulfonate (PFHxS), perfluorooctane sulfonate (PFOS), perfluorononanoic acid (PFNA), perfluorodecanoic acid (PFDA), perfluoroundecanoic acid (PFUdA), and perfluorododecanoic acid (PFDOA) are purchased from Wellington Laboratories (Wellington Laboratories Inc., Ontario, Canada, N1G 3M5). The ^13^C-labelled internal standard solution is prepared by withdrawing, with a 500 *μ*L syringe, 2.5 mL of a commercial mixture of PFAS 13°C or 18O-labelled identified as “MPFAC-MXA,” at a concentration of 2000 ng/mL diluted with acetonitrile up to the volume of 25 mL in a class A glass flask and stored in the refrigerator at 4 ± 3°C until use. The serum samples are analyzed for nine perfluorocarboxylic acids: PFBA, PFPeA, PFHxA, PFHpA, PFOA, PFNA, PFDA, PFUdA, and PFDoA and four perfluorosulfonates: PFBS, PFHxS, PFHpS, and PFOS; unlabelled standards of these PFASs are purchased from Wellington Laboratories (Wellington Laboratories Inc., Ontario, Canada, N1G 3M5). The unlabelled PFAS standard solution identified as “PFAS,” at a concentration of 200 ng/mL, is prepared by withdrawing 1 mL of a commercial mixture of natural PFAS identified as “PFAC-MXB,” at a concentration of 2000 ng/mL diluted with acetonitrile up to the volume of 10 mL in a glass class A flask. This solution is stored at 4 ± 3°C until use. The injection standard solution (^13^C_4_ PFHpA 50 ng/mL in acetonitrile) identified as “13C4PFHpA” is prepared by withdrawing 1 mL of a commercial mixture of ^13^C_4_ PFHpA 50 *μ*g/mL and diluted with acetonitrile at a volume of 1000 mL in a glass class A flask. It is stored at 4 ± 3°C until use. HPLC-grade acetonitrile (≥99.9% purity) is purchased from VWR Chemicals (Radnor, Pennsylvania, United States), and HPLC-grade water is purchased from J.T. Becker (Thermo Fisher Scientific, Hampton, New Hampshire, United States).

### 2.3. Instrumental Analysis

Instrumental analysis is carried out by HPLC (Waters Alliance 2695, Waters Corporation, Milford, MA, USA). Chromatographic separation is achieved using a Kinetex C18 Column (5 µm, 100 mm × 2.1 mm ID, 100 Å) supplied by Phenomenex (Torrance, CA, USA) operated at a temperature of 45°C. A cartridge column (YMC ODS-A 5 *μ*m, 20 × 4.0 mm ID, 120 Å) supplied by YMC Europe (Dinslaken, Germany) is attached in line before the column to trap residues from the mobile phase or the system. An injection volume of 10 *μ*L is determined to be optimal considering the required sensitivity of the method and the chromatographic performance. Larger injection volumes have been shown to cause matrix effect. The mobile phases are an acetic acid/ammonium acetate solution in water (A) and acetonitrile (B). Solution A is prepared by adding 19 mg ammonium acetate and 4.5 mL acetic acid to 250 mL of water. We have investigated the effect of the pH of solution A on the chromatographic behavior of the analytes and found that an improvement of short-chained PFAS peak shapes is achieved at lower pH values; pH 2.24 resulted to be good enough to analyze short-chained PFASs and acceptable to limit system deterioration. The flow during the injection is 0.35 mL/min, and the HPLC pump gradient timetable is shown in Table 1. The HPLC is interfaced with a triple quadrupole mass spectrometer (Micromass Quattro micro TM API, Waters Corporation, Milford, MA, USA).

Analytes are detected by electrospray negative ionization in ion multiple reaction monitoring (MRM) mode, and argon is used as collision gas. A minimum of ten scans across the chromatographic peak is required to ensure adequate peak shape. Working conditions and acquisition parameters are reported in Tables 2 and 3.

## 3. Results

**Figure 2:**
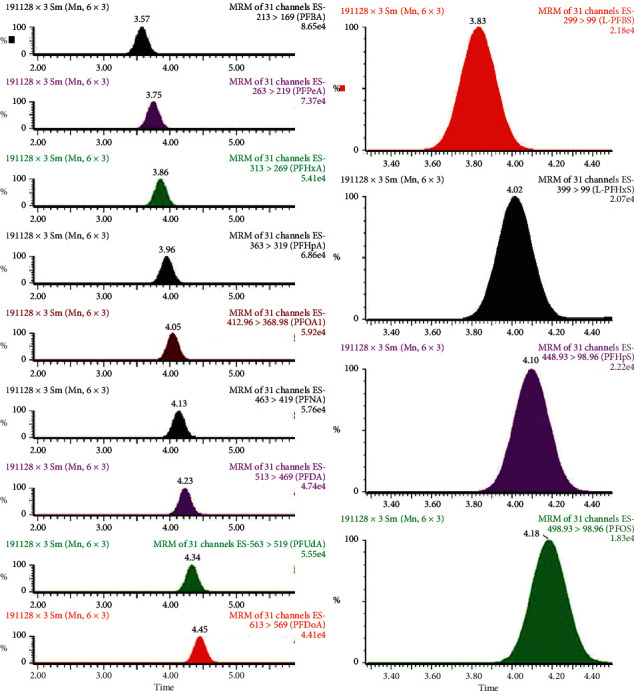
Chromatogram (MRM) of PFAS standard solution (perfluorocarboxylic acids (left) and perfluorosulfonate compounds (right)).

**Figure 3:**
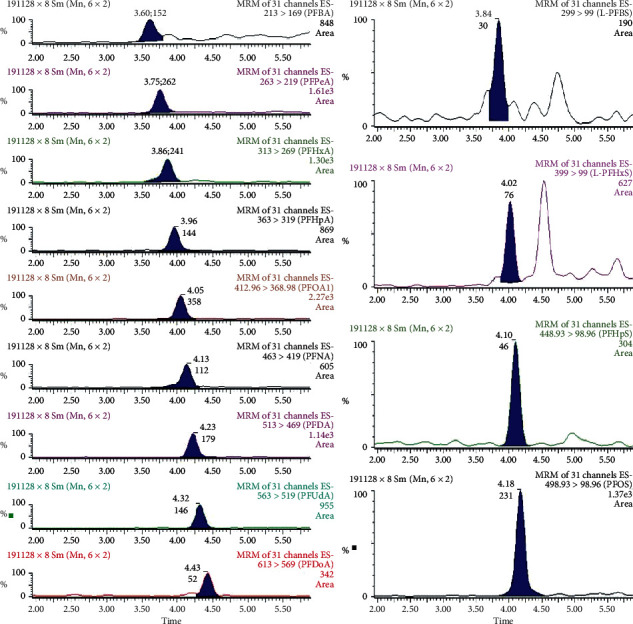
Chromatogram (MRM) of a real serum sample (perfluorocarboxylic acids (left) and perfluorosulfonate compounds (right) are present in the sample at very low levels).

In human serum, perfluorosulfonates (particularly, PFHxS and PFOS) consist of significant quantities of branched isomers, whereas perfluorocarboxylic acids are predominantly linear. In our method, we quantify perfluorosulfonates as the sum of linear and branched isomers. For PFOS quantification, we selected the transition 499 > 80 as it is more sensitive than the m/*z* 499 > 99 transition and generally preferred when quantifying both linear and branched isomers. Data acquisition and processing are performed using MassLynx, ver. 4.1 and TargetLynx Application Manager. Analytes are quantified against a reference standard by applying the isotope dilution technique. The reference standard for PFAS quantification is prepared with each batch of test samples. The limits of quantification range are 0.01–0.5 ng/mL. Figures 2 and 3 show some examples of chromatograms, which illustrate the separation of perfluorocarboxylic acids and perfluorosulfonate compounds typically found in human serum.

**Figure 4:**
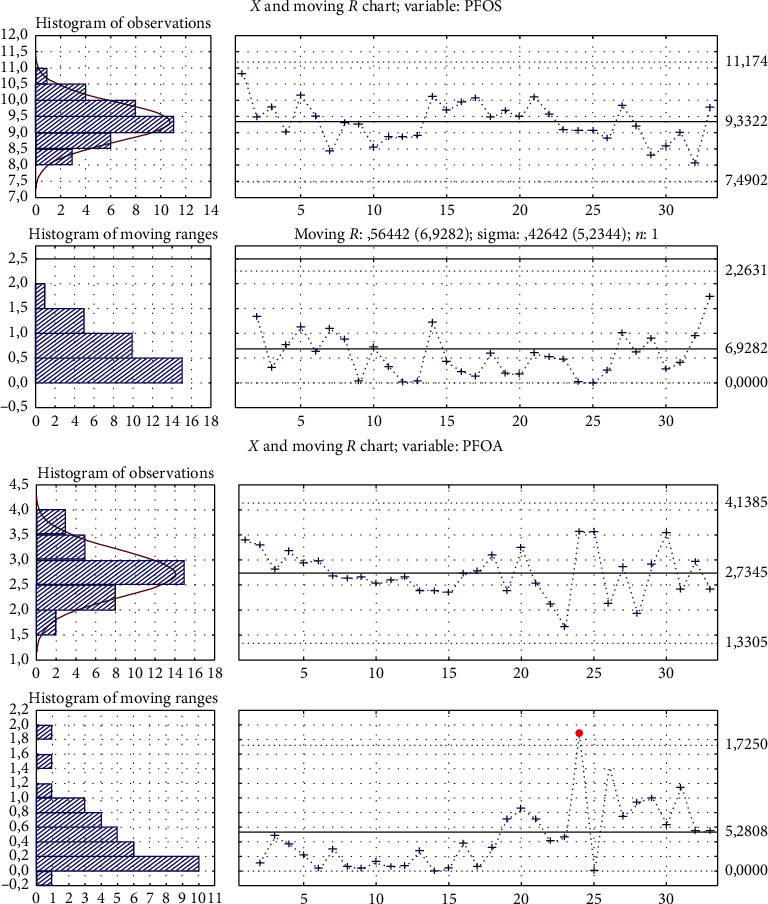
Quality control charts of PFOA and PFOS.

As required by the internal control quality, each laboratory bottle and vial is tested before first use: washing acetonitrile is added, concentrated, and analyzed. If it is PFAS-free, then the glassware can be used. According to the quality system, for each batch of test samples (20 test samples in one batch), at least one procedural blank and one quality control sample (QC) are analyzed. The blank and the control sample are spiked, extracted, and treated like the test samples. The concentration of the quality control sample is plotted on a control chart to verify that the procedure is under control. If the analytical batch results to be out of control, results are rejected and causes of the out of control are analyzed and corrected until the system quality control procedures give positive results. We have been using for years the described method for the analysis of PFAS in serum samples within the framework of various national and international projects and in interlaboratory comparison exercises and consider it suitable for routine analysis.  Figure 4 shows an example of the quality control charts we use for each batch of test samples (20 test samples in one batch). We have selected control charts of the most known and most determinable compounds in human serum, PFOS, and PFOA; control charts of all the determined analytes are reported in supplementary data 1.

## 4. Conclusion

In the present study, a simple and fast analytical method was developed to simultaneously quantify 13 perfluoroalkyl compounds. This method has been developed for the quantification of 13 perfluorocarboxylic acids and perfluorosulfonate compounds (belonging to the class of alkyl per- and polyfluorinated substances (PFAS)) in human serum.

## Figures and Tables

**Table 1:** Water alliance 2695 HPLC pump gradient timetable.

Time	A%	B%
0.00	90	10
0.10	0	100
4.00	0	100
7.00	90	10

**Table 2:** MS tune conditions.

*Voltages*	
Capillary (KV)	1

*Temperatures*	
Source temp (°C)	120
Desolvation temp (°C)	450

*Gas flow*	
Desolvation (L/hr)	500
Cone (L/hr)	25

**Table 3:** Acquisition parameters.

Analyte	Trace	RT (min)	Dwells (s)	Cone (V)	Collision (V)
PFBA	213 > 169	3.60	0.02	12	9
PFPeA	263 > 219	3.76	0.02	15	9
PFHxA	313 > 269	3.88	0.02	15	10
PFHpA	363 > 319	3.97	0.02	15	9
PFOA	413 > 369	4.05	0.02	15	11
PFNA	463 > 419	4.13	0.02	15	11
PFDA	513 > 469	4.23	0.02	15	13
PFUdA	563 > 519	4.31	0.02	15	11
PFDoA	613 > 569	4.42	0.02	20	12
PFBS	299 > 99	3.84	0.02	40	25
PFHxS	399 > 99	4.02	0.02	50	30
PFHpS	449 > 99	4.08	0.02	55	35
PFOS	499 > 80	4.16	0.02	55	45

PFBA^13^C_4_	217 > 172	3.60	0.02	12	9
PFHxA^13^C_2_	315 > 270	3.88	0.02	15	10
PFHpA^13^C_4_	367 > 322	3.97	0.02	15	9
PFOA^13^C_4_	417 > 372	4.05	0.02	15	11
PFNA^13^C_5_	468 > 423	4.13	0.02	15	11
PFDA^13^C_2_	515 > 470	4.23	0.02	15	13
PFUdA^13^C_2_	565 > 520	4.31	0.02	15	11
PFDoA^13^C_2_	615 > 570	4.42	0.02	20	12
PFHxS^18^O_2_	403 > 103	4.02	0.02	50	30
PFOS^13^C_4_	503 > 80	4.16	0.02	55	45

## Data Availability

The reports of the AMAP and HBM4EU (with a good z-score) intercalibration exercises (https://www.hbm4eu.eu; AMAP Ring Exercise Test for Persistent Organic Pollutants in Human Serum https://www.inspq.qc.ca/en/ctq/eqas/amap/description [10, 15–18]) support the findings of this study.

## Supplementary Materials

Supplementary Materials Supplementary data 1: quality control sample charts—quality control charts of PFASs concentrations in the quality control sample were analyzed and collected in our laboratory in the last 5 years. Supplementary data 2: control charts' *z*-score of HBM4EU and AMAP—control charts of *z*-score values obtained in the last 3 years by our laboratory in the interlaboratory comparison exercises organized within the HBM4EU project and the Arctic Monitoring and Assessment Programme (AMAP). Supplementary data 3: AMAP report 2016–2017—reports of the interlaboratory comparison exercises organized by the Arctic Monitoring and Assessment Programme (AMAP) in 2016–2017.
