# Supplementary material for: A Simple and Rapid Method for Quantitative HPLC MS/MS Determination of Selected Perfluorocarboxylic Acids and Perfluorosulfonates in Human Serum
Source: Int J Anal Chem. 2020 Oct 16;2020:8878618. doi: 10.1155/2020/8878618 (PMC7585657; doi:10.1155/2020/8878618)
Supplement: Supplementary Materials — Supplementary data 1: quality control sample charts—quality control charts of PFASs concentrations in the quality control sample were analyzed and collected in our laboratory in the last 5 years. Supplementary data 2: control charts' z-score of HBM4EU and AMAP—control charts of z-score values obtained in the last 3 years by our laboratory in the interlaboratory comparison exercises organized within the HBM4EU project and the Arctic Monitoring and Assessment Programme (AMAP). Supplementary data 3: AMAP report 2016–2017—reports of the interlaboratory comparison exercises organized by the Arctic Monitoring and Assessment Programme (AMAP) in 2016–2017. [file 8878618.f1.zip › 8878618.f1/Supplementary Data 1. Quality control sample charts.pdf]

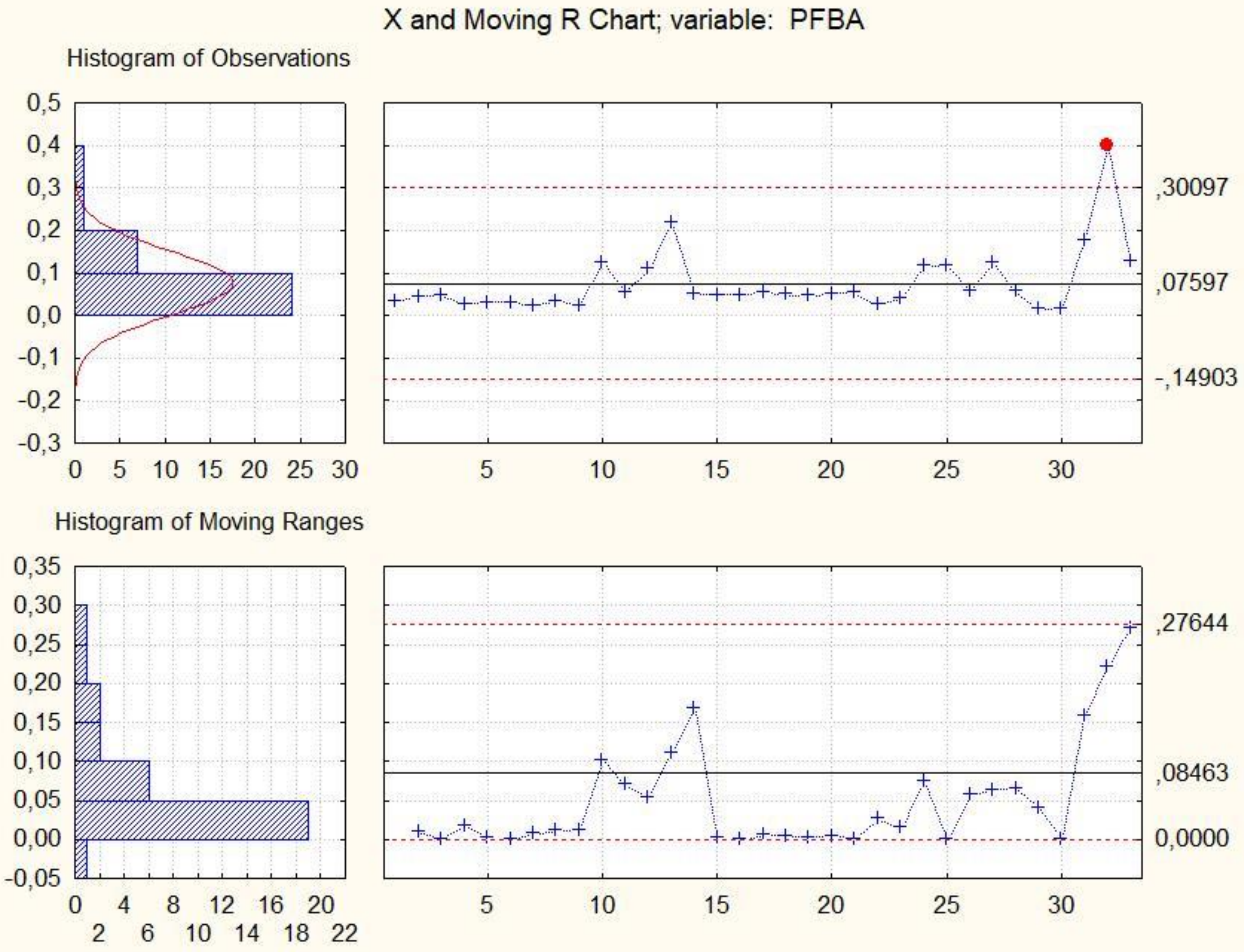

X and Moving R Chart; variable: PFBA
Histogram of Observations
Histogram of Moving Ranges
,30097
,07597
-,14903
,27644
,08463
0,0000

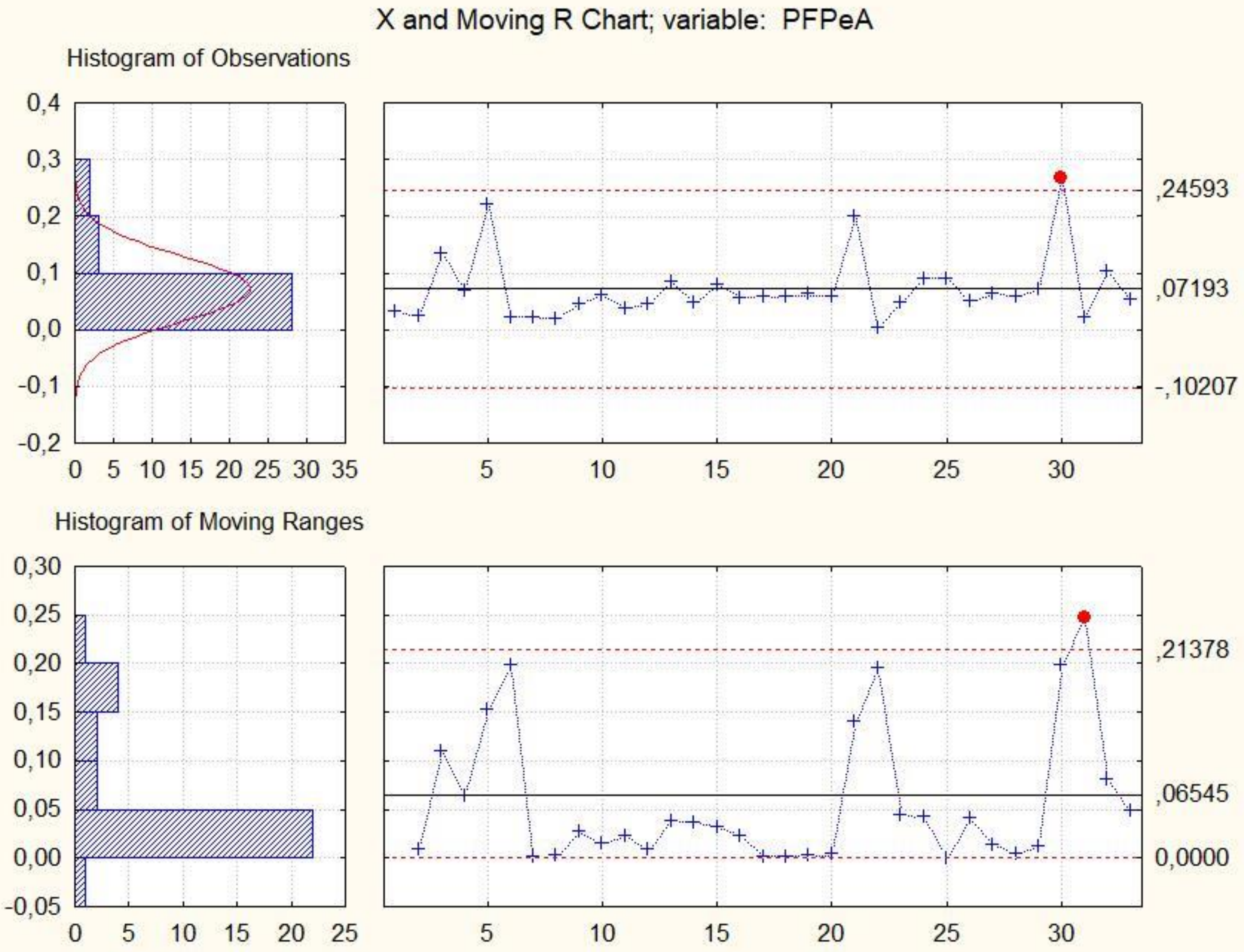
X and Moving R Chart; variable: PFPeA
Histogram of Observations
Histogram of Moving Ranges
,24593
,07193
-,10207
,21378
,06545
0,0000

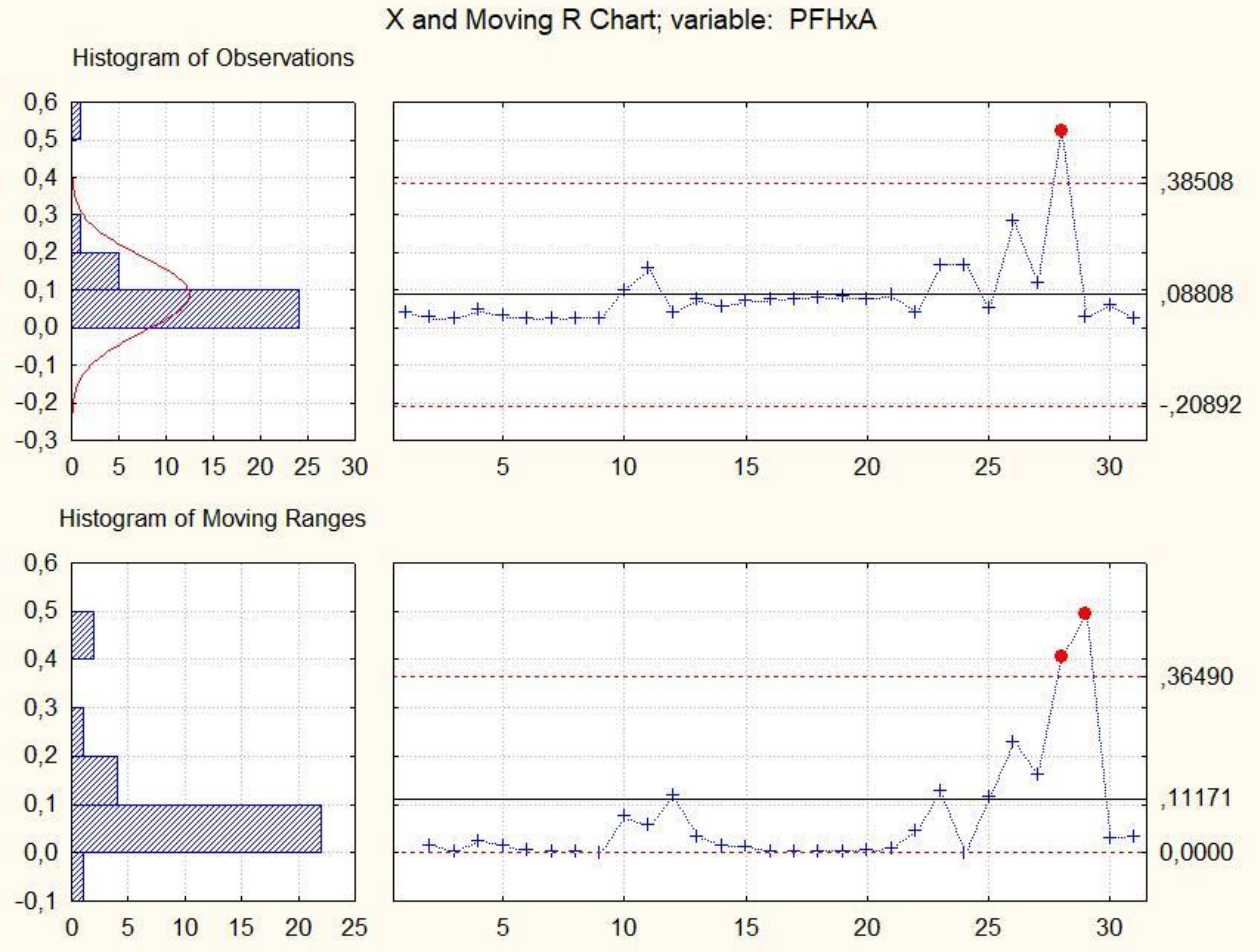
X and Moving R Chart; variable: PFHxA
Histogram of Observations
Histogram of Moving Ranges
,38508
,08808
-,20892
,36490
,11171
0,0000

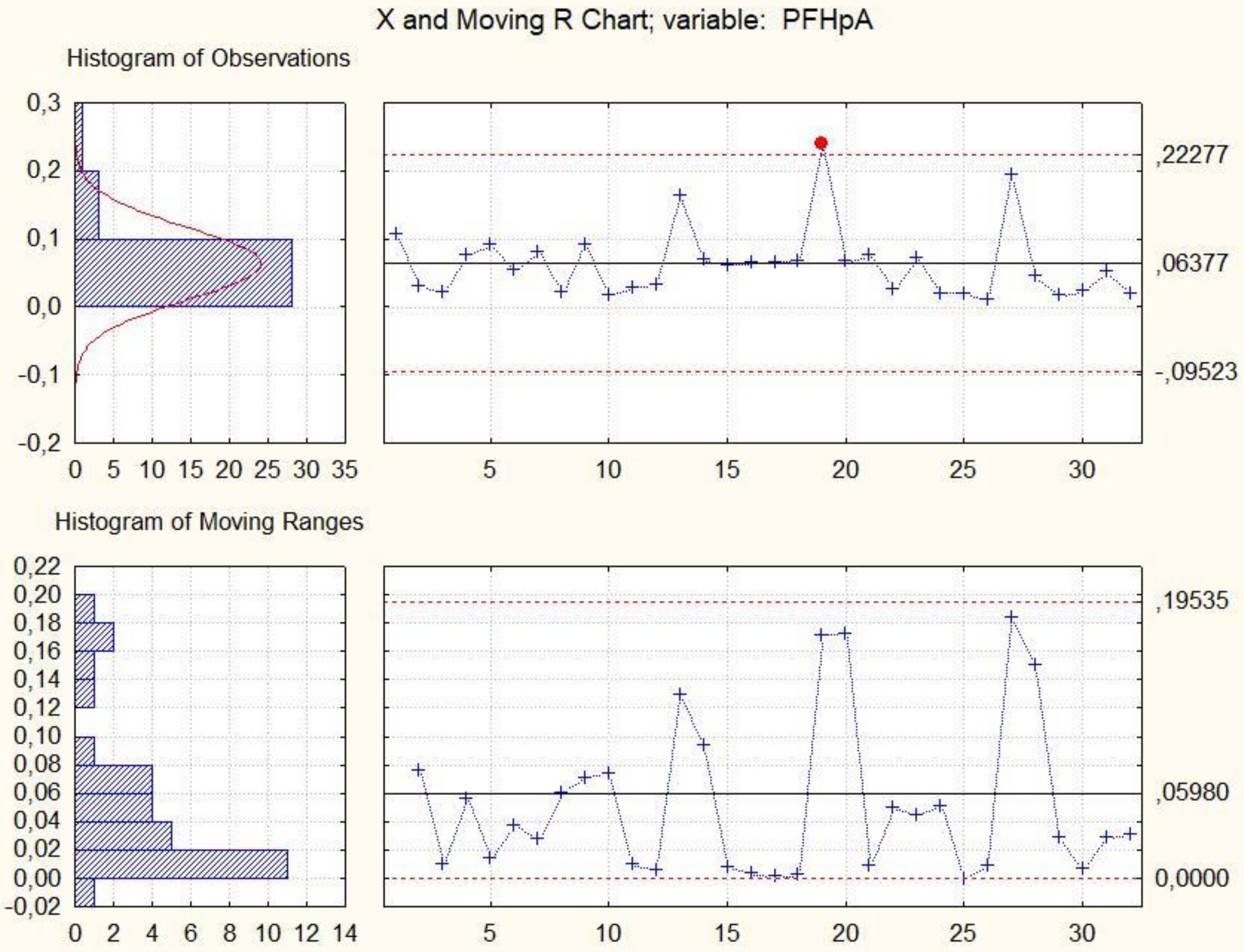
X and Moving R Chart; variable: PFHpA
Histogram of Observations
0,3
0,2
0,1
0,0
-0,1
-0,2
0 5 10 15 20 25 30 35
,22277
,06377
-,09523
5 10 15 20 25 30
Histogram of Moving Ranges
0,22
0,20
0,18
0,16
0,14
0,12
0,10
0,08
0,06
0,04
0,02
0,00
-0,02
0 2 4 6 8 10 12 14
,19535
,05980
0,0000
5 10 15 20 25 30

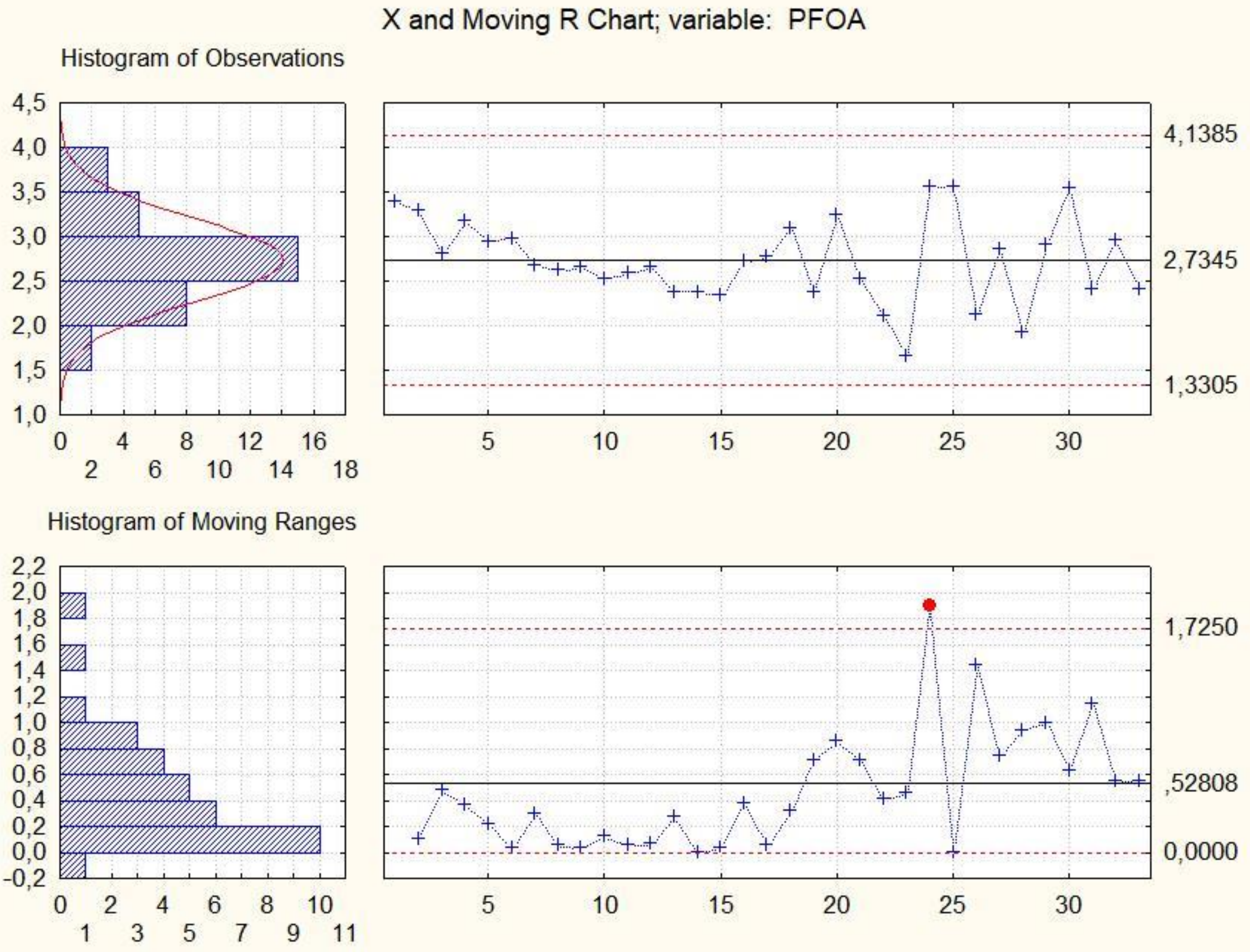
X and Moving R Chart; variable: PFOA
Histogram of Observations
Histogram of Moving Ranges
4,1385
2,7345
1,3305
1,7250
,52808
0,0000

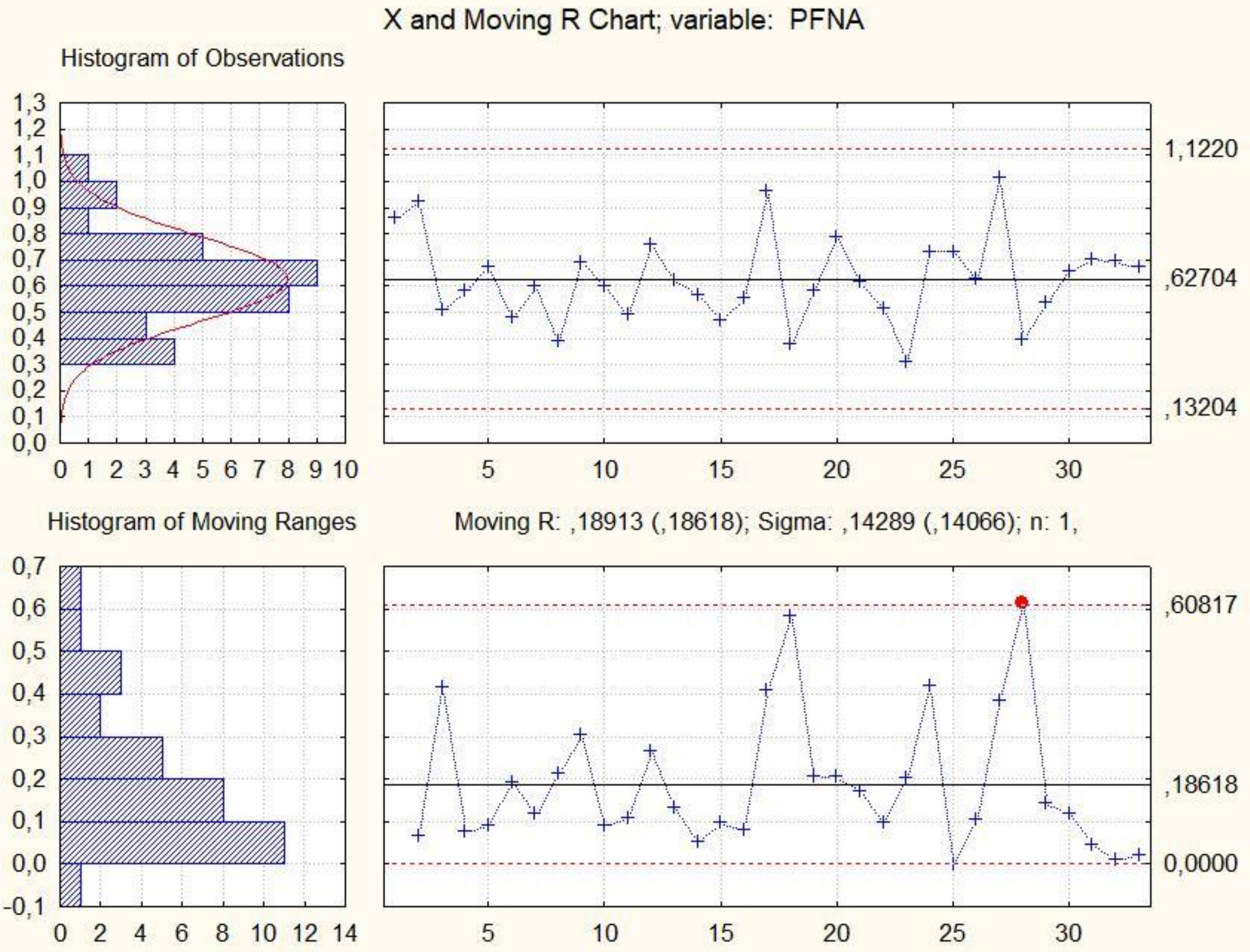

X and Moving R Chart; variable: PFNA
Histogram of Observations
1,1220
,62704
,13204
Histogram of Moving Ranges
Moving R: ,18913 (,18618); Sigma: ,14289 (,14066); n: 1,
,60817
,18618
0,0000

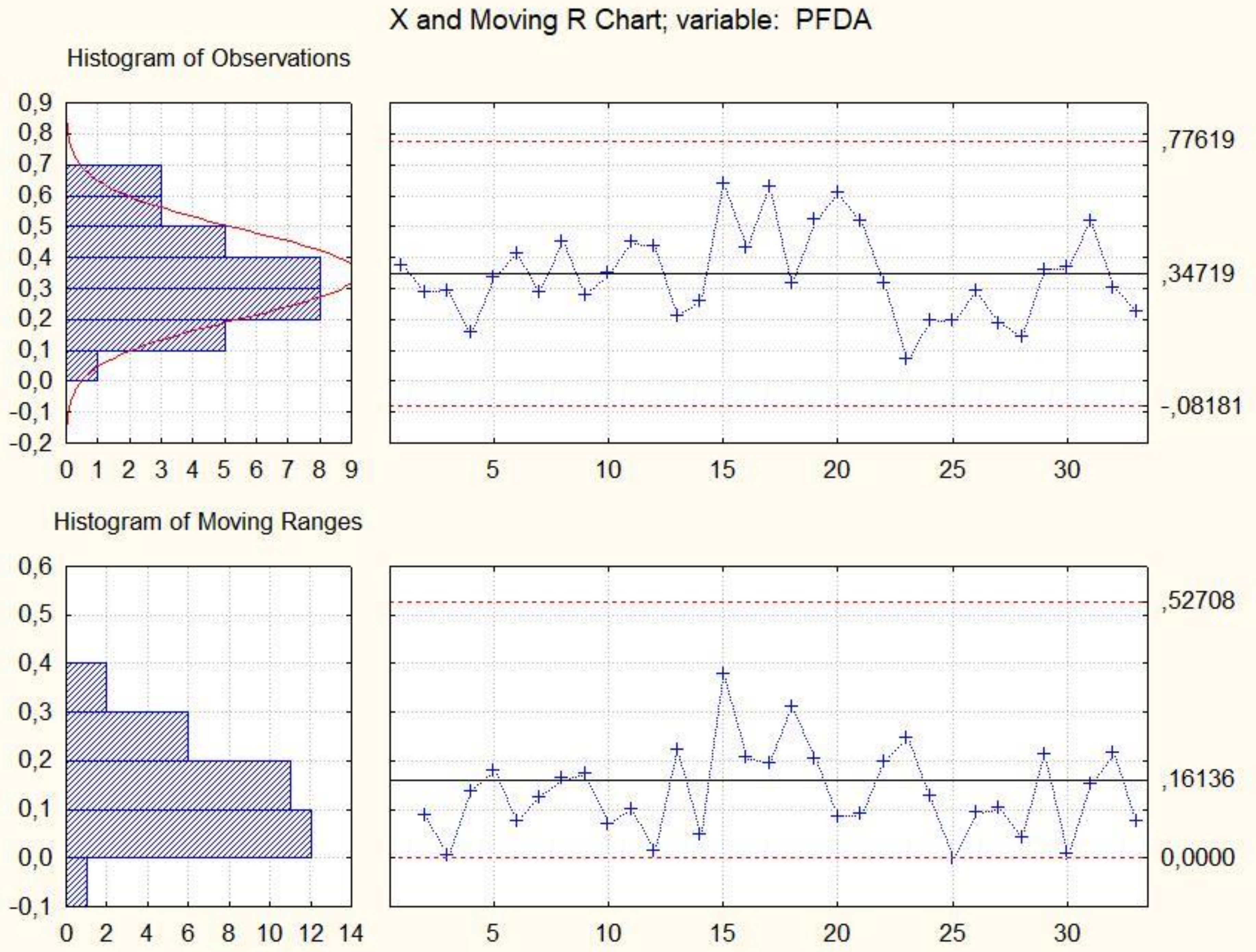
X and Moving R Chart; variable: PFDA
Histogram of Observations
Histogram of Moving Ranges
,77619
,34719
-,08181
,52708
,16136
0,0000

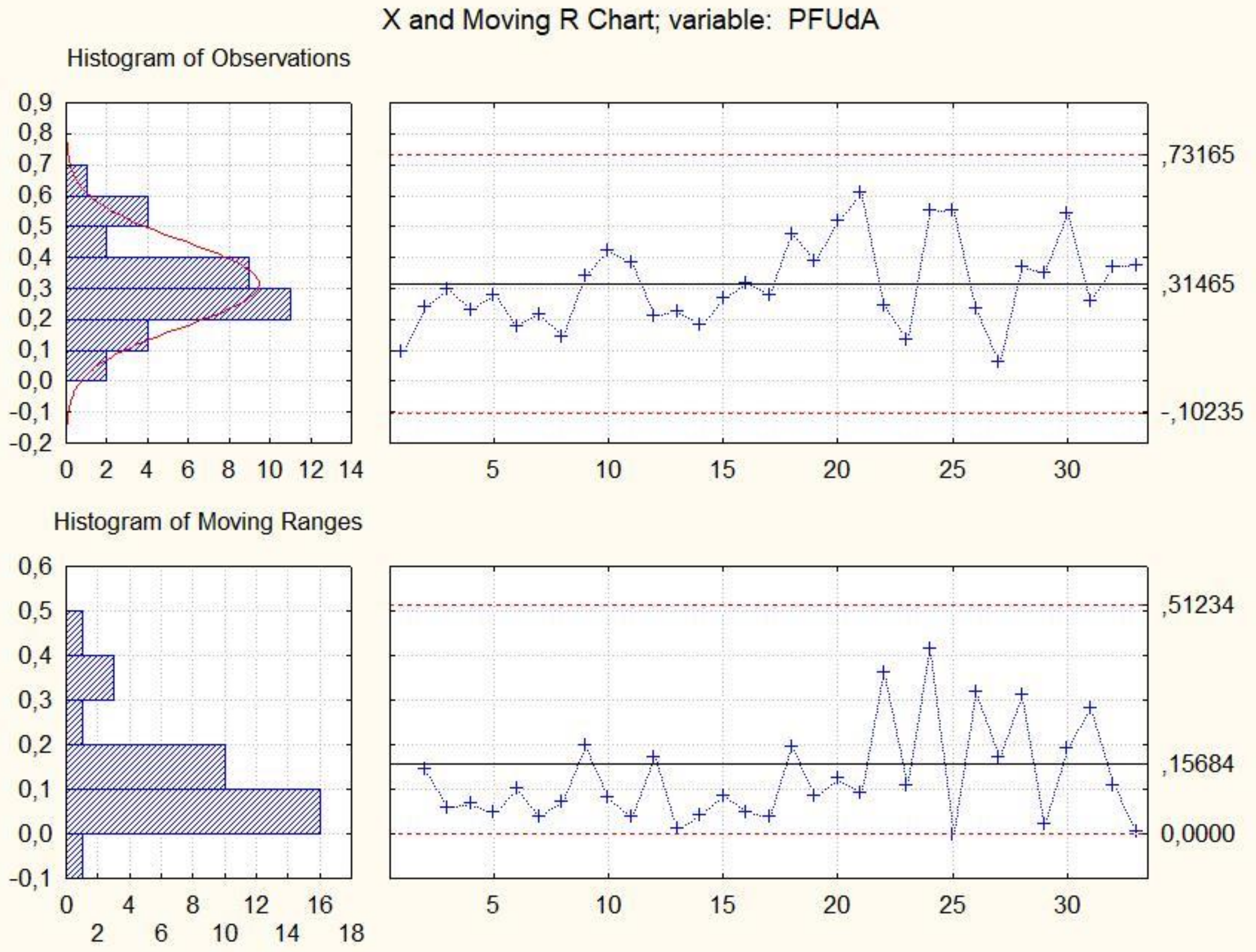
X and Moving R Chart; variable: PFUdA
Histogram of Observations
Histogram of Moving Ranges
,73165
,31465
-,10235
,51234
,15684
0,0000

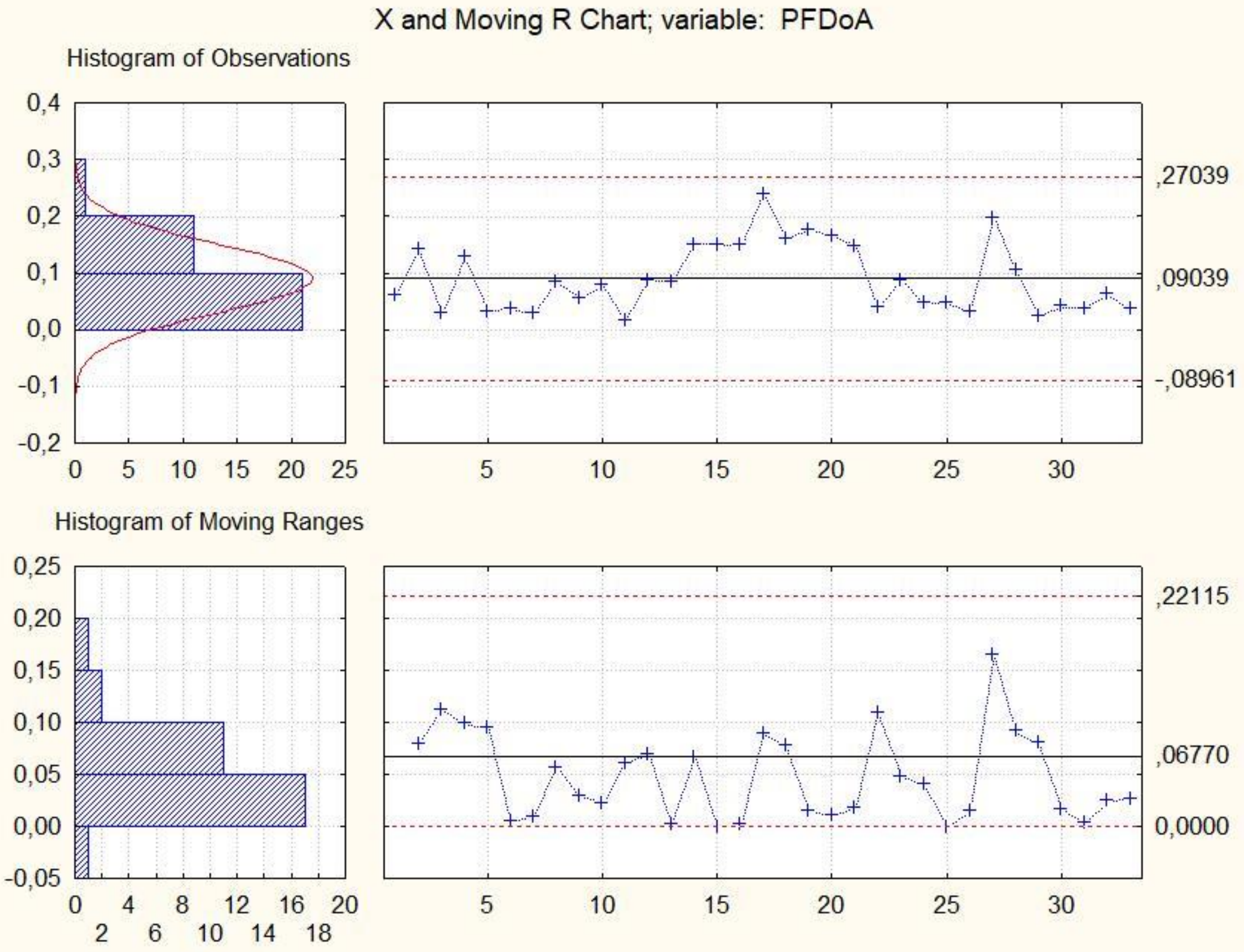
X and Moving R Chart; variable: PFDoA
Histogram of Observations
Histogram of Moving Ranges
,27039
,09039
-,08961
,22115
,06770
0,0000
0,4
0,3
0,2
0,1
0,0
-0,1
-0,2
0,25
0,20
0,15
0,10
0,05
0,00
-0,05
0 5 10 15 20 25
0 2 4 6 8 10 12 14 16 18 20
5 10 15 20 25 30

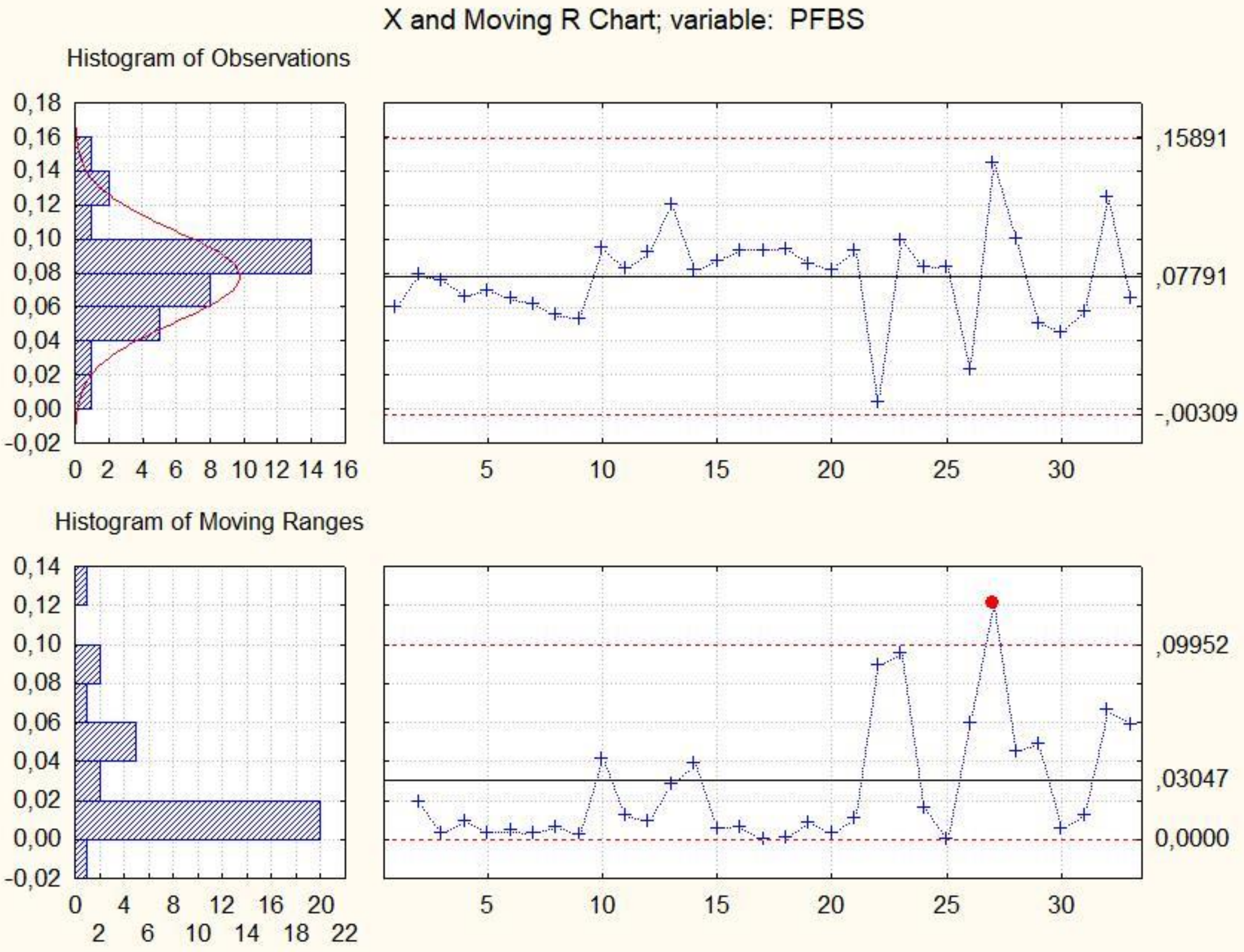

X and Moving R Chart; variable: PFBS
Histogram of Observations
Histogram of Moving Ranges
,15891
,07791
-,00309
,09952
,03047
0,0000
0,18
0,16
0,14
0,12
0,10
0,08
0,06
0,04
0,02
0,00
-0,02
0 2 4 6 8 10 12 14 16
0 4 8 12 16 20
2 6 10 14 18 22
5 10 15 20 25 30

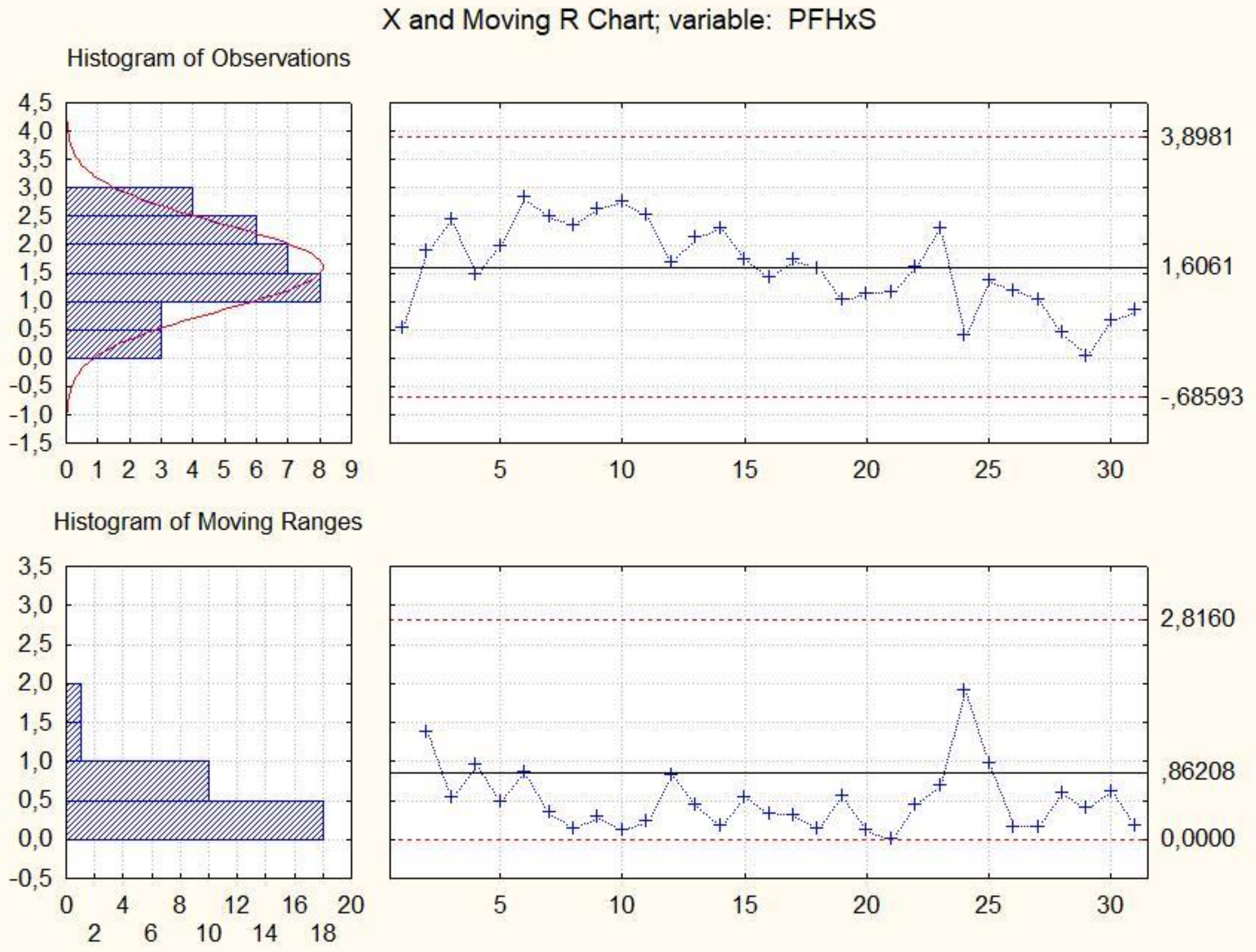
X and Moving R Chart; variable: PFHxS
Histogram of Observations
Histogram of Moving Ranges
3,8981
1,6061
-,68593
2,8160
,86208
0,0000

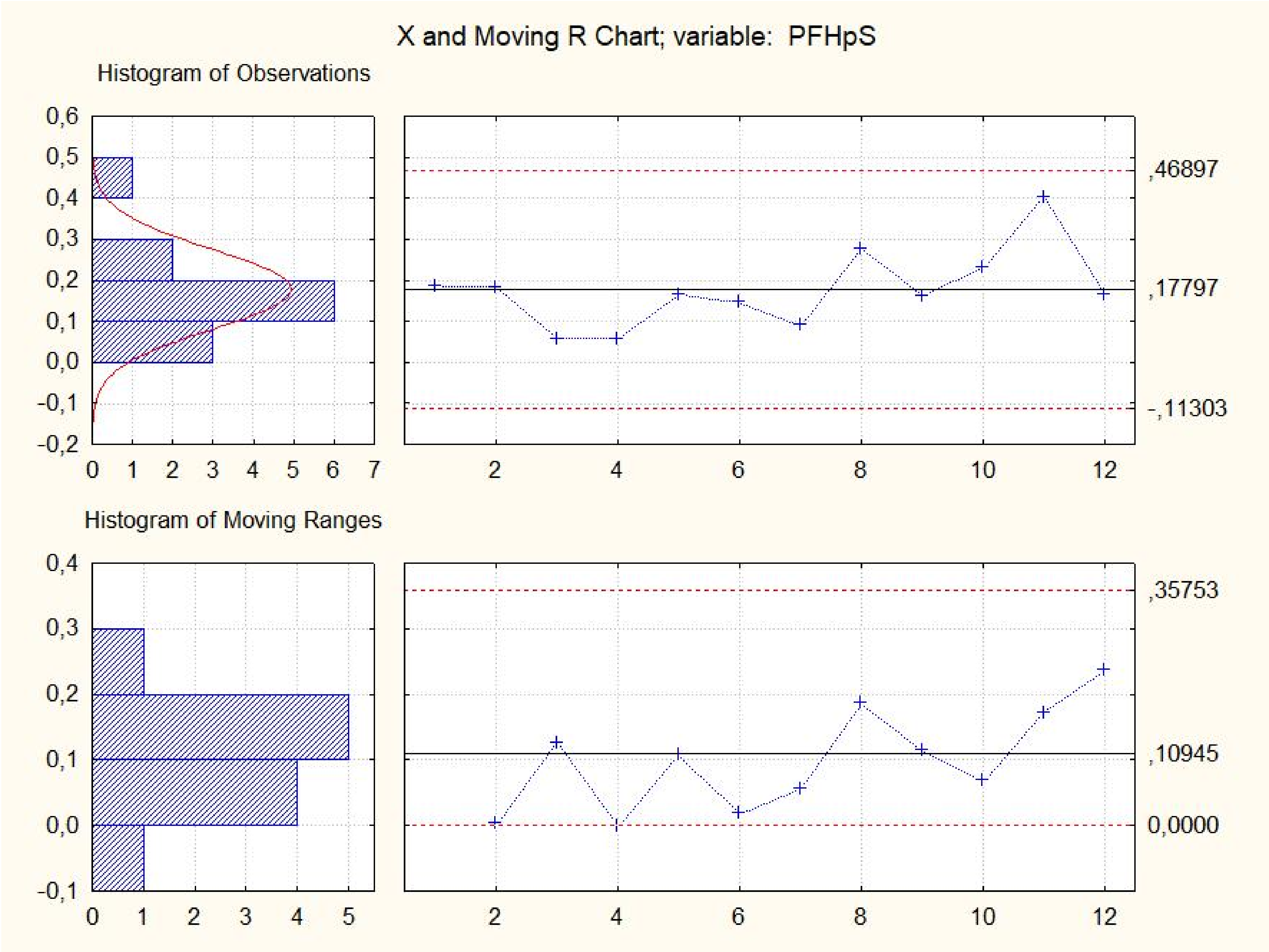
X and Moving R Chart; variable: PFHpS
Histogram of Observations
0,6
0,5
0,4
0,3
0,2
0,1
0,0
-0,1
-0,2
0 1 2 3 4 5 6 7
,46897
,17797
-,11303
2 4 6 8 10 12
Histogram of Moving Ranges
0,4
0,3
0,2
0,1
0,0
-0,1
0 1 2 3 4 5
,35753
,10945
0,0000
2 4 6 8 10 12

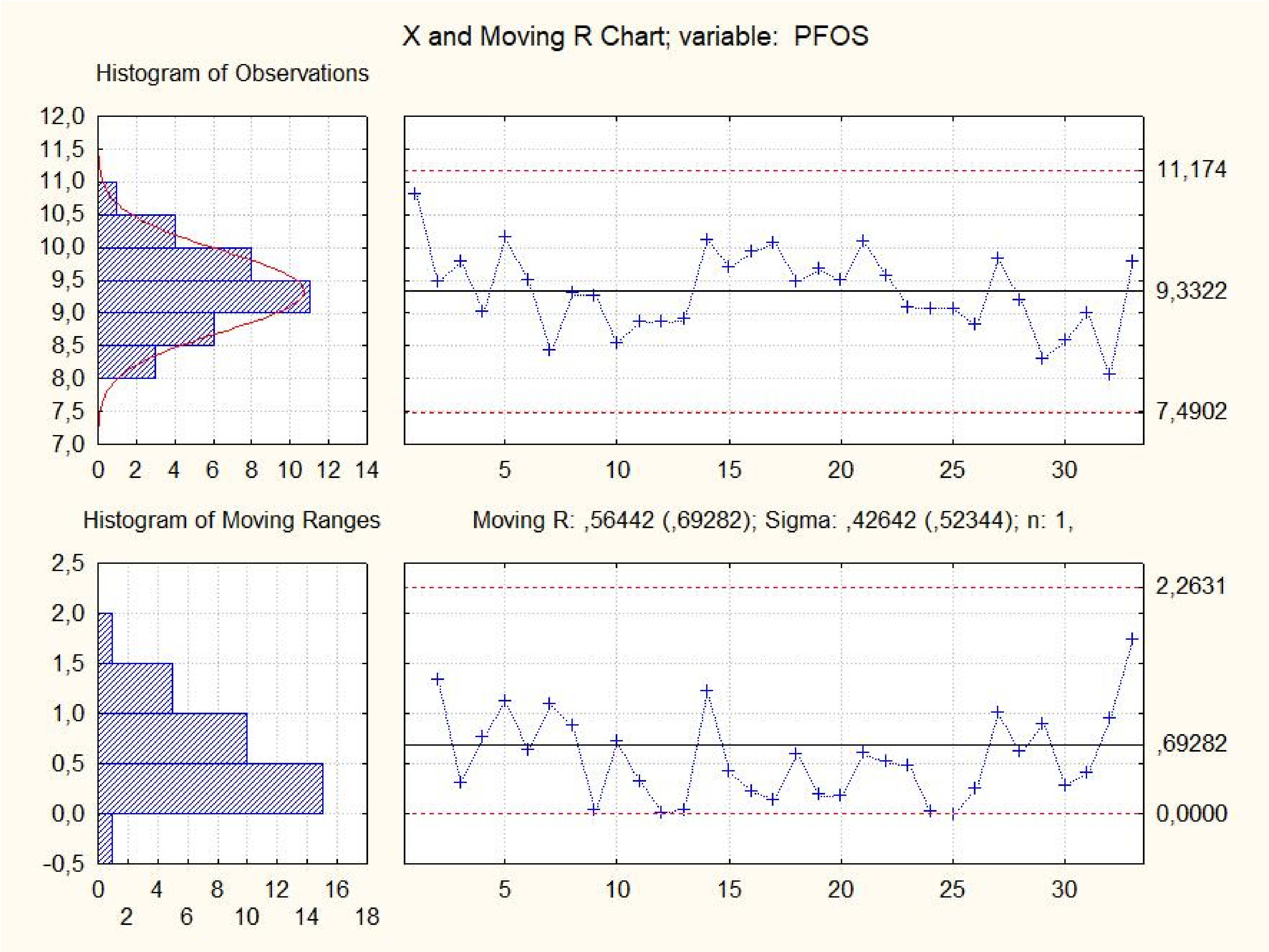
X and Moving R Chart; variable: PFOS
Histogram of Observations
12,0
11,5
11,0
10,5
10,0
9,5
9,0
8,5
8,0
7,5
7,0
0 2 4 6 8 10 12 14
5 10 15 20 25 30
11,174
9,3322
7,4902
Histogram of Moving Ranges
Moving R: ,56442 (,69282); Sigma: ,42642 (,52344); n: 1,
2,5
2,0
1,5
1,0
0,5
0,0
-0,5
0 4 8 12 16
2 6 10 14 18
2,2631
,69282
0,0000
