# Supplementary material for: A Simple and Rapid Method for Quantitative HPLC MS/MS Determination of Selected Perfluorocarboxylic Acids and Perfluorosulfonates in Human Serum
Source: Int J Anal Chem. 2020 Oct 16;2020:8878618. doi: 10.1155/2020/8878618 (PMC7585657; doi:10.1155/2020/8878618)
Supplement: Supplementary Materials — Supplementary data 1: quality control sample charts—quality control charts of PFASs concentrations in the quality control sample were analyzed and collected in our laboratory in the last 5 years. Supplementary data 2: control charts' z-score of HBM4EU and AMAP—control charts of z-score values obtained in the last 3 years by our laboratory in the interlaboratory comparison exercises organized within the HBM4EU project and the Arctic Monitoring and Assessment Programme (AMAP). Supplementary data 3: AMAP report 2016–2017—reports of the interlaboratory comparison exercises organized by the Arctic Monitoring and Assessment Programme (AMAP) in 2016–2017. [file 8878618.f1.zip › 8878618.f1/Supplementary data 3.Report AMAP 2016-2017.pdf]

Institut national de santé publique Québec
Centre de toxicologie

AMAP RING TEST FOR PERSISTENT ORGANIC POLLUTANTS IN HUMAN SERUM (AMAP)

REPORT FOR ROUND: **2016-01**

PTMS SHIPPING DATE: **2016-01-25**

DATE OF PUBLICATION: **2016-04-05**

SCC Accredited PTP
PSEA Accrédité CCN TM

Scope of accreditation: Total cholesterol, total lipids, triglycerides, PCB IUPAC #52, PCB IUPAC #101, PCB IUPAC #183 and PCB IUPAC #187 are not included in our scope of accreditation.
The assigned value obtained for PCB IUPAC #74 in material AM-S-W1602 exceeds the range of concentration from our scope of accreditation.

Centre de toxicologie/INSPQ
945, Ave Wolfe
Québec, QC
Canada, G1V 5B3
Phone: (418) 650-5115 Ext. 5254
Fax : (418) 654-2148
E-mail : ctq@inspq.qc.ca
http://www.inspq.qc.ca/ctq

TABLE OF CONTENTS

INTRODUCTION 1

SÉRUM

Dieldrin 2

ß-HCH 5

Heptachlor 8

Heptachlor epoxide 11

Hexachlorobenzene 14

Oxychlordane 17

p,p'-DDE 20

p,p'-DDT 23

PBDE IUPAC # 28 26

PBDE IUPAC # 47 29

PBDE IUPAC # 99 32

PBDE IUPAC # 100 35

PBDE IUPAC # 153 38

PBDE IUPAC # 154 41

PBDE IUPAC # 183 44

PBDE IUPAC # 209 47

PCB IUPAC # 28 50

PCB IUPAC # 52 53

PCB IUPAC # 74 56

PCB IUPAC # 99 59

PCB IUPAC # 101 62

PCB IUPAC # 105 65

PCB IUPAC # 118 68

PCB IUPAC # 138 71

PCB IUPAC # 153 74

PCB IUPAC # 170 77

PCB IUPAC # 180 80
PCB IUPAC # 183 83
PCB IUPAC # 187 86
PFHxA 89
PFHxS 92
PFNA 95
PFOA 98
PFOS 101
PFUdA 104
Total Cholesterol 107
Total lipids 110
Toxaphene Parlar # 26 113
Toxaphene Parlar # 50 116
trans-nonachlor 119
Triglycerides 122
ASSIGNED VALUES 125
GROUPING OF ANALYTICAL METHODS FOR STATISTICS 127
END OF REPORT 128

945, Ave Wolfe
Quebec, QC
Canada, G1V 5B3
Phone: (418) 650-5115 Ext. 5254
Fax : (418) 654-2148
E-mail : ctq@inspq.qc.ca
http://www.inspq.qc.a/ctq

INTRODUCTION

Dear AMAP participants:

This report includes the results and performance evaluations of round 2016-01.

Participating laboratories are identified only by their unique subscription number. Identity of participants will be kept strictly confidential by the AMAP organizer.

For this PT exercise, all analytes meet the homogeneity criteria as per ISO/CEI 17043 and 13528 guidelines.

A study had been previously performed to demonstrate that all the analytes are stable for the duration of the PT exercise and meet the stability criteria according to ISO/CEI 17043 and 13528 guidelines.

Please note that the appendices containing statistical approaches are no longer included in the reports. You will find them within the "Participant's Guide".

We are available to assist you at any time. If you have any questions or concerns regarding our program, do not hesitate to contact us. Your comments help us enhance the quality of our schemes.

Best regards,

David Bisson, M.Sc. Chemist
Programs Coordinator
External Quality Assessment Schemes and Reference Materials

Centre de toxicologie du Québec / INSPQ
Tel.: (418) 650-5115, extension 4649
E-mail: david.bisson@inspq.qc.ca
Web Site: www.inspq.qc.ca/CTQ/paqe

Individual results

Serum Dieldrin (µg/L)

Round #2016-01

Participant	AM-S-W1601	z'-score	AM-S-W1602	z'-score	AM-S-W1603	z'-score	Method
**1042**	0.0783	---	1.45	-0.79	0.671	0.27	GC-MS CI
**2446**	0.08	---	1.65	0.09	0.600	-0.43	ND
**4336**	<LQ	---	1.95	1.41	0.760	1.14	GC-MS-MS
**8559**	<LD	---	1.58	-0.22	0.543	-0.99	GC

	Assigned value	Standard uncertainty	σ pt	Acceptable range	K-S (Lilliefors)	Species
**AM-S-W1601**	---	---	---	---	---	---
**AM-S-W1602**	1.63	0.115	0.196	1.18 - 2.08	Accepted	---
**AM-S-W1603**	0.644	0.0664	0.0772	0.440 - 0.848	Accepted	---

Due to the low number of data, the results of dieldrin in material AM-S-W1601 are not presented in this report.

Statistics
Serum Dieldrin (µg/L)

All methods	AM-S-W1601	AM-S-W1602	AM-S-W1603
**N**	2	4	4
**Robust mean Algo A**	---	1.63	0.644
**Robust STDev**	---	0.183	0.106
**Median**	----	1.62	0.636
**STDev from MAD**	----	0.148	0.0950
**Arithmetic mean**	----	1.66	0.644
**STDev**	----	0.212	0.0937
**Comparison AM-S-W1505**	0.0899		
	0.00262		
**CV or Variability**	---	11.3%	16.5%

When fewer than 20 results were considered for statistical treatment of all or a sub-sample of results, the accuracy of statistical data may be questionable.

Distribution

Serum Dieldrin (µg/L)

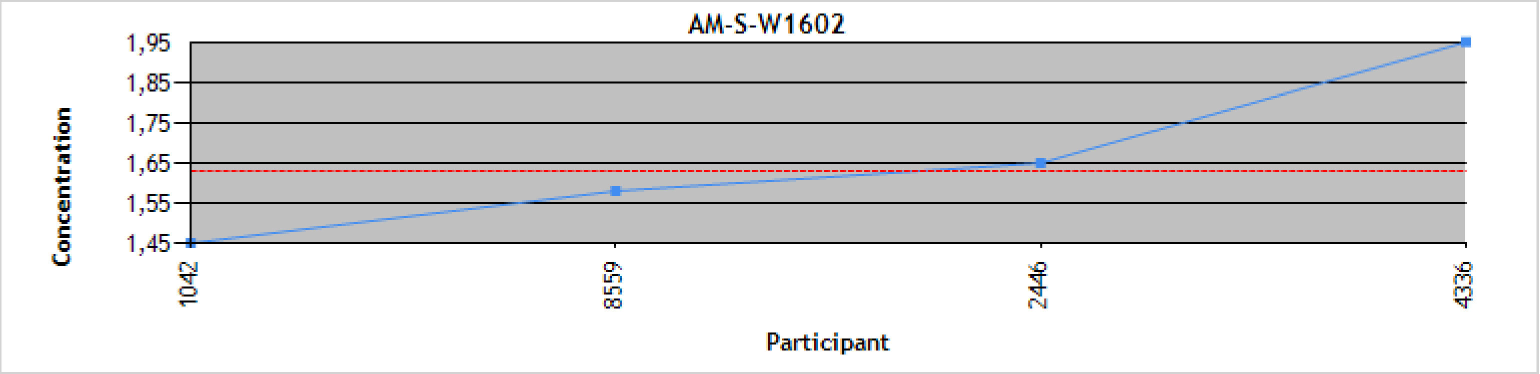

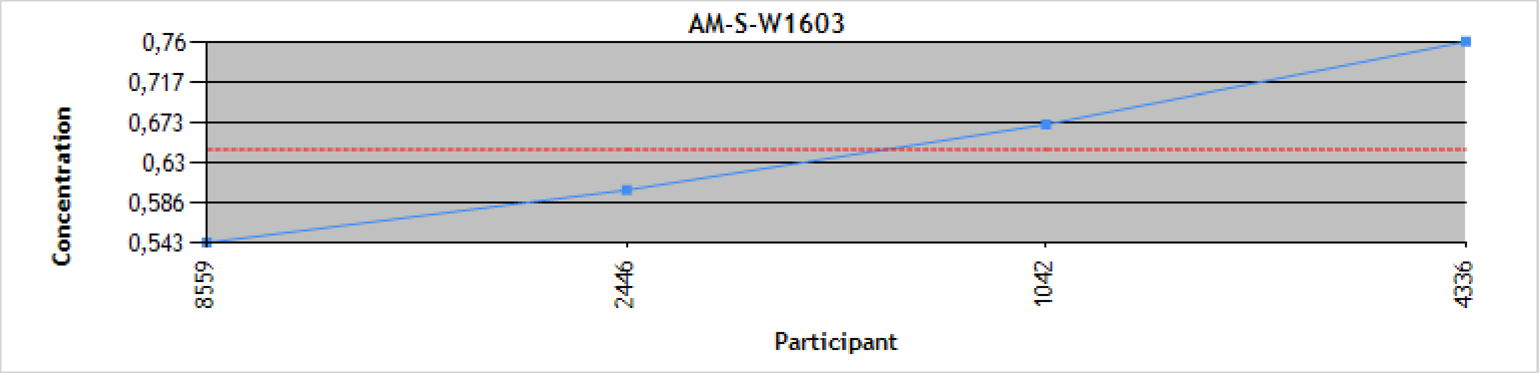

Individual results

Serum ß-HCH (µg/L)

Round #2016-01

Participant	AM-S-W1601	z'-score	AM-S-W1602	z'-score	AM-S-W1603	z'-score	Method
748	1.61	0.45	0.881	0.01	1.67	-0.40	GC
1003	1.46	-0.11	0.814	-0.41	1.74	-0.19	GC-MS-MS
1042	1.63	0.54	0.861	-0.12	1.89	0.29	GC-MS CI
1629	1.32	-0.63	1.10	1.35	2.21	1.27	GC-MS-MS
2446	1.52	0.11	0.990	0.68	1.95	0.46	ND
3112	1.29	-0.75	0.749	-0.81	1.45	-1.08	GC-MS CI
4336	1.99	1.87	1.10	1.37	2.28	1.48	GC-MS-MS
4635	1.27	-0.81	0.695	-1.15	1.50	-0.94	GC-MS EI
6865	1.60	0.41	0.911	0.19	1.85	0.15	ND
7660	1.49	0.00	0.824	-0.34	1.72	-0.25	GC-MS CI
8559	1.15	-1.27	0.665	-1.33	1.51	-0.90	ND
8916	1.26	-0.85	0.761	-0.74	1.55	-0.77	GC
9301	1.96	1.75	1.09	1.30	2.32	1.61	GC-MS-MS

	Assigned value	Standard uncertainty	σ pt	Acceptable range	K-S (Lilliefors)	Species
AM-S-W1601	1.49	0.0894	0.252	0.955 - 2.03	Accepted	---
AM-S-W1602	0.880	0.0593	0.150	0.558 - 1.20	Accepted	---
AM-S-W1603	1.80	0.109	0.306	1.15 - 2.45	Accepted	---

Statistics
Serum ß-HCH (µg/L)

All methods	AM-S-W1601	AM-S-W1602	AM-S-W1603
N	13	13	13
Robust mean Algo A	1.49	0.880	1.80
Robust STDev	0.258	0.171	0.313
Median	1.49	0.861	1.74
STDev from MAD	0.252	0.166	0.311
Arithmetic mean	1.50	0.880	1.82
STDev	0.258	0.151	0.301
Comparison AM-S-W1505	1.52		
	0.207		
CV or Variability	17.3%	19.4%	17.4%

GC-MS CI	AM-S-W1601	AM-S-W1602	AM-S-W1603
N	3	3	3
Robust mean Algo A	1.47	0.811	1.69
Robust STDev	0.196	0.0646	0.252
Median	1.49	0.824	1.72
STDev from MAD	0.213	0.0536	0.259
Arithmetic mean	1.47	0.811	1.69
STDev	0.173	0.0569	0.222
CV or Variability	13.3%	8.0%	14.9%

GC-MS-MS	AM-S-W1601	AM-S-W1602	AM-S-W1603
N	4	4	4
Robust mean Algo A	1.68	1.09	2.23
Robust STDev	0.389	0.00891	0.0973
Median	1.71	1.09	2.25
STDev from MAD	0.393	0.00742	0.0808
Arithmetic mean	1.68	1.03	2.14
STDev	0.343	0.141	0.269
CV or Variability	23.1%	0.8%	4.4%

When fewer than 20 results were considered for statistical treatment of all or a sub-sample of results, the accuracy of statistical data may be questionable.

Distribution

Serum ß-HCH (µg/L)

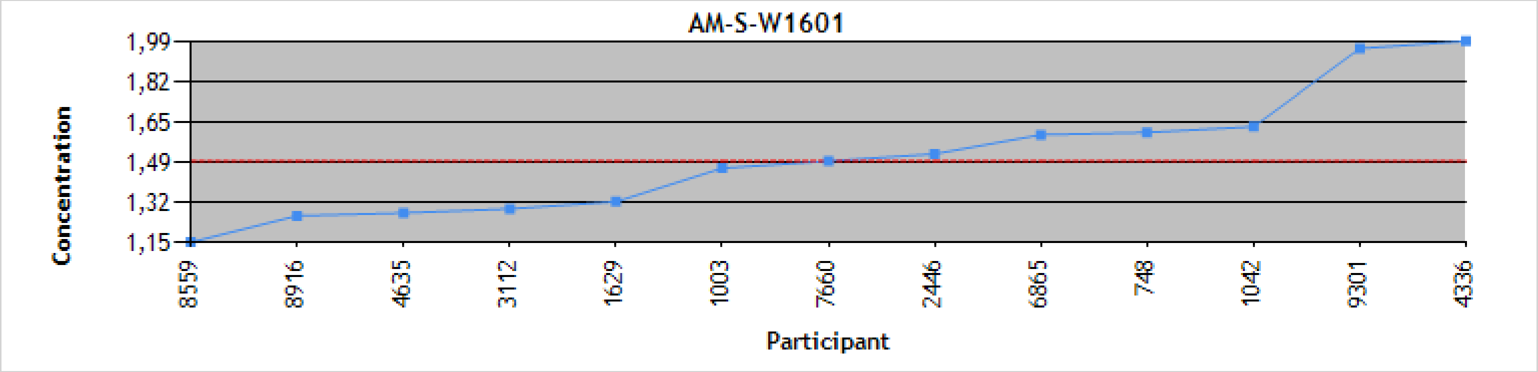

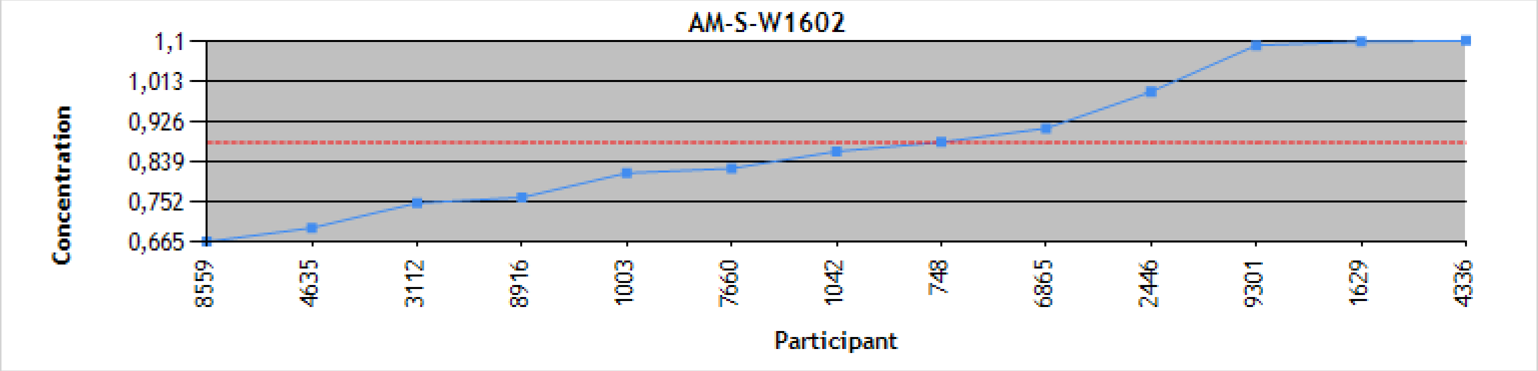

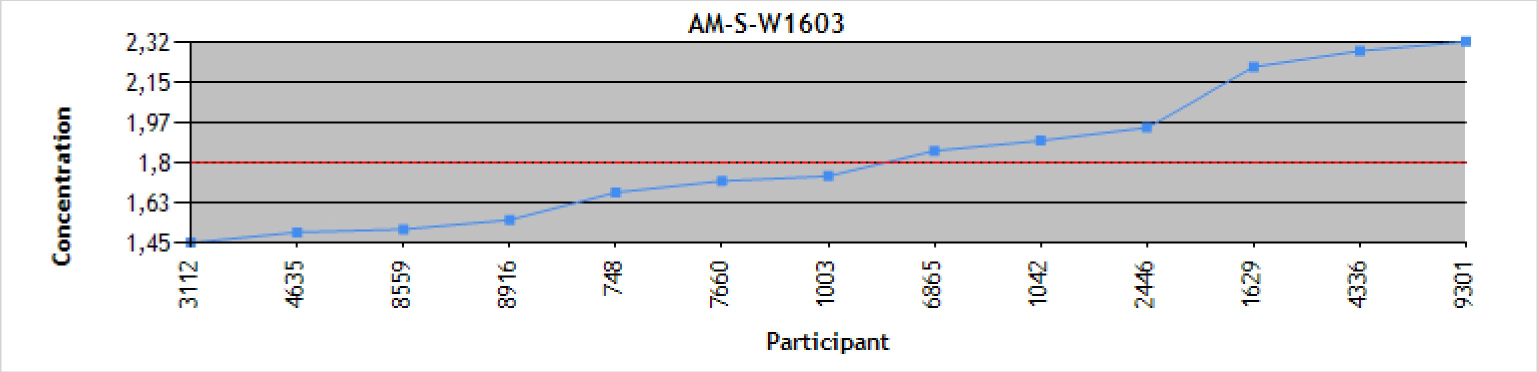

Individual results

Serum Heptachlor (µg/L)

Round #2016-01

Participant	AM-S-W1601	z'-score	AM-S-W1602	z'-score	AM-S-W1603	z'-score	Method
**1042**	1.21	0.22	1.46	0.32	0.768	0.00	GC-MS CI
**2446**	1.14	-0.08	1.34	-0.11	0.770	0.01	ND
**4336**	1.20	0.17	1.44	0.25	0.830	0.39	GC-MS-MS
**8559**	0.836	-1.38	1.16	-0.74	0.705	-0.40	ND

	Assigned value	Standard uncertainty	σ pt	Acceptable range	K-S (Lilliefors)	Species
**AM-S-W1601**	1.16	0.0402	0.232	0.689 - 1.63	Accepted	---
**AM-S-W1602**	1.37	0.0663	0.275	0.805 - 1.94	Accepted	---
**AM-S-W1603**	0.768	0.0362	0.154	0.452 - 1.08	Accepted	---

Statistics

Serum Heptachlor (µg/L)

All methods	AM-S-W1601	AM-S-W1602	AM-S-W1603
**N**	4	4	4
**Robust mean Algo A**	1.16	1.37	0.768
**Robust STDev**	0.0644	0.106	0.0579
**Median**	1.17	1.39	0.769
**STDev from MAD**	0.0536	0.0882	0.0459
**Arithmetic mean**	1.10	1.35	0.768
**STDev**	0.177	0.137	0.0510
**Comparison AM-S-W1505**	1.21		
	0.270		
**CV or Variability**	5.5%	7.7%	7.5%

When fewer than 20 results were considered for statistical treatment of all or a sub-sample of results, the accuracy of statistical data may be questionable.

Distribution
Serum Heptachlor (µg/L)

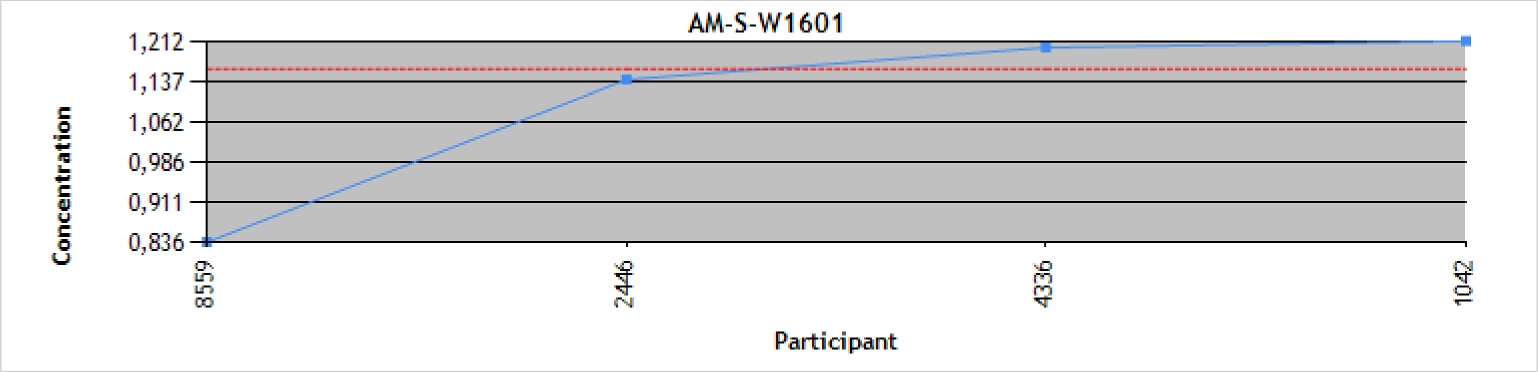

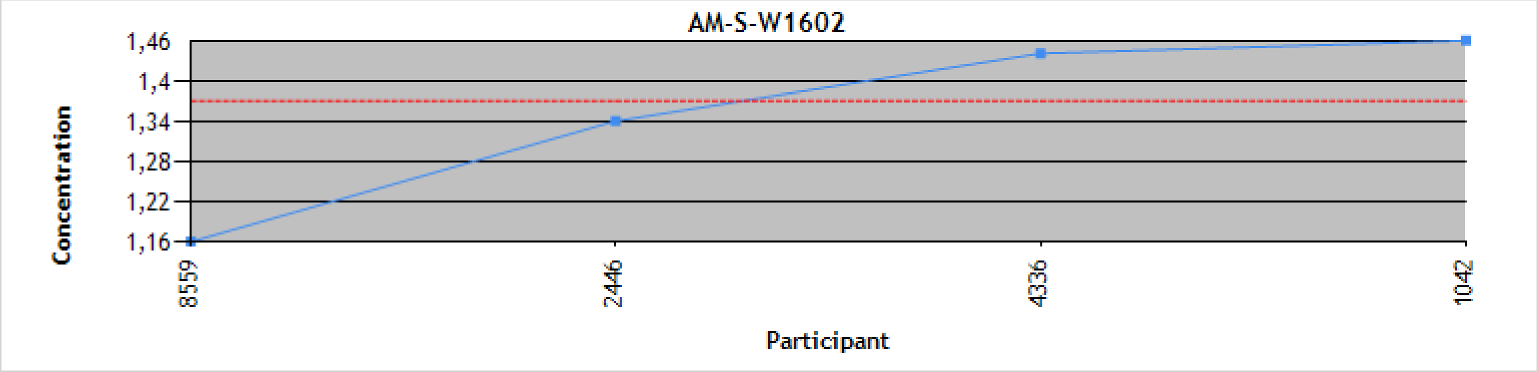

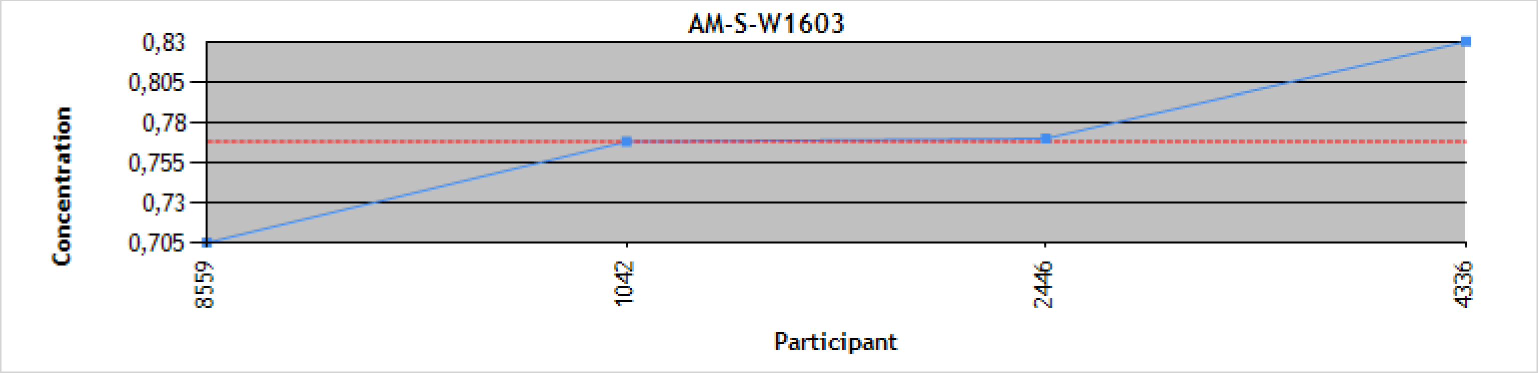

Individual results

Serum Heptachlor epoxide (µg/L)

Round #2016-01

Participant	AM-S-W1601	z'-score	AM-S-W1602	z'-score	AM-S-W1603	z'-score	Method
**1042**	1.68	0.43	1.32	0.70	0.264	0.10	GC-MS CI
**2446**	1.50	-0.28	1.18	-0.05	0.250	-0.25	ND
**4336**	1.66	0.36	1.11	-0.43	0.880	**15.59**	GC-MS-MS
**8559**	1.43	-0.56	1.19	0.00	0.252	-0.20	ND

	Assigned value	Standard uncertainty	σ pt	Acceptable range	K-S (Lilliefors)	Species
**AM-S-W1601**	1.57	0.0859	0.235	1.07 - 2.07	Accepted	---
**AM-S-W1602**	1.19	0.0476	0.178	0.821 - 1.56	Accepted	---
**AM-S-W1603**	0.260	0.00781	0.0390	0.180 - 0.340	Rejected[1]	---

Statistics

Serum Heptachlor epoxide (µg/L)

All methods	AM-S-W1601	AM-S-W1602	AM-S-W1603
N	4	4	4
Robust mean Algo A	1.57	1.19	0.260
Robust STDev	0.138	0.0762	0.0125
Median	1.58	1.19	0.258
STDev from MAD	0.132	0.0593	0.0104
Arithmetic mean	1.57	1.20	0.412
STDev	0.121	0.0876	0.312
Comparison AM-S-W1505	1.47		
	0.180		
CV or Variability	8.8%	6.4%	4.8%

When fewer than 20 results were considered for statistical treatment of all or a sub-sample of results, the accuracy of statistical data may be questionable.

Distribution

Serum Heptachlor epoxide (µg/L)

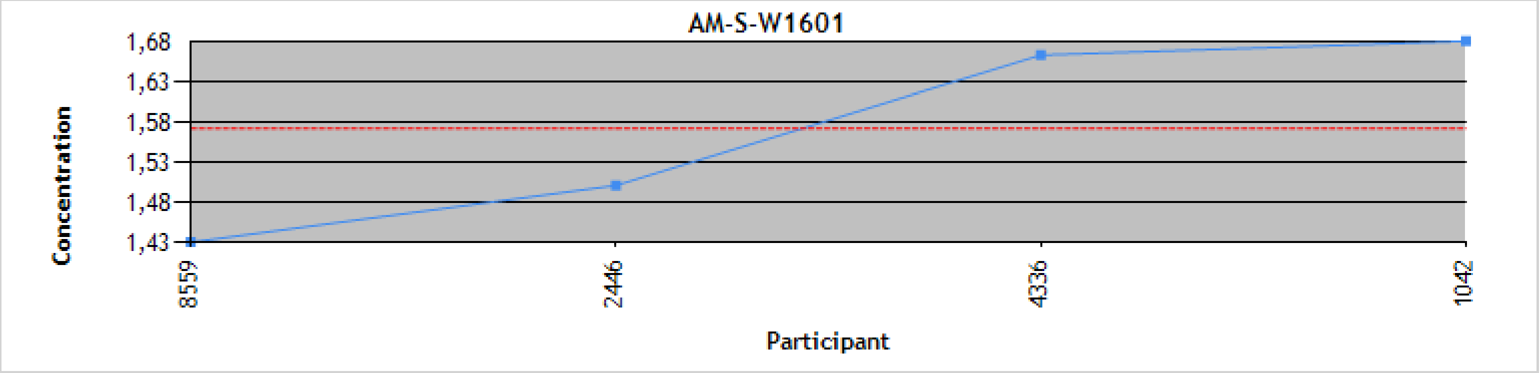

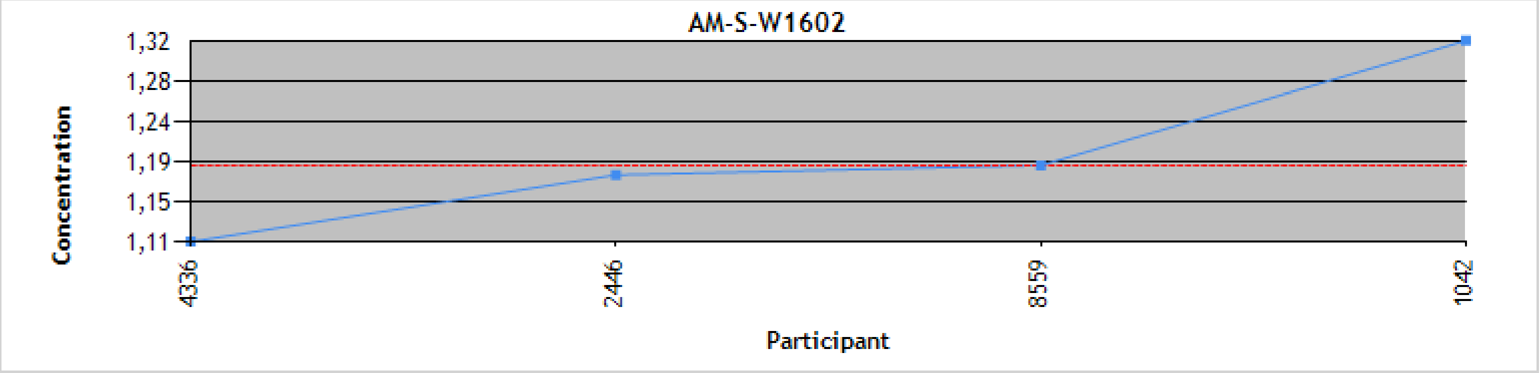

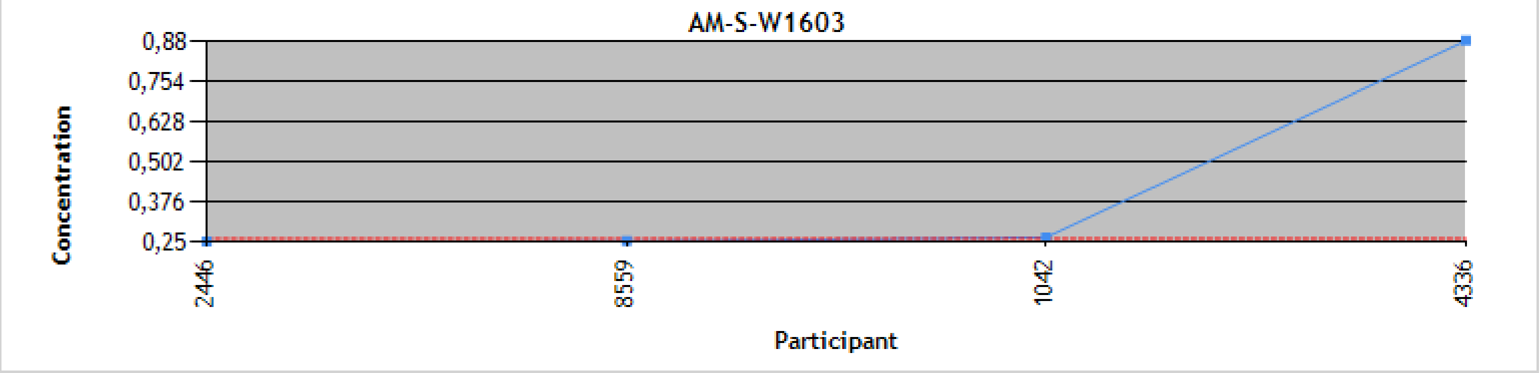

Individual results

Serum Hexachlorobenzene (µg/L)

Round #2016-01

Participant	AM-S-W1601	z'-score	AM-S-W1602	z'-score	AM-S-W1603	z'-score	Method
270	0.984	-1.94	0.732	-1.71	0.492	-1.70	ND
748	1.50	-0.14	1.02	-0.25	0.626	-0.75	GC
1003	1.61	0.24	1.14	0.35	0.753	0.15	GC-MS-MS
1042	1.59	0.17	1.16	0.48	0.774	0.30	GC-MS CI
1629	2.02	1.68	1.50	**2.17**	0.937	1.45	GC-MS-MS
2446	1.57	0.10	1.06	-0.05	0.780	0.34	ND
3112	1.21	-1.16	0.957	-0.57	0.574	-1.12	GC-MS CI
4336	1.61	0.24	0.960	-0.56	0.670	-0.44	GC-MS-MS
6865	1.57	0.10	1.07	0.00	0.734	0.01	GC
7660	1.61	0.25	1.16	0.48	0.767	0.25	GC-MS CI
8559	1.05	-1.71	0.874	-0.99	0.638	-0.66	ND
8916	0.960	**-2.03**	0.705	-1.85	0.466	-1.88	GC
9301	2.19	**2.27**	1.53	**2.33**	1.03	**2.11**	GC-MS-MS
9756	1.86	1.12	1.30	1.17	0.948	1.53	GC-MS-MS
9880	---	---	---	---	0.792	0.42	GC-MS-MS

	Assigned value	Standard uncertainty	σ pt	Acceptable range	K-S (Lilliefors)	Species
AM-S-W1601	1.54	0.106	0.267	0.966 - 2.11	Accepted	---
AM-S-W1602	1.07	0.0636	0.187	0.676 - 1.46	Accepted	---
AM-S-W1603	0.732	0.0559	0.130	0.449 - 1.02	Accepted	---

Statistics
Serum Hexachlorobenzene (µg/L)

All methods	AM-S-W1601	AM-S-W1602	AM-S-W1603
N	14	14	15
Robust mean Algo A	1.54	1.07	0.732
Robust STDev	0.316	0.190	0.173
Median	1.58	1.07	0.753
STDev from MAD	0.267	0.158	0.171
Arithmetic mean	1.52	1.08	0.732
STDev	0.369	0.245	0.161
Comparison AM-S-W1505	1.67		
	0.120		
CV or Variability	20.5%	17.8%	23.7%

GC	AM-S-W1601	AM-S-W1602	AM-S-W1603
N	3	3	3
Robust mean Algo A	1.47	1.00	0.609
Robust STDev	0.131	0.0936	0.153
Median	1.50	1.02	0.626
STDev from MAD	0.104	0.0742	0.160
Arithmetic mean	1.34	0.932	0.609
STDev	0.334	0.198	0.135
CV or Variability	8.9%	9.4%	25.1%

GC-MS CI	AM-S-W1601	AM-S-W1602	AM-S-W1603
N	3	3	3
Robust mean Algo A	1.58	1.16	0.765
Robust STDev	0.0429	0.00125	0.0132
Median	1.59	1.16	0.767
STDev from MAD	0.0340	0.000994	0.0105
Arithmetic mean	1.47	1.10	0.705
STDev	0.227	0.120	0.114
CV or Variability	2.7%	0.1%	1.7%

GC-MS-MS	AM-S-W1601	AM-S-W1602	AM-S-W1603
N	5	5	6
Robust mean Algo A	1.86	1.29	0.855
Robust STDev	0.289	0.274	0.156
Median	1.86	1.30	0.864
STDev from MAD	0.371	0.295	0.145
Arithmetic mean	1.86	1.29	0.855
STDev	0.255	0.241	0.138
CV or Variability	15.6%	21.3%	18.2%

When fewer than 20 results were considered for statistical treatment of all or a sub-sample of results, the accuracy of statistical data may be questionable.

Distribution
Serum Hexachlorobenzene (µg/L)

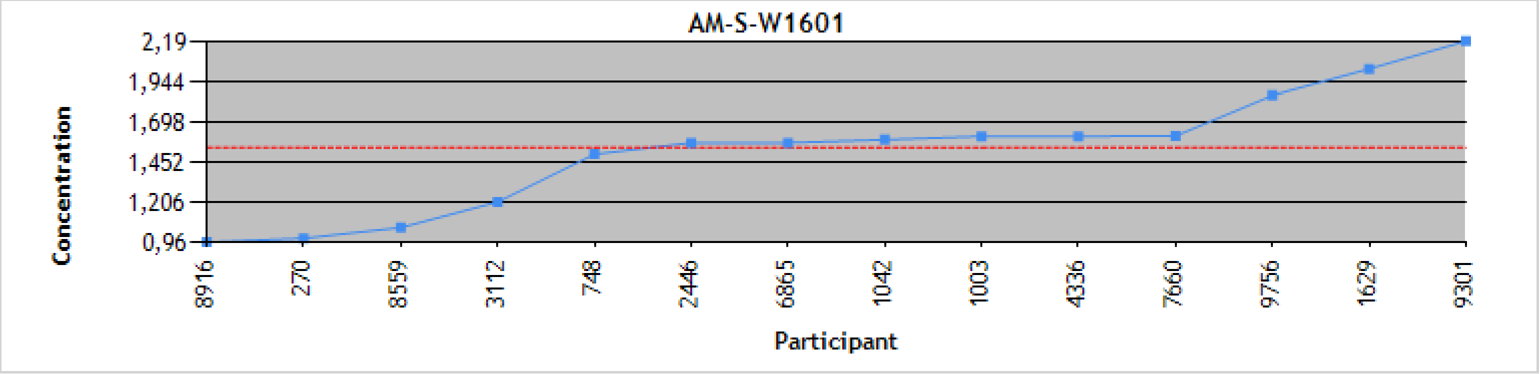

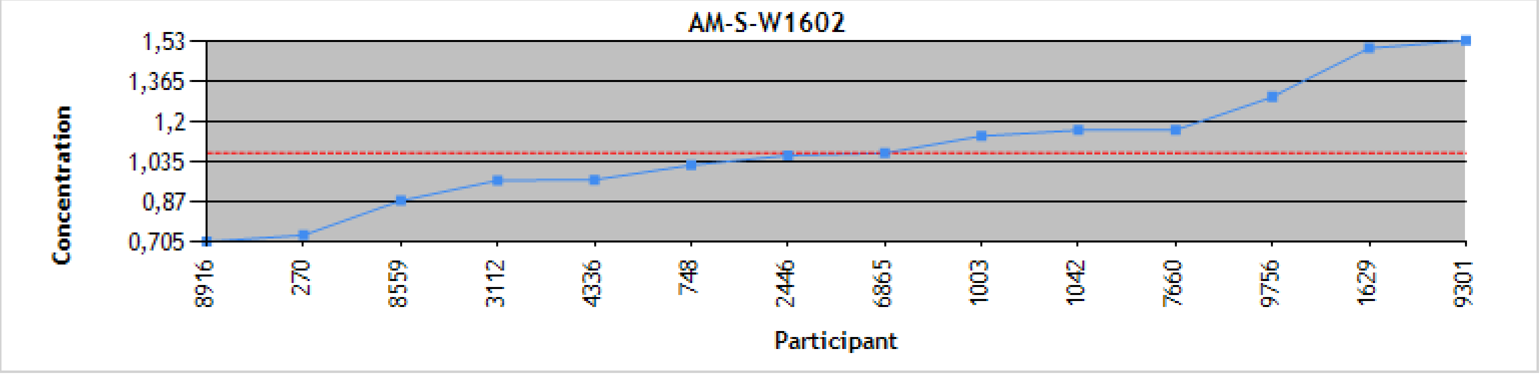

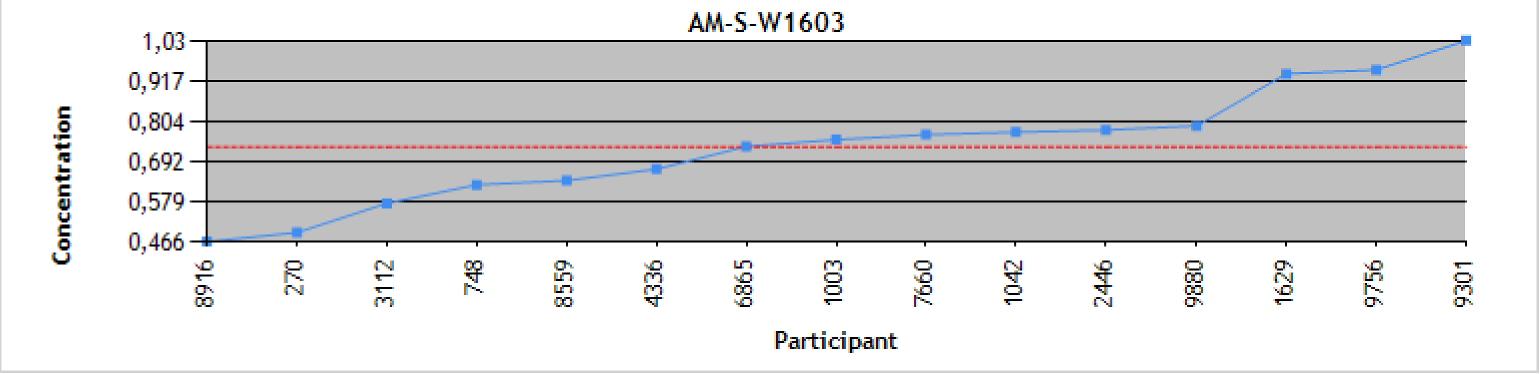

Individual results

Serum Oxychlordane (µg/L)

Round #2016-01

Participant	AM-S-W1601	z'-score	AM-S-W1602	z'-score	AM-S-W1603	z'-score	Method
1003	1.78	0.47	0.311	0.25	1.64	0.68	GC-MS-MS
1042	1.83	0.70	0.319	0.42	1.68	0.89	GC-MS CI
2446	1.66	-0.09	0.280	-0.39	1.46	-0.26	ND
3112	1.41	-1.27	0.238	-1.26	1.33	-0.93	GC-MS CI
4635	1.95	1.28	0.344	0.93	1.73	1.17	GC-MS EI
6865	1.59	-0.43	0.297	-0.04	1.44	-0.36	ND
7660	1.71	0.14	0.303	0.09	1.53	0.13	GC-MS CI
8559	1.59	-0.43	0.280	-0.39	1.48	-0.16	ND
8916	1.36	-1.50	0.250	-1.01	1.36	-0.78	GC
9301	1.87	0.90	0.346	0.97	1.75	1.25	GC-MS-MS
9880	---	---	---	---	0.919	**-3.08**	GC-MS-MS

	Assigned value	Standard uncertainty	σ pt	Acceptable range	K-S (Lilliefors)	Species
AM-S-W1601	1.68	0.0812	0.195	1.26 - 2.10	Accepted	---
AM-S-W1602	0.299	0.0144	0.0461	0.202 - 0.396	Accepted	---
AM-S-W1603	1.51	0.0746	0.177	1.13 - 1.89	Accepted	---

Statistics
Serum Oxychlordane (µg/L)

All methods	AM-S-W1601	AM-S-W1602	AM-S-W1603
N	10	10	11
Robust mean Algo A	1.68	0.299	1.51
Robust STDev	0.205	0.0364	0.198
Median	1.68	0.300	1.48
STDev from MAD	0.177	0.0300	0.219
Arithmetic mean	1.68	0.297	1.48
STDev	0.192	0.0359	0.236
Comparison AM-S-W1505	1.67		
	0.258		
CV or Variability	12.2%	12.2%	13.1%

GC-MS CI	AM-S-W1601	AM-S-W1602	AM-S-W1603
N	3	3	3
Robust mean Algo A	1.66	0.297	1.52
Robust STDev	0.223	0.0295	0.199
Median	1.71	0.303	1.53
STDev from MAD	0.176	0.0233	0.218
Arithmetic mean	1.65	0.287	1.52
STDev	0.214	0.0430	0.175
CV or Variability	13.4%	9.9%	13.1%

GC-MS-MS	AM-S-W1601	AM-S-W1602	AM-S-W1603
N	NA	NA	3
Robust mean Algo A	NA	NA	1.60
Robust STDev	NA	NA	0.206
Median	NA	NA	1.64
STDev from MAD	NA	NA	0.163
Arithmetic mean	NA	NA	1.44
STDev	NA	NA	0.451
CV or Variability	NA	NA	12.9%

When fewer than 20 results were considered for statistical treatment of all or a sub-sample of results, the accuracy of statistical data may be questionable.

Distribution

Serum Oxychlordane (µg/L)

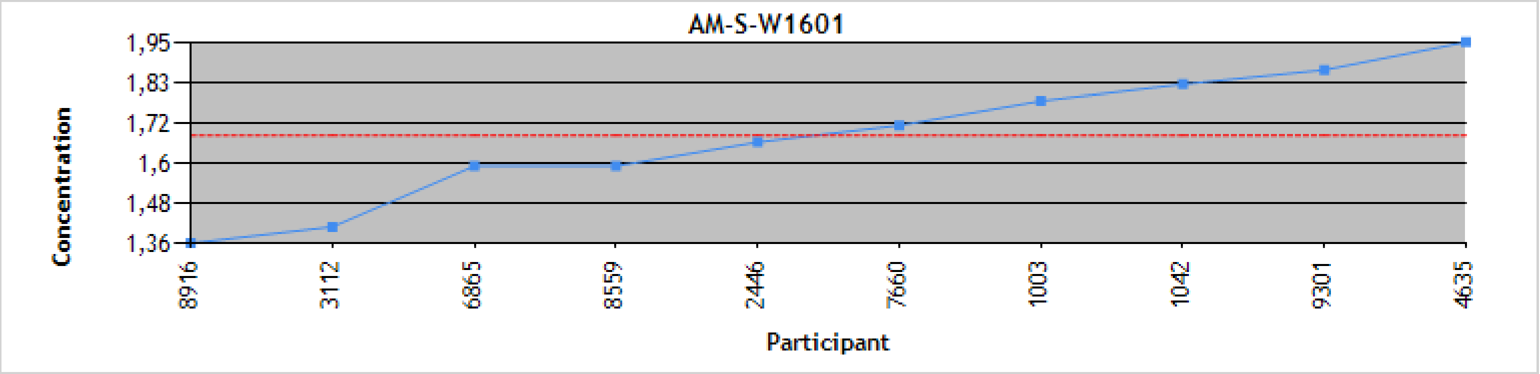

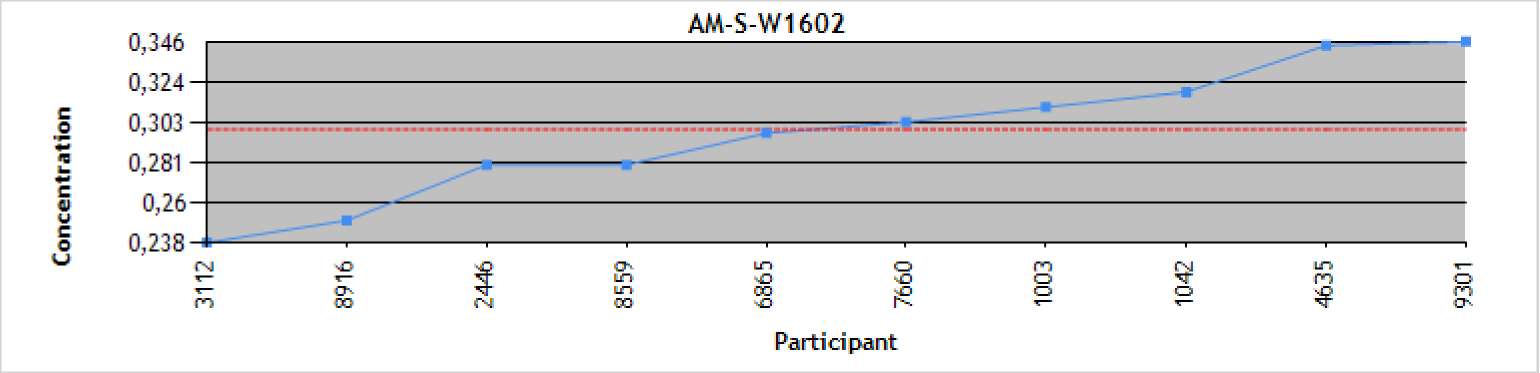

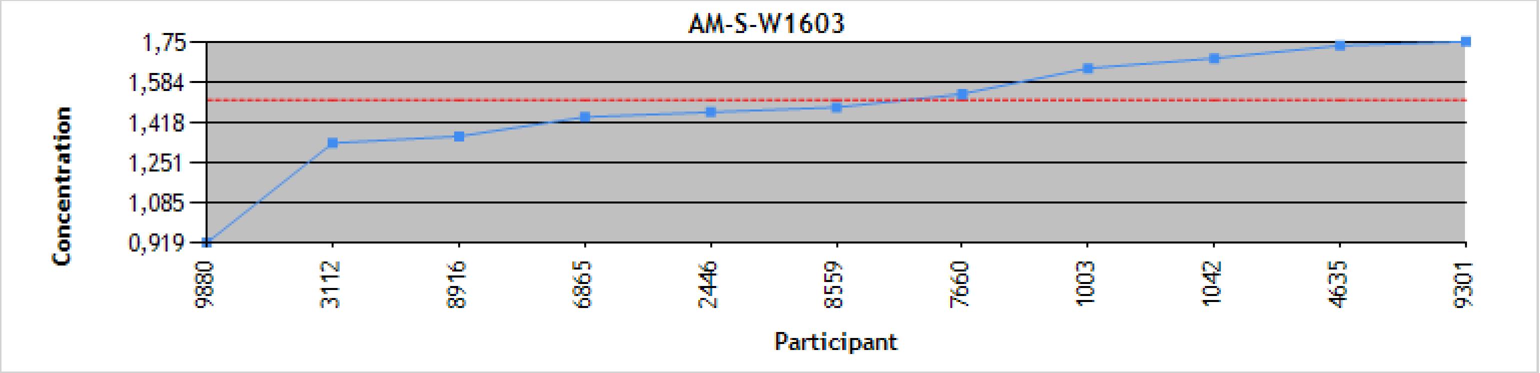

Individual results

Serum p,p'-DDE (µg/L)

Round #2016-01

Participant	AM-S-W1601	z'-score	AM-S-W1602	z'-score	AM-S-W1603	z'-score	Method
270	6.17	-0.14	4.96	-0.49	1.49	-0.11	ND
748	5.54	-1.09	5.29	0.13	1.50	-0.06	GC
1003	6.43	0.26	5.29	0.13	1.53	0.11	GC-MS-MS
1042	6.51	0.38	5.26	0.08	1.51	0.00	GC-MS CI
1629	5.49	-1.18	4.66	-1.03	1.69	0.99	GC-MS-MS
2446	6.46	0.30	5.08	-0.26	1.49	-0.11	ND
3112	5.46	-1.22	4.19	-1.90	1.14	**-2.10**	GC-MS CI
4336	5.91	-0.53	5.65	0.79	1.47	-0.23	GC-MS-MS
4635	7.18	1.39	5.69	0.87	1.60	0.50	GC-MS EI
6865	6.28	0.03	4.99	-0.43	1.32	-1.07	ND
7660	6.24	-0.03	5.11	-0.20	1.49	-0.12	GC-MS CI
8559	5.28	-1.49	4.70	-0.96	1.52	0.06	ND
8916	6.72	0.70	5.61	0.72	1.37	-0.82	GC
9301	7.04	1.18	5.69	0.87	1.64	0.73	GC-MS-MS
9756	7.25	1.50	5.76	1.00	1.83	1.80	GC-MS-MS
9880	---	---	---	---	1.51	0.00	GC-MS-MS

	Assigned value	Standard uncertainty	σ pt	Acceptable range	K-S (Lilliefors)	Species
AM-S-W1601	6.26	0.231	0.617	4.94 - 7.58	Accepted	---
AM-S-W1602	5.22	0.148	0.520	4.14 - 6.30	Accepted	---
AM-S-W1603	1.51	0.0169	0.176	1.16 - 1.86	Accepted	---

Statistics
Serum p,p'-DDE (µg/L)

All methods	AM-S-W1601	AM-S-W1602	AM-S-W1603
N	15	15	16
Robust mean Algo A	6.26	5.22	1.51
Robust STDev	0.715	0.459	0.0540
Median	6.28	5.26	1.50
STDev from MAD	0.650	0.517	0.0445
Arithmetic mean	6.26	5.20	1.51
STDev	0.634	0.452	0.154
Comparison AM-S-W1505	6.65		
	0.693		
CV or Variability	11.4%	8.8%	3.6%

GC-MS CI	AM-S-W1601	AM-S-W1602	AM-S-W1603
N	3	3	3
Robust mean Algo A	6.13	5.05	1.48
Robust STDev	0.507	0.284	0.0398
Median	6.24	5.11	1.49
STDev from MAD	0.402	0.225	0.0316
Arithmetic mean	6.07	4.86	1.38
STDev	0.546	0.579	0.209
CV or Variability	8.3%	5.6%	2.7%

GC-MS-MS	AM-S-W1601	AM-S-W1602	AM-S-W1603
N	5	5	6
Robust mean Algo A	6.42	5.58	1.60
Robust STDev	0.842	0.188	0.135
Median	6.43	5.65	1.59
STDev from MAD	0.905	0.163	0.130
Arithmetic mean	6.42	5.41	1.61
STDev	0.743	0.456	0.135
CV or Variability	13.1%	3.4%	8.4%

When fewer than 20 results were considered for statistical treatment of all or a sub-sample of results, the accuracy of statistical data may be questionable.

Distribution

Serum p,p'-DDE (µg/L)

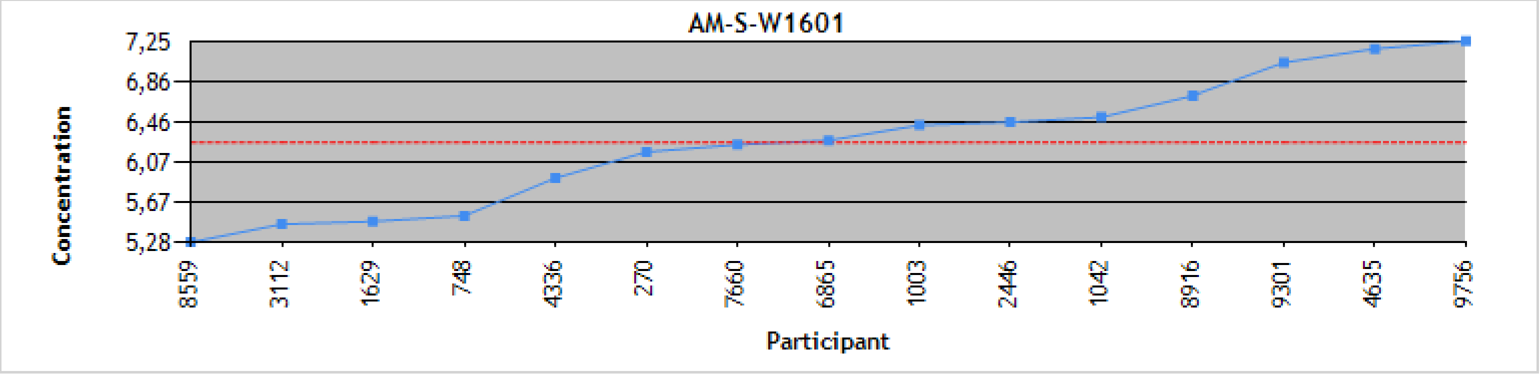

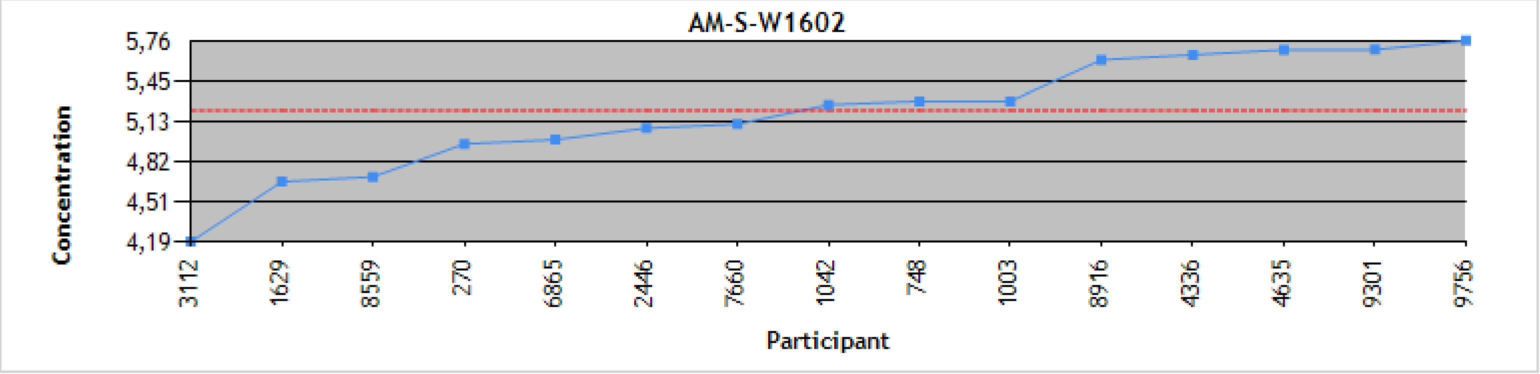

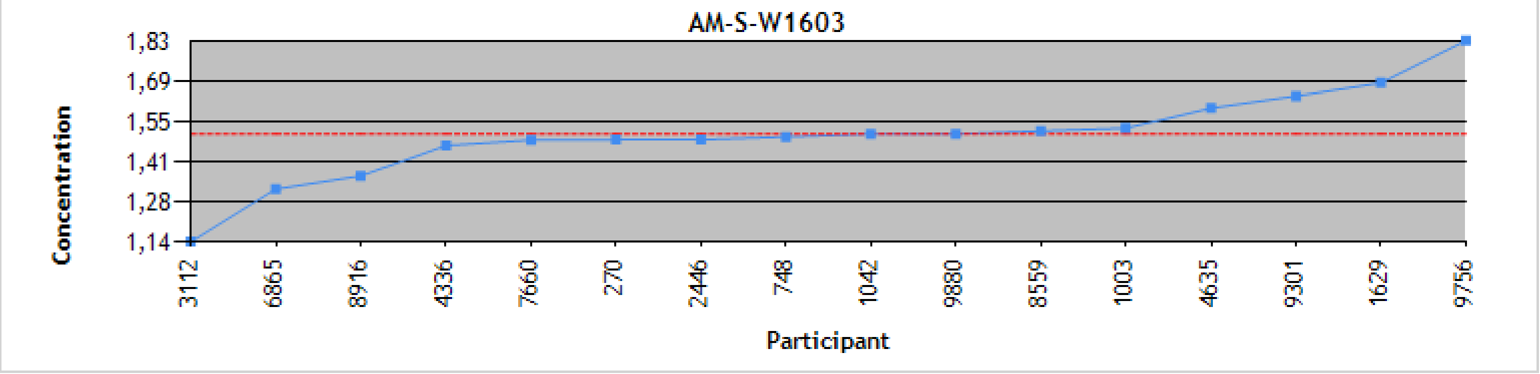

Individual results

Serum p,p'-DDT (µg/L)

Round #2016-01

Participant	AM-S-W1601	z'-score	AM-S-W1602	z'-score	AM-S-W1603	z'-score	Method
270	1.79	0.96	0.908	1.12	1.52	0.80	ND
748	1.51	0.00	0.794	0.34	1.42	0.40	GC
1003	1.02	-1.68	0.754	0.07	1.16	-0.63	GC-MS-MS
1042	1.67	0.53	0.766	0.15	1.40	0.33	GC-MS CI
1629	1.10	-1.40	0.635	-0.75	0.932	-1.54	GC-MS-MS
2446	1.38	-0.44	0.630	-0.78	1.30	-0.08	ND
3112	1.51	0.00	0.695	-0.33	1.30	-0.09	GC-MS CI
4336	1.26	-0.86	0.680	-0.44	1.06	-1.03	GC-MS-MS
4635	1.81	1.02	0.829	0.58	1.52	0.80	GC-MS EI
6865	1.29	-0.75	0.596	-1.01	1.14	-0.71	ND
7660	1.65	0.48	0.740	-0.02	1.44	0.47	GC-MS CI
8559	1.49	-0.07	0.724	-0.14	1.16	-0.63	ND
8916	1.39	-0.41	0.687	-0.39	1.19	-0.52	GC
9301	2.01	1.71	0.870	0.86	1.68	1.42	GC-MS-MS
9756	1.81	1.03	0.967	1.52	1.67	1.39	GC-MS-MS

	Assigned value	Standard uncertainty	σ pt	Acceptable range	K-S (Lilliefors)	Species
AM-S-W1601	1.51	0.100	0.275	0.925 - 2.10	Accepted	---
AM-S-W1602	0.744	0.0331	0.143	0.451 - 1.04	Accepted	---
AM-S-W1603	1.32	0.0733	0.242	0.814 - 1.83	Accepted	---

Statistics
Serum p,p'-DDT (µg/L)

All methods	AM-S-W1601	AM-S-W1602	AM-S-W1603
N	15	15	15
Robust mean Algo A	1.51	0.744	1.32
Robust STDev	0.310	0.103	0.227
Median	1.51	0.740	1.30
STDev from MAD	0.326	0.0895	0.208
Arithmetic mean	1.51	0.752	1.33
STDev	0.281	0.107	0.221
Comparison AM-S-W1505	1.61		
	0.211		
CV or Variability	20.5%	13.8%	17.2%

GC-MS CI	AM-S-W1601	AM-S-W1602	AM-S-W1603
N	3	3	3
Robust mean Algo A	1.65	0.734	1.39
Robust STDev	0.0280	0.0406	0.0676
Median	1.65	0.740	1.40
STDev from MAD	0.0222	0.0376	0.0535
Arithmetic mean	1.61	0.734	1.38
STDev	0.0862	0.0358	0.0737
CV or Variability	1.7%	5.5%	4.9%

GC-MS-MS	AM-S-W1601	AM-S-W1602	AM-S-W1603
N	5	5	5
Robust mean Algo A	1.39	0.781	1.30
Robust STDev	0.426	0.155	0.395
Median	1.26	0.754	1.16
STDev from MAD	0.356	0.172	0.339
Arithmetic mean	1.44	0.781	1.30
STDev	0.443	0.137	0.352
CV or Variability	30.6%	19.9%	30.4%

When fewer than 20 results were considered for statistical treatment of all or a sub-sample of results, the accuracy of statistical data may be questionable.

Distribution

Serum p,p'-DDT (µg/L)

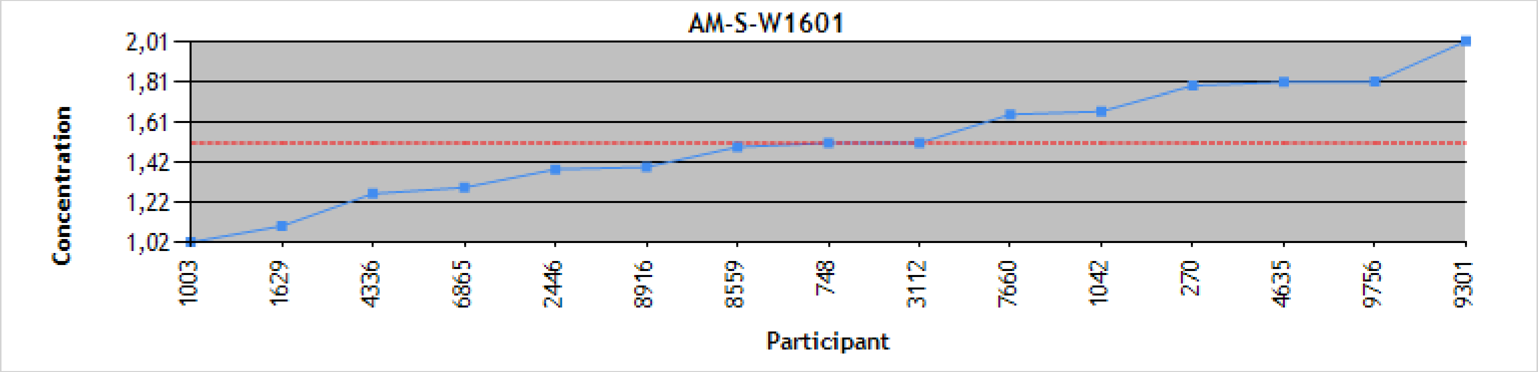

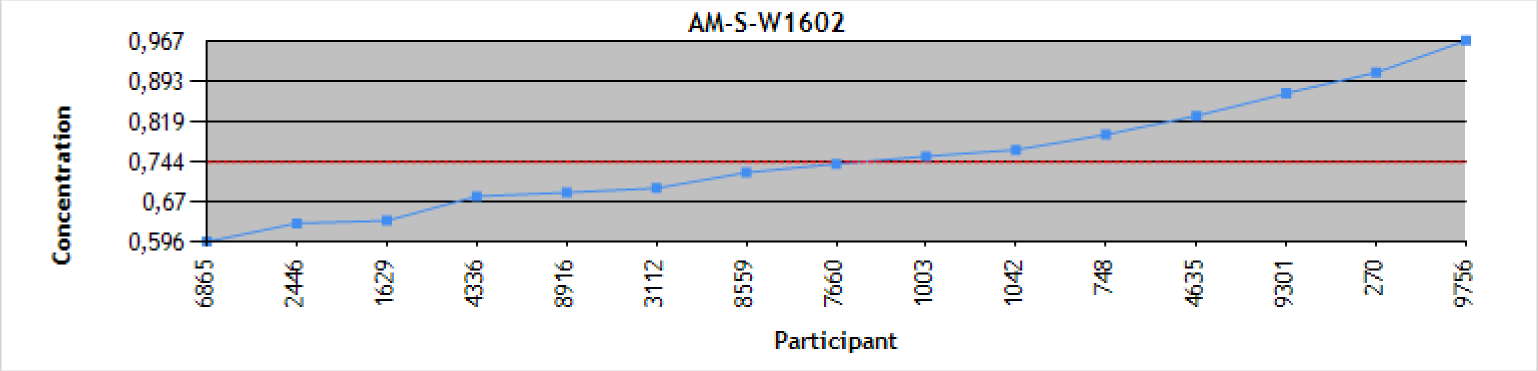

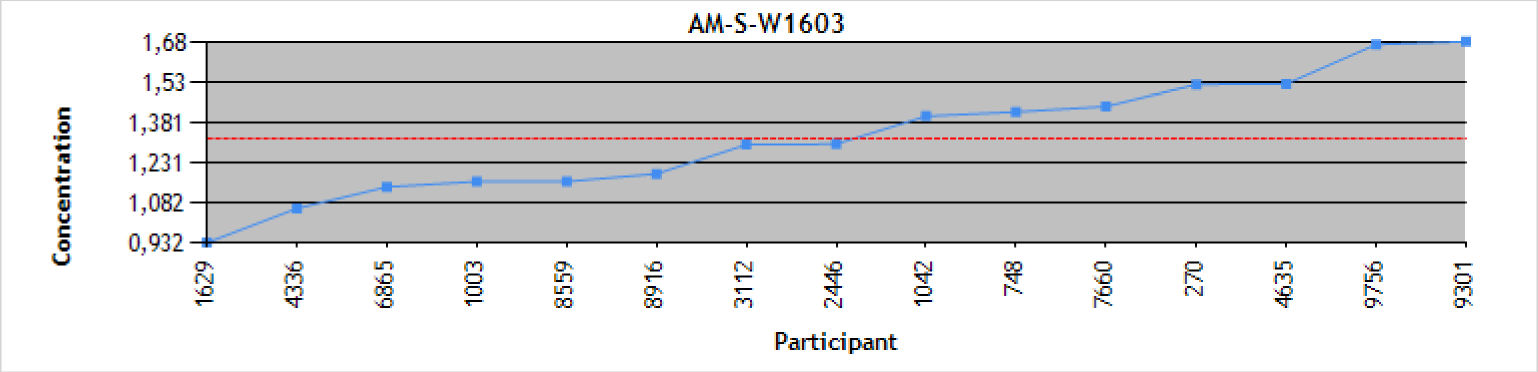

Individual results

Serum PBDE IUPAC # 28 (µg/L)

Round #2016-01

Participant	AM-S-W1601	z'-score	AM-S-W1602	z'-score	AM-S-W1603	z'-score	Method
**748**	0.0935	-0.27	0.428	-0.42	0.0617	-1.32	GC-MS CI
**1042**	0.0949	-0.16	0.448	-0.05	0.0770	0.07	GC-MS CI
**1629**	0.105	0.61	0.462	0.20	0.0840	0.71	GC-MS EI
**2446**	0.0990	0.15	0.472	0.38	0.0760	-0.02	ND
**3112**	0.0810	-1.22	0.397	-0.98	0.0620	-1.29	GC-MS CI
**4635**	0.0950	-0.15	0.440	-0.20	0.0750	-0.11	GC-MS EI
**5039**	0.115	1.33	0.504	0.97	0.104	**2.50**	GC-MS-MS
**7660**	0.0912	-0.44	0.450	-0.02	0.0713	-0.44	GC-MS CI
**8916**	0.100	0.23	0.446	-0.09	0.0810	0.44	GC-MS EI
**9301**	0.0985	0.11	0.463	0.22	0.0785	0.21	GC-MS EI

	Assigned value	Standard uncertainty	σ pt	Acceptable range	K-S (Lilliefors)	Species
**AM-S-W1601**	0.0970	0.00223	0.0130	0.0707 - 0.123	Accepted	---
**AM-S-W1602**	0.451	0.00839	0.0543	0.341 - 0.561	Accepted	---
**AM-S-W1603**	0.0762	0.00319	0.0105	0.0542 - 0.0982	Accepted	---

Statistics
Serum PBDE IUPAC # 28 (µg/L)

All methods	AM-S-W1601	AM-S-W1602	AM-S-W1603
N	10	10	10
Robust mean Algo A	0.0970	0.451	0.0762
Robust STDev	0.00565	0.0212	0.00806
Median	0.0968	0.449	0.0765
STDev from MAD	0.00482	0.0198	0.00716
Arithmetic mean	0.0973	0.451	0.0770
STDev	0.00879	0.0282	0.0119
Comparison AM-S-W1505	0.0950		
	0.00695		
CV or Variability	5.8%	4.7%	10.6%

GC-MS CI	AM-S-W1601	AM-S-W1602	AM-S-W1603
N	4	4	4
Robust mean Algo A	0.0919	0.435	0.0680
Robust STDev	0.00333	0.0197	0.00848
Median	0.0923	0.438	0.0667
STDev from MAD	0.00277	0.0163	0.00715
Arithmetic mean	0.0901	0.431	0.0680
STDev	0.00628	0.0247	0.00748
CV or Variability	3.6%	4.5%	12.5%

GC-MS EI	AM-S-W1601	AM-S-W1602	AM-S-W1603
N	4	4	4
Robust mean Algo A	0.0996	0.453	0.0796
Robust STDev	0.00461	0.0131	0.00433
Median	0.0993	0.454	0.0798
STDev from MAD	0.00371	0.0126	0.00408
Arithmetic mean	0.0996	0.453	0.0796
STDev	0.00415	0.0115	0.00382
CV or Variability	4.6%	2.9%	5.4%

When fewer than 20 results were considered for statistical treatment of all or a sub-sample of results, the accuracy of statistical data may be questionable.

Distribution

Serum PBDE IUPAC # 28 (µg/L)

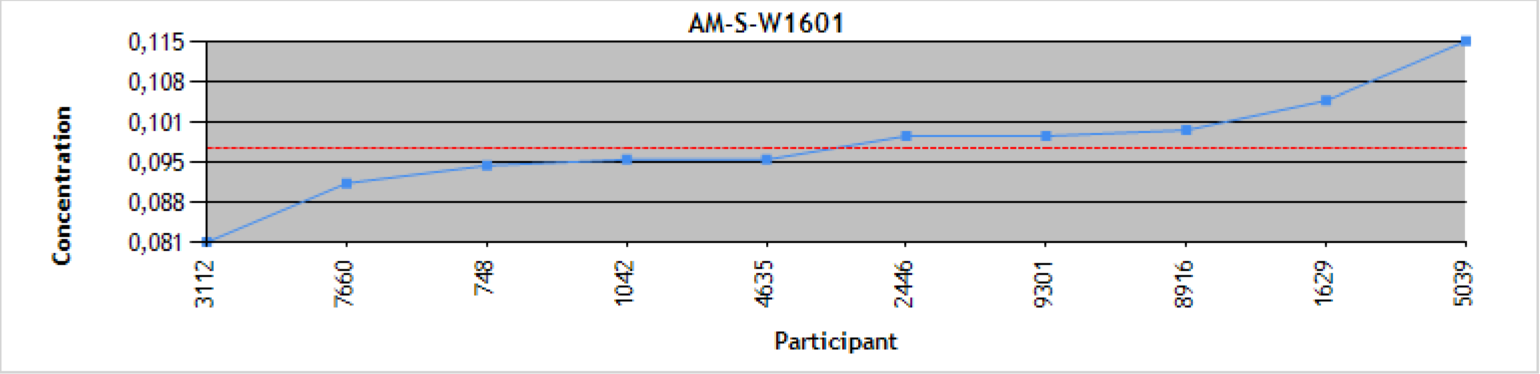

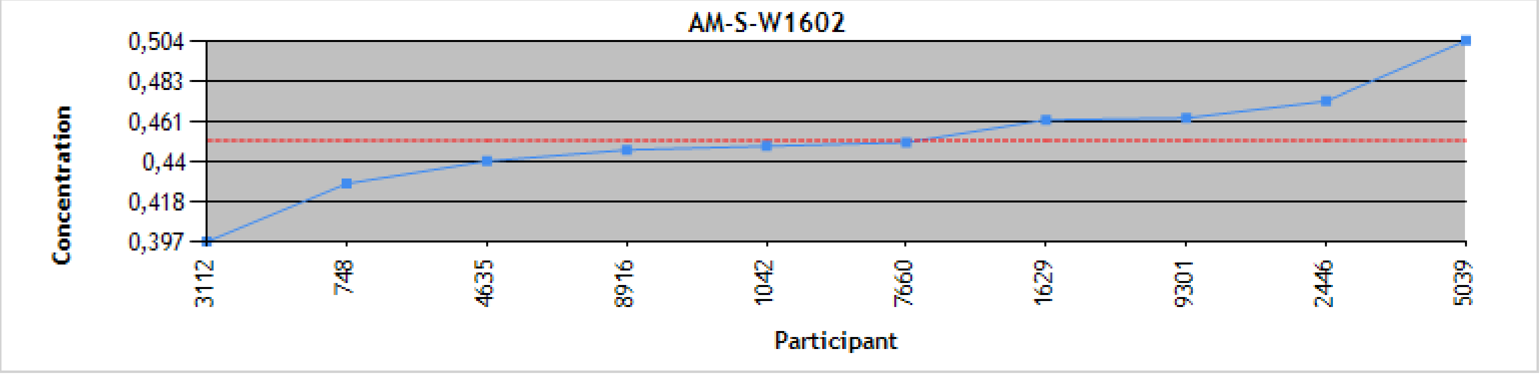

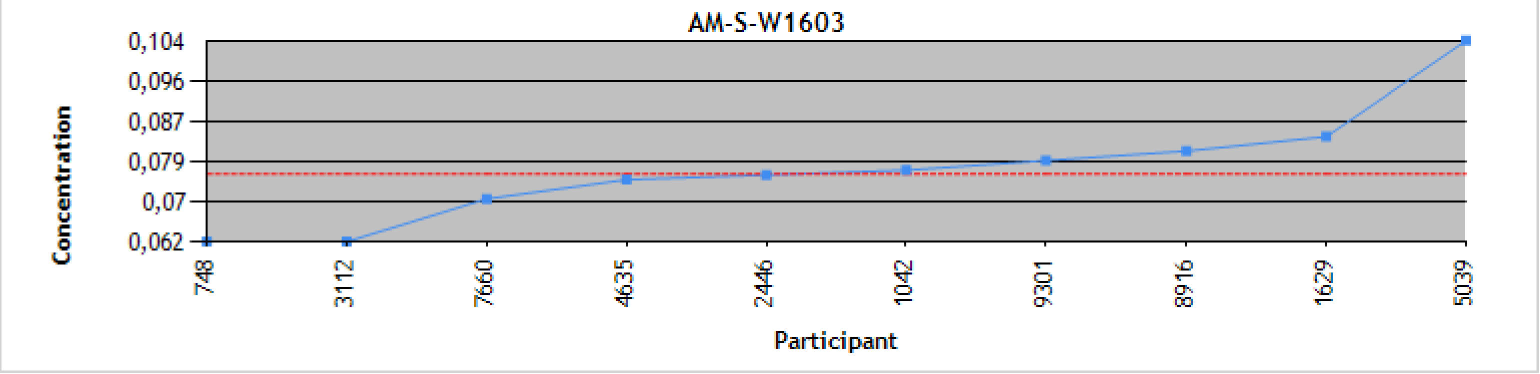

Individual results

Serum PBDE IUPAC # 47 (µg/L)

Round #2016-01

Participant	AM-S-W1601	z'-score	AM-S-W1602	z'-score	AM-S-W1603	z'-score	Method
**748**	0.234	-1.04	1.11	**-2.02**	0.798	-0.79	GC-MS CI
**1003**	0.255	-0.47	1.46	0.00	0.855	-0.25	GC-MS-MS
**1042**	0.265	-0.19	1.45	-0.07	0.863	-0.17	GC-MS CI
**1629**	0.283	0.30	1.54	0.46	0.916	0.33	GC-MS EI
**2446**	0.281	0.25	1.57	0.61	0.959	0.74	ND
**3112**	0.247	-0.69	1.36	-0.56	0.940	0.56	GC-MS CI
**4635**	0.268	-0.11	1.38	-0.47	0.828	-0.50	GC-MS EI
**5039**	1.37	**30.00**	1.88	**2.41**	1.54	**6.20**	GC-MS-MS
**6865**	0.314	1.15	1.13	-1.91	0.758	-1.16	ND
**7660**	0.264	-0.23	1.50	0.23	0.835	-0.44	GC-MS CI
**8916**	0.273	0.03	1.60	0.81	0.912	0.29	GC-MS EI
**9301**	0.271	-0.03	1.34	-0.69	0.775	-1.00	GC-MS EI
**9756**	0.285	0.36	1.80	1.96	1.02	1.31	GC-MS-MS

	Assigned value	Standard uncertainty	σ pt	Acceptable range	K-S (Lilliefors)	Species
**AM-S-W1601**	0.272	0.00672	0.0358	0.199 - 0.345	Rejected[1]	---
**AM-S-W1602**	1.46	0.0608	0.162	1.11 - 1.81	Accepted	---
**AM-S-W1603**	0.881	0.0328	0.101	0.670 - 1.09	Rejected[1]	---

Statistics
Serum PBDE IUPAC # 47 (µg/L)

All methods	AM-S-W1601	AM-S-W1602	AM-S-W1603
N	13	13	13
Robust mean Algo A	0.272	1.46	0.881
Robust STDev	0.0194	0.175	0.0945
Median	0.271	1.46	0.863
STDev from MAD	0.0178	0.156	0.0967
Arithmetic mean	0.354	1.47	0.923
STDev	0.305	0.222	0.199
Comparison AM-S-W1505	0.263		
	0.0221		
CV or Variability	7.1%	12.0%	10.7%

GC-MS CI	AM-S-W1601	AM-S-W1602	AM-S-W1603
N	4	4	4
Robust mean Algo A	0.253	1.39	0.854
Robust STDev	0.0162	0.123	0.0591
Median	0.255	1.41	0.849
STDev from MAD	0.0134	0.102	0.0484
Arithmetic mean	0.252	1.36	0.859
STDev	0.0148	0.173	0.0602
CV or Variability	6.4%	8.8%	6.9%

GC-MS EI	AM-S-W1601	AM-S-W1602	AM-S-W1603
N	4	4	4
Robust mean Algo A	0.272	1.46	0.858
Robust STDev	0.00455	0.142	0.0777
Median	0.272	1.46	0.870
STDev from MAD	0.00371	0.148	0.0653
Arithmetic mean	0.274	1.46	0.858
STDev	0.00650	0.125	0.0685
CV or Variability	1.7%	9.7%	9.1%

GC-MS-MS	AM-S-W1601	AM-S-W1602	AM-S-W1603
N	3	3	3
Robust mean Algo A	0.297	1.77	1.09
Robust STDev	0.0562	0.144	0.309
Median	0.285	1.80	1.02
STDev from MAD	0.0445	0.114	0.245
Arithmetic mean	0.635	1.71	1.14
STDev	0.633	0.222	0.355
CV or Variability	18.9%	8.2%	28.4%

When fewer than 20 results were considered for statistical treatment of all or a sub-sample of results, the accuracy of statistical data may be questionable.

Distribution
Serum PBDE IUPAC # 47 (µg/L)

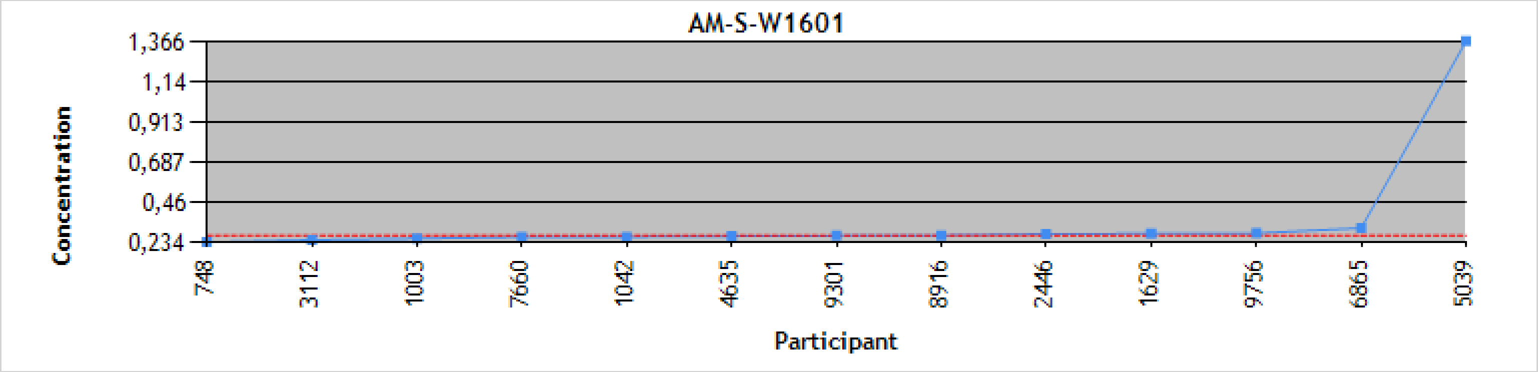

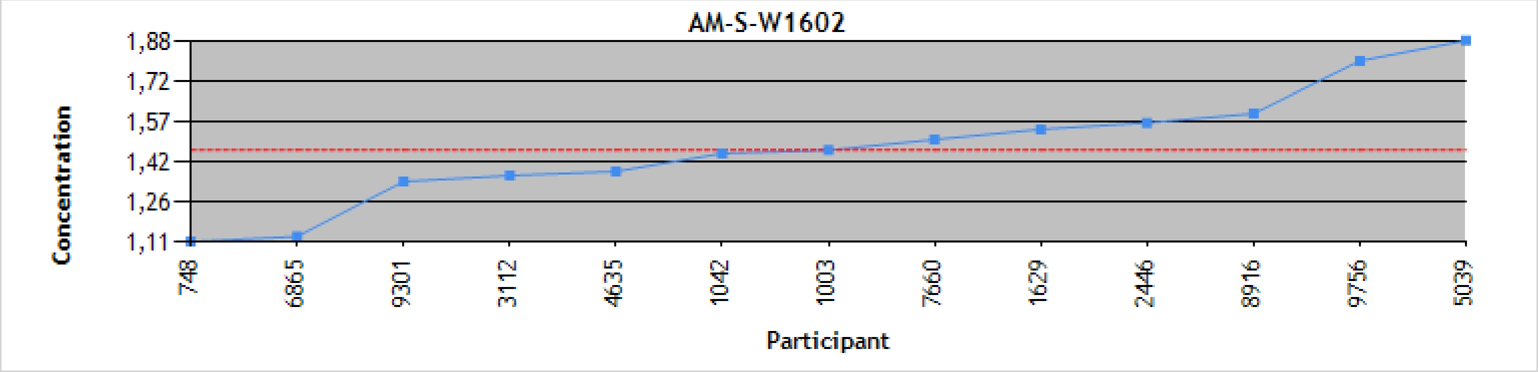

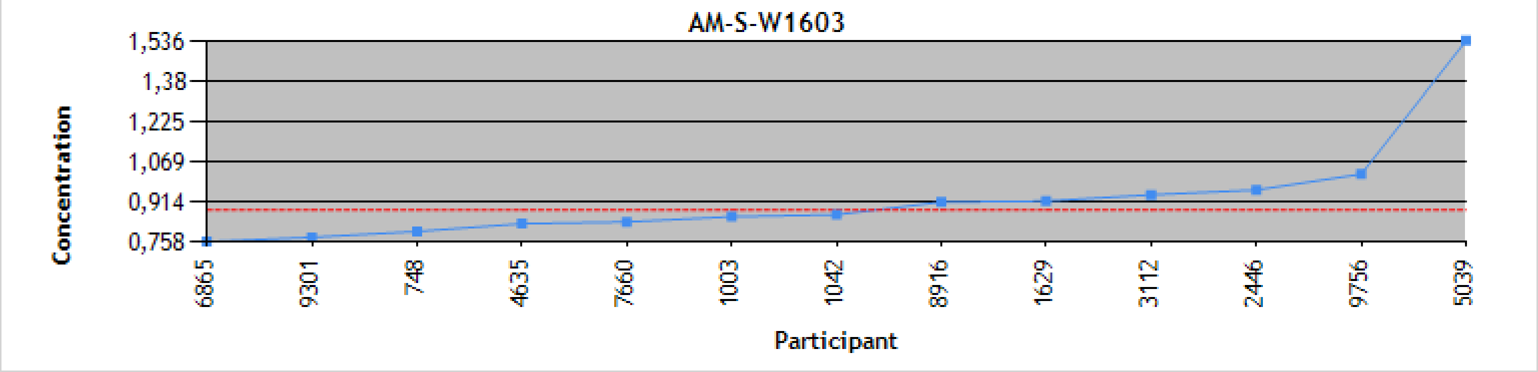

Individual results

Serum PBDE IUPAC # 99 (µg/L)

Round #2016-01

Participant	AM-S-W1601	z'-score	AM-S-W1602	z'-score	AM-S-W1603	z'-score	Method
**748**	0.327	-0.77	0.149	-0.40	0.669	0.16	GC-MS CI
**1003**	0.355	-0.14	0.157	-0.08	0.704	0.65	GC-MS-MS
**1042**	0.399	0.86	0.164	0.22	0.621	-0.53	GC-MS CI
**1629**	0.400	0.88	0.175	0.64	0.657	-0.01	GC-MS EI
**2446**	0.353	-0.18	0.160	0.04	0.648	-0.14	ND
**3112**	0.329	-0.72	0.140	-0.76	0.668	0.14	GC-MS CI
**4635**	0.351	-0.23	0.157	-0.08	0.653	-0.07	GC-MS EI
**5039**	0.618	**5.80**	0.285	**5.02**	0.861	**2.87**	GC-MS-MS
**6865**	0.355	-0.14	0.188	1.16	0.595	-0.89	ND
**7660**	0.351	-0.22	0.156	-0.13	0.635	-0.33	GC-MS CI
**8916**	0.378	0.38	0.153	-0.24	0.729	1.00	GC-MS EI
**9301**	0.338	-0.52	0.157	-0.08	0.628	-0.42	GC-MS EI

	Assigned value	Standard uncertainty	σ pt	Acceptable range	K-S (Lilliefors)	Species
**AM-S-W1601**	0.361	0.0107	0.0429	0.273 - 0.449	Rejected[1]	---
**AM-S-W1602**	0.159	0.00332	0.0248	0.109 - 0.209	Rejected[1]	---
**AM-S-W1603**	0.658	0.0142	0.0695	0.516 - 0.800	Rejected[1]	---

Statistics

Serum PBDE IUPAC # 99 (µg/L)

All methods	AM-S-W1601	AM-S-W1602	AM-S-W1603
N	12	12	12
Robust mean Algo A	0.361	0.159	0.658
Robust STDev	0.0297	0.00920	0.0394
Median	0.354	0.157	0.655
STDev from MAD	0.0297	0.00846	0.0350
Arithmetic mean	0.379	0.170	0.672
STDev	0.0786	0.0381	0.0695
Comparison AM-S-W1505	0.342		
	0.0258		
CV or Variability	8.2%	5.8%	6.0%

GC-MS CI	AM-S-W1601	AM-S-W1602	AM-S-W1603
N	4	4	4
Robust mean Algo A	0.344	0.152	0.648
Robust STDev	0.0216	0.0117	0.0274
Median	0.340	0.152	0.651
STDev from MAD	0.0179	0.0114	0.0253
Arithmetic mean	0.352	0.152	0.648
STDev	0.0335	0.0103	0.0242
CV or Variability	6.3%	7.7%	4.2%

GC-MS EI	AM-S-W1601	AM-S-W1602	AM-S-W1603
N	4	4	4
Robust mean Algo A	0.367	0.157	0.656
Robust STDev	0.0314	0.00391	0.0275
Median	0.365	0.157	0.655
STDev from MAD	0.0297	0.00297	0.0215
Arithmetic mean	0.367	0.161	0.667
STDev	0.0277	0.00985	0.0434
CV or Variability	8.6%	2.5%	4.2%

When fewer than 20 results were considered for statistical treatment of all or a sub-sample of results, the accuracy of statistical data may be questionable.

Distribution

Serum PBDE IUPAC # 99 (µg/L)

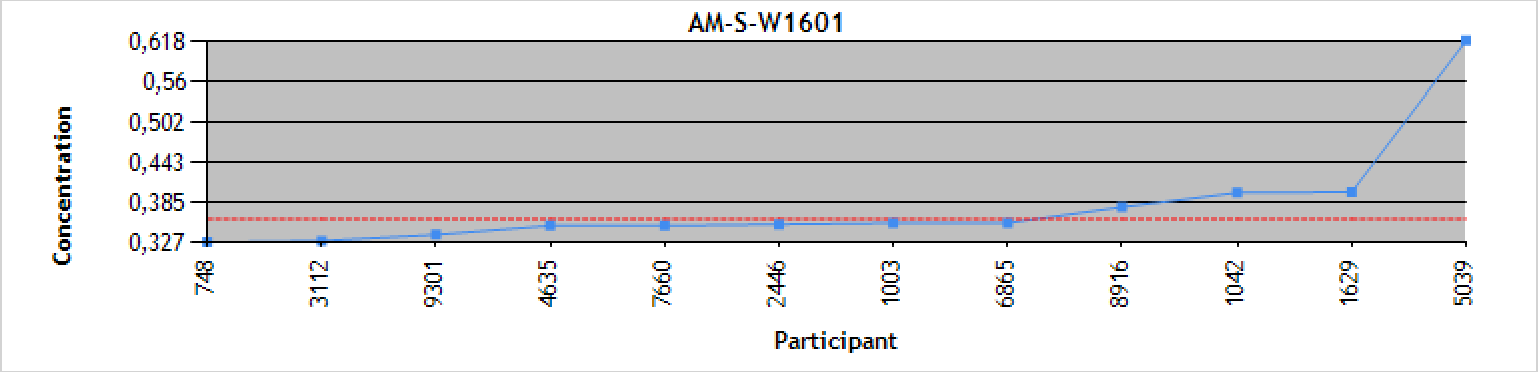

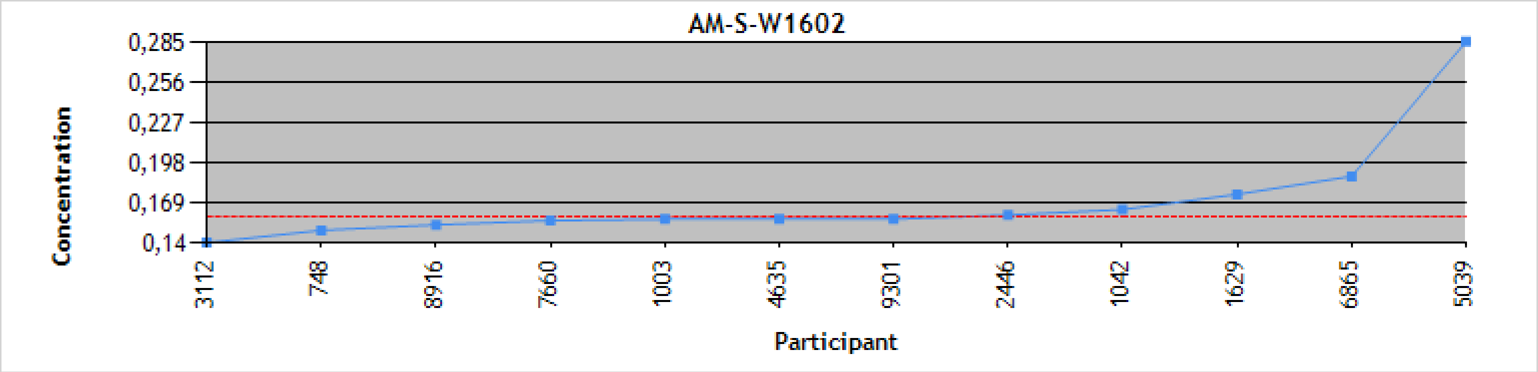

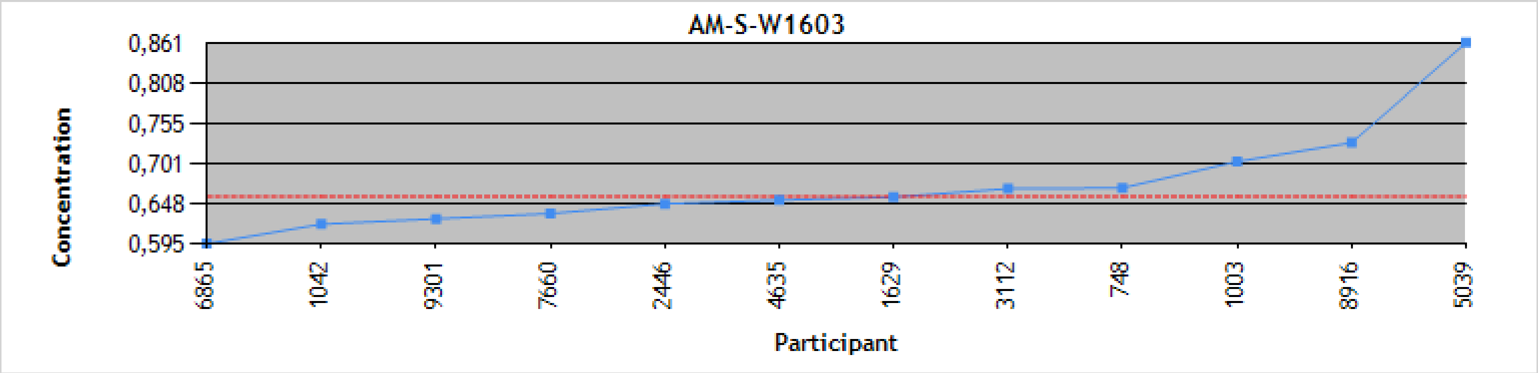

Individual results

Serum PBDE IUPAC # 100 (µg/L)

Round #2016-01

Participant	AM-S-W1601	z'-score	AM-S-W1602	z'-score	AM-S-W1603	z'-score	Method
748	0.0613	**-2.06**	0.179	**-2.48**	0.406	**-2.75**	GC-MS CI
1042	0.0846	-0.53	0.247	-0.48	0.579	-0.50	GC-MS CI
1629	0.0990	0.41	0.275	0.35	0.688	0.93	GC-MS EI
2446	0.0850	-0.50	0.248	-0.44	0.661	0.57	ND
3112	0.0730	-1.29	0.209	-1.60	0.521	-1.25	GC-MS CI
4635	0.0940	0.09	0.264	0.03	0.644	0.35	GC-MS EI
5039	0.191	**6.42**	0.290	0.81	0.604	-0.17	GC-MS-MS
6865	0.103	0.67	0.259	-0.12	0.526	-1.19	ND
7660	0.0924	-0.02	0.269	0.19	0.628	0.14	GC-MS CI
8916	0.0920	-0.05	0.283	0.59	0.683	0.86	GC-MS EI
9301	0.113	1.33	0.313	1.48	0.744	1.66	GC-MS EI

	Assigned value	Standard uncertainty	σ pt	Acceptable range	K-S (Lilliefors)	Species
AM-S-W1601	0.0927	0.00521	0.0143	0.0622 - 0.123	Rejected[1]	---
AM-S-W1602	0.263	0.0106	0.0321	0.195 - 0.331	Accepted	---
AM-S-W1603	0.617	0.0331	0.0691	0.464 - 0.770	Accepted	---

Statistics

Serum PBDE IUPAC # 100 (µg/L)

All methods	AM-S-W1601	AM-S-W1602	AM-S-W1603
N	11	11	11
Robust mean Algo A	0.0927	0.263	0.617
Robust STDev	0.0138	0.0280	0.0877
Median	0.0924	0.264	0.628
STDev from MAD	0.0116	0.0254	0.0816
Arithmetic mean	0.0989	0.258	0.608
STDev	0.0335	0.0374	0.0954
Comparison AM-S-W1505	0.0876		
	0.0123		
CV or Variability	14.9%	10.7%	14.2%

GC-MS CI	AM-S-W1601	AM-S-W1602	AM-S-W1603
N	4	4	4
Robust mean Algo A	0.0778	0.226	0.540
Robust STDev	0.0154	0.0455	0.0960
Median	0.0788	0.228	0.550
STDev from MAD	0.0144	0.0448	0.0793
Arithmetic mean	0.0778	0.226	0.534
STDev	0.0136	0.0401	0.0956
CV or Variability	19.8%	20.1%	17.8%

GC-MS EI	AM-S-W1601	AM-S-W1602	AM-S-W1603
N	4	4	4
Robust mean Algo A	0.0973	0.281	0.687
Robust STDev	0.00623	0.0173	0.0420
Median	0.0965	0.279	0.686
STDev from MAD	0.00519	0.0141	0.0326
Arithmetic mean	0.0995	0.284	0.690
STDev	0.00947	0.0210	0.0412
CV or Variability	6.4%	6.1%	6.1%

When fewer than 20 results were considered for statistical treatment of all or a sub-sample of results, the accuracy of statistical data may be questionable.

Distribution

Serum PBDE IUPAC # 100 (µg/L)

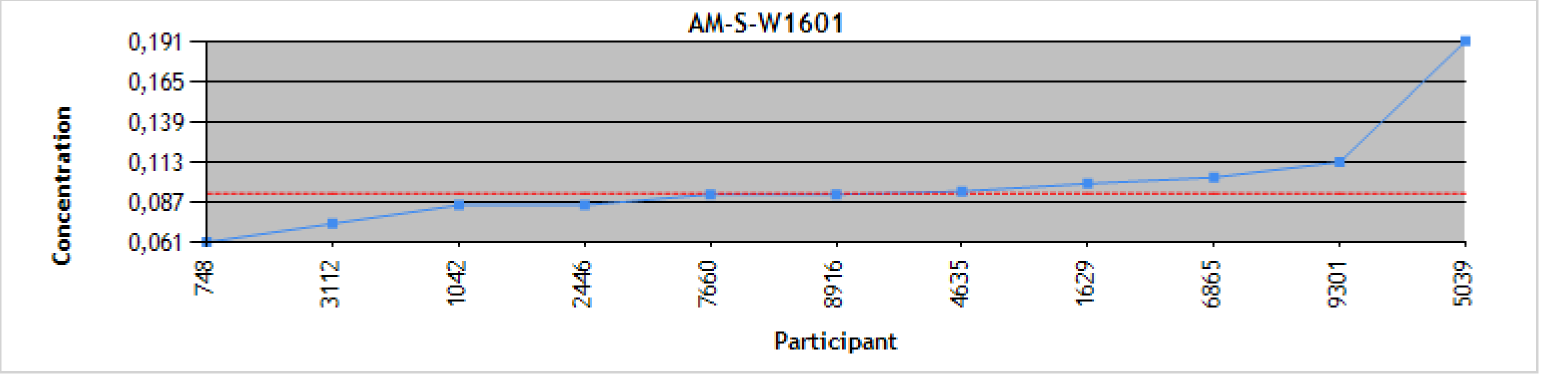

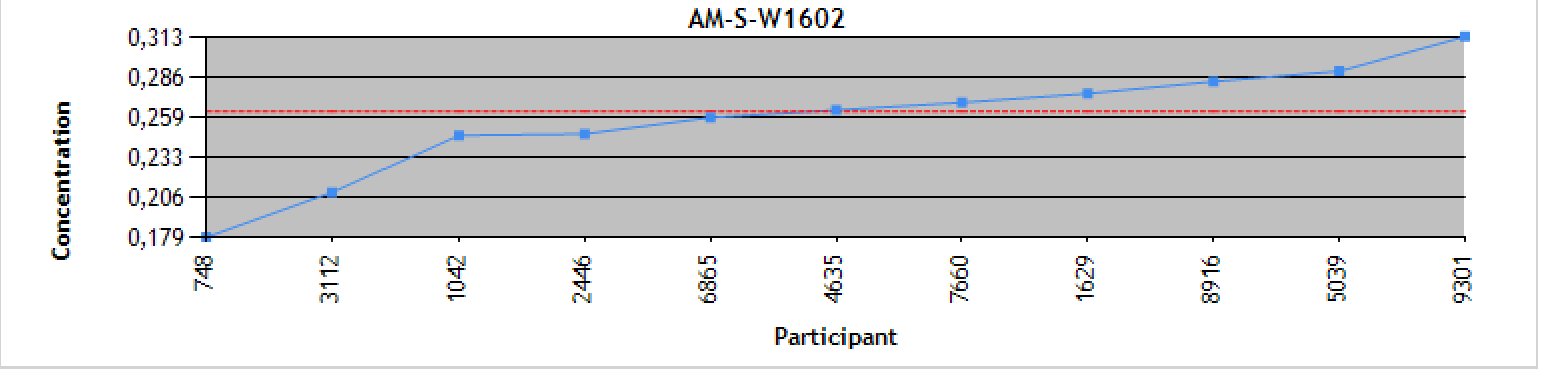

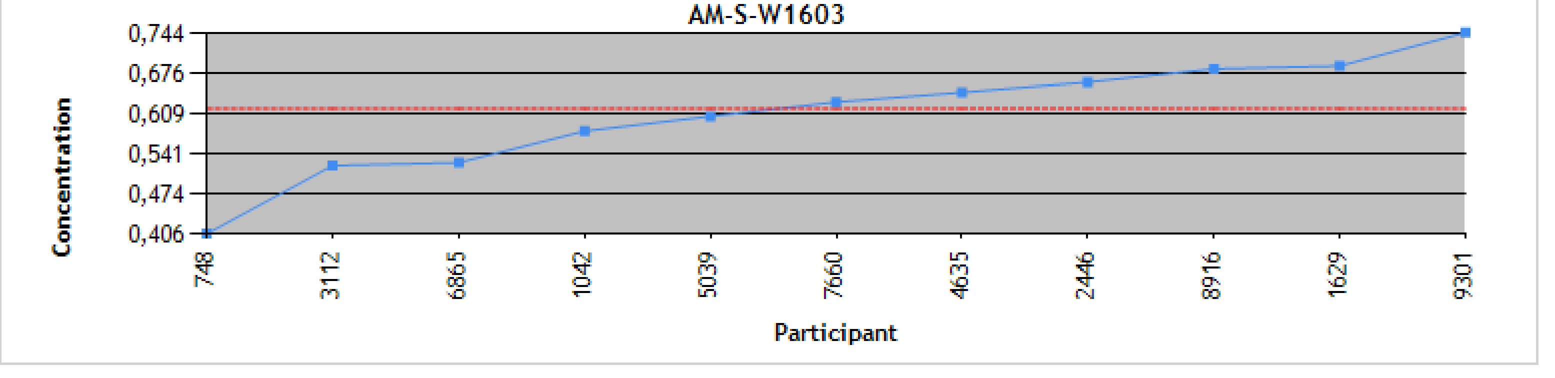

Individual results

Serum PBDE IUPAC # 153 (µg/L)

Round #2016-01

Participant	AM-S-W1601	z'-score	AM-S-W1602	z'-score	AM-S-W1603	z'-score	Method
**748**	0.477	-0.20	0.0660	**-2.02**	0.940	0.00	GC-MS CI
**1003**	0.458	-0.49	0.0900	-0.71	0.897	-0.34	GC-MS-MS
**1042**	0.565	1.15	0.110	0.36	1.02	0.65	GC-MS CI
**1629**	0.530	0.61	0.103	0.00	0.970	0.24	GC-MS EI
**2446**	0.462	-0.43	0.0900	-0.71	0.808	-1.06	ND
**3112**	0.469	-0.32	0.140	**2.02**	0.910	-0.24	GC-MS CI
**4635**	0.492	0.03	0.100	-0.16	0.914	-0.21	GC-MS EI
**5039**	0.457	-0.51	0.0875	-0.85	0.818	-0.98	GC-MS-MS
**6865**	0.449	-0.63	0.140	**2.02**	0.793	-1.18	ND
**7660**	0.518	0.42	0.0978	-0.28	1.01	0.58	GC-MS CI
**8916**	0.518	0.43	0.119	0.87	1.11	1.34	GC-MS EI
**9301**	0.492	0.03	0.108	0.27	0.980	0.32	GC-MS EI
**9756**	0.506	0.24	0.101	-0.11	1.06	0.96	GC-MS-MS

	Assigned value	Standard uncertainty	σ pt	Acceptable range	K-S (Lilliefors)	Species
**AM-S-W1601**	0.490	0.0121	0.0643	0.359 - 0.621	Accepted	---
**AM-S-W1602**	0.103	0.00580	0.0173	0.0665 - 0.140	Accepted	---
**AM-S-W1603**	0.940	0.0380	0.119	0.690 - 1.19	Accepted	---

Statistics

Serum PBDE IUPAC # 153 (µg/L)

All methods	AM-S-W1601	AM-S-W1602	AM-S-W1603
N	13	13	13
Robust mean Algo A	0.490	0.103	0.940
Robust STDev	0.0349	0.0167	0.110
Median	0.492	0.101	0.940
STDev from MAD	0.0386	0.0163	0.108
Arithmetic mean	0.492	0.104	0.941
STDev	0.0344	0.0205	0.0975
Comparison AM-S-W1505	0.485		
	0.0430		
CV or Variability	7.1%	16.3%	11.7%

GC-MS CI	AM-S-W1601	AM-S-W1602	AM-S-W1603
N	4	4	4
Robust mean Algo A	0.504	0.103	0.971
Robust STDev	0.0433	0.0347	0.0617
Median	0.497	0.104	0.976
STDev from MAD	0.0360	0.0313	0.0598
Arithmetic mean	0.507	0.103	0.971
STDev	0.0441	0.0306	0.0544
CV or Variability	8.6%	33.6%	6.4%

GC-MS EI	AM-S-W1601	AM-S-W1602	AM-S-W1603
N	4	4	4
Robust mean Algo A	0.508	0.106	0.978
Robust STDev	0.0217	0.00714	0.0625
Median	0.505	0.106	0.975
STDev from MAD	0.0193	0.00593	0.0489
Arithmetic mean	0.508	0.108	0.993
STDev	0.0191	0.00835	0.0815
CV or Variability	4.3%	6.7%	6.4%

GC-MS-MS	AM-S-W1601	AM-S-W1602	AM-S-W1603
N	3	3	3
Robust mean Algo A	0.458	0.0910	0.925
Robust STDev	0.00206	0.00468	0.140
Median	0.458	0.0900	0.897
STDev from MAD	0.00163	0.00371	0.118
Arithmetic mean	0.474	0.0928	0.925
STDev	0.0280	0.00718	0.124
CV or Variability	0.4%	5.1%	15.2%

When fewer than 20 results were considered for statistical treatment of all or a sub-sample of results, the accuracy of statistical data may be questionable.

Distribution
Serum PBDE IUPAC # 153 (µg/L)

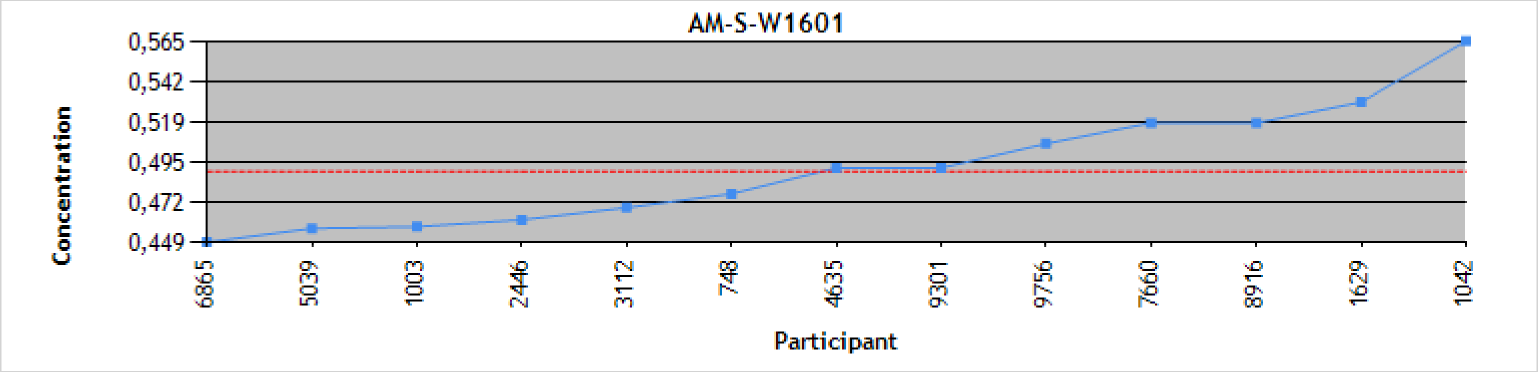

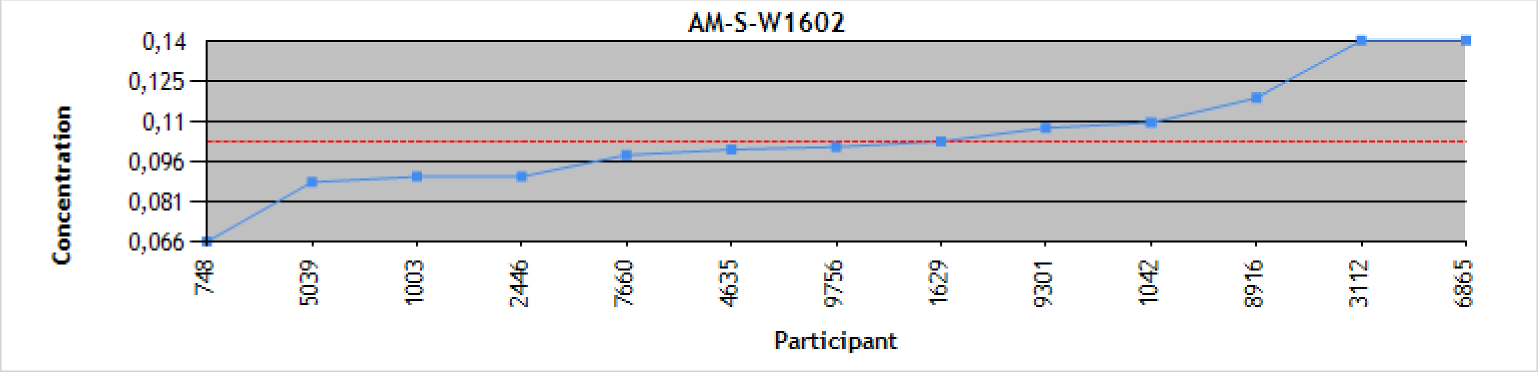

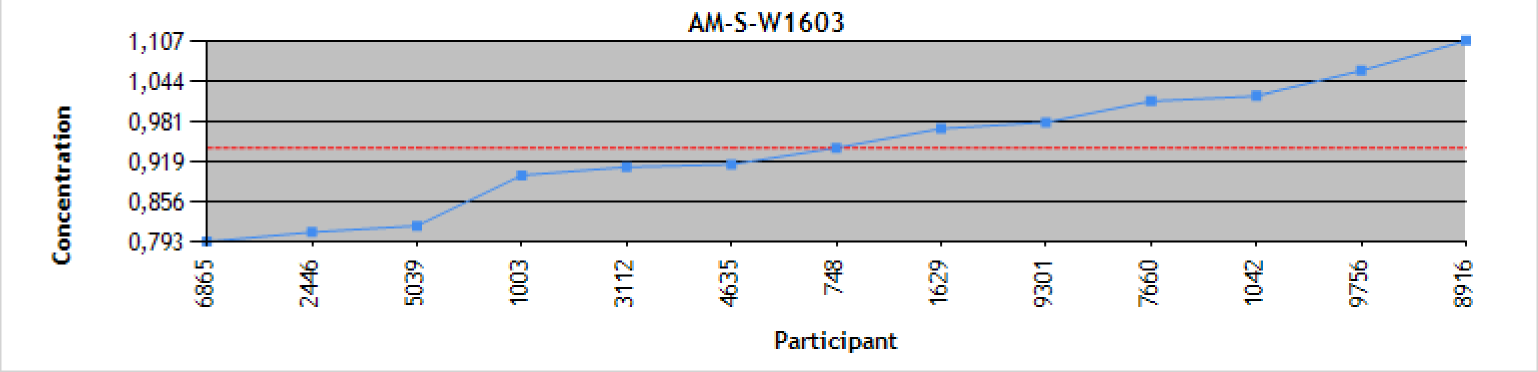

Individual results

Serum PBDE IUPAC # 154 (µg/L)

Round #2016-01

Participant	AM-S-W1601	z'-score	AM-S-W1602	z'-score	AM-S-W1603	z'-score	Method
**748**	0.224	**-2.15**	0.633	**-2.03**	0.0917	-1.93	GC-MS CI
**1042**	0.322	0.19	0.858	0.51	0.154	0.40	GC-MS CI
**1629**	0.327	0.31	0.823	0.11	0.177	1.28	GC-MS EI
**2446**	0.293	-0.50	0.794	-0.21	0.133	-0.38	ND
**3112**	0.254	-1.43	0.258	**-6.26**	0.114	-1.09	GC-MS CI
**4635**	0.338	0.57	0.874	0.69	0.156	0.49	GC-MS EI
**5039**	0.381	1.59	0.827	0.16	0.115	-1.06	GC-MS-MS
**6865**	0.344	0.72	0.809	-0.05	0.193	1.88	ND
**8916**	0.316	0.05	0.837	0.27	0.162	0.72	GC-MS EI
**9301**	0.297	-0.41	0.787	-0.29	0.137	-0.23	GC-MS EI

	Assigned value	Standard uncertainty	σ pt	Acceptable range	K-S (Lilliefors)	Species
**AM-S-W1601**	0.314	0.0151	0.0391	0.230 - 0.398	Accepted	---
**AM-S-W1602**	0.813	0.0172	0.0869	0.636 - 0.990	Accepted	---
**AM-S-W1603**	0.143	0.0138	0.0227	0.0898 - 0.196	Accepted	---

Statistics

Serum PBDE IUPAC # 154 (µg/L)

All methods	AM-S-W1601	AM-S-W1602	AM-S-W1603
N	10	10	10
Robust mean Algo A	0.314	0.813	0.143
Robust STDev	0.0382	0.0435	0.0350
Median	0.319	0.816	0.145
STDev from MAD	0.0349	0.0378	0.0349
Arithmetic mean	0.310	0.750	0.143
STDev	0.0452	0.185	0.0311
Comparison AM-S-W1505	0.297		
	0.0353		
CV or Variability	12.2%	5.4%	24.4%

GC-MS CI	AM-S-W1601	AM-S-W1602	AM-S-W1603
N	3	3	3
Robust mean Algo A	0.266	0.583	0.120
Robust STDev	0.0562	0.344	0.0356
Median	0.254	0.633	0.114
STDev from MAD	0.0445	0.333	0.0331
Arithmetic mean	0.267	0.583	0.120
STDev	0.0501	0.303	0.0314
CV or Variability	21.1%	58.9%	29.7%

GC-MS EI	AM-S-W1601	AM-S-W1602	AM-S-W1603
N	4	4	4
Robust mean Algo A	0.320	0.830	0.158
Robust STDev	0.0198	0.0408	0.0188
Median	0.322	0.830	0.159
STDev from MAD	0.0163	0.0371	0.0156
Arithmetic mean	0.320	0.830	0.158
STDev	0.0175	0.0360	0.0166
CV or Variability	6.2%	4.9%	11.9%

When fewer than 20 results were considered for statistical treatment of all or a sub-sample of results, the accuracy of statistical data may be questionable.

Distribution

Serum PBDE IUPAC # 154 (µg/L)

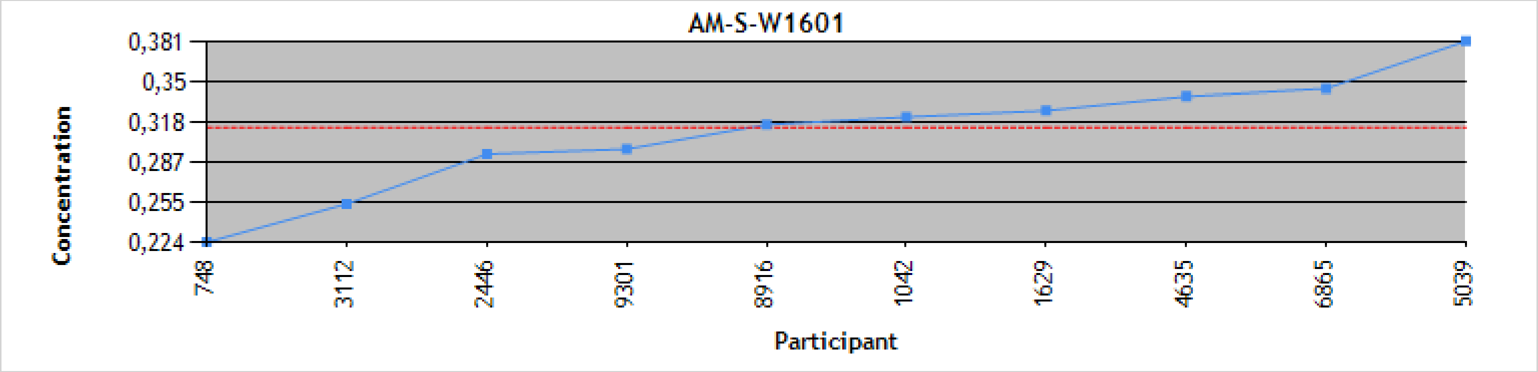

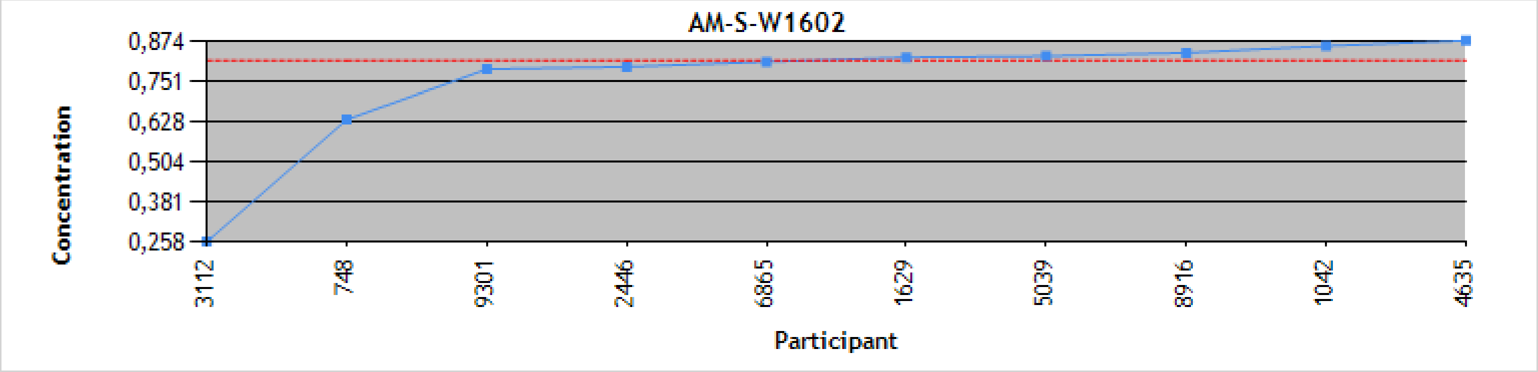

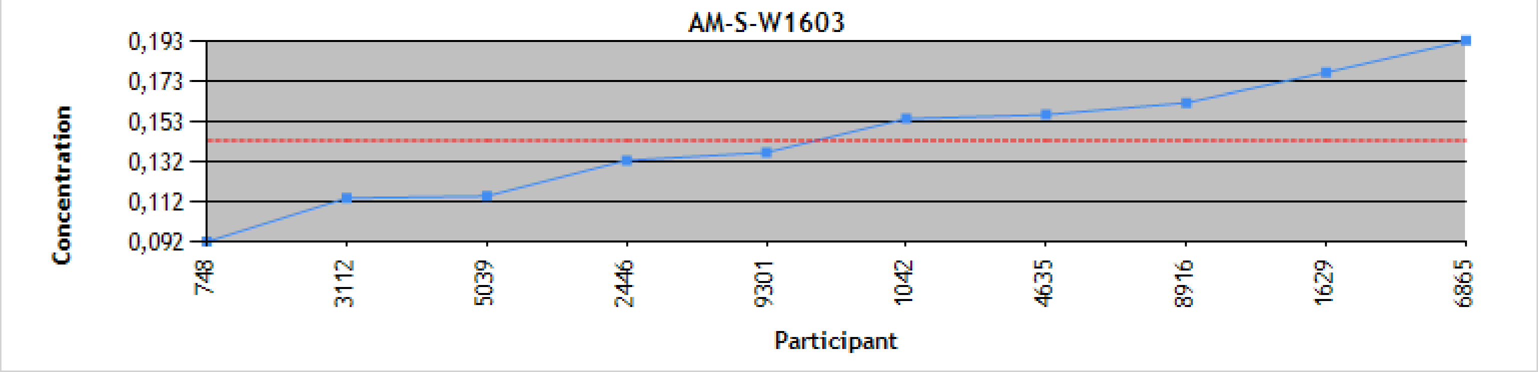

Individual results

Serum PBDE IUPAC # 183 (µg/L)

Round #2016-01

Participant	AM-S-W1601	z'-score	AM-S-W1602	z'-score	AM-S-W1603	z'-score	Method
**748**	0.390	-1.29	0.235	-1.63	0.512	-1.34	GC-MS CI
**1042**	0.600	0.79	0.402	0.68	0.896	1.18	GC-MS CI
**1629**	0.549	0.29	0.356	0.04	0.799	0.54	GC-MS EI
**2446**	0.525	0.05	0.411	0.80	0.695	-0.14	ND
**3112**	0.421	-0.98	0.0910	**-3.61**	0.631	-0.56	GC-MS CI
**4635**	0.534	0.14	0.342	-0.15	0.842	0.83	GC-MS EI
**5039**	0.485	-0.35	0.385	0.45	0.485	-1.51	GC-MS-MS
**9301**	0.548	0.28	0.363	0.14	0.838	0.80	GC-MS EI

	Assigned value	Standard uncertainty	σ pt	Acceptable range	K-S (Lilliefors)	Species
**AM-S-W1601**	0.520	0.0247	0.0977	0.318 - 0.722	Accepted	---
**AM-S-W1602**	0.353	0.0246	0.0683	0.208 - 0.498	Accepted	---
**AM-S-W1603**	0.716	0.0760	0.132	0.411 - 1.02	Accepted	---

Statistics
Serum PBDE IUPAC # 183 (µg/L)

All methods	AM-S-W1601	AM-S-W1602	AM-S-W1603
N	8	8	8
Robust mean Algo A	0.520	0.353	0.716
Robust STDev	0.0558	0.0556	0.172
Median	0.530	0.360	0.747
STDev from MAD	0.0474	0.0509	0.156
Arithmetic mean	0.506	0.323	0.712
STDev	0.0703	0.109	0.157
Comparison AM-S-W1505	0.479		
	0.102		
CV or Variability	10.7%	15.8%	24.0%

GC-MS CI	AM-S-W1601	AM-S-W1602	AM-S-W1603
N	3	3	3
Robust mean Algo A	0.434	0.243	0.680
Robust STDev	0.0580	0.177	0.223
Median	0.421	0.235	0.631
STDev from MAD	0.0460	0.214	0.176
Arithmetic mean	0.470	0.243	0.680
STDev	0.113	0.156	0.197
CV or Variability	13.4%	72.8%	32.8%

GC-MS EI	AM-S-W1601	AM-S-W1602	AM-S-W1603
N	3	3	3
Robust mean Algo A	0.548	0.354	0.836
Robust STDev	0.00187	0.0121	0.00749
Median	0.548	0.356	0.838
STDev from MAD	0.00148	0.0104	0.00593
Arithmetic mean	0.544	0.354	0.826
STDev	0.00839	0.0107	0.0238
CV or Variability	0.3%	3.4%	0.9%

When fewer than 20 results were considered for statistical treatment of all or a sub-sample of results, the accuracy of statistical data may be questionable.

Distribution

Serum PBDE IUPAC # 183 (µg/L)

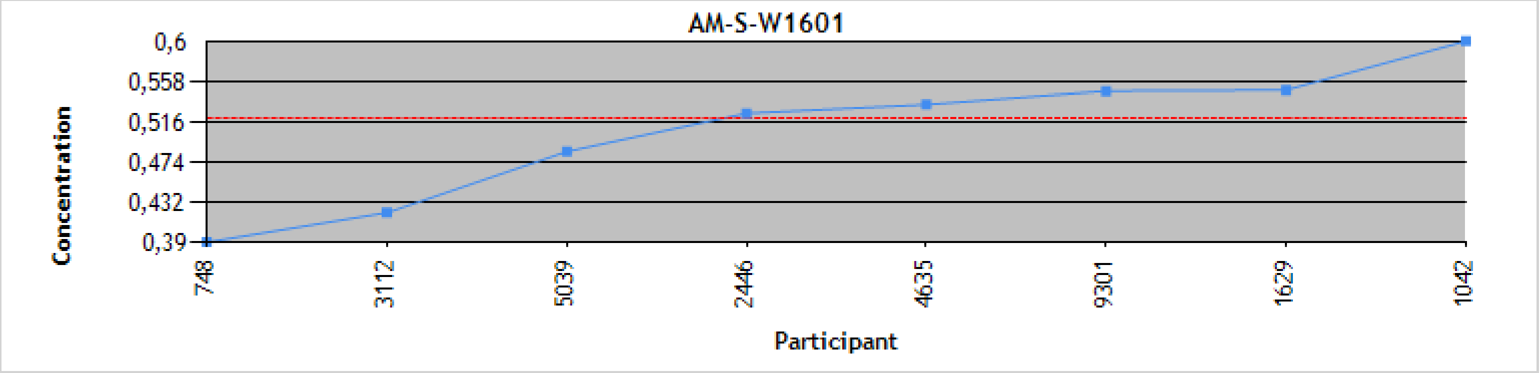

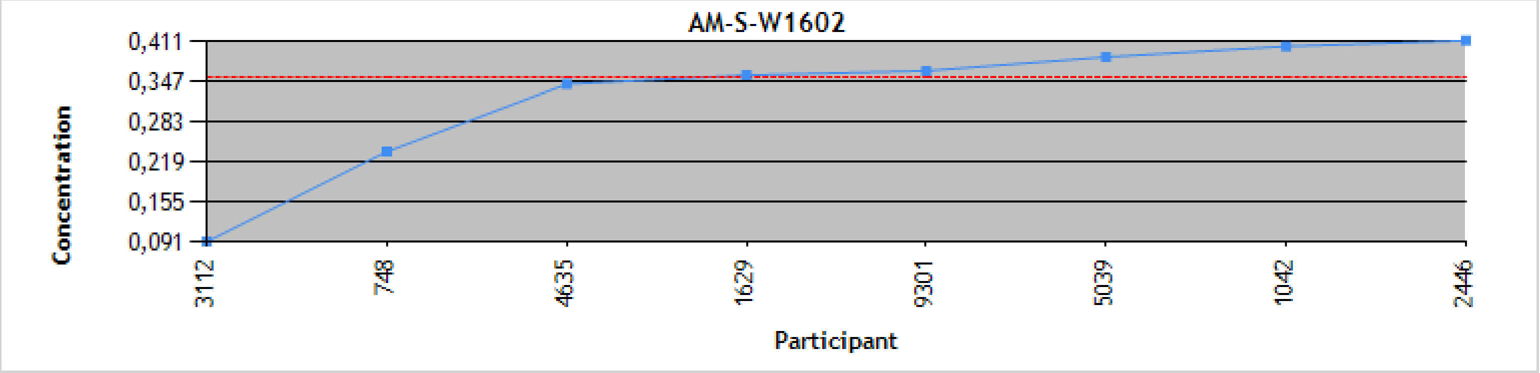

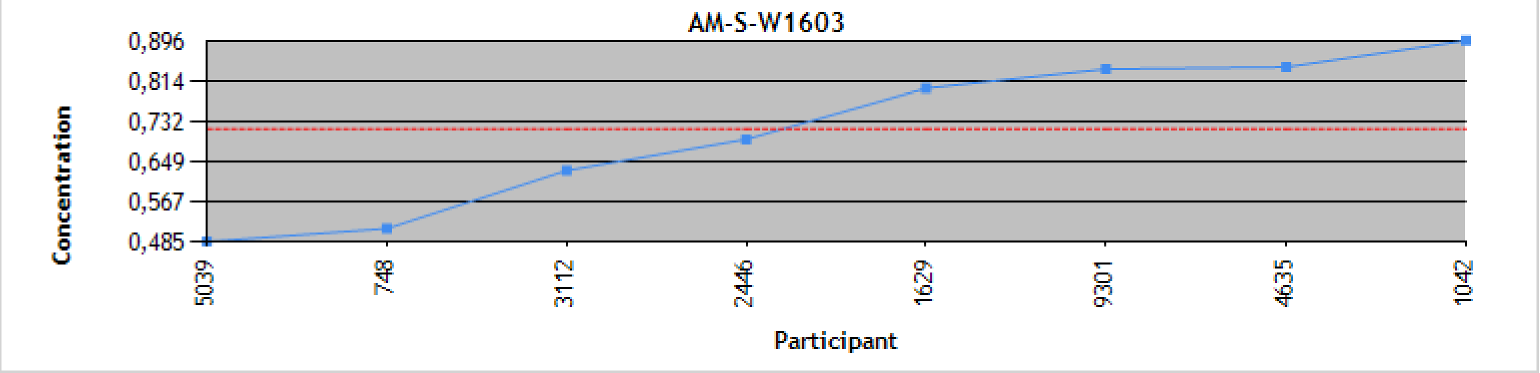

Individual results
Serum PBDE IUPAC # 209 (µg/L)
Round #2016-01

Participant	AM-S-W1601	z'-score	AM-S-W1602	z'-score	AM-S-W1603	z'-score	Method
**748**	1.26	-0.55	1.03	-0.47	0.259	-0.24	GC-MS CI
**1042**	1.47	0.18	1.24	0.34	0.302	0.37	GC-MS CI
**1629**	2.08	**2.27**	1.29	0.55	0.136	-1.99	GC-MS EI
**2446**	0.583	**-2.86**	0.361	**-3.09**	---	---	ND
**2724**	1.07	-1.20	0.840	-1.21	0.230	-0.65	GC-MS CI
**3112**	1.40	-0.07	1.05	-0.38	0.263	-0.18	GC-MS CI
**4635**	1.71	0.98	1.44	1.15	0.358	1.16	GC-MS EI
**9301**	1.60	0.62	1.64	1.92	0.445	**2.40**	GC-MS EI

	Assigned value	Standard uncertainty	σ pt	Acceptable range	K-S (Lilliefors)	Species
**AM-S-W1601**	1.42	0.165	0.242	0.834 - 2.01	Accepted	---
**AM-S-W1602**	1.15	0.160	0.199	0.639 - 1.66	Accepted	---
**AM-S-W1603**	0.276	0.0339	0.0617	0.135 - 0.417	Accepted	---

Statistics
Serum PBDE IUPAC # 209 (µg/L)

All methods	AM-S-W1601	AM-S-W1602	AM-S-W1603
N	8	8	7
Robust mean Algo A	1.42	1.15	0.276
Robust STDev	0.373	0.362	0.0718
Median	1.44	1.15	0.263
STDev from MAD	0.331	0.327	0.0578
Arithmetic mean	1.40	1.11	0.285
STDev	0.448	0.393	0.0980
Comparison AM-S-W1505	1.34		
	0.280		
CV or Variability	26.2%	31.6%	26.0%

GC-MS CI	AM-S-W1601	AM-S-W1602	AM-S-W1603
N	4	4	4
Robust mean Algo A	1.31	1.04	0.262
Robust STDev	0.189	0.184	0.0315
Median	1.33	1.04	0.261
STDev from MAD	0.157	0.154	0.0245
Arithmetic mean	1.30	1.04	0.263
STDev	0.177	0.163	0.0296
CV or Variability	14.5%	17.7%	12.0%

GC-MS EI	AM-S-W1601	AM-S-W1602	AM-S-W1603
N	3	3	3
Robust mean Algo A	1.75	1.46	0.322
Robust STDev	0.198	0.199	0.163
Median	1.71	1.44	0.358
STDev from MAD	0.157	0.227	0.129
Arithmetic mean	1.80	1.46	0.313
STDev	0.254	0.175	0.159
CV or Variability	11.3%	13.7%	50.5%

When fewer than 20 results were considered for statistical treatment of all or a sub-sample of results, the accuracy of statistical data may be questionable.

Distribution

Serum PBDE IUPAC # 209 (µg/L)

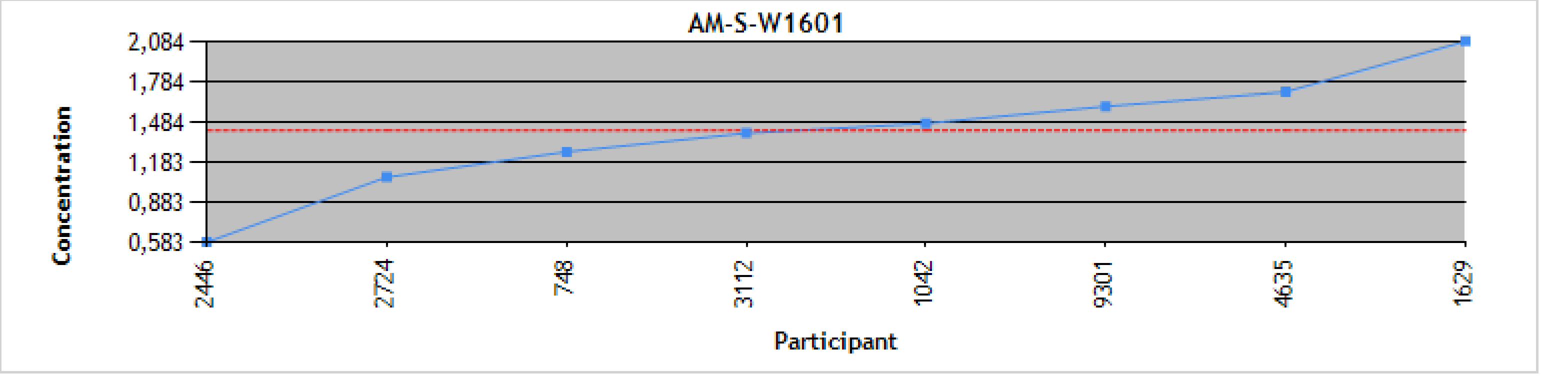

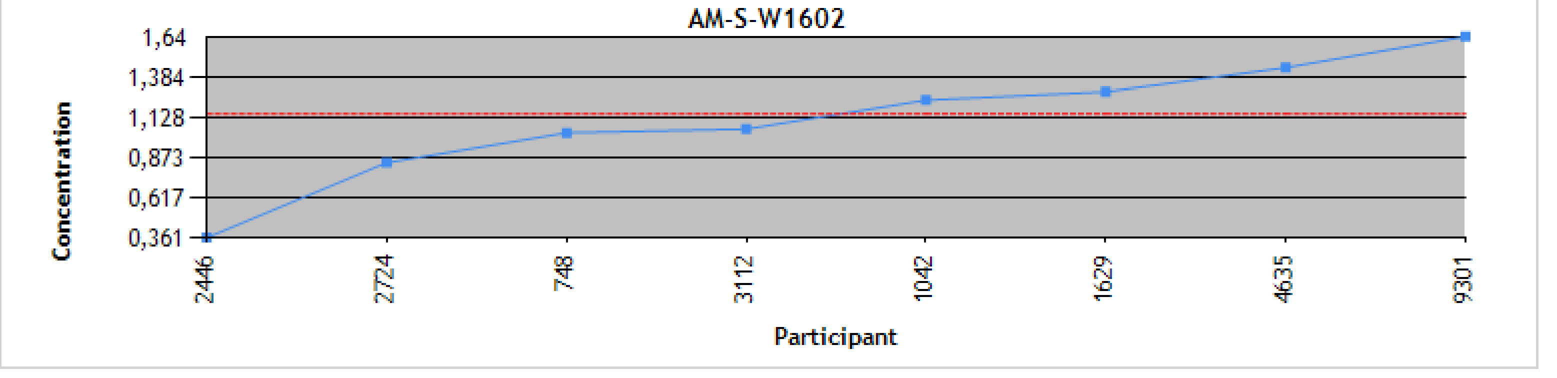

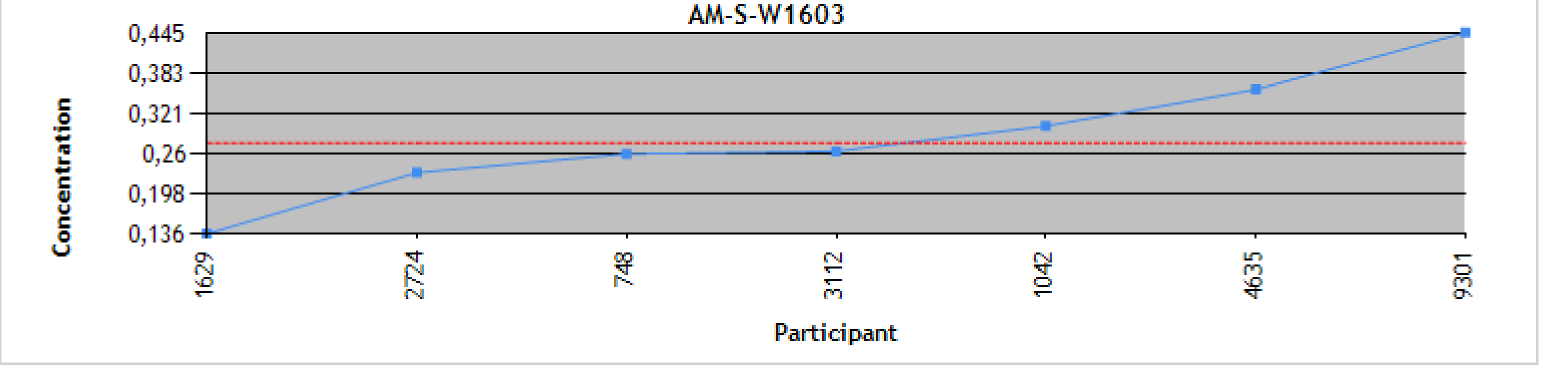

Individual results
Serum PCB IUPAC # 28 (µg/L)
Round #2016-01

Participant	AM-S-W1601	z'-score	AM-S-W1602	z'-score	AM-S-W1603	z'-score	Method
270	0.974	0.70	0.363	1.93	0.823	0.38	ND
332	0.821	-0.25	0.240	-0.23	0.753	-0.11	ND
748	0.848	-0.09	0.259	0.11	0.765	-0.03	GC
1042	0.855	-0.04	0.250	-0.06	0.750	-0.14	GC-MS CI
1629	1.10	1.46	0.314	1.06	0.973	1.45	GC-MS-MS
2446	0.831	-0.19	0.227	-0.46	0.751	-0.13	ND
3112	0.722	-0.87	0.246	-0.12	0.634	-0.96	GC-MS CI
4635	0.927	0.40	0.279	0.46	0.796	0.19	GC-MS EI
5039	1.18	1.95	0.260	0.12	0.970	1.42	GC-MS-MS
6865	0.897	0.22	0.228	-0.44	0.789	0.14	ND
7660	0.911	0.30	0.264	0.20	0.778	0.06	GC-MS CI
8559	0.624	-1.48	0.248	-0.09	0.742	-0.19	ND
8916	0.584	-1.73	0.223	-0.53	0.527	-1.72	GC

	Assigned value	Standard uncertainty	σ pt	Acceptable range	K-S (Lilliefors)	Species
AM-S-W1601	0.862	0.0434	0.155	0.540 - 1.18	Accepted	---
AM-S-W1602	0.253	0.00806	0.0563	0.139 - 0.367	Rejected[1]	---
AM-S-W1603	0.769	0.0147	0.140	0.487 - 1.05	Accepted	---

Statistics

Serum PCB IUPAC # 28 (µg/L)

All methods	AM-S-W1601	AM-S-W1602	AM-S-W1603
N	13	13	13
Robust mean Algo A	0.862	0.253	0.769
Robust STDev	0.125	0.0233	0.0424
Median	0.855	0.250	0.765
STDev from MAD	0.107	0.0218	0.0356
Arithmetic mean	0.867	0.262	0.773
STDev	0.166	0.0389	0.117
Comparison AM-S-W1505	0.908		
	0.140		
CV or Variability	14.5%	9.2%	5.5%

GC-MS CI	AM-S-W1601	AM-S-W1602	AM-S-W1603
N	3	3	3
Robust mean Algo A	0.832	0.251	0.738
Robust STDev	0.105	0.00655	0.0526
Median	0.855	0.250	0.750
STDev from MAD	0.0835	0.00519	0.0417
Arithmetic mean	0.829	0.253	0.720
STDev	0.0971	0.00966	0.0762
CV or Variability	12.7%	2.6%	7.1%

When fewer than 20 results were considered for statistical treatment of all or a sub-sample of results, the accuracy of statistical data may be questionable.

Distribution

Serum PCB IUPAC # 28 (µg/L)

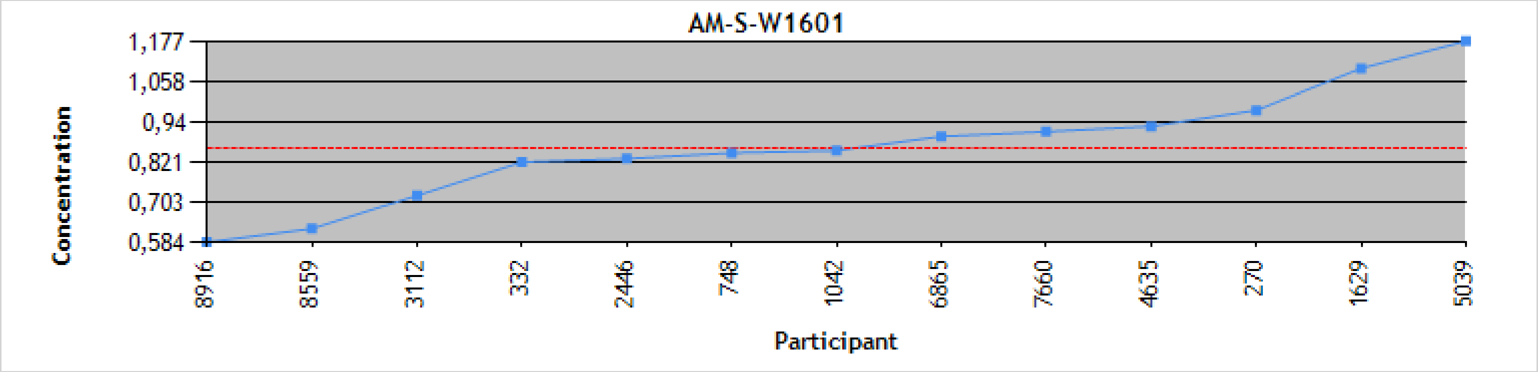

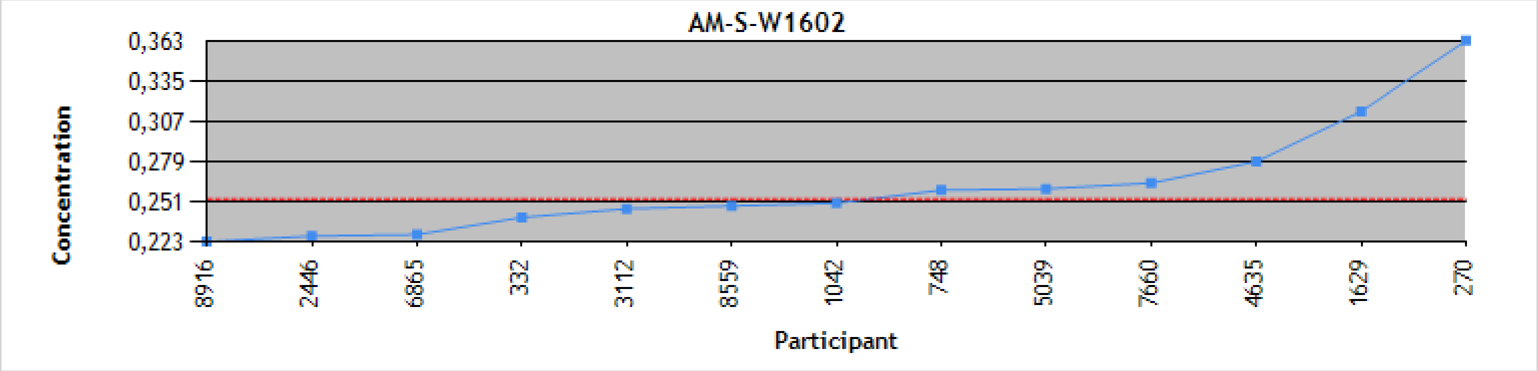

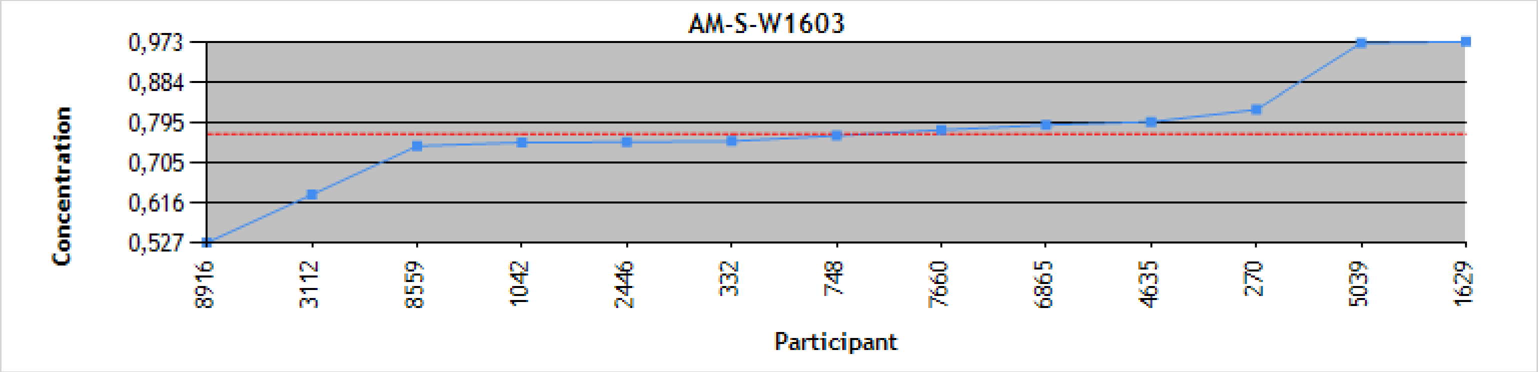

Individual results

Serum PCB IUPAC # 52 (µg/L)

Round #2016-01

Participant	AM-S-W1601	z'-score	AM-S-W1602	z'-score	AM-S-W1603	z'-score	Method
**270**	<LD	---	0.851	0.45	0.418	0.25	ND
**1042**	<LD	---	0.776	-0.02	0.376	-0.27	GC-MS CI
**1629**	0.00110	-1.02	0.893	0.71	0.451	0.65	GC-MS-MS
**2446**	0.00600	-0.84	0.711	-0.43	0.396	-0.02	ND
**3112**	---	---	0.458	**-2.01**	0.218	**-2.22**	GC-MS CI
**5039**	0.0780	1.85	0.739	-0.25	0.415	0.21	GC-MS-MS
**6865**	<LQ	---	0.775	-0.03	0.323	-0.92	ND
**7660**	<LD	---	0.849	0.44	0.397	-0.01	GC-MS CI
**8559**	<LD	---	0.766	-0.08	0.433	0.43	ND

	Assigned value	Standard uncertainty	σ pt	Acceptable range	K-S (Lilliefors)	Species
**AM-S-W1601**	0.0284	0.0249	0.00567	0.00 - 0.0794	Accepted	---
**AM-S-W1602**	0.779	0.0360	0.156	0.459 - 1.10	Accepted	---
**AM-S-W1603**	0.398	0.0159	0.0795	0.236 - 0.560	Accepted	---

PCB IUPAC # 52 is not included in the scope of our accreditation.

Statistics

Serum PCB IUPAC # 52 (µg/L)

All methods	AM-S-W1601	AM-S-W1602	AM-S-W1603
N	3	9	9
Robust mean Algo A	0.00800	0.779	0.398
Robust STDev	0.00917	0.0863	0.0382
Median	0.00600	0.775	0.397
STDev from MAD	0.00727	0.0949	0.0312
Arithmetic mean	0.0284	0.758	0.381
STDev	0.0431	0.127	0.0712
CV or Variability	151.8%	11.1%	9.6%

GC-MS CI	AM-S-W1601	AM-S-W1602	AM-S-W1603
N	NA	3	3
Robust mean Algo A	NA	0.747	0.368
Robust STDev	NA	0.135	0.0385
Median	NA	0.776	0.376
STDev from MAD	NA	0.107	0.0305
Arithmetic mean	NA	0.694	0.330
STDev	NA	0.208	0.0979
CV or Variability	NA	18.1%	10.5%

When fewer than 20 results were considered for statistical treatment of all or a sub-sample of results, the accuracy of statistical data may be questionable.

For PCB IUPAC # 52 in material AM-S-W1601: according to the limit number of results, the arithmetic mean have been stated more appropriate for assigned value determination.

Distribution

Serum PCB IUPAC # 52 (µg/L)

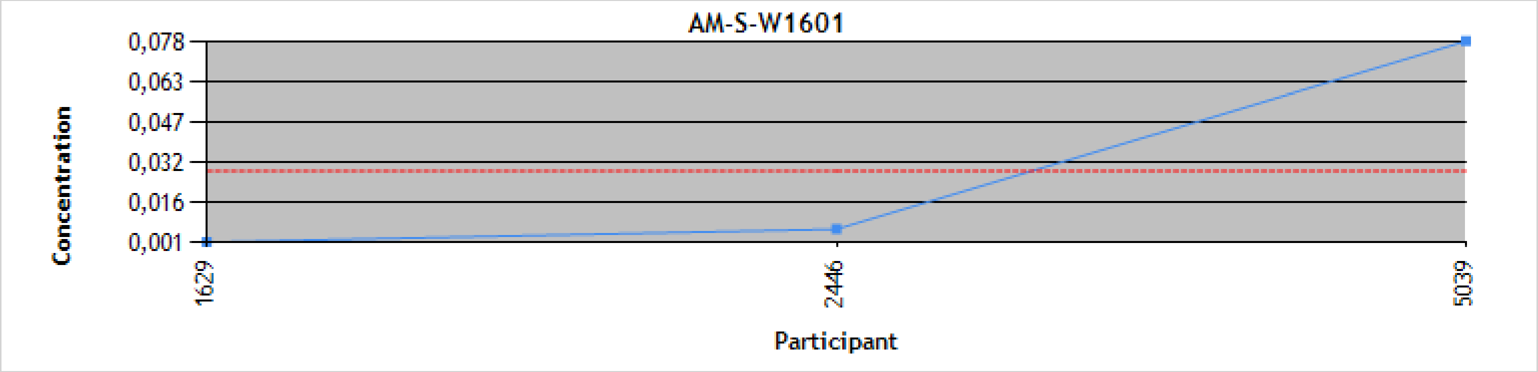

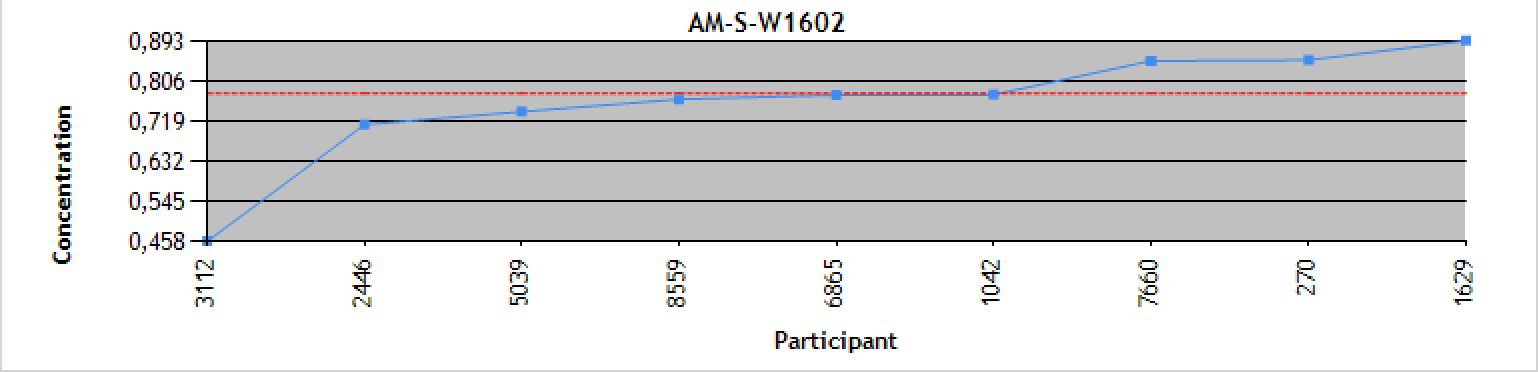

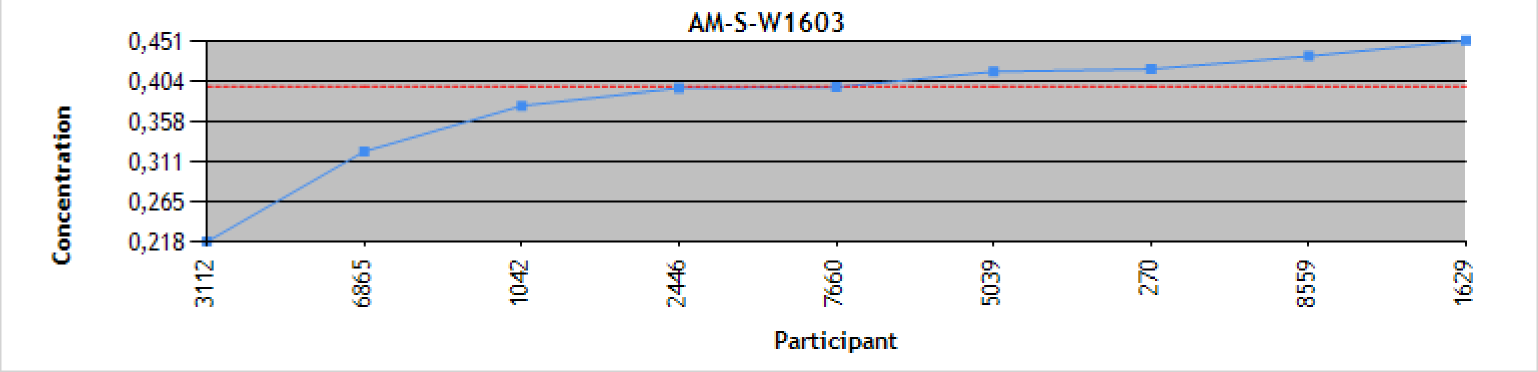

Individual results

Serum PCB IUPAC # 74 (µg/L)

Round #2016-01

Participant	AM-S-W1601	z'-score	AM-S-W1602	z'-score	AM-S-W1603	z'-score	Method
**332**	0.576	0.14	0.939	0.85	0.476	0.49	ND
**1003**	0.515	-0.81	0.820	-0.50	0.443	-0.16	GC-MS-MS
**1042**	0.548	-0.30	0.827	-0.41	0.443	-0.16	GC-MS CI
**1629**	0.665	1.54	1.01	1.69	0.517	1.29	GC-MS-MS
**3112**	0.623	0.88	0.910	0.52	0.457	0.12	GC-MS CI
**4635**	0.579	0.19	0.859	-0.06	0.468	0.33	GC-MS EI
**6865**	0.593	0.41	0.807	-0.64	0.404	-0.92	ND
**7660**	0.567	-0.01	0.872	0.09	0.445	-0.12	GC-MS CI
**8559**	0.467	-1.57	0.777	-0.98	0.440	-0.21	ND
**8916**	0.420	**-2.30**	0.638	**-2.55**	0.335	**-2.26**	GC
**9301**	0.620	0.83	1.02	1.76	0.516	1.27	GC-MS-MS

	Assigned value	Standard uncertainty	σ pt	Acceptable range	K-S (Lilliefors)	Species
**AM-S-W1601**	0.567	0.0254	0.0586	0.439 - 0.695	Accepted	---
**AM-S-W1602**	0.864*	0.0334	0.0822	0.687 - 1.04	Accepted	---
**AM-S-W1603**	0.451	0.0139	0.0494	0.348 - 0.554	Accepted	---

* The assigned value obtained exceeds the range of concentration from our scope of accreditation.

Statistics
Serum PCB IUPAC # 74 (µg/L)

All methods	AM-S-W1601	AM-S-W1602	AM-S-W1603
N	11	11	11
Robust mean Algo A	0.567	0.864	0.451
Robust STDev	0.0675	0.0886	0.0369
Median	0.576	0.859	0.445
STDev from MAD	0.0653	0.0771	0.0342
Arithmetic mean	0.561	0.862	0.449
STDev	0.0713	0.109	0.0505
Comparison AM-S-W1505	0.581		
	0.0691		
CV or Variability	11.9%	10.2%	8.2%

GC-MS CI	AM-S-W1601	AM-S-W1602	AM-S-W1603
N	3	3	3
Robust mean Algo A	0.574	0.870	0.446
Robust STDev	0.0352	0.0469	0.00399
Median	0.567	0.872	0.445
STDev from MAD	0.0279	0.0570	0.00316
Arithmetic mean	0.579	0.870	0.448
STDev	0.0392	0.0413	0.00767
CV or Variability	6.1%	5.4%	0.9%

GC-MS-MS	AM-S-W1601	AM-S-W1602	AM-S-W1603
N	3	3	3
Robust mean Algo A	0.602	1.01	0.516
Robust STDev	0.0848	0.0112	0.00225
Median	0.620	1.01	0.516
STDev from MAD	0.0672	0.00890	0.00178
Arithmetic mean	0.600	0.951	0.492
STDev	0.0771	0.114	0.0425
CV or Variability	14.1%	1.1%	0.4%

When fewer than 20 results were considered for statistical treatment of all or a sub-sample of results, the accuracy of statistical data may be questionable.

Distribution

Serum PCB IUPAC # 74 (µg/L)

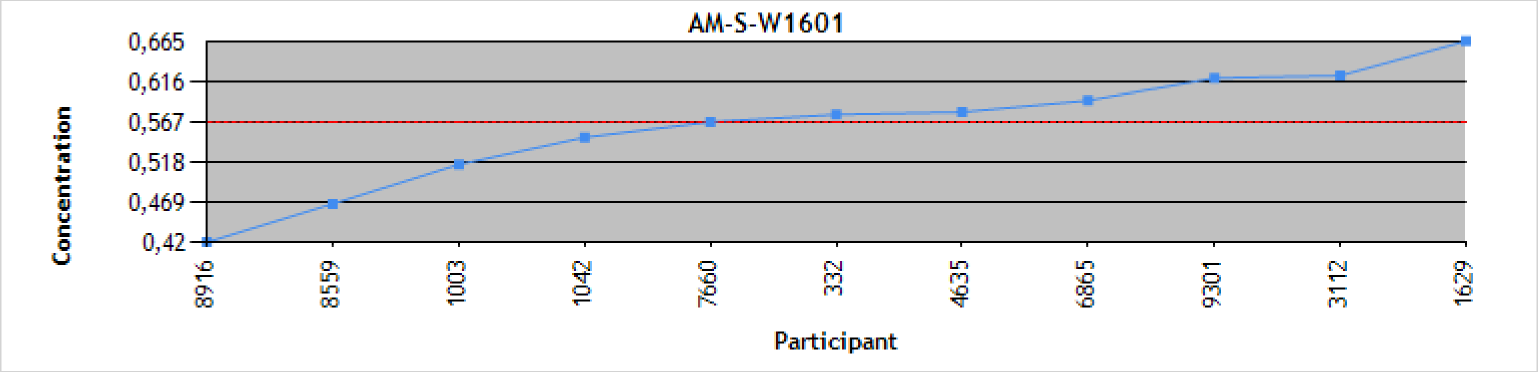

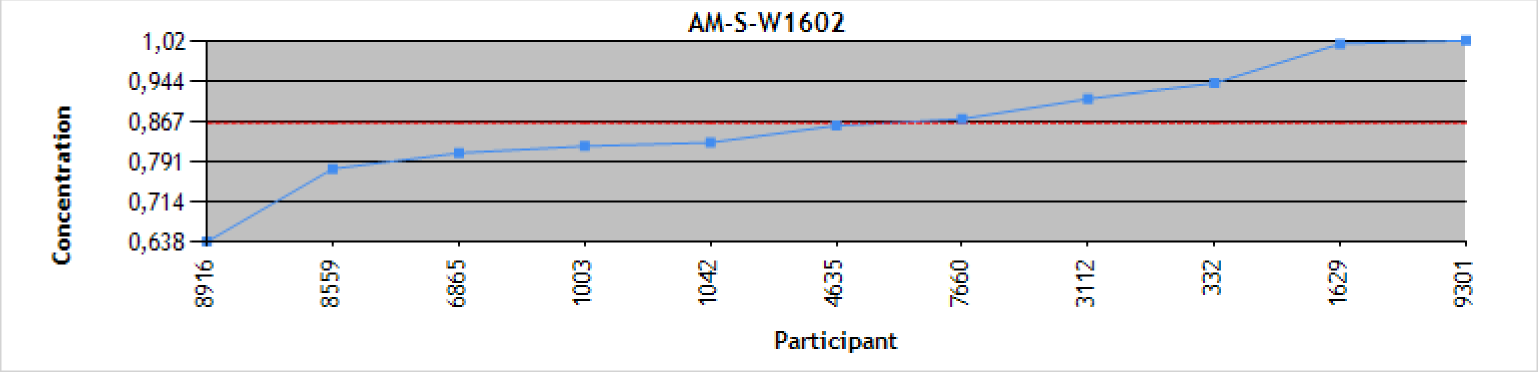

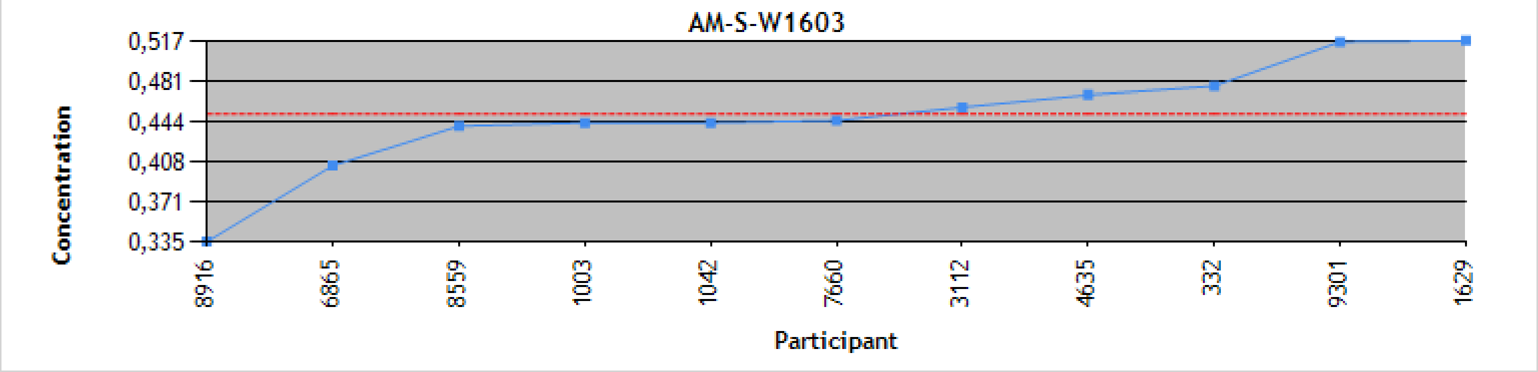

Individual results

Serum PCB IUPAC # 99 (µg/L)

Round #2016-01

Participant	AM-S-W1601	z'-score	AM-S-W1602	z'-score	AM-S-W1603	z'-score	Method
**270**	0.419	0.48	0.141	0.38	0.663	0.05	ND
**332**	0.366	-0.53	0.121	-0.57	0.641	-0.22	ND
**1003**	0.368	-0.50	0.141	0.38	0.692	0.41	GC-MS-MS
**1042**	0.398	0.09	0.132	-0.03	0.642	-0.21	GC-MS CI
**1629**	0.423	0.56	0.141	0.39	0.649	-0.13	GC-MS-MS
**3112**	0.455	1.16	0.133	0.00	0.652	-0.09	GC-MS CI
**4635**	0.453	1.12	0.145	0.57	0.745	1.06	GC-MS EI
**6865**	0.350	-0.84	0.0850	**-2.29**	0.669	0.12	ND
**7660**	0.403	0.17	0.134	0.03	0.667	0.10	GC-MS CI
**8559**	0.358	-0.69	<LD	---	0.810	1.86	ND
**8916**	0.360	-0.65	0.138	0.24	0.538	-1.49	GC
**9301**	0.377	-0.32	0.111	-1.05	0.619	-0.49	GC-MS-MS

	Assigned value	Standard uncertainty	σ pt	Acceptable range	K-S (Lilliefors)	Species
**AM-S-W1601**	0.394	0.0148	0.0503	0.289 - 0.499	Accepted	---
**AM-S-W1602**	0.133	0.00429	0.0205	0.0911 - 0.175	Accepted	---
**AM-S-W1603**	0.659	0.0103	0.0806	0.496 - 0.822	Accepted	---

Statistics

Serum PCB IUPAC # 99 (µg/L)

All methods	AM-S-W1601	AM-S-W1602	AM-S-W1603
N	12	11	12
Robust mean Algo A	0.394	0.133	0.659
Robust STDev	0.0410	0.0114	0.0286
Median	0.388	0.134	0.658
STDev from MAD	0.0426	0.0110	0.0235
Arithmetic mean	0.394	0.129	0.666
STDev	0.0368	0.0177	0.0660
Comparison AM-S-W1505	0.410		
	0.0396		
CV or Variability	10.4%	8.6%	4.3%

GC-MS CI	AM-S-W1601	AM-S-W1602	AM-S-W1603
N	3	3	3
Robust mean Algo A	0.405	0.133	0.654
Robust STDev	0.00859	0.000720	0.0140
Median	0.403	0.133	0.652
STDev from MAD	0.00681	0.000905	0.0144
Arithmetic mean	0.419	0.133	0.654
STDev	0.0314	0.000635	0.0124
CV or Variability	2.1%	0.5%	2.1%

GC-MS-MS	AM-S-W1601	AM-S-W1602	AM-S-W1603
N	3	3	3
Robust mean Algo A	0.381	0.141	0.653
Robust STDev	0.0168	0.000374	0.0416
Median	0.377	0.141	0.649
STDev from MAD	0.0133	0.000297	0.0440
Arithmetic mean	0.389	0.131	0.653
STDev	0.0297	0.0174	0.0367
CV or Variability	4.4%	0.3%	6.4%

When fewer than 20 results were considered for statistical treatment of all or a sub-sample of results, the accuracy of statistical data may be questionable.

Distribution

Serum PCB IUPAC # 99 (µg/L)

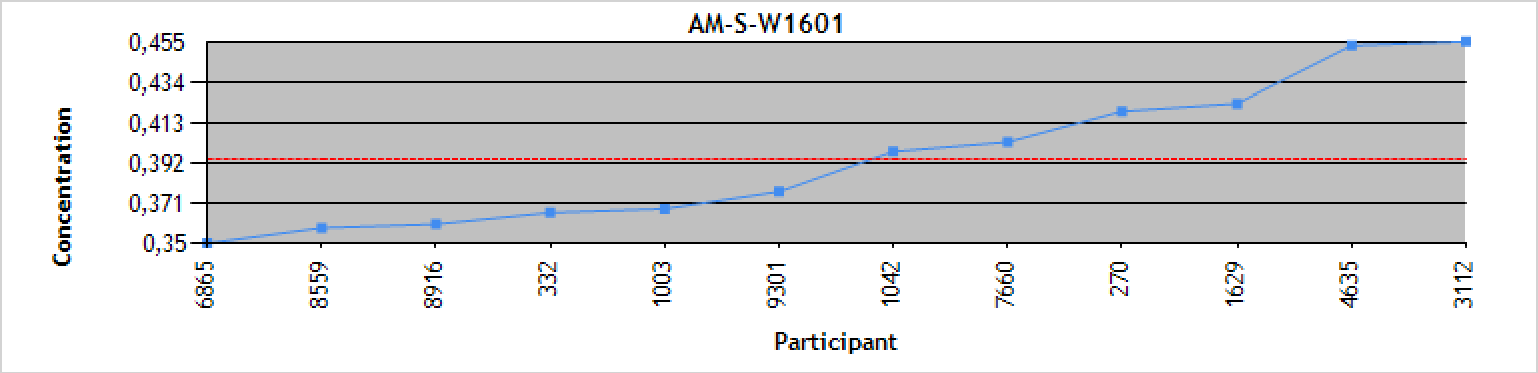

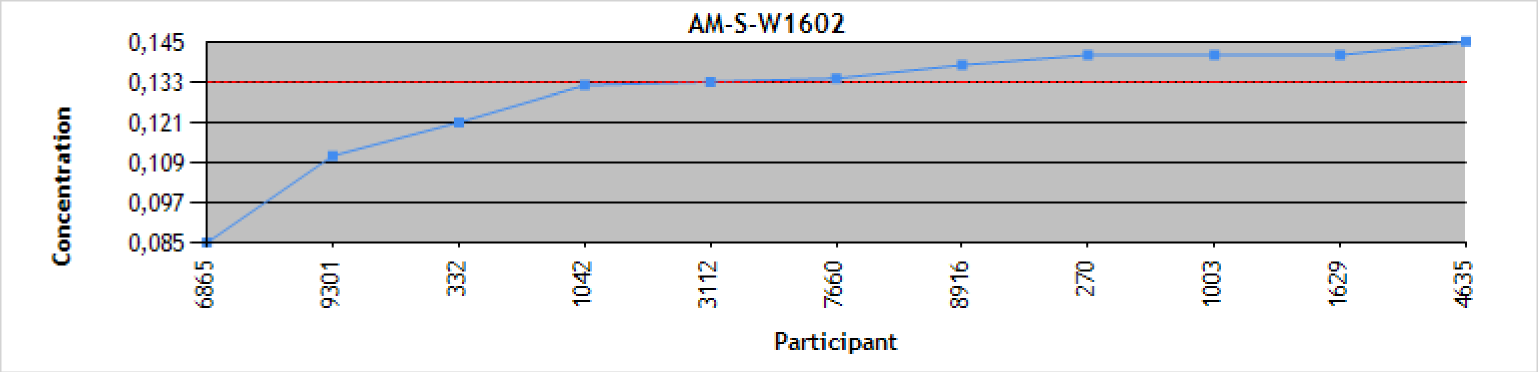

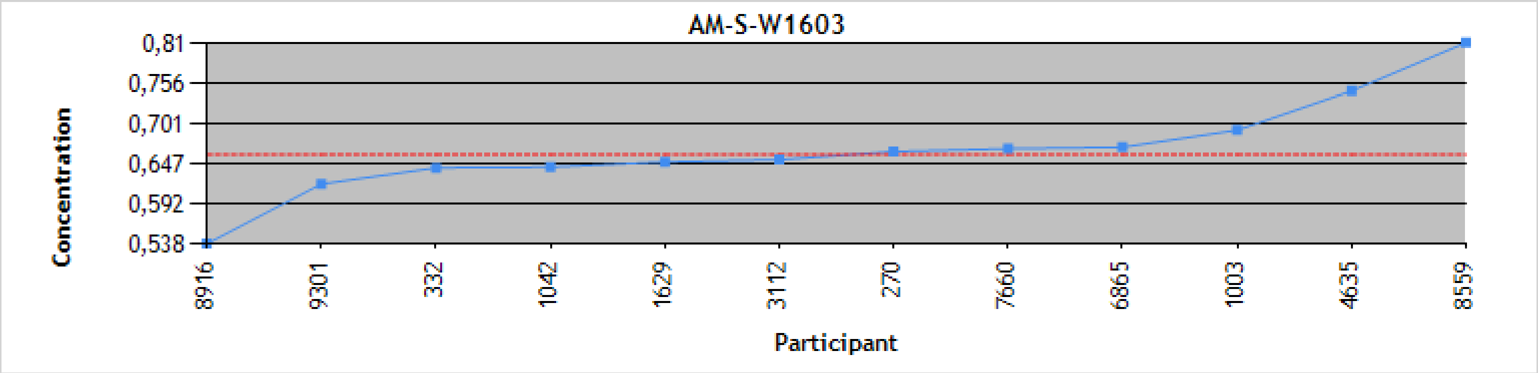

Individual results
Serum PCB IUPAC # 101 (µg/L)
Round #2016-01

Participant	AM-S-W1601	z'-score	AM-S-W1602	z'-score	AM-S-W1603	z'-score	Method
270	<LD	---	0.672	0.51	0.331	0.40	ND
1042	<LD	---	0.630	0.17	0.310	0.06	GC-MS CI
1629	0.00380	-0.98	0.649	0.32	0.295	-0.18	GC-MS-MS
2446	0.00400	-0.98	0.573	-0.29	0.282	-0.39	ND
5039	0.187	1.96	0.561	-0.39	0.328	0.35	GC-MS-MS
6865	<LQ	---	0.510	-0.80	0.282	-0.39	ND
7660	<LD	---	0.642	0.27	0.310	0.07	GC-MS CI
8559	<LD	---	0.649	0.32	0.340	0.55	ND
9301	<LD	---	0.569	-0.32	0.281	-0.40	GC-MS-MS

	Assigned value	Standard uncertainty	σ pt	Acceptable range	K-S (Lilliefors)	Species
AM-S-W1601	0.0649	0.0610	0.0130	0.00 - 0.190	Accepted	---
AM-S-W1602	0.609	0.0231	0.122	0.361 - 0.857	Accepted	---
AM-S-W1603	0.306	0.0108	0.0613	0.182 - 0.430	Accepted	---

PCB IUPAC # 101 is not included in the scope of our accreditation.

Statistics

Serum PCB IUPAC # 101 (µg/L)

All methods	AM-S-W1601	AM-S-W1602	AM-S-W1603
N	3	9	9
Robust mean Algo A	0.00408	0.609	0.306
Robust STDev	0.000374	0.0555	0.0259
Median	0.00400	0.630	0.310
STDev from MAD	0.000297	0.0622	0.0318
Arithmetic mean	0.0649	0.606	0.306
STDev	0.106	0.0544	0.0229
CV or Variability	162.8%	9.1%	8.5%

GC-MS-MS	AM-S-W1601	AM-S-W1602	AM-S-W1603
N	NA	3	3
Robust mean Algo A	NA	0.572	0.300
Robust STDev	NA	0.0153	0.0255
Median	NA	0.569	0.295
STDev from MAD	NA	0.0122	0.0202
Arithmetic mean	NA	0.593	0.301
STDev	NA	0.0486	0.0240
CV or Variability	NA	2.7%	8.5%

When fewer than 20 results were considered for statistical treatment of all or a sub-sample of results, the accuracy of statistical data may be questionable.

For PCB IUPAC #101: according to the limit number of results, the arithmetic mean have been stated more appropriate for assigned value determination.

Distribution

Serum PCB IUPAC # 101 (µg/L)

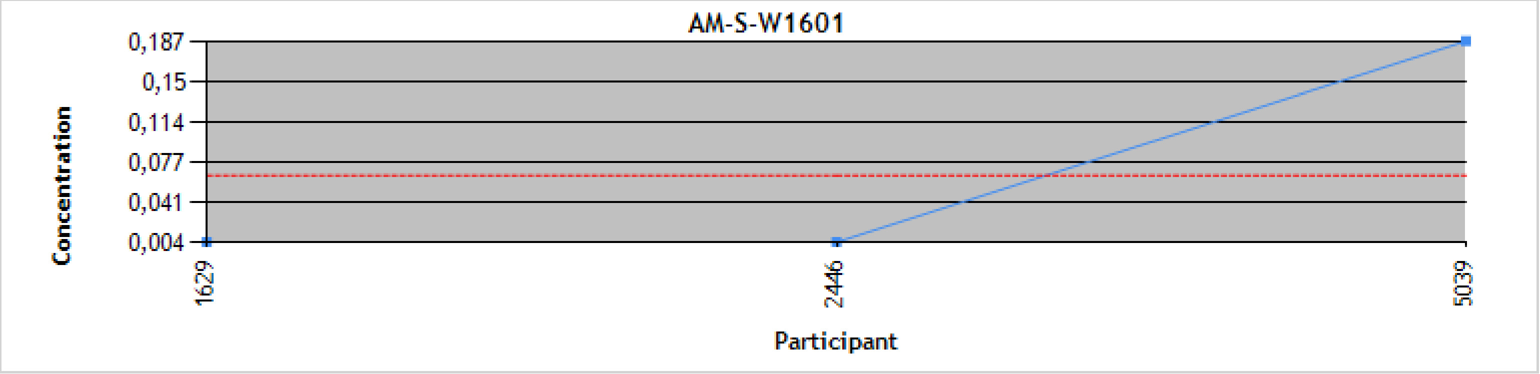

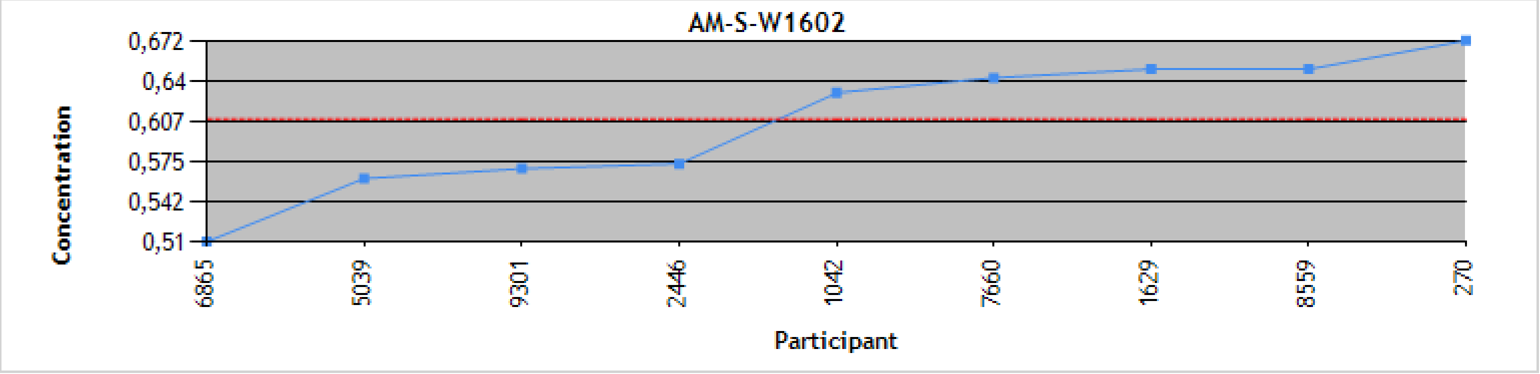

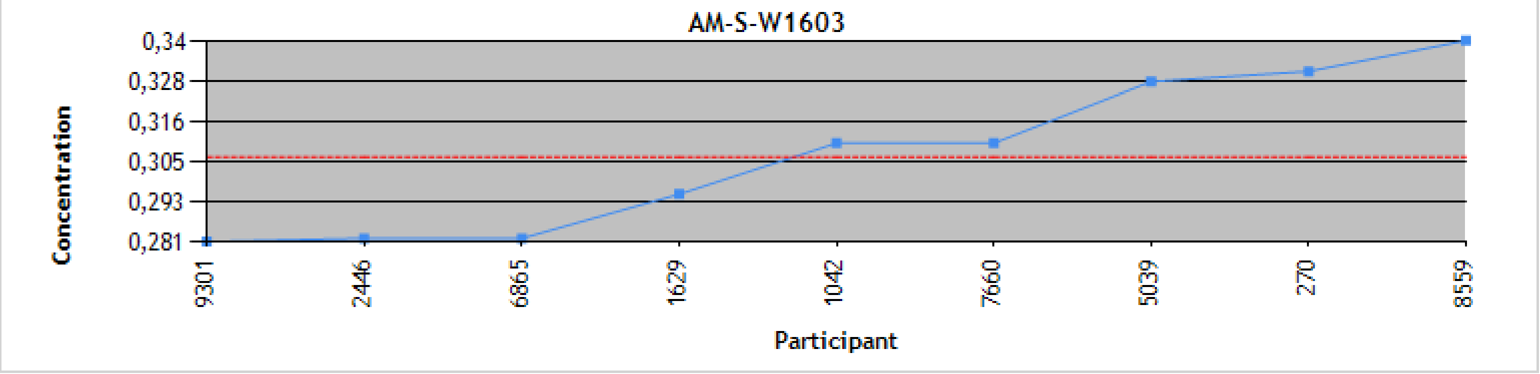

Individual results

Serum PCB IUPAC # 105 (µg/L)

Round #2016-01

Participant	AM-S-W1601	z'-score	AM-S-W1602	z'-score	AM-S-W1603	z'-score	Method
**270**	0.248	0.45	0.415	-0.15	0.136	0.22	ND
**332**	0.220	-0.52	0.402	-0.43	0.115	-0.94	ND
**1042**	0.248	0.45	0.441	0.41	0.135	0.19	GC-MS CI
**1629**	0.297	**2.17**	0.461	0.84	0.152	1.11	GC-MS-MS
**2446**	0.228	-0.24	0.415	-0.15	0.127	-0.28	ND
**3112**	0.208	-0.94	0.362	-1.29	0.104	-1.54	GC-MS CI
**4635**	0.255	0.70	0.443	0.45	0.139	0.39	GC-MS EI
**6865**	0.230	-0.17	0.408	-0.30	0.139	0.39	ND
**7660**	0.254	0.65	0.448	0.56	0.137	0.30	GC-MS CI
**8559**	0.185	-1.74	0.564	**3.05**	0.0880	**-2.43**	ND
**8916**	0.242	0.24	0.410	-0.26	0.140	0.44	GC
**9301**	0.216	-0.66	0.378	-0.94	0.116	-0.88	GC-MS-MS

	Assigned value	Standard uncertainty	σ pt	Acceptable range	K-S (Lilliefors)	Species
**AM-S-W1601**	0.235	0.00892	0.0273	0.178 - 0.292	Accepted	---
**AM-S-W1602**	0.422	0.0134	0.0446	0.329 - 0.515	Accepted	---
**AM-S-W1603**	0.132	0.00384	0.0177	0.0957 - 0.168	Accepted	---

Statistics

Serum PCB IUPAC # 105 (µg/L)

All methods	AM-S-W1601	AM-S-W1602	AM-S-W1603
N	12	12	12
Robust mean Algo A	0.235	0.422	0.132
Robust STDev	0.0247	0.0370	0.0106
Median	0.236	0.415	0.136
STDev from MAD	0.0250	0.0400	0.00964
Arithmetic mean	0.236	0.429	0.127
STDev	0.0285	0.0513	0.0183
Comparison AM-S-W1505	0.247		
	0.0315		
CV or Variability	10.5%	8.8%	8.1%

GC-MS CI	AM-S-W1601	AM-S-W1602	AM-S-W1603
N	3	3	3
Robust mean Algo A	0.245	0.438	0.135
Robust STDev	0.0109	0.0136	0.00371
Median	0.248	0.441	0.135
STDev from MAD	0.00863	0.0108	0.00294
Arithmetic mean	0.237	0.417	0.126
STDev	0.0249	0.0478	0.0188
CV or Variability	4.4%	3.1%	2.8%

When fewer than 20 results were considered for statistical treatment of all or a sub-sample of results, the accuracy of statistical data may be questionable.

Distribution

Serum PCB IUPAC # 105 (µg/L)

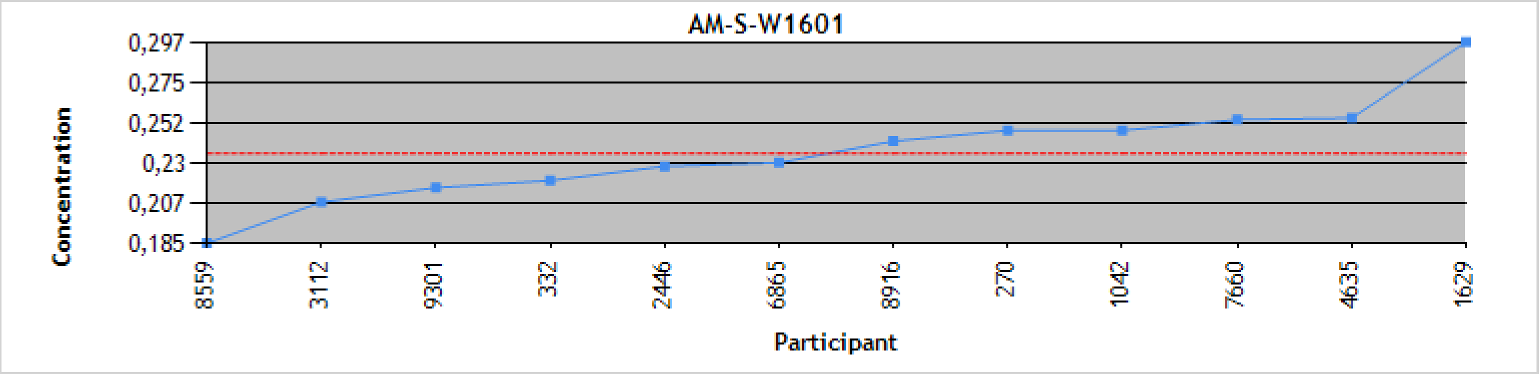

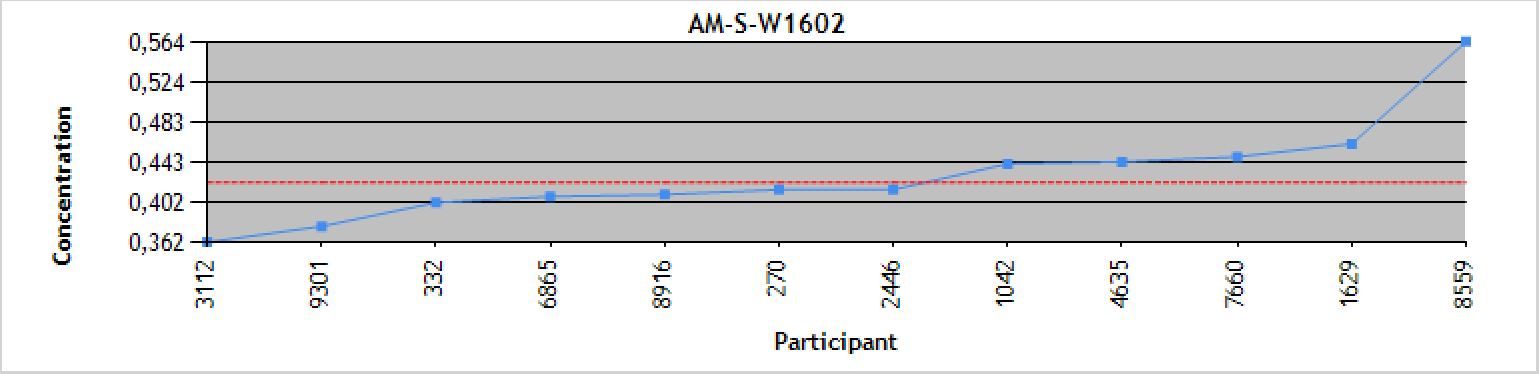

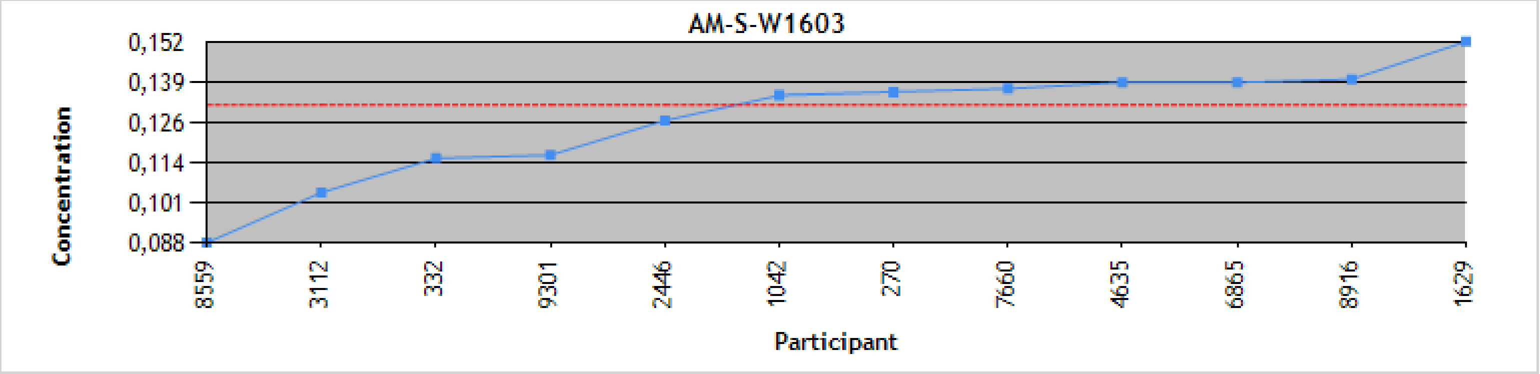

Individual results

Serum PCB IUPAC # 118 (µg/L)

Round #2016-01

Participant	AM-S-W1601	z'-score	AM-S-W1602	z'-score	AM-S-W1603	z'-score	Method
**270**	0.168	1.23	0.339	0.60	0.749	-0.01	ND
**332**	0.124	-0.77	0.292	-0.49	0.708	-0.40	ND
**748**	0.189	2.18	0.384	1.64	0.752	0.02	GC
**1003**	0.138	-0.14	0.324	0.25	0.823	0.70	GC-MS-MS
**1042**	0.137	-0.18	0.308	-0.12	0.777	0.26	GC-MS CI
**1629**	0.151	0.44	0.314	0.02	0.826	0.72	GC-MS-MS
**2446**	0.131	-0.45	0.278	-0.81	0.680	-0.67	ND
**3112**	0.101	-1.82	0.232	-1.87	0.562	-1.79	GC-MS CI
**4635**	0.142	0.05	0.317	0.09	0.784	0.32	GC-MS EI
**5039**	0.311	7.71	0.323	0.23	0.617	-1.27	GC-MS-MS
**6865**	0.161	0.91	0.335	0.51	0.893	1.36	ND
**7660**	0.140	-0.04	0.311	-0.05	0.766	0.16	GC-MS CI
**8559**	0.109	-1.45	0.322	0.21	0.801	0.49	ND
**9301**	0.113	-1.27	0.256	-1.32	0.658	-0.88	GC-MS-MS
**9756**	0.152	0.50	0.309	-0.09	0.775	0.24	GC-MS-MS

	Assigned value	Standard uncertainty	σ pt	Acceptable range	K-S (Lilliefors)	Species
**AM-S-W1601**	0.141	0.00836	0.0204	0.0969 - 0.185	Rejected[1]	---
**AM-S-W1602**	0.313	0.00610	0.0429	0.226 - 0.400	Accepted	---
**AM-S-W1603**	0.750	0.0260	0.102	0.540 - 0.960	Accepted	---

Statistics
Serum PCB IUPAC # 118 (µg/L)

All methods	AM-S-W1601	AM-S-W1602	AM-S-W1603
N	15	15	15
Robust mean Algo A	0.141	0.313	0.750
Robust STDev	0.0259	0.0189	0.0804
Median	0.140	0.314	0.766
STDev from MAD	0.0237	0.0153	0.0841
Arithmetic mean	0.151	0.310	0.745
STDev	0.0498	0.0357	0.0864
Comparison AM-S-W1505	0.143		
	0.0247		
CV or Variability	18.3%	6.0%	10.7%

GC-MS CI	AM-S-W1601	AM-S-W1602	AM-S-W1603
N	3	3	3
Robust mean Algo A	0.136	0.307	0.762
Robust STDev	0.00571	0.00543	0.0209
Median	0.137	0.308	0.766
STDev from MAD	0.00452	0.00430	0.0166
Arithmetic mean	0.126	0.284	0.702
STDev	0.0217	0.0447	0.121
CV or Variability	4.2%	1.8%	2.7%

GC-MS-MS	AM-S-W1601	AM-S-W1602	AM-S-W1603
N	5	5	5
Robust mean Algo A	0.148	0.313	0.750
Robust STDev	0.0236	0.0144	0.0935
Median	0.151	0.314	0.775
STDev from MAD	0.0188	0.0139	0.0753
Arithmetic mean	0.173	0.305	0.740
STDev	0.0786	0.0282	0.0965
CV or Variability	15.9%	4.6%	12.5%

When fewer than 20 results were considered for statistical treatment of all or a sub-sample of results, the accuracy of statistical data may be questionable.

Distribution

Serum PCB IUPAC # 118 (µg/L)

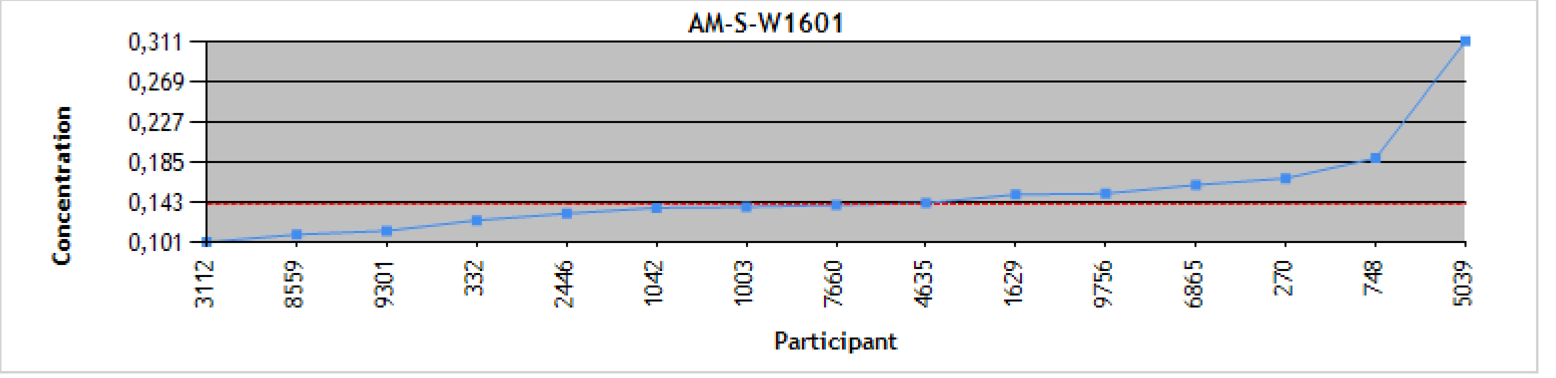

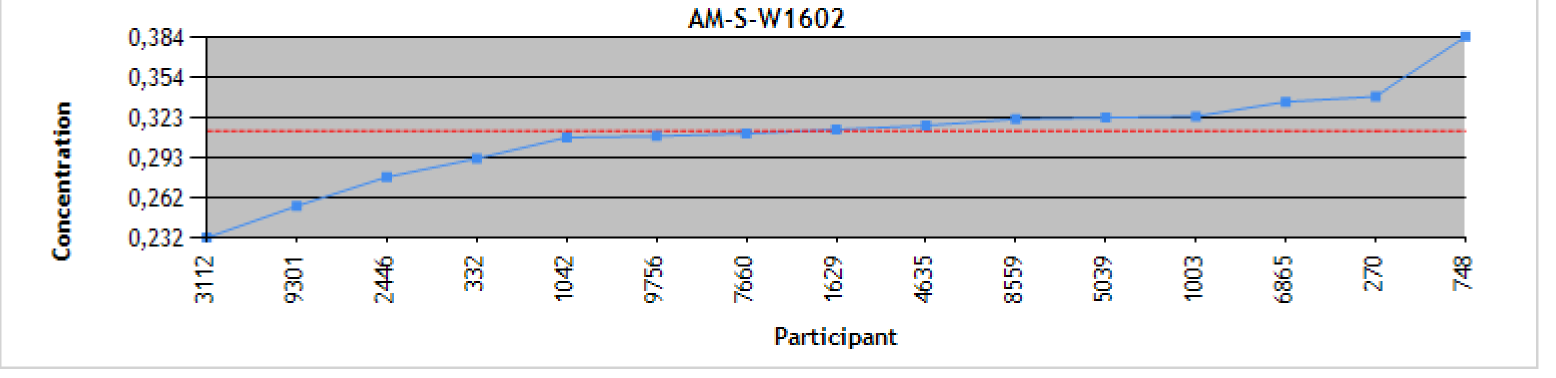

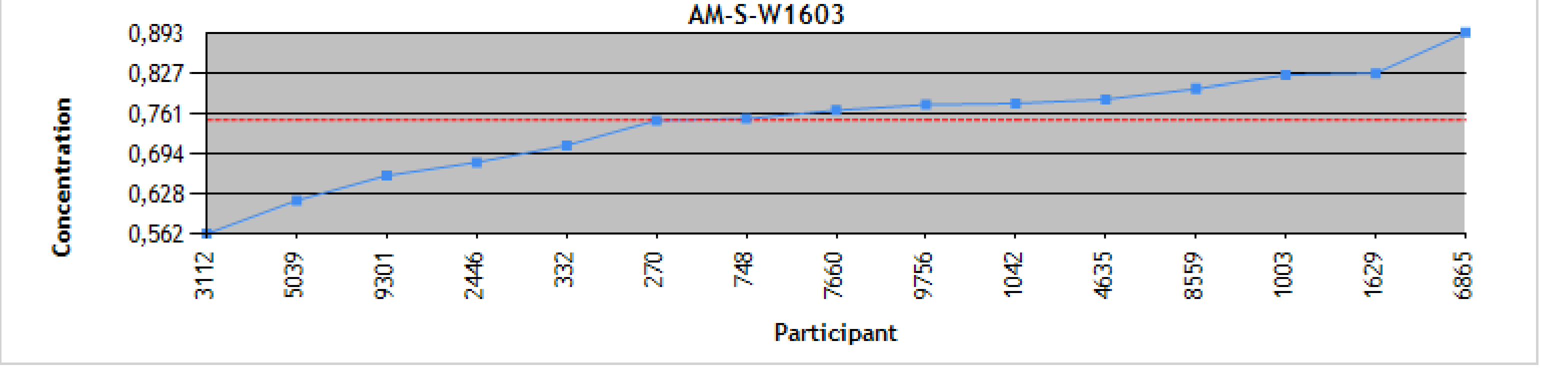

Individual results

Serum PCB IUPAC # 138 (µg/L)

Round #2016-01

Participant	AM-S-W1601	z'-score	AM-S-W1602	z'-score	AM-S-W1603	z'-score	Method
**270**	0.580	-0.84	1.66	-0.84	0.219	-0.28	ND
**332**	0.891	1.80	2.46	1.76	0.289	1.21	ND
**748**	0.819	1.19	1.97	0.16	1.32	**23.02**	GC
**1003**	0.734	0.47	2.14	0.72	0.246	0.30	GC-MS-MS
**1042**	0.612	-0.57	1.88	-0.12	0.199	-0.69	GC-MS CI
**1629**	0.610	-0.59	1.79	-0.43	0.213	-0.41	GC-MS-MS
**2446**	0.579	-0.85	1.76	-0.51	0.182	-1.06	ND
**3112**	0.576	-0.88	1.70	-0.73	0.187	-0.95	GC-MS CI
**5039**	0.399	**-2.38**	1.05	**-2.85**	0.185	-0.99	GC-MS-MS
**6865**	0.672	-0.06	1.71	-0.68	0.226	-0.13	ND
**7660**	0.626	-0.45	1.83	-0.29	0.200	-0.67	GC-MS CI
**8559**	0.784	0.89	2.45	1.73	0.310	1.65	ND
**8916**	0.734	0.47	1.92	-0.01	0.261	0.61	GC
**9301**	0.698	0.16	2.16	0.78	0.217	-0.32	GC-MS-MS
**9756**	0.807	1.09	2.20	0.91	0.269	0.78	GC-MS-MS

	Assigned value	Standard uncertainty	σ pt	Acceptable range	K-S (Lilliefors)	Species
**AM-S-W1601**	0.679	0.0397	0.111	0.444 - 0.914	Accepted	---
**AM-S-W1602**	1.92	0.0908	0.293	1.31 - 2.53	Accepted	---
**AM-S-W1603**	0.232	0.0145	0.0449	0.138 - 0.326	Rejected[1]	---

PCB IUPAC # 163 was added to all PTM with the following levels:
AM-S-W1601 = 0.23 µg/L
AM-S-W1602 = 0.66 µg/L
AM-S-W1603 = 0.072 µg/L

Statistics
Serum PCB IUPAC # 138 (µg/L)

All methods	AM-S-W1601	AM-S-W1602	AM-S-W1603
N	15	15	15
Robust mean Algo A	0.679	1.92	0.232
Robust STDev	0.123	0.281	0.0450
Median	0.672	1.88	0.219
STDev from MAD	0.136	0.276	0.0475
Arithmetic mean	0.675	1.91	0.302
STDev	0.125	0.352	0.284
Comparison AM-S-W1505	0.709		
	0.139		
CV or Variability	18.1%	14.7%	19.4%

GC-MS CI	AM-S-W1601	AM-S-W1602	AM-S-W1603
N	3	3	3
Robust mean Algo A	0.606	1.81	0.199
Robust STDev	0.0274	0.0986	0.00174
Median	0.612	1.83	0.199
STDev from MAD	0.0217	0.0781	0.00138
Arithmetic mean	0.605	1.80	0.196
STDev	0.0258	0.0960	0.00740
CV or Variability	4.5%	5.5%	0.9%

GC-MS-MS	AM-S-W1601	AM-S-W1602	AM-S-W1603
N	5	5	5
Robust mean Algo A	0.670	2.10	0.226
Robust STDev	0.134	0.102	0.0366
Median	0.698	2.14	0.217
STDev from MAD	0.131	0.0890	0.0430
Arithmetic mean	0.650	1.87	0.226
STDev	0.157	0.488	0.0323
CV or Variability	20.0%	4.9%	16.2%

When fewer than 20 results were considered for statistical treatment of all or a sub-sample of results, the accuracy of statistical data may be questionable.

Distribution

Serum PCB IUPAC # 138 (µg/L)

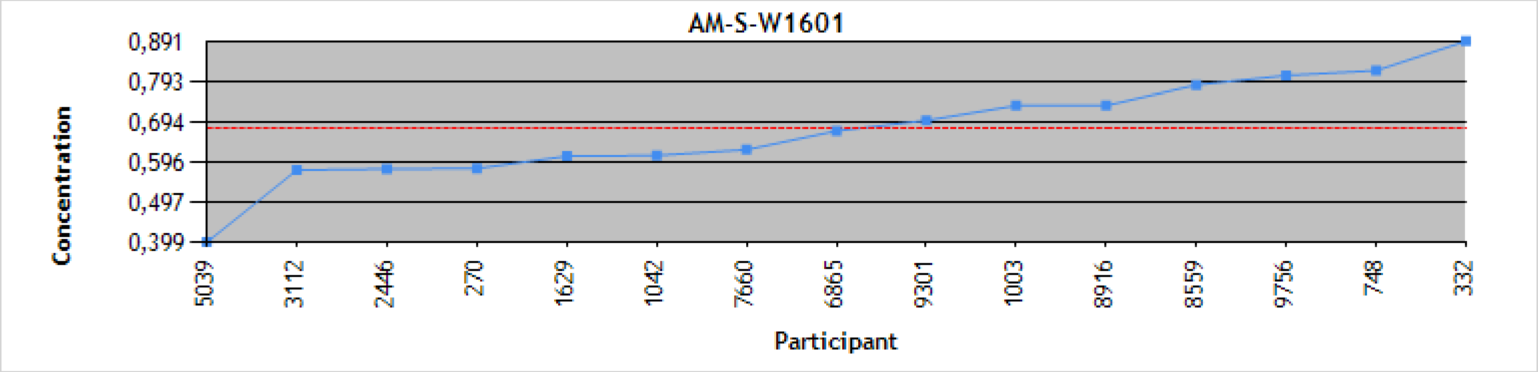

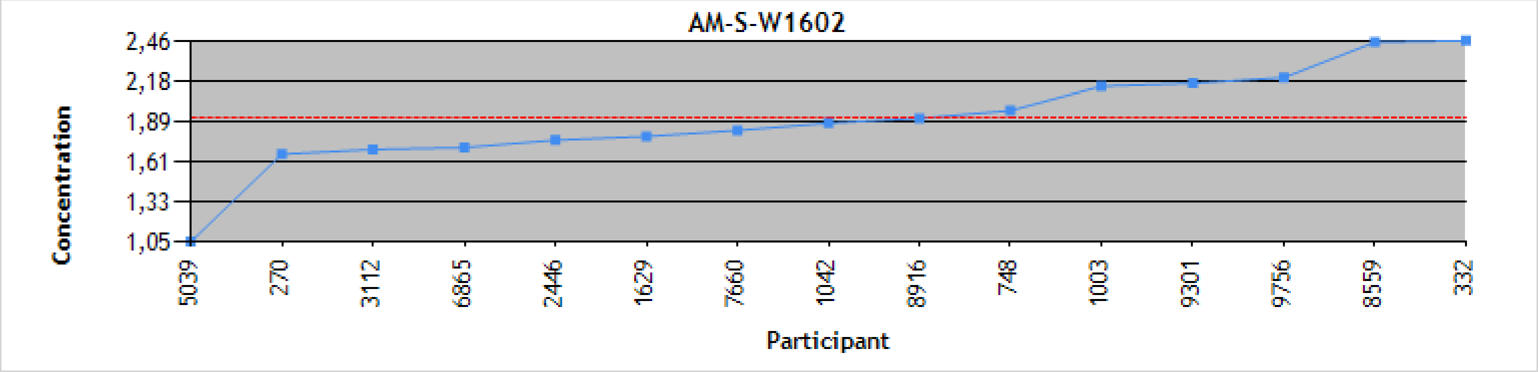

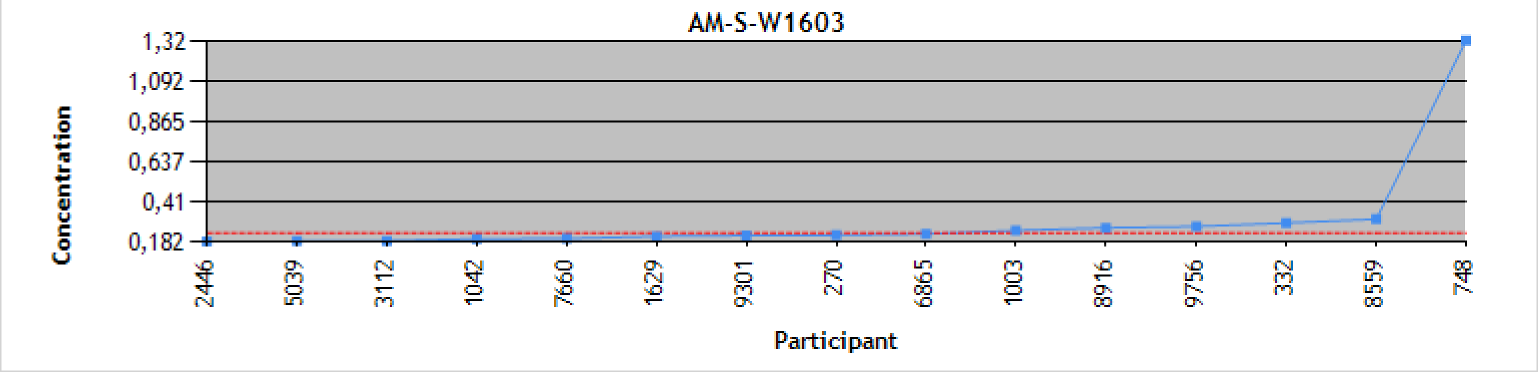

Individual results

Serum PCB IUPAC # 153 (µg/L)

Round #2016-01

Participant	AM-S-W1601	z'-score	AM-S-W1602	z'-score	AM-S-W1603	z'-score	Method
**270**	2.26	**2.26**	1.08	1.89	1.18	1.97	ND
**332**	1.64	-0.24	0.835	-0.09	0.878	-0.28	ND
**748**	1.50	-0.80	0.829	-0.14	0.836	-0.60	GC
**1003**	1.42	-1.12	0.820	-0.21	0.885	-0.23	GC-MS-MS
**1042**	1.83	0.54	0.892	0.37	0.956	0.30	GC-MS CI
**1629**	1.83	0.53	0.867	0.17	0.905	-0.08	GC-MS-MS
**2446**	1.69	-0.06	0.770	-0.61	0.912	-0.03	ND
**3112**	1.56	-0.58	0.711	-1.09	0.776	-1.04	GC-MS CI
**4635**	1.91	0.84	0.921	0.60	1.00	0.65	GC-MS EI
**5039**	1.67	-0.12	0.912	0.53	0.965	0.36	GC-MS-MS
**6865**	1.74	0.16	0.818	-0.23	0.931	0.11	ND
**7660**	1.74	0.16	0.839	-0.05	0.900	-0.12	GC-MS CI
**8559**	1.66	-0.16	0.899	0.43	1.01	0.70	ND
**8916**	1.27	-1.74	0.653	-1.56	0.684	-1.73	GC
**9301**	1.66	-0.16	0.764	-0.66	0.836	-0.60	GC-MS-MS
**9756**	2.73	**4.14**	1.32	**3.82**	1.40	**3.61**	GC-MS-MS

	Assigned value	Standard uncertainty	σ pt	Acceptable range	K-S (Lilliefors)	Species
**AM-S-W1601**	1.70	0.0642	0.241	1.20 - 2.20	Rejected[1]	---
**AM-S-W1602**	0.846	0.0295	0.121	0.598 - 1.09	Rejected[1]	---
**AM-S-W1603**	0.916	0.0310	0.130	0.648 - 1.18	Accepted	---

PCB IUPAC # 163 was added to all PTM with the following levels:

AM-S-W1601 = 0.69 µg/L
AM-S-W1602 = 0.28 µg/L
AM-S-W1603 = 0.34 µg/L

Statistics

Serum PCB IUPAC # 153 (µg/L)

All methods	AM-S-W1601	AM-S-W1602	AM-S-W1603
N	16	16	16
Robust mean Algo A	1.70	0.846	0.916
Robust STDev	0.205	0.0945	0.0992
Median	1.68	0.837	0.908
STDev from MAD	0.206	0.0957	0.0955
Arithmetic mean	1.76	0.871	0.941
STDev	0.341	0.154	0.164
Comparison AM-S-W1505	1.71		
	0.105		
CV or Variability	12.1%	11.2%	10.8%

GC-MS CI	AM-S-W1601	AM-S-W1602	AM-S-W1603
N	3	3	3
Robust mean Algo A	1.71	0.818	0.877
Robust STDev	0.161	0.0979	0.105
Median	1.74	0.839	0.900
STDev from MAD	0.137	0.0776	0.0836
Arithmetic mean	1.71	0.814	0.877
STDev	0.142	0.0929	0.0922
CV or Variability	9.4%	12.0%	11.9%

GC-MS-MS	AM-S-W1601	AM-S-W1602	AM-S-W1603
N	5	5	5
Robust mean Algo A	1.72	0.867	0.926
Robust STDev	0.258	0.0906	0.0884
Median	1.67	0.867	0.905
STDev from MAD	0.242	0.0691	0.0888
Arithmetic mean	1.86	0.937	0.998
STDev	0.507	0.221	0.229
CV or Variability	15.0%	10.5%	9.5%

When fewer than 20 results were considered for statistical treatment of all or a sub-sample of results, the accuracy of statistical data may be questionable.

Distribution

Serum PCB IUPAC # 153 (µg/L)

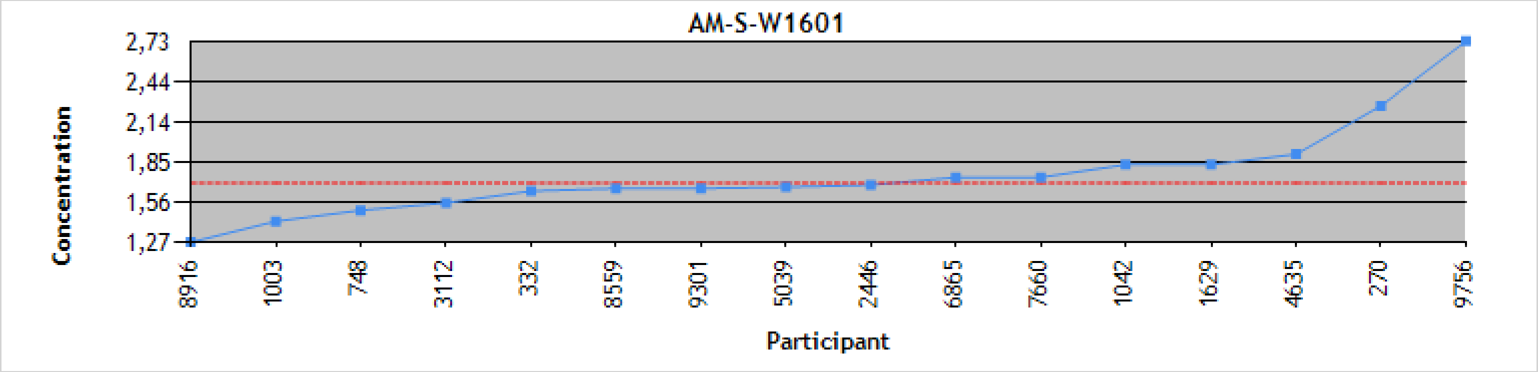

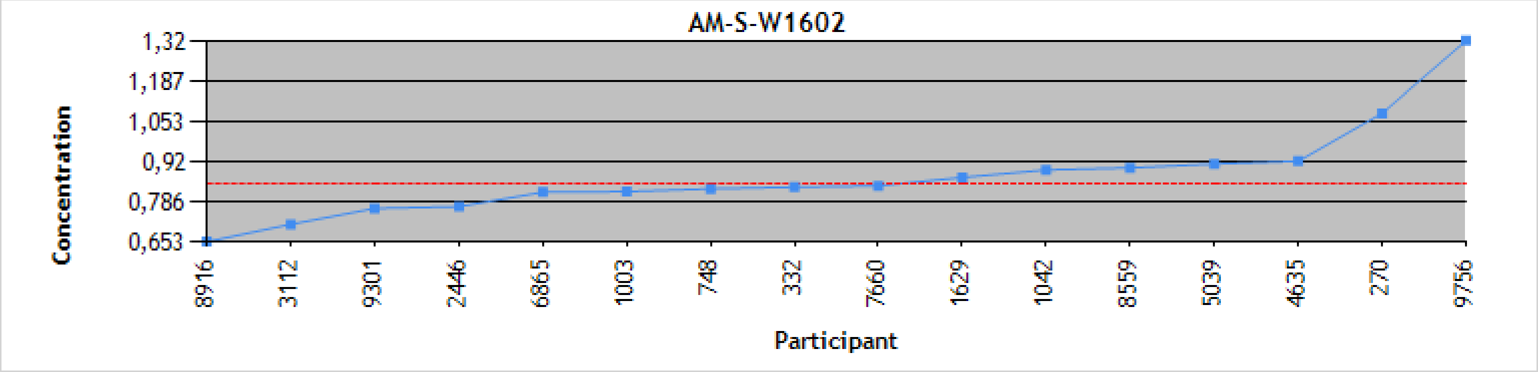

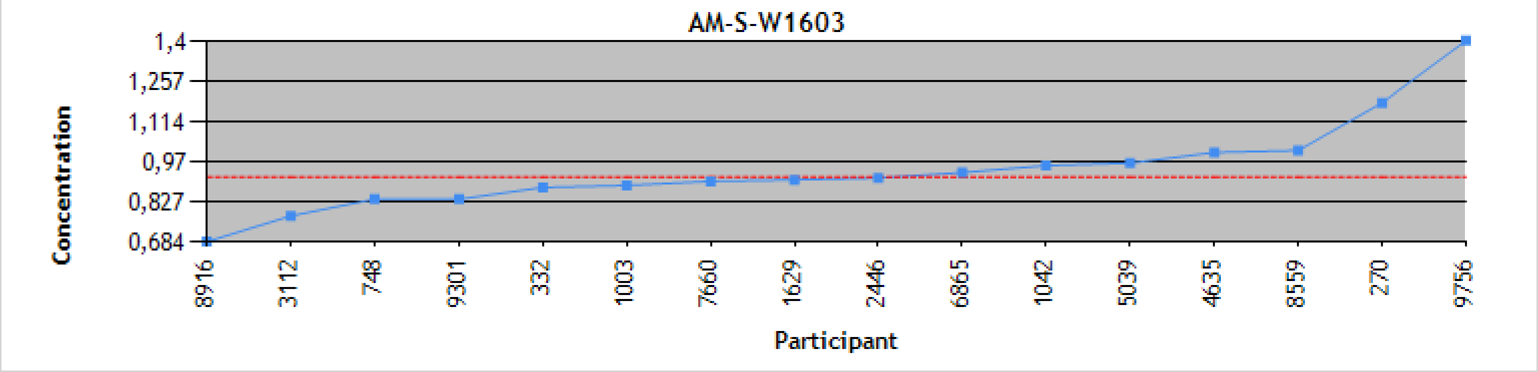

Individual results

Serum PCB IUPAC # 170 (µg/L)

Round #2016-01

Participant	AM-S-W1601	z'-score	AM-S-W1602	z'-score	AM-S-W1603	z'-score	Method
332	0.767	-0.02	0.257	-0.12	0.653	0.01	ND
1003	0.831	0.55	0.272	0.24	0.674	0.23	GC-MS-MS
1042	0.785	0.14	0.263	0.03	0.664	0.13	GC-MS CI
1629	0.829	0.53	0.274	0.29	0.717	0.67	GC-MS-MS
3112	0.756	-0.12	0.244	-0.43	0.615	-0.38	GC-MS CI
4635	0.816	0.42	0.271	0.22	0.657	0.05	GC-MS EI
6865	0.712	-0.51	0.225	-0.89	0.591	-0.63	ND
7660	0.795	0.23	0.266	0.09	0.650	-0.03	GC-MS CI
8559	0.711	-0.52	0.317	1.32	0.731	0.82	ND
8916	0.681	-0.78	0.246	-0.38	0.581	-0.74	GC
9301	0.650	-1.06	0.198	-1.53	0.521	-1.36	GC-MS-MS
9756	0.931	1.44	0.271	0.22	0.736	0.87	GC-MS-MS

	Assigned value	Standard uncertainty	σ pt	Acceptable range	K-S (Lilliefors)	Species
AM-S-W1601	0.769	0.0280	0.109	0.544 - 0.994	Accepted	---
AM-S-W1602	0.262	0.00547	0.0414	0.178 - 0.346	Accepted	---
AM-S-W1603	0.652	0.0242	0.0934	0.459 - 0.845	Accepted	---

Statistics
Serum PCB IUPAC # 170 (µg/L)

All methods	AM-S-W1601	AM-S-W1602	AM-S-W1603
N	12	12	12
Robust mean Algo A	0.769	0.262	0.652
Robust STDev	0.0775	0.0152	0.0671
Median	0.776	0.265	0.655
STDev from MAD	0.0801	0.0126	0.0757
Arithmetic mean	0.772	0.259	0.649
STDev	0.0771	0.0292	0.0642
Comparison AM-S-W1505	0.783		
	0.0511		
CV or Variability	10.1%	5.8%	10.3%

GC-MS CI	AM-S-W1601	AM-S-W1602	AM-S-W1603
N	3	3	3
Robust mean Algo A	0.781	0.262	0.643
Robust STDev	0.0187	0.00500	0.0278
Median	0.785	0.263	0.650
STDev from MAD	0.0148	0.00396	0.0220
Arithmetic mean	0.778	0.258	0.643
STDev	0.0201	0.0119	0.0253
CV or Variability	2.4%	1.9%	4.3%

GC-MS-MS	AM-S-W1601	AM-S-W1602	AM-S-W1603
N	4	4	4
Robust mean Algo A	0.827	0.271	0.688
Robust STDev	0.0995	0.00276	0.0552
Median	0.830	0.272	0.696
STDev from MAD	0.0758	0.00222	0.0460
Arithmetic mean	0.810	0.254	0.662
STDev	0.117	0.0372	0.0975
CV or Variability	12.0%	1.0%	8.0%

When fewer than 20 results were considered for statistical treatment of all or a sub-sample of results, the accuracy of statistical data may be questionable.

Distribution

Serum PCB IUPAC # 170 (µg/L)

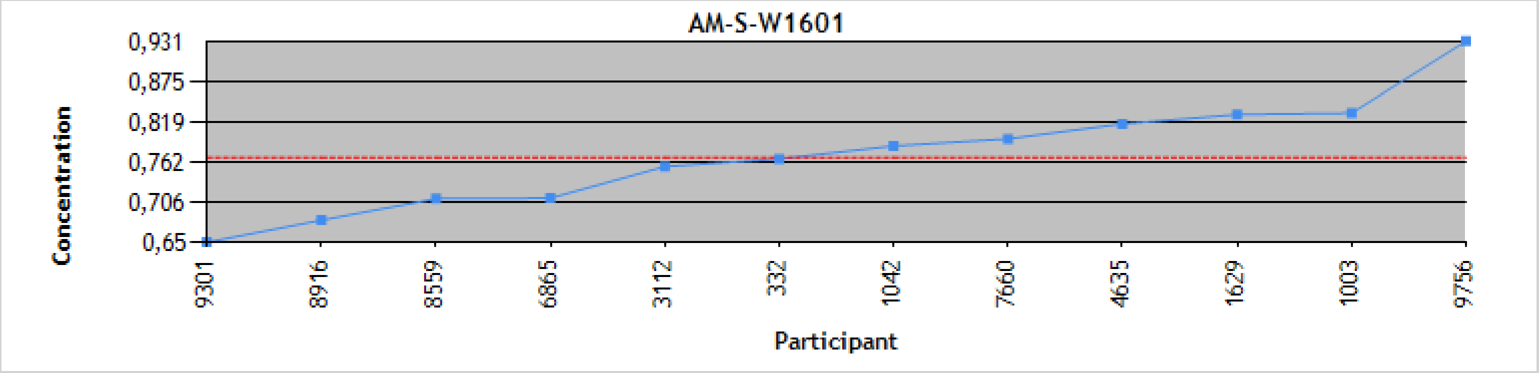

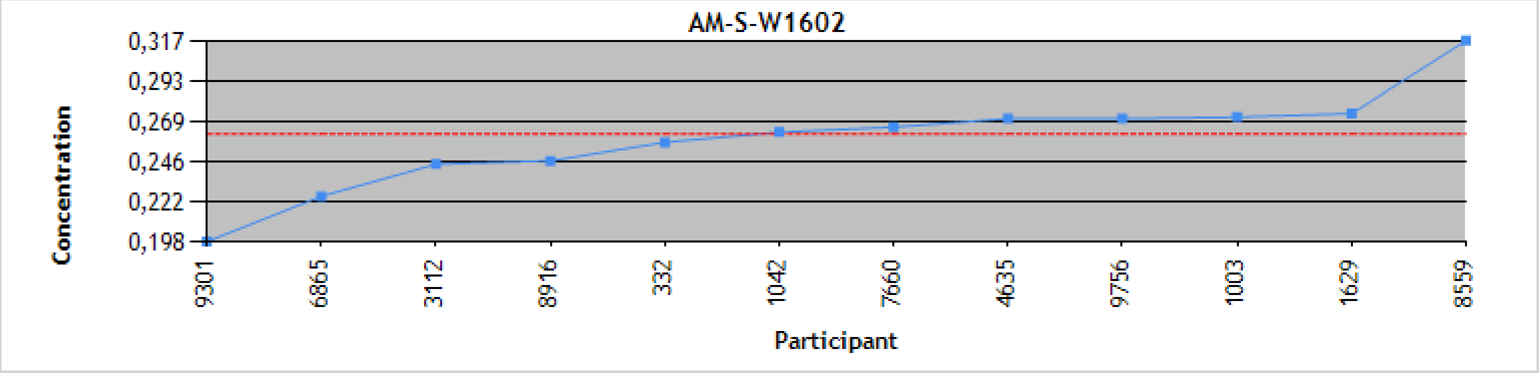

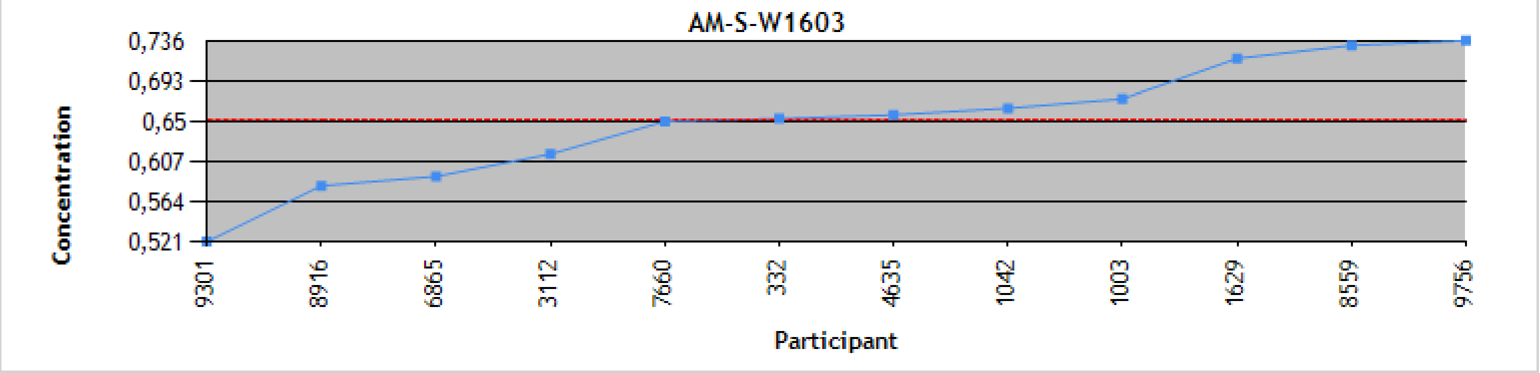

Individual results

Serum PCB IUPAC # 180 (µg/L)

Round #2016-01

Participant	AM-S-W1601	z'-score	AM-S-W1602	z'-score	AM-S-W1603	z'-score	Method
**270**	1.25	0.66	0.808	0.68	1.90	0.48	ND
**332**	1.13	-0.06	0.763	0.27	1.84	0.27	ND
**748**	0.833	-1.81	0.590	-1.29	0.305	**-5.55**	GC
**1003**	1.07	-0.40	0.734	0.01	1.66	-0.42	GC-MS-MS
**1042**	1.19	0.29	0.770	0.33	1.86	0.33	GC-MS CI
**1629**	1.33	1.15	0.855	1.10	2.08	1.18	GC-MS-MS
**2446**	1.22	0.48	0.721	-0.11	1.71	-0.22	ND
**3112**	1.14	0.01	0.717	-0.14	1.81	0.16	GC-MS CI
**4635**	1.11	-0.18	0.714	-0.17	1.71	-0.22	GC-MS EI
**5039**	0.833	-1.81	0.654	-0.71	1.45	-1.23	GC-MS-MS
**6865**	1.09	-0.30	0.690	-0.39	1.68	-0.34	ND
**7660**	1.23	0.51	0.788	0.50	1.90	0.49	GC-MS CI
**8559**	1.10	-0.24	0.813	0.72	2.01	0.91	ND
**8916**	1.10	-0.23	0.554	-1.62	1.59	-0.68	GC
**9301**	1.04	-0.59	0.649	-0.76	1.66	-0.42	GC-MS-MS
**9756**	1.40	1.53	0.897	1.48	2.24	1.78	GC-MS-MS

	Assigned value	Standard uncertainty	σ pt	Acceptable range	K-S (Lilliefors)	Species
**AM-S-W1601**	1.14	0.0363	0.165	0.803 - 1.48	Accepted	---
**AM-S-W1602**	0.733	0.0304	0.106	0.511 - 0.955	Accepted	---
**AM-S-W1603**	1.77	0.0615	0.257	1.24 - 2.30	Accepted	---

Statistics
Serum PCB IUPAC # 180 (µg/L)

All methods	AM-S-W1601	AM-S-W1602	AM-S-W1603
N	16	16	16
Robust mean Algo A	1.14	0.733	1.77
Robust STDev	0.116	0.0973	0.197
Median	1.12	0.728	1.76
STDev from MAD	0.111	0.0993	0.175
Arithmetic mean	1.13	0.732	1.71
STDev	0.151	0.0922	0.424
Comparison AM-S-W1505	1.10		
	0.0717		
CV or Variability	10.2%	13.3%	11.1%

GC-MS CI	AM-S-W1601	AM-S-W1602	AM-S-W1603
N	3	3	3
Robust mean Algo A	1.19	0.762	1.86
Robust STDev	0.0485	0.0347	0.0490
Median	1.19	0.770	1.86
STDev from MAD	0.0545	0.0275	0.0600
Arithmetic mean	1.19	0.758	1.86
STDev	0.0428	0.0369	0.0432
CV or Variability	4.1%	4.5%	2.6%

GC-MS-MS	AM-S-W1601	AM-S-W1602	AM-S-W1603
N	5	5	5
Robust mean Algo A	1.14	0.758	1.80
Robust STDev	0.262	0.129	0.339
Median	1.07	0.734	1.66
STDev from MAD	0.355	0.126	0.317
Arithmetic mean	1.14	0.758	1.82
STDev	0.231	0.114	0.330
CV or Variability	23.1%	17.1%	18.9%

When fewer than 20 results were considered for statistical treatment of all or a sub-sample of results, the accuracy of statistical data may be questionable.

Distribution
Serum PCB IUPAC # 180 (µg/L)

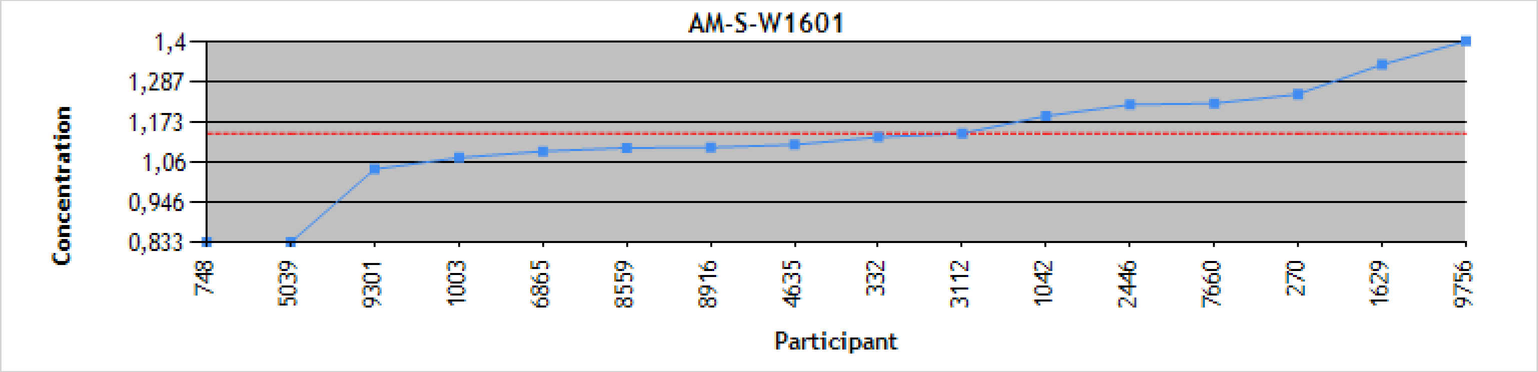

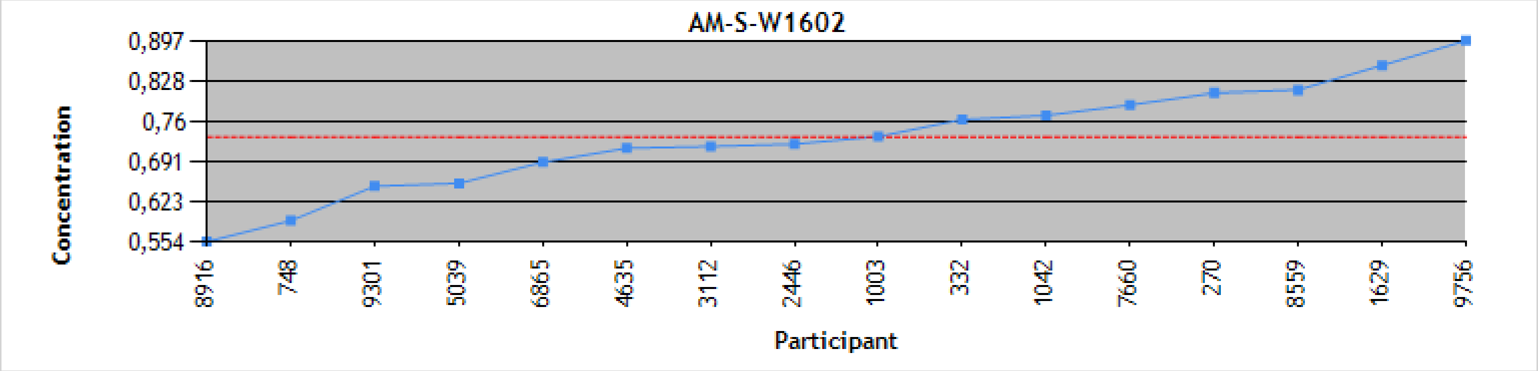

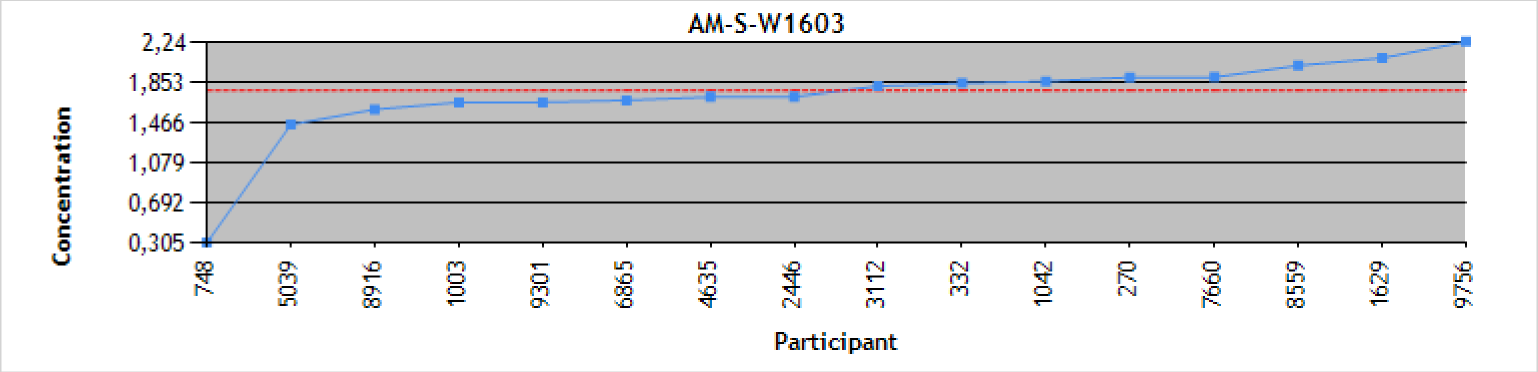

Individual results
Serum PCB IUPAC # 183 (µg/L)
Round #2016-01

Participant	AM-S-W1601	z'-score	AM-S-W1602	z'-score	AM-S-W1603	z'-score	Method
270	<LD	---	0.175	0.20	0.699	-0.61	ND
332	<LQ	---	0.152	-0.46	0.776	-0.15	ND
1003	0.00500	-0.14	0.180	0.35	0.893	0.56	GC-MS-MS
1042	0.00654	0.01	0.173	0.15	0.828	0.17	GC-MS CI
1629	0.00750	0.10	0.187	0.53	0.883	0.50	GC-MS-MS
6865	<LQ	---	0.142	-0.75	0.707	-0.56	ND
7660	0.00677	0.03	0.171	0.10	0.824	0.14	GC-MS CI
8559	<LD	---	0.189	0.61	0.905	0.64	ND
9301	<LD	---	0.139	-0.84	0.685	-0.70	GC-MS-MS

	Assigned value	Standard uncertainty	σ pt	Acceptable range	K-S (Lilliefors)	Species
AM-S-W1601	0.00645	0.000526	0.00129	0.00366 - 0.00924	Accepted	---
AM-S-W1602	0.168	0.00833	0.0336	0.0987 - 0.237	Accepted	---
AM-S-W1603	0.800	0.0411	0.160	0.470 - 1.13	Accepted	---

PCB IUPAC # 183 is not included in the scope of our accreditation.

Statistics

Serum PCB IUPAC # 183 (µg/L)

All methods	AM-S-W1601	AM-S-W1602	AM-S-W1603
N	4	9	9
Robust mean Algo A	0.00660	0.168	0.800
Robust STDev	0.000895	0.0200	0.0987
Median	0.00666	0.173	0.824
STDev from MAD	0.000712	0.0199	0.103
Arithmetic mean	0.00645	0.168	0.800
STDev	0.00105	0.0187	0.0870
CV or Variability	16.3%	11.9%	12.3%

GC-MS-MS	AM-S-W1601	AM-S-W1602	AM-S-W1603
N	NA	3	3
Robust mean Algo A	NA	0.177	0.879
Robust STDev	NA	0.0122	0.0187
Median	NA	0.180	0.883
STDev from MAD	NA	0.00964	0.0148
Arithmetic mean	NA	0.169	0.820
STDev	NA	0.0258	0.117
CV or Variability	NA	6.9%	2.1%

When fewer than 20 results were considered for statistical treatment of all or a sub-sample of results, the accuracy of statistical data may be questionable.

For PCB IUPAC # 183 in material AM-S-W1601: according to the limit number of results, the arithmetic mean have been stated more appropriate for assigned value determination.

Distribution

Serum PCB IUPAC # 183 (µg/L)

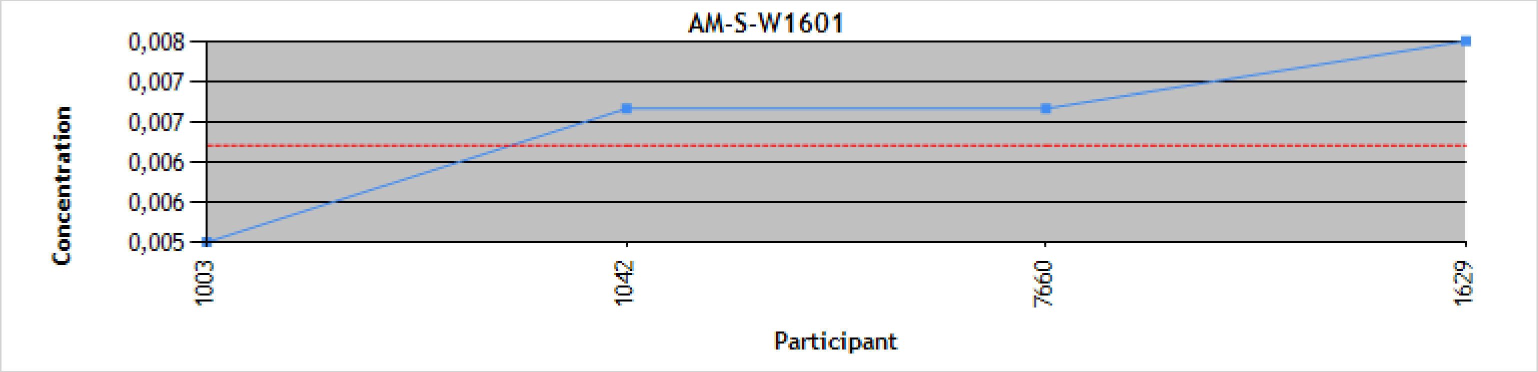

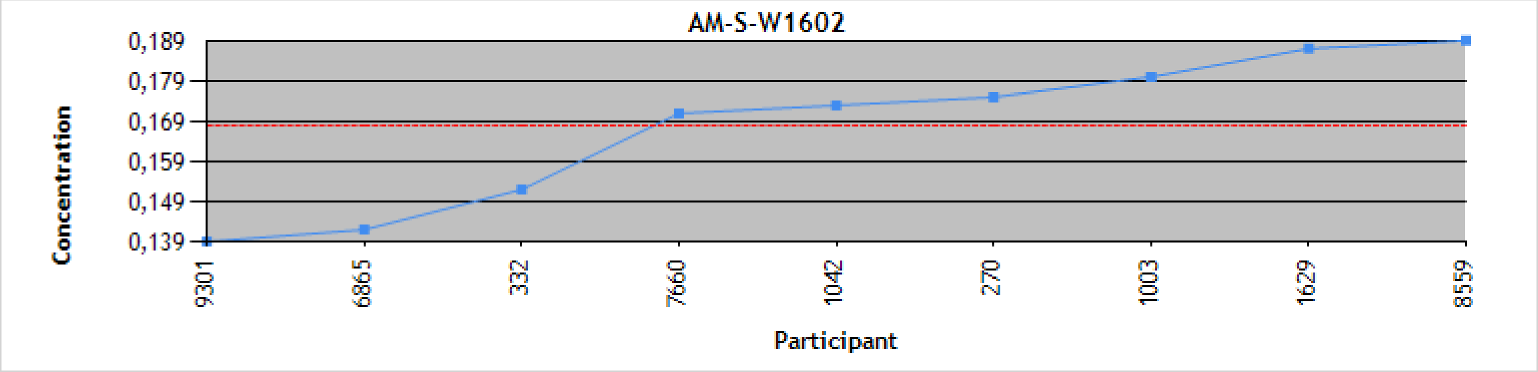

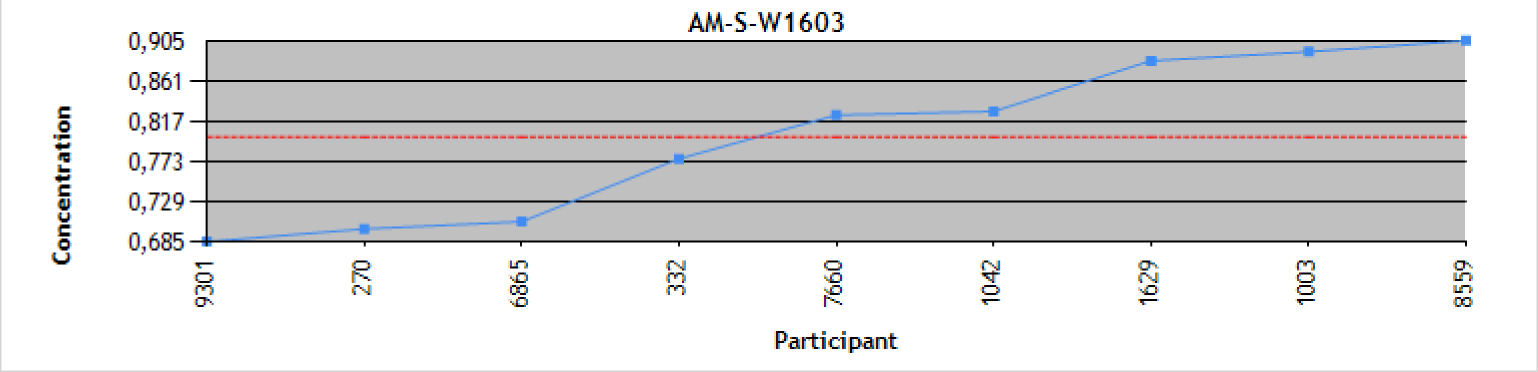

Individual results

Serum PCB IUPAC # 187 (µg/L)

Round #2016-01

Participant	AM-S-W1601	z'-score	AM-S-W1602	z'-score	AM-S-W1603	z'-score	Method
**270**	<LD	---	0.973	0.02	1.69	-0.13	ND
**332**	<LQ	---	0.897	-0.36	1.62	-0.34	ND
**1003**	0.0270	-0.23	1.09	0.61	1.91	0.49	GC-MS-MS
**1042**	0.0287	-0.06	1.04	0.36	1.86	0.32	GC-MS CI
**1629**	0.0372	0.78	1.12	0.77	2.03	0.80	GC-MS-MS
**6865**	0.0320	0.27	0.868	-0.51	1.58	-0.45	ND
**7660**	0.0287	-0.06	0.984	0.08	1.75	0.03	GC-MS CI
**8559**	<LD	---	0.916	-0.27	1.73	-0.03	ND
**9301**	0.0256	-0.36	0.830	-0.70	1.52	-0.62	GC-MS-MS

	Assigned value	Standard uncertainty	σ pt	Acceptable range	K-S (Lilliefors)	Species
**AM-S-W1601**	0.0293	0.00180	0.00586	0.0170 - 0.0416	Accepted	---
**AM-S-W1602**	0.969	0.0475	0.194	0.570 - 1.37	Accepted	---
**AM-S-W1603**	1.74	0.0760	0.348	1.03 - 2.45	Accepted	---

PCB IUPAC # 187 is not included in the scope of our accreditation.

Statistics

Serum PCB IUPAC # 187 (µg/L)

All methods	AM-S-W1601	AM-S-W1602	AM-S-W1603
**N**	6	9	9
**Robust mean Algo A**	0.0293	0.969	1.74
**Robust STDev**	0.00352	0.114	0.182
**Median**	0.0287	0.973	1.73
**STDev from MAD**	0.00349	0.113	0.186
**Arithmetic mean**	0.0299	0.969	1.74
**STDev**	0.00419	0.101	0.164
**CV or Variability**	12.0%	11.8%	10.5%

GC-MS-MS	AM-S-W1601	AM-S-W1602	AM-S-W1603
**N**	3	3	3
**Robust mean Algo A**	0.0276	1.08	1.87
**Robust STDev**	0.00262	0.0599	0.210
**Median**	0.0270	1.09	1.91
**STDev from MAD**	0.00208	0.0475	0.166
**Arithmetic mean**	0.0299	1.01	1.82
**STDev**	0.00633	0.160	0.265
**CV or Variability**	9.5%	5.6%	11.2%

When fewer than 20 results were considered for statistical treatment of all or a sub-sample of results, the accuracy of statistical data may be questionable.

Distribution

Serum PCB IUPAC # 187 (µg/L)

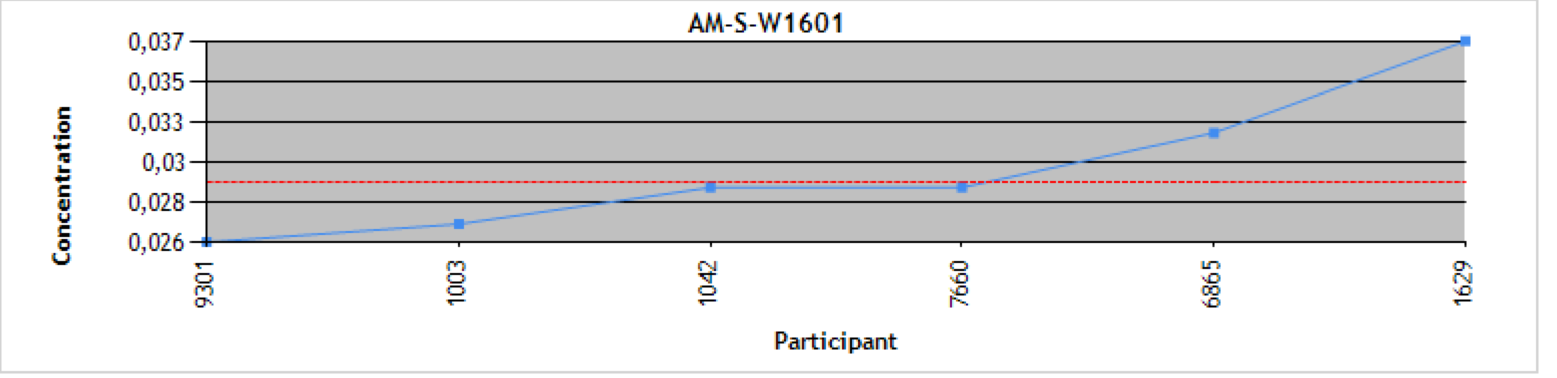

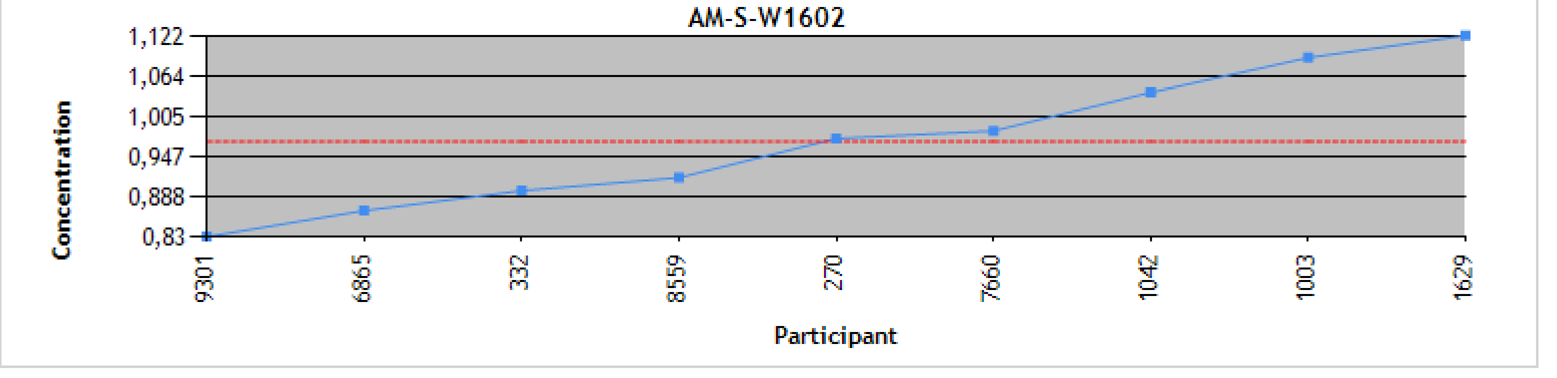

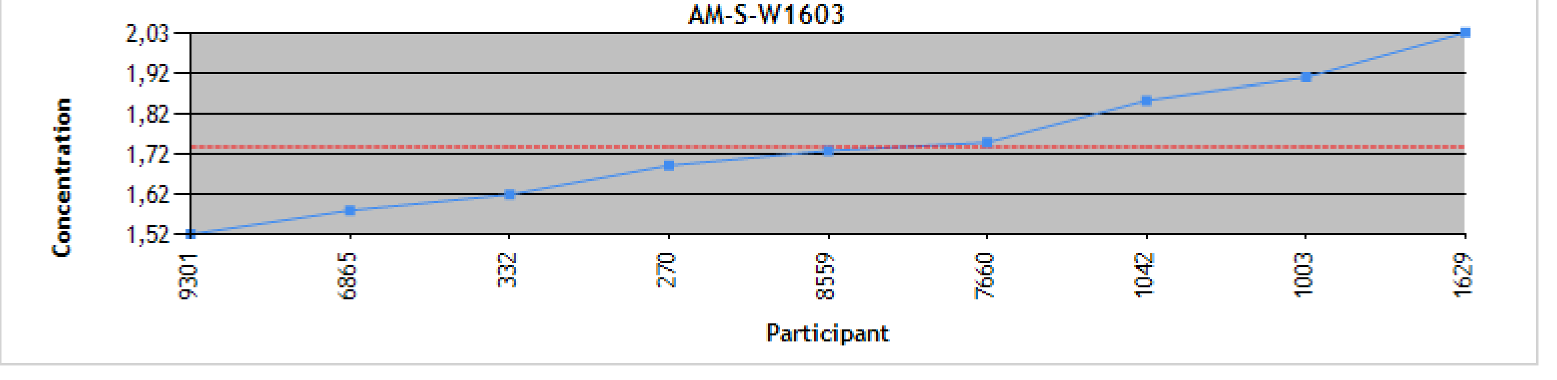

Individual results

Serum PFHxA (µg/L)

Round #2016-01

Participant	AM-S-Y1601	z'-score	AM-S-Y1602	z'-score	AM-S-Y1603	z'-score	Method
**1003**	6.24	-0.51	4.01	-0.79	0.980	-0.31	LC-MS
**1042**	7.33	0.52	4.80	0.30	1.17	0.90	LC-MS
**1382**	6.53	-0.24	3.46	-1.55	1.02	-0.06	LC-MS
**1629**	6.96	0.17	4.93	0.48	0.910	-0.74	ND
**2446**	7.09	0.29	4.60	0.03	0.970	-0.37	ND
**2791**	7.55	0.73	5.22	0.89	1.27	1.48	LC-MS
**3112**	6.17	-0.58	4.05	-0.73	0.975	-0.34	LC-MS
**4087**	5.40	-1.30	3.90	-0.94	0.300	**-4.52**	ND
**5786**	6.69	-0.08	4.51	-0.10	1.08	0.31	ND
**8916**	5.89	-0.84	4.90	0.44	0.950	-0.49	LC-MS
**9450**	7.32	0.51	4.77	0.27	1.10	0.45	LC-MS
**9756**	8.73	1.84	5.72	1.57	1.33	1.84	LC-MS

	Assigned value	Standard uncertainty	σ pt	Acceptable range	K-S (Lilliefors)	Species
**AM-S-Y1601**	6.78	0.295	1.02	4.66 - 8.90	Accepted	---
**AM-S-Y1602**	4.58	0.222	0.688	3.13 - 6.03	Accepted	---
**AM-S-Y1603**	1.03	0.0476	0.155	0.706 - 1.35	Accepted	---

Statistics
Serum PFHxA (µg/L)

All methods	AM-S-Y1601	AM-S-Y1602	AM-S-Y1603
N	12	12	12
Robust mean Algo A	6.78	4.58	1.03
Robust STDev	0.818	0.614	0.132
Median	6.83	4.69	1.00
STDev from MAD	0.809	0.577	0.126
Arithmetic mean	6.82	4.57	1.00
STDev	0.881	0.628	0.258
CV or Variability	12.1%	13.4%	12.8%

LC-MS	AM-S-Y1601	AM-S-Y1602	AM-S-Y1603
N	8	8	8
Robust mean Algo A	6.93	4.62	1.09
Robust STDev	0.967	0.822	0.152
Median	6.92	4.78	1.06
STDev from MAD	0.971	0.866	0.146
Arithmetic mean	6.97	4.62	1.10
STDev	0.943	0.731	0.144
CV or Variability	14.0%	17.8%	13.9%

When fewer than 20 results were considered for statistical treatment of all or a sub-sample of results, the accuracy of statistical data may be questionable.

Distribution

Serum PFHxA (µg/L)

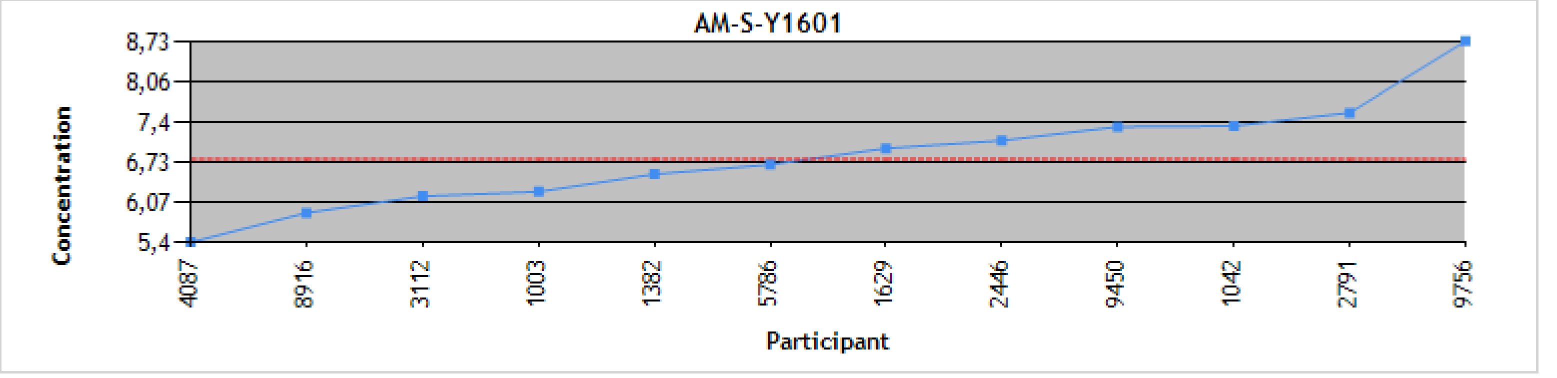

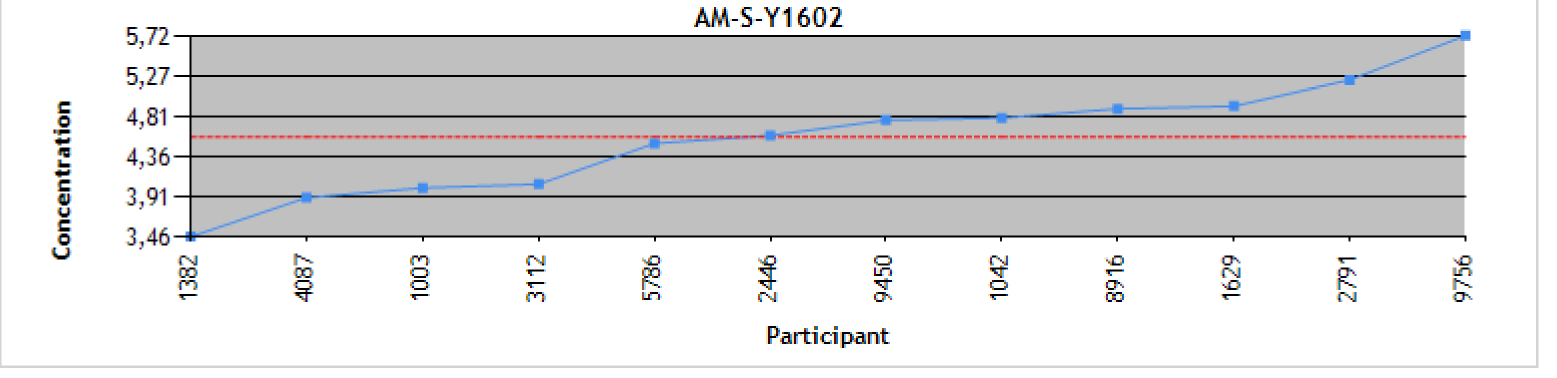

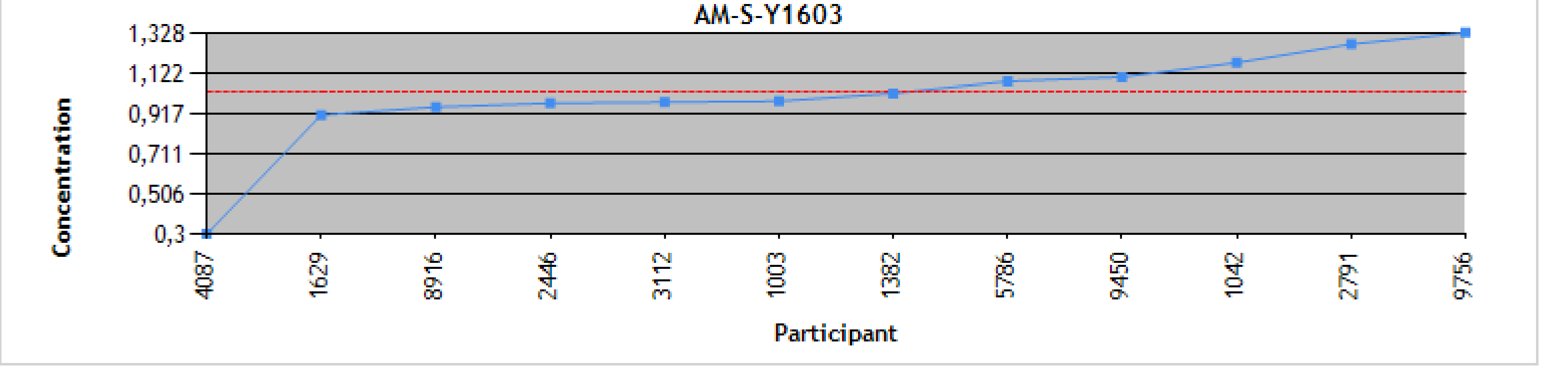

Individual results

Serum PFHxS (µg/L)

Round #2016-01

Participant	AM-S-Y1601	z'-score	AM-S-Y1602	z'-score	AM-S-Y1603	z'-score	Method
**1003**	9.85	-1.19	17.4	-1.36	4.65	-1.07	LC-MS
**1042**	13.5	0.74	23.5	0.44	6.18	0.69	LC-MS
**1382**	11.1	-0.53	15.1	**-2.04**	5.00	-0.67	LC-MS
**1629**	13.8	0.90	24.9	0.86	6.16	0.67	ND
**2446**	12.5	0.21	23.9	0.55	5.55	-0.03	ND
**2791**	12.6	0.26	23.5	0.44	5.94	0.41	LC-MS
**3112**	10.4	-0.92	18.2	-1.11	4.69	-1.02	LC-MS
**4087**	11.2	-0.48	21.7	-0.09	5.70	0.14	ND
**5786**	11.3	-0.42	19.7	-0.68	5.24	-0.39	ND
**8916**	12.8	0.37	22.8	0.24	5.61	0.03	LC-MS
**9301**	14.0	1.00	22.8	0.24	10.8	**5.99**	LC-MS
**9450**	11.5	-0.33	19.8	-0.66	5.11	-0.54	LC-MS
**9756**	13.5	0.72	24.1	0.63	6.10	0.60	LC-MS

	Assigned value	Standard uncertainty	σ pt	Acceptable range	K-S (Lilliefors)	Species
**AM-S-Y1601**	12.1	0.534	1.82	8.30 - 15.9	Accepted	---
**AM-S-Y1602**	22.0	0.769	3.30	15.2 - 28.8	Accepted	---
**AM-S-Y1603**	5.58	0.242	0.837	3.84 - 7.32	Rejected[1]	---

Statistics
Serum PFHxS (µg/L)

All methods	AM-S-Y1601	AM-S-Y1602	AM-S-Y1603
N	13	13	13
Robust mean Algo A	12.1	22.0	5.58
Robust STDev	1.54	2.22	0.698
Median	12.5	22.8	5.61
STDev from MAD	1.76	1.98	0.742
Arithmetic mean	12.1	21.3	5.90
STDev	1.36	3.03	1.56
CV or Variability	12.7%	10.1%	12.5%

LC-MS	AM-S-Y1601	AM-S-Y1602	AM-S-Y1603
N	9	9	9
Robust mean Algo A	12.2	21.8	5.57
Robust STDev	1.64	2.15	0.864
Median	12.6	22.8	5.61
STDev from MAD	1.68	1.98	0.849
Arithmetic mean	12.1	20.8	6.01
STDev	1.49	3.27	1.89
CV or Variability	13.5%	9.9%	15.5%

When fewer than 20 results were considered for statistical treatment of all or a sub-sample of results, the accuracy of statistical data may be questionable.

Distribution

Serum PFHxS (µg/L)

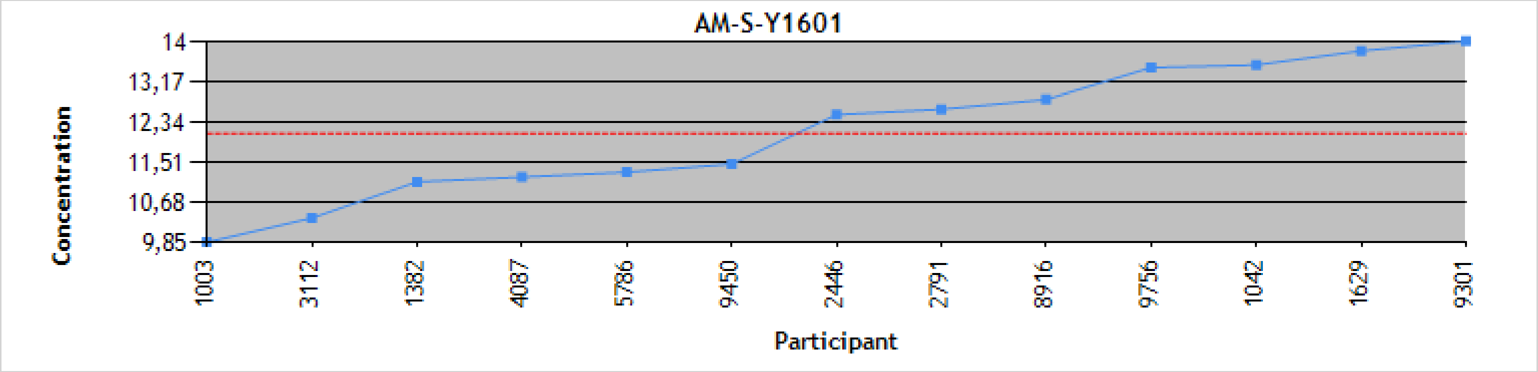

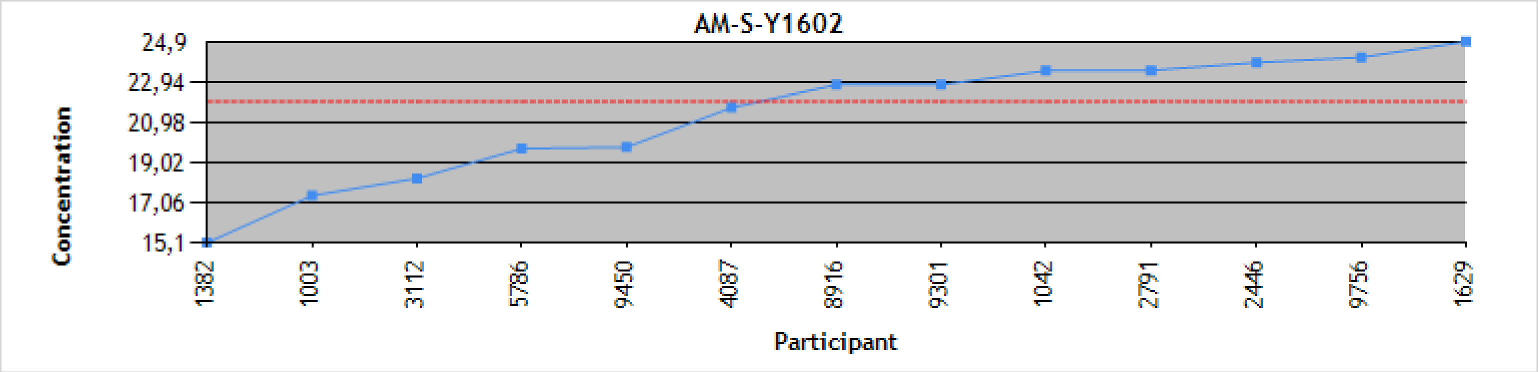

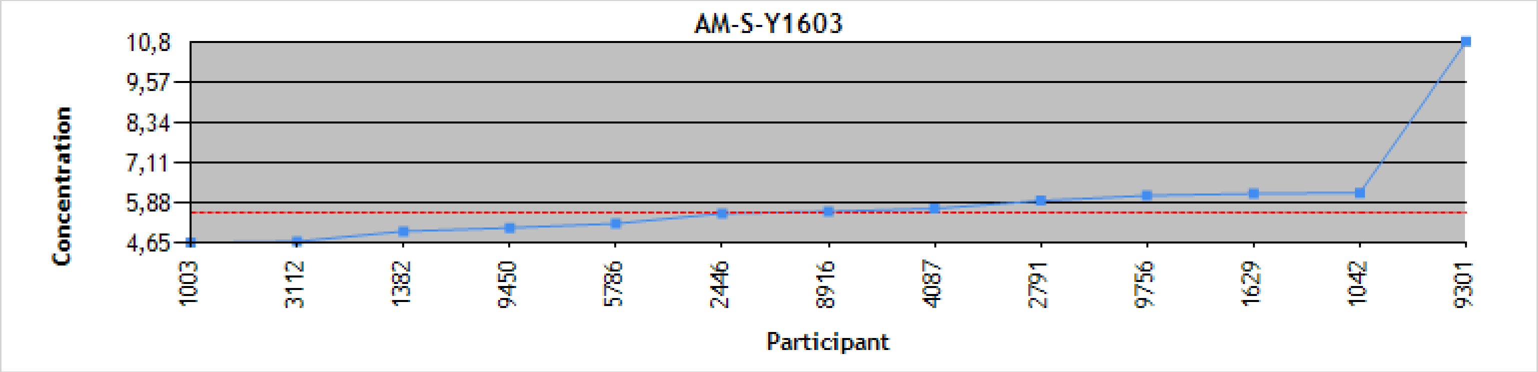

Individual results

Serum PFNA (µg/L)

Round #2016-01

Participant	AM-S-Y1601	z'-score	AM-S-Y1602	z'-score	AM-S-Y1603	z'-score	Method
**1003**	1.40	-1.39	2.68	-0.77	0.720	-0.18	LC-MS
**1042**	2.01	0.78	3.27	0.49	0.849	0.91	LC-MS
**1382**	1.76	-0.11	2.40	-1.36	0.760	0.16	LC-MS
**1629**	2.32	1.89	3.18	0.30	0.700	-0.35	ND
**2446**	1.71	-0.29	3.10	0.13	0.680	-0.52	ND
**2791**	1.95	0.57	3.27	0.49	0.809	0.58	LC-MS
**3112**	1.61	-0.65	2.72	-0.68	0.650	-0.77	LC-MS
**4087**	1.60	-0.68	3.10	0.13	0.800	0.50	ND
**5786**	1.68	-0.39	2.71	-0.70	0.580	-1.37	ND
**8916**	1.98	0.68	3.66	1.32	0.860	1.01	LC-MS
**9301**	1.61	-0.64	2.90	-0.30	0.546	-1.66	LC-MS
**9450**	1.82	0.11	2.99	-0.10	0.770	0.25	LC-MS
**9756**	1.96	0.61	3.36	0.67	0.868	1.08	LC-MS

	Assigned value	Standard uncertainty	σ pt	Acceptable range	K-S (Lilliefors)	Species
**AM-S-Y1601**	1.79	0.0791	0.268	1.23 - 2.35	Accepted	---
**AM-S-Y1602**	3.04	0.112	0.455	2.10 - 3.98	Accepted	---
**AM-S-Y1603**	0.741	0.0386	0.111	0.506 - 0.976	Accepted	---

Statistics
Serum PFNA (µg/L)

All methods	AM-S-Y1601	AM-S-Y1602	AM-S-Y1603
N	13	13	13
Robust mean Algo A	1.79	3.04	0.741
Robust STDev	0.228	0.323	0.111
Median	1.76	3.10	0.760
STDev from MAD	0.237	0.297	0.119
Arithmetic mean	1.80	3.03	0.738
STDev	0.240	0.340	0.104
CV or Variability	12.8%	10.6%	15.0%

LC-MS	AM-S-Y1601	AM-S-Y1602	AM-S-Y1603
N	9	9	9
Robust mean Algo A	1.80	3.02	0.765
Robust STDev	0.222	0.438	0.106
Median	1.82	2.99	0.770
STDev from MAD	0.234	0.413	0.117
Arithmetic mean	1.79	3.03	0.759
STDev	0.212	0.395	0.107
CV or Variability	12.4%	14.5%	13.9%

When fewer than 20 results were considered for statistical treatment of all or a sub-sample of results, the accuracy of statistical data may be questionable.

Distribution

Serum PFNA (µg/L)

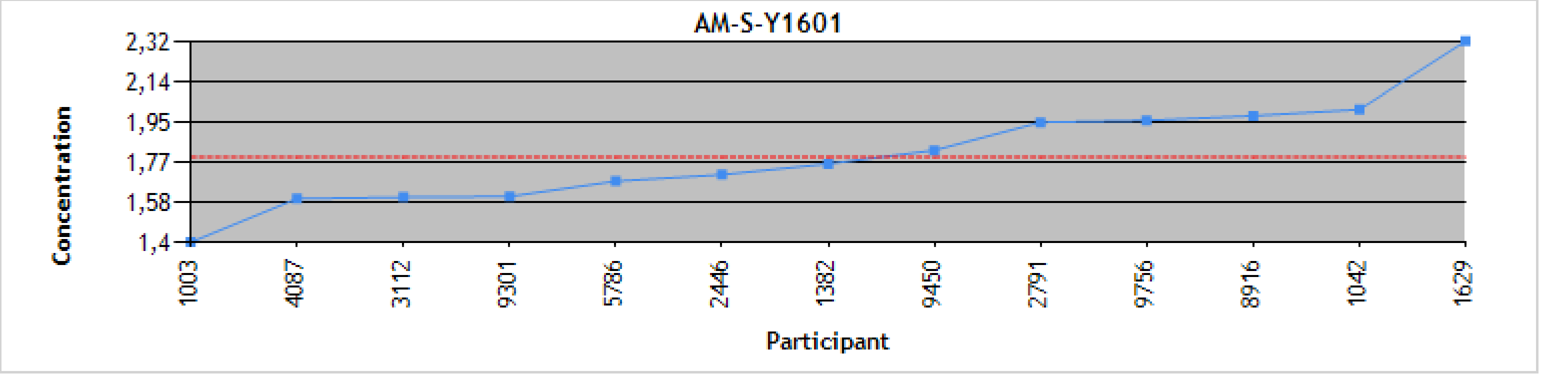

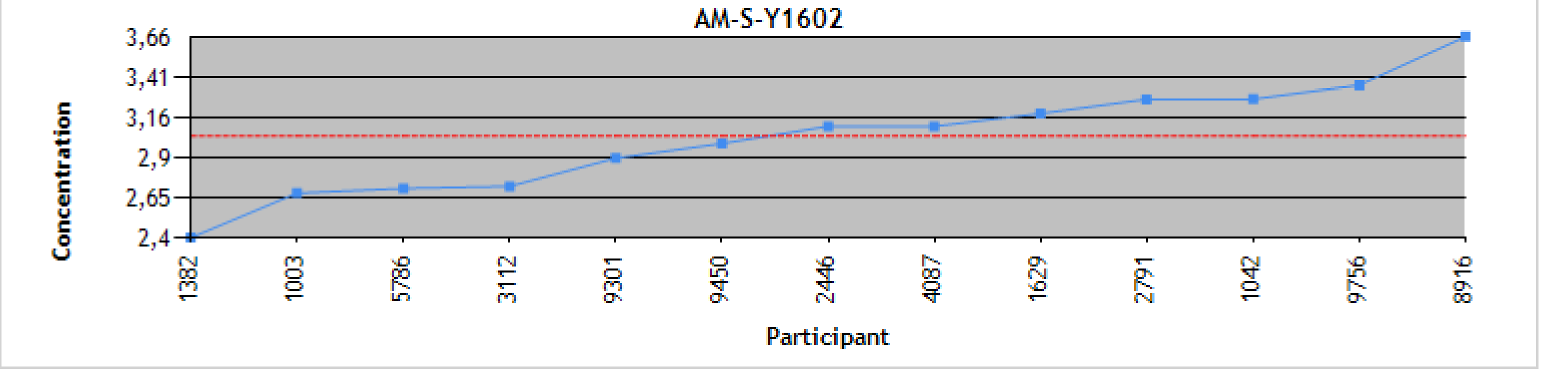

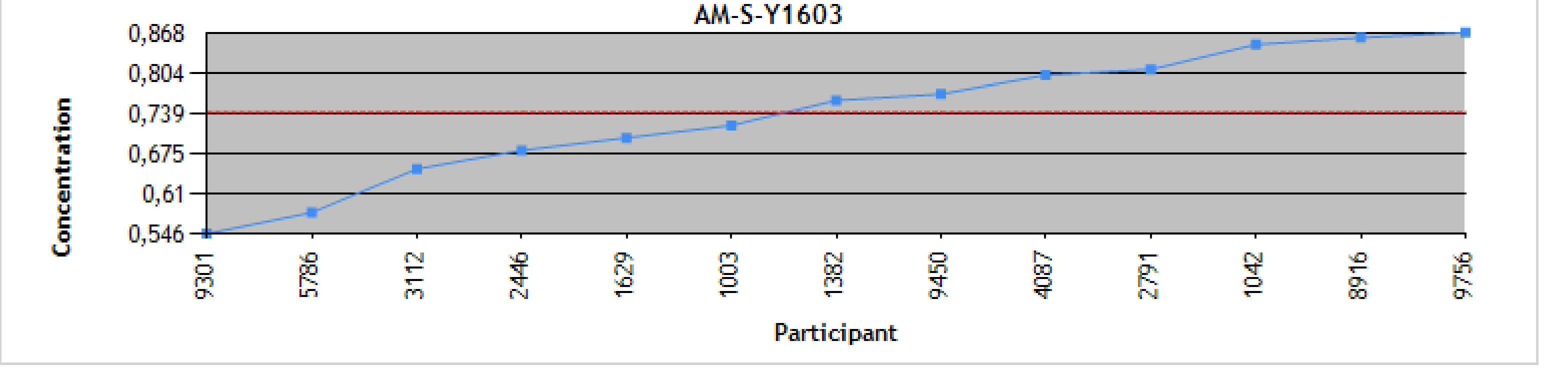

Individual results

Serum PFOA (µg/L)

Round #2016-01

Participant	AM-S-Y1601	z'-score	AM-S-Y1602	z'-score	AM-S-Y1603	z'-score	Method
**1003**	21.8	-1.14	11.9	-1.59	1.79	-0.64	LC-MS
**1042**	27.1	0.68	16.0	0.80	2.23	0.71	LC-MS
**1382**	23.3	-0.62	10.8	**-2.23**	2.02	0.06	LC-MS
**1629**	28.9	1.32	15.0	0.24	2.27	0.83	ND
**2446**	26.5	0.49	15.6	0.57	1.77	-0.71	ND
**2791**	24.9	-0.07	14.9	0.18	2.21	0.64	LC-MS
**3112**	22.5	-0.92	13.0	-0.92	1.71	-0.89	LC-MS
**4087**	24.4	-0.24	15.6	0.59	2.28	0.86	ND
**5786**	24.4	-0.24	13.7	-0.53	1.40	-1.84	ND
**7944**	19.8	-1.84	10.9	**-2.18**	0.932	**-3.28**	LC-MS
**8916**	26.9	0.62	15.6	0.59	2.21	0.64	LC-MS
**9301**	27.2	0.73	15.4	0.47	2.07	0.21	LC-MS
**9450**	25.3	0.08	14.5	-0.09	2.02	0.07	LC-MS
**9756**	26.6	0.51	16.2	0.95	2.25	0.77	LC-MS

	Assigned value	Standard uncertainty	σ pt	Acceptable range	K-S (Lilliefors)	Species
**AM-S-Y1601**	25.1	0.865	2.75	19.3 - 30.9	Accepted	---
**AM-S-Y1602**	14.6	0.446	1.65	11.2 - 18.0	Accepted	---
**AM-S-Y1603**	2.00	0.100	0.310	1.35 - 2.65	Accepted	---

Statistics
Serum PFOA (µg/L)

All methods	AM-S-Y1601	AM-S-Y1602	AM-S-Y1603
N	14	14	14
Robust mean Algo A	25.1	14.6	2.00
Robust STDev	2.59	1.33	0.301
Median	25.1	15.0	2.05
STDev from MAD	2.67	1.23	0.318
Arithmetic mean	25.0	14.2	1.94
STDev	2.49	1.86	0.392
CV or Variability	10.3%	9.1%	15.0%

LC-MS	AM-S-Y1601	AM-S-Y1602	AM-S-Y1603
N	10	10	10
Robust mean Algo A	24.6	14.1	2.02
Robust STDev	2.68	2.13	0.254
Median	25.1	14.7	2.05
STDev from MAD	2.78	2.09	0.258
Arithmetic mean	24.5	13.9	1.94
STDev	2.58	2.09	0.401
CV or Variability	10.9%	15.1%	12.6%

When fewer than 20 results were considered for statistical treatment of all or a sub-sample of results, the accuracy of statistical data may be questionable.

Distribution

Serum PFOA (µg/L)

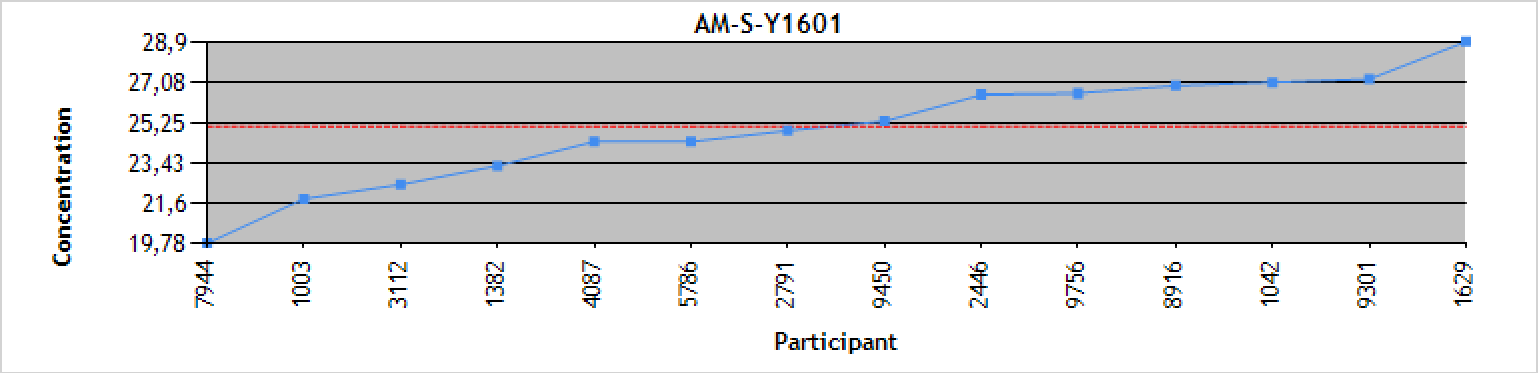

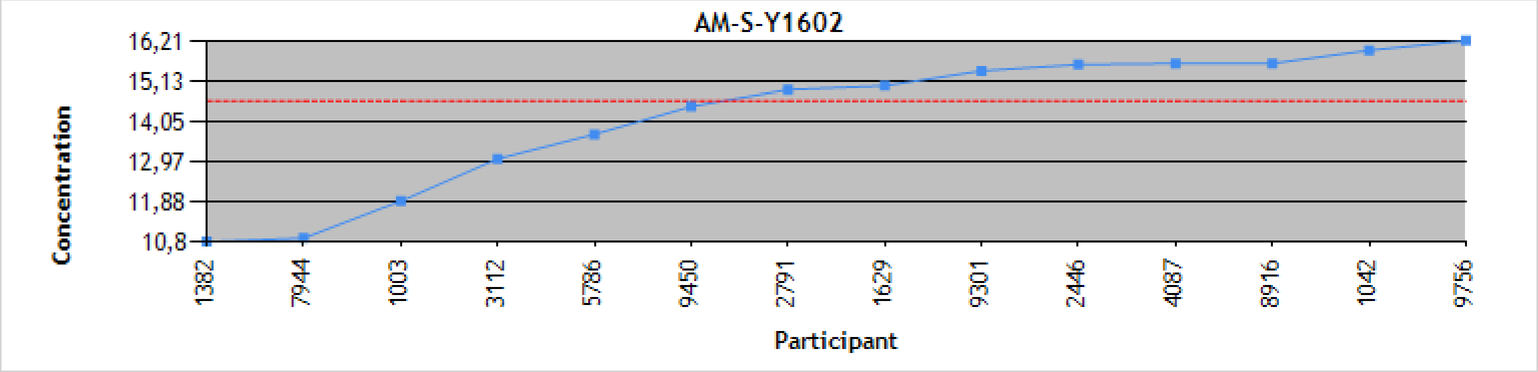

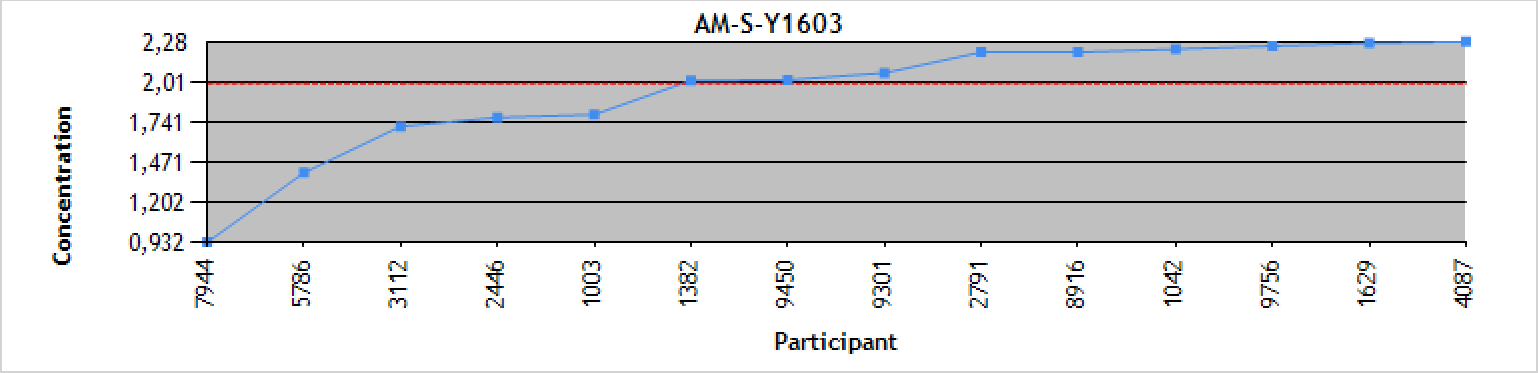

Individual results

Serum PFOS (µg/L)

Round #2016-01

Participant	AM-S-Y1601	z'-score	AM-S-Y1602	z'-score	AM-S-Y1603	z'-score	Method
**1003**	65.0	-1.95	100	**-2.10**	32.2	-1.75	LC-MS
**1042**	89.5	-0.19	147	0.09	43.4	-0.17	LC-MS
**1382**	86.2	-0.43	110	-1.64	41.2	-0.48	LC-MS
**1629**	95.9	0.27	142	-0.14	45.2	0.08	ND
**2446**	105	0.90	174	1.33	48.2	0.50	ND
**2791**	99.0	0.49	149	0.19	52.3	1.09	LC-MS
**3112**	64.3	**-2.01**	100	**-2.09**	30.5	-1.98	LC-MS
**4087**	83.4	-0.63	147	0.11	50.3	0.80	ND
**5786**	91.4	-0.06	148	0.14	41.9	-0.38	ND
**7944**	94.5	0.16	138	-0.30	43.6	-0.15	LC-MS
**8916**	94.2	0.14	150	0.23	44.4	-0.03	LC-MS
**9301**	108	1.13	155	0.47	55.6	1.55	LC-MS
**9450**	86.8	-0.39	139	-0.29	41.5	-0.44	LC-MS
**9756**	101	0.60	168	1.07	48.2	0.51	LC-MS

	Assigned value	Standard uncertainty	σ pt	Acceptable range	K-S (Lilliefors)	Species
**AM-S-Y1601**	92.2	3.39	13.5	64.4 - 120	Accepted	---
**AM-S-Y1602**	145	4.47	20.9	102 - 188	Accepted	---
**AM-S-Y1603**	44.6	1.91	6.83	30.4 - 58.8	Accepted	---

Statistics
Serum PFOS (µg/L)

All methods	AM-S-Y1601	AM-S-Y1602	AM-S-Y1603
**N**	14	14	14
**Robust mean Algo A**	92.2	145	44.6
**Robust STDev**	10.1	13.4	5.72
**Median**	92.8	147	44.0
**STDev from MAD**	9.49	12.0	5.16
**Arithmetic mean**	90.2	141	44.2
**STDev**	12.9	22.5	6.90
**CV or Variability**	11.0%	9.2%	12.8%

LC-MS	AM-S-Y1601	AM-S-Y1602	AM-S-Y1603
**N**	10	10	10
**Robust mean Algo A**	91.2	141	43.6
**Robust STDev**	10.7	17.2	6.28
**Median**	91.9	143	43.5
**STDev from MAD**	9.49	14.3	5.21
**Arithmetic mean**	88.8	136	43.3
**STDev**	14.3	23.9	7.83
**CV or Variability**	11.8%	12.2%	14.4%

When fewer than 20 results were considered for statistical treatment of all or a sub-sample of results, the accuracy of statistical data may be questionable.

Distribution
Serum PFOS (µg/L)

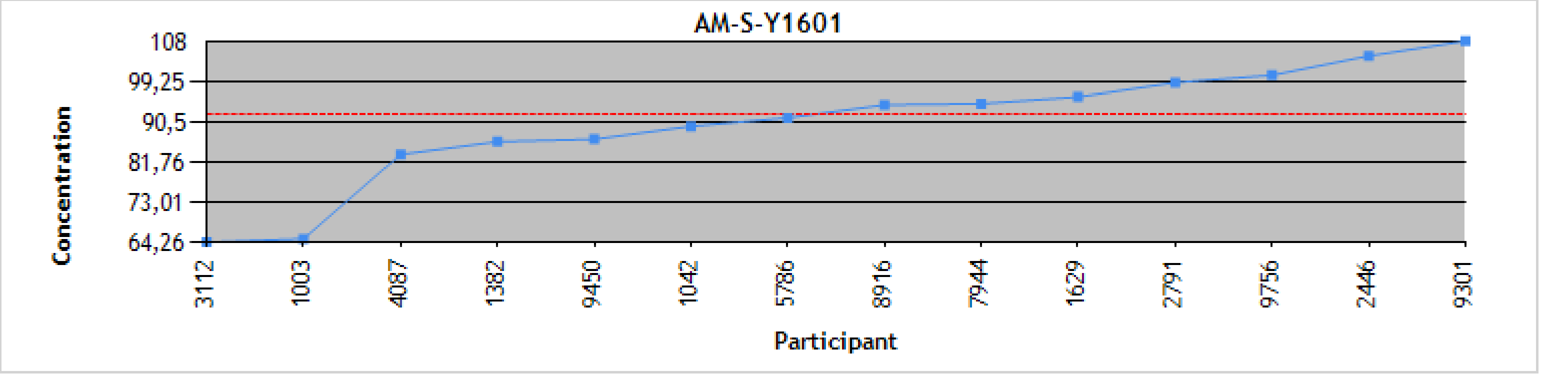

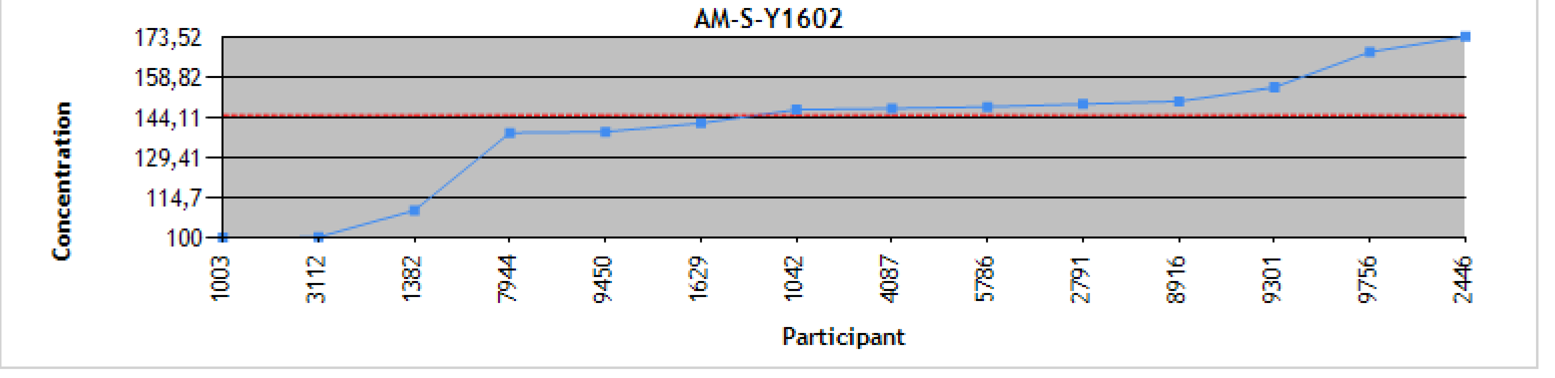

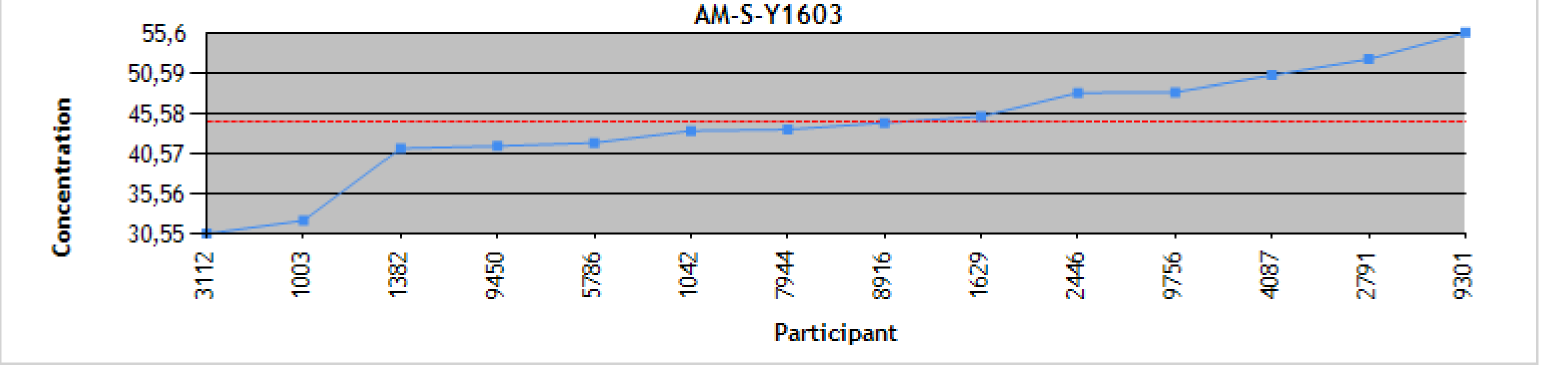

Individual results
Serum PFUdA (µg/L)
Round #2016-01

Participant	AM-S-Y1601	z'-score	AM-S-Y1602	z'-score	AM-S-Y1603	z'-score	Method
**1003**	1.79	-0.74	2.85	-1.26	0.350	-0.46	LC-MS
**1042**	2.24	0.71	3.88	0.57	0.405	0.45	LC-MS
**1382**	1.83	-0.61	2.48	-1.91	0.360	-0.30	LC-MS
**1629**	2.18	0.51	4.02	0.81	0.280	-1.63	ND
**2446**	2.01	-0.03	3.77	0.37	0.350	-0.46	ND
**3112**	1.71	-0.99	2.87	-1.23	0.341	-0.61	LC-MS
**4087**	1.90	-0.39	4.40	1.49	0.500	**2.03**	ND
**5786**	1.97	-0.16	3.65	0.16	0.330	-0.80	ND
**8916**	2.25	0.74	3.88	0.57	0.460	1.36	LC-MS
**9301**	2.11	0.29	3.11	-0.80	0.421	0.71	LC-MS
**9450**	2.14	0.39	3.55	-0.02	0.368	-0.17	LC-MS
**9756**	2.08	0.18	3.65	0.15	0.422	0.73	LC-MS

	Assigned value	Standard uncertainty	σ pt	Acceptable range	K-S (Lilliefors)	Species
**AM-S-Y1601**	2.02	0.0723	0.303	1.40 - 2.64	Accepted	---
**AM-S-Y1602**	3.56	0.186	0.534	2.43 - 4.69	Accepted	---
**AM-S-Y1603**	0.378	0.0203	0.0567	0.258 - 0.498	Accepted	---

Statistics

Serum PFUdA (µg/L)

All methods	AM-S-Y1601	AM-S-Y1602	AM-S-Y1603
N	12	12	12
Robust mean Algo A	2.02	3.56	0.378
Robust STDev	0.200	0.516	0.0562
Median	2.04	3.65	0.364
STDev from MAD	0.208	0.448	0.0558
Arithmetic mean	2.02	3.51	0.382
STDev	0.180	0.565	0.0612
CV or Variability	9.9%	14.5%	14.9%

LC-MS	AM-S-Y1601	AM-S-Y1602	AM-S-Y1603
N	8	8	8
Robust mean Algo A	2.02	3.28	0.391
Robust STDev	0.230	0.599	0.0479
Median	2.09	3.33	0.387
STDev from MAD	0.227	0.701	0.0515
Arithmetic mean	2.02	3.28	0.391
STDev	0.211	0.528	0.0422
CV or Variability	11.4%	18.2%	12.3%

When fewer than 20 results were considered for statistical treatment of all or a sub-sample of results, the accuracy of statistical data may be questionable.

Distribution

Serum PFUdA (µg/L)

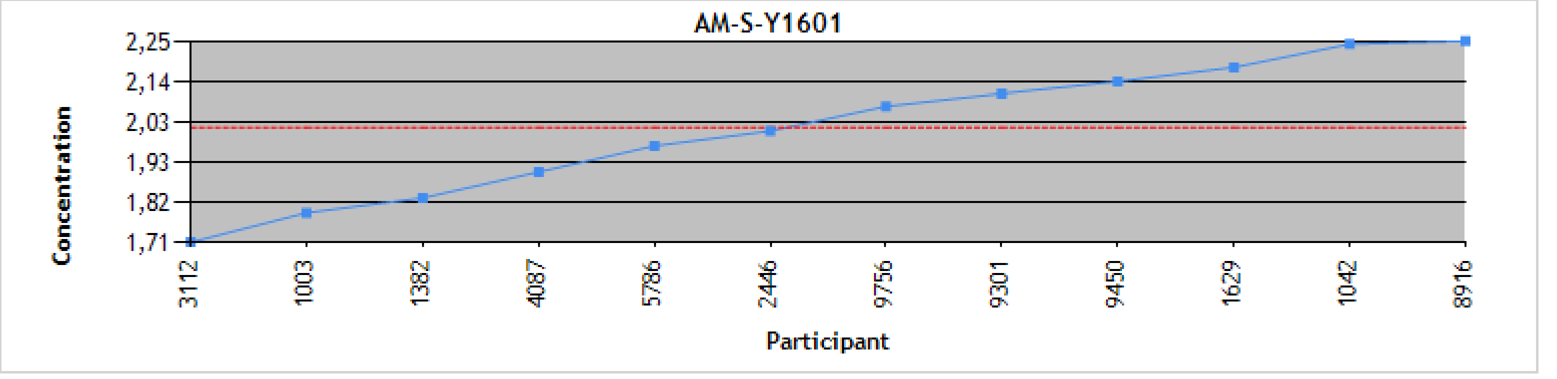

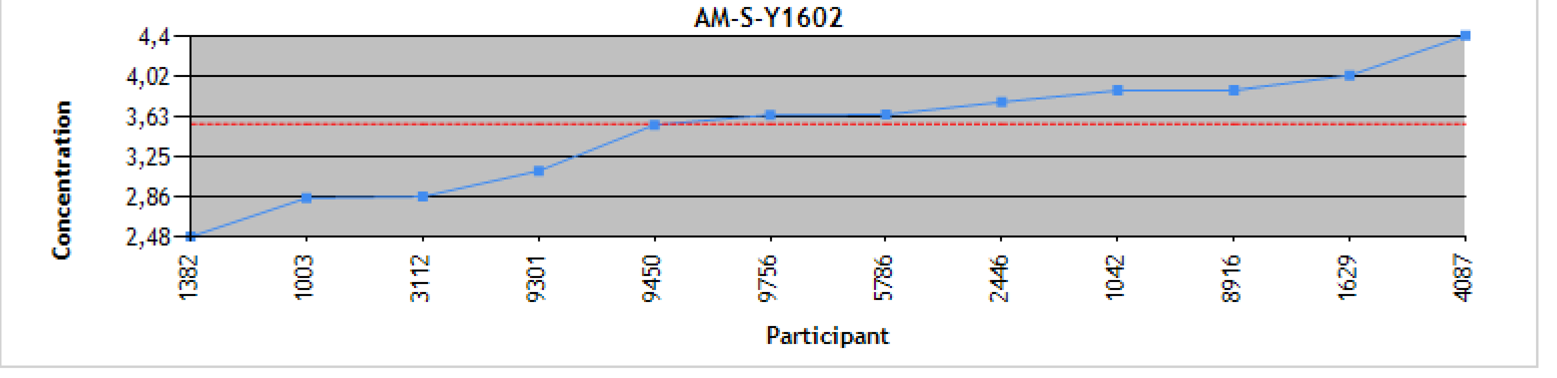

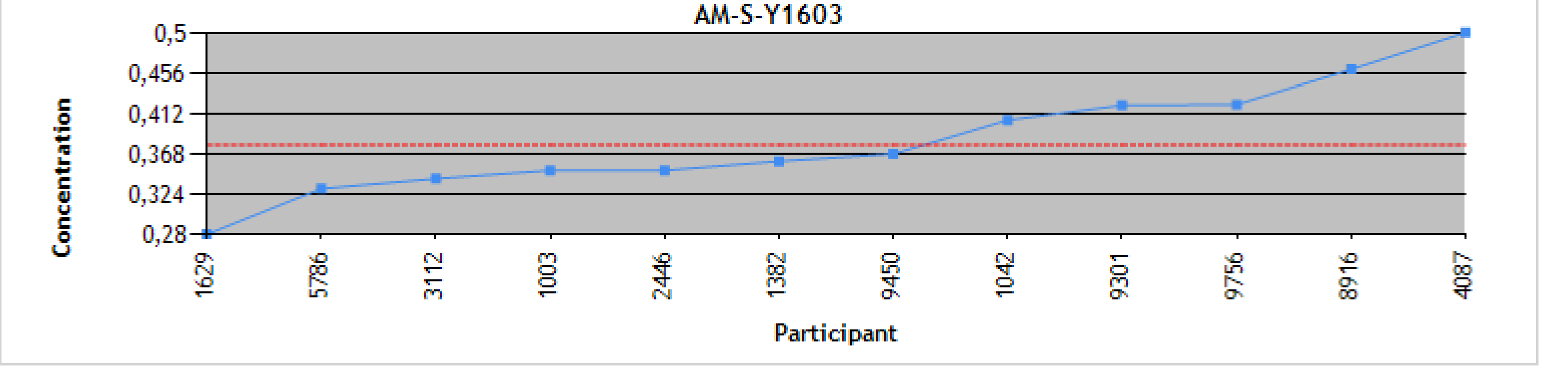

Individual results

Serum Total Cholesterol (g/L)

Round #2016-01

Participant	AM-S-W1601	z'-score	AM-S-W1602	z'-score	AM-S-W1603	z'-score	Method
1042	1.90	-0.58	1.88	-0.64	1.64	-0.50	ND
2446	1.94	0.16	1.96	0.58	1.70	0.50	ND
4635	1.95	0.26	1.97	0.68	1.69	0.40	ND
7660	1.92	-0.13	1.88	-0.60	1.63	-0.63	ND

	Assigned value	Standard uncertainty	σ pt	Acceptable range	K-S (Lilliefors)	Species
AM-S-W1601	1.93	0.0134	0.0593	1.81 - 2.05	Accepted	---
AM-S-W1602	1.92	0.0353	0.0592	1.78 - 2.06	Accepted	---
AM-S-W1603	1.67	0.0251	0.0549	1.55 - 1.79	Accepted	---

Total cholesterol is not included in the scope of our accreditation.

Statistics

Serum Total Cholesterol (g/L)

All methods	AM-S-W1601	AM-S-W1602	AM-S-W1603
**N**	4	4	4
**Robust mean Algo A**	1.93	1.92	1.67
**Robust STDev**	0.0214	0.0564	0.0402
**Median**	1.93	1.92	1.67
**STDev from MAD**	0.0178	0.0623	0.0445
**Arithmetic mean**	1.93	1.92	1.67
**STDev**	0.0229	0.0497	0.0355
**Comparison AM-S-W1505**	1.92		
	0.0556		
**CV or Variability**	1.1%	2.9%	2.4%

When fewer than 20 results were considered for statistical treatment of all or a sub-sample of results, the accuracy of statistical data may be questionable.

Distribution
Serum Total Cholesterol (g/L)

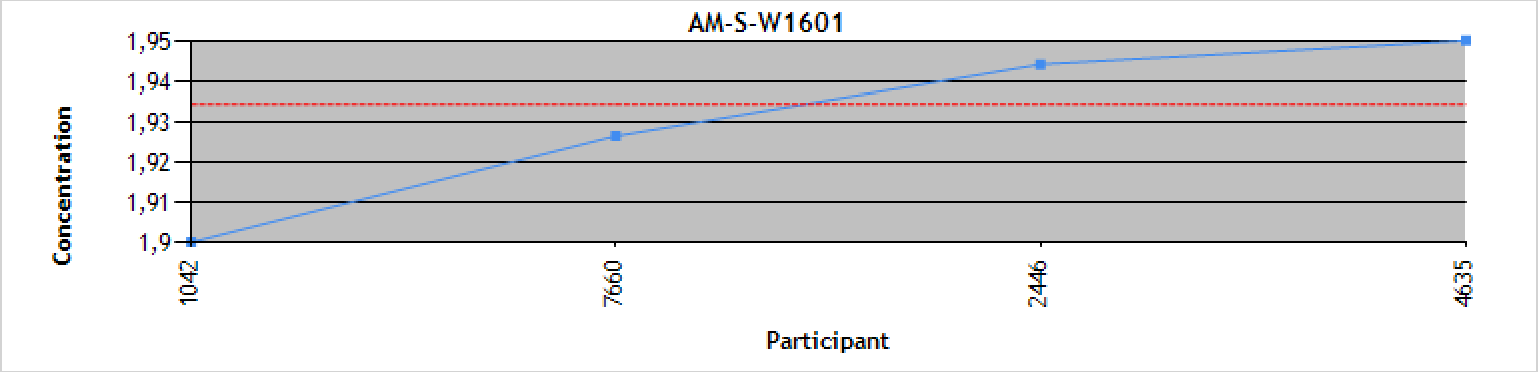

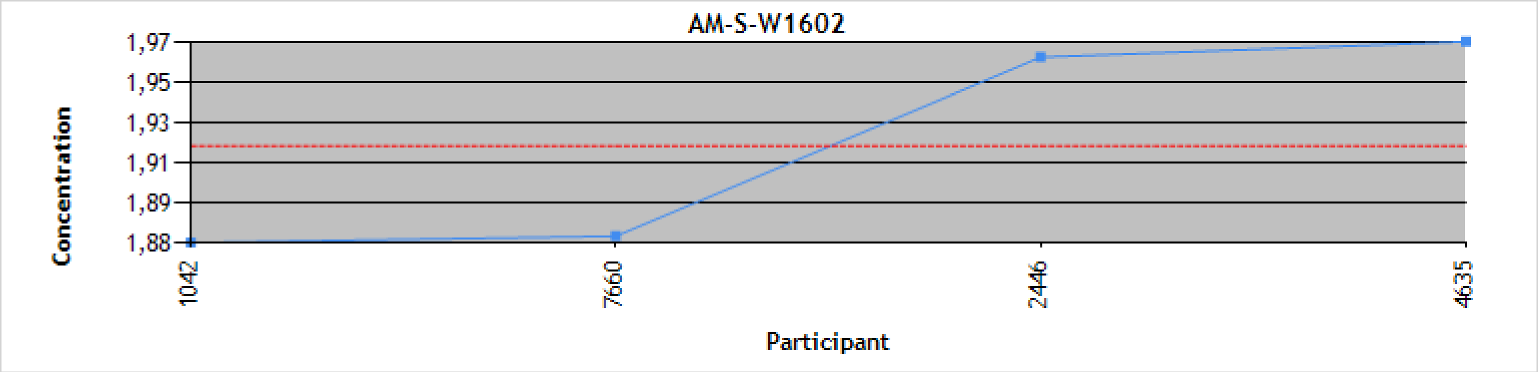

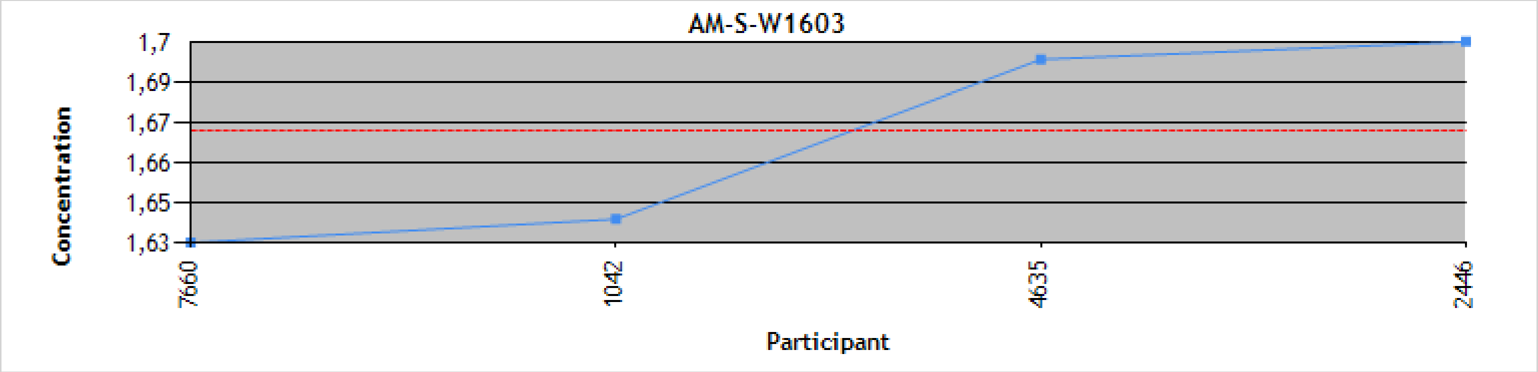

Individual results

Serum Total lipids (g/L)

Round #2016-01

Participant	AM-S-W1601	z'-score	AM-S-W1602	z'-score	AM-S-W1603	z'-score	Method
**1042**	6.11	0.78	6.11	0.87	5.66	0.61	ND
**2446**	5.99	0.59	6.10	0.86	5.44	0.21	ND
**3112**	5.48	-0.22	4.40	-1.74	4.76	-1.00	ND
**4635**	6.17	0.88	6.21	1.03	5.63	0.56	ND
**7660**	6.23	0.97	6.24	1.07	5.64	0.57	ND
**8559**	5.25	-0.59	5.25	-0.44	4.75	-1.02	ND
**8916**	4.98	-1.02	5.25	-0.44	4.75	-1.02	GC
**9880**	3.70	**-3.05**	4.07	**-2.25**	---	---	ND

	Assigned value	Standard uncertainty	σ pt	Acceptable range	K-S (Lilliefors)	Species
**AM-S-W1601**	5.62	0.296	0.555	4.36 - 6.88	Accepted	---
**AM-S-W1602**	5.54	0.357	0.548	4.23 - 6.85	Accepted	---
**AM-S-W1603**	5.32	0.188	0.526	4.20 - 6.44	Accepted	---

Total lipids is not included in the scope of our accreditation.

Statistics

Serum Total lipids (g/L)

All methods	AM-S-W1601	AM-S-W1602	AM-S-W1603
N	8	8	7
Robust mean Algo A	5.62	5.54	5.32
Robust STDev	0.670	0.808	0.398
Median	5.74	5.68	5.44
STDev from MAD	0.683	0.722	0.326
Arithmetic mean	5.49	5.45	5.23
STDev	0.859	0.858	0.455
Comparison AM-S-W1505	5.89		
	0.569		
CV or Variability	11.9%	14.6%	7.5%

When fewer than 20 results were considered for statistical treatment of all or a sub-sample of results, the accuracy of statistical data may be questionable.

Distribution
Serum Total lipids (g/L)

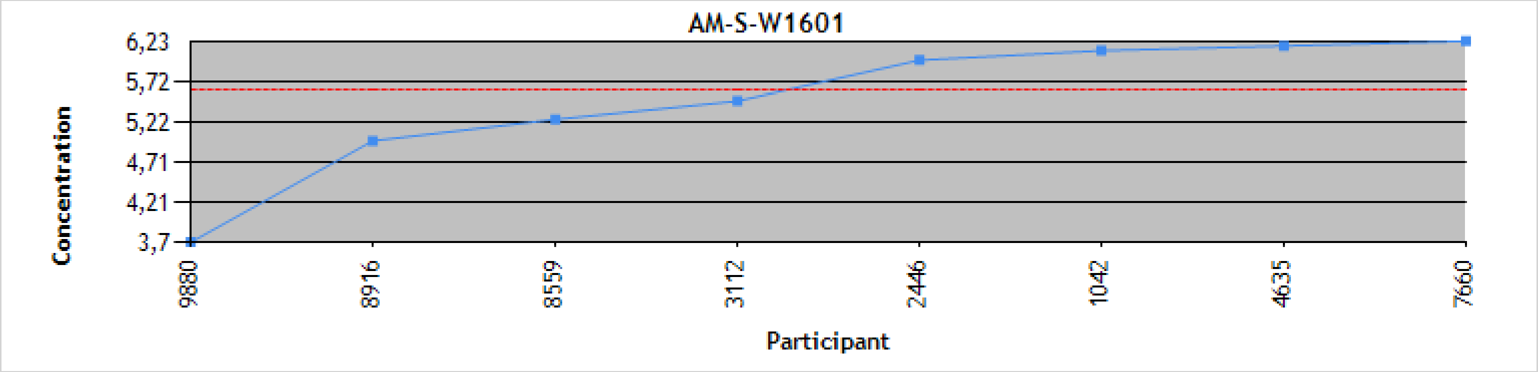

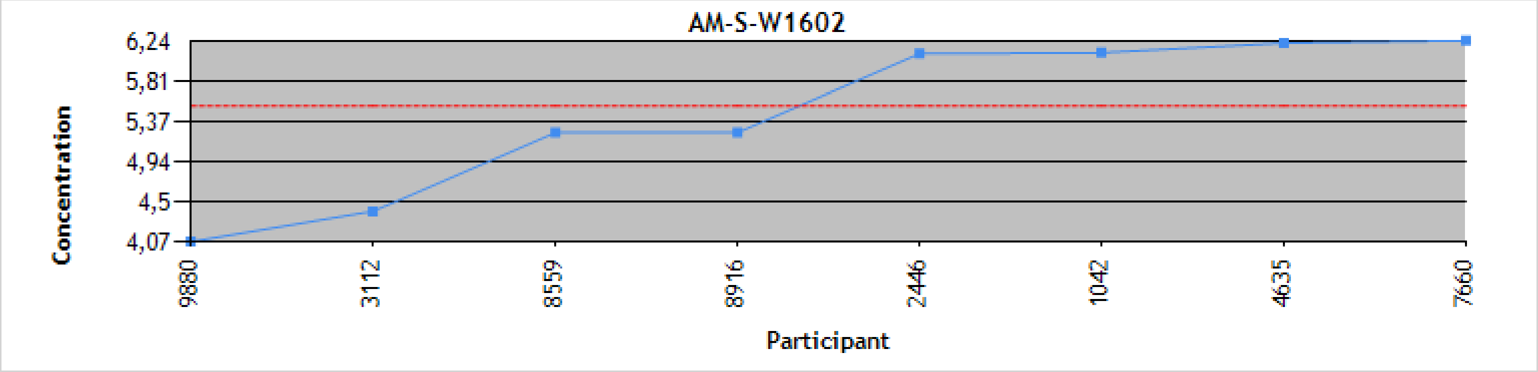

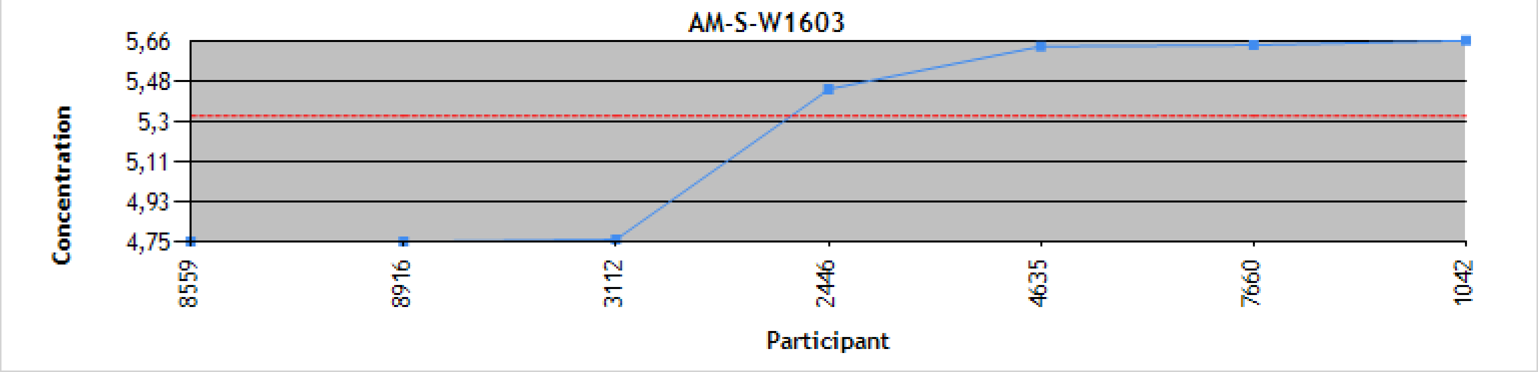

Individual results

Serum Toxaphene Parlar # 26 (µg/L)

Round #2016-01

Participant	AM-S-W1601	z'-score	AM-S-W1602	z'-score	AM-S-W1603	z'-score	Method
**1042**	0.366	0.28	0.139	-0.09	0.0705	-0.03	GC-MS CI
**6865**	0.323	-0.51	0.141	0.00	0.0703	-0.05	ND
**7660**	0.382	0.56	0.141	-0.02	0.0710	0.01	GC-MS CI
**8916**	0.332	-0.34	0.146	0.23	0.0780	0.60	GC

	Assigned value	Standard uncertainty	σ pt	Acceptable range	K-S (Lilliefors)	Species
**AM-S-W1601**	0.351	0.0198	0.0516	0.240 - 0.462	Accepted	---
**AM-S-W1602**	0.141	0.00114	0.0218	0.0973 - 0.185	Accepted	---
**AM-S-W1603**	0.0709	0.000406	0.0119	0.0472 - 0.0946	Rejected[1]	---

Statistics
Serum Toxaphene Parlar # 26 (µg/L)

All methods	AM-S-W1601	AM-S-W1602	AM-S-W1603
**N**	4	4	4
**Robust mean Algo A**	0.351	0.141	0.0709
**Robust STDev**	0.0317	0.00183	0.000650
**Median**	0.349	0.141	0.0708
**STDev from MAD**	0.0322	0.00144	0.000541
**Arithmetic mean**	0.351	0.142	0.0725
**STDev**	0.0280	0.00300	0.00371
**Comparison AM-S-W1505**	0.373		
	0.0162		
**CV or Variability**	9.0%	1.3%	0.9%

When fewer than 20 results were considered for statistical treatment of all or a sub-sample of results, the accuracy of statistical data may be questionable.

Distribution
Serum Toxaphene Parlar # 26 (µg/L)

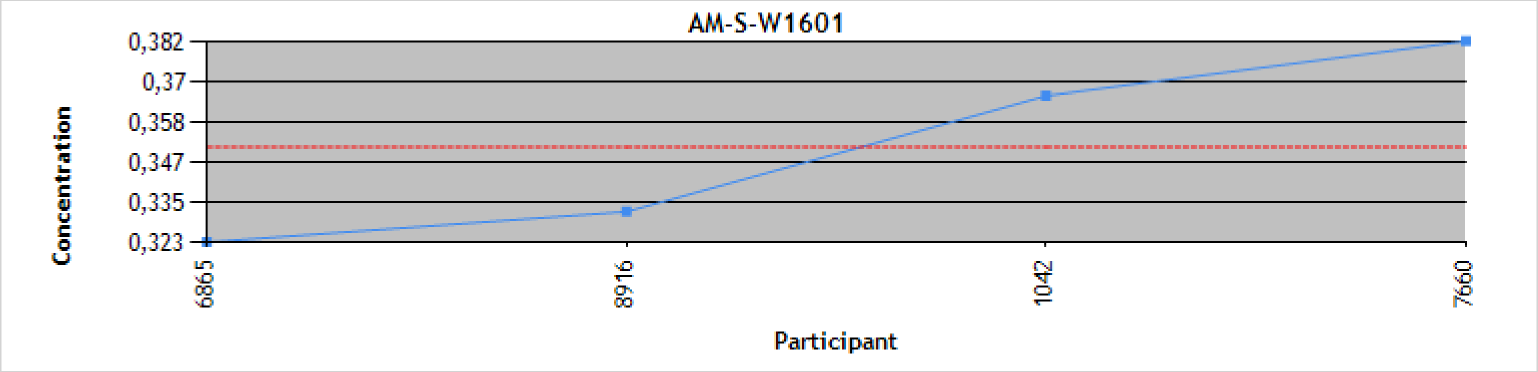

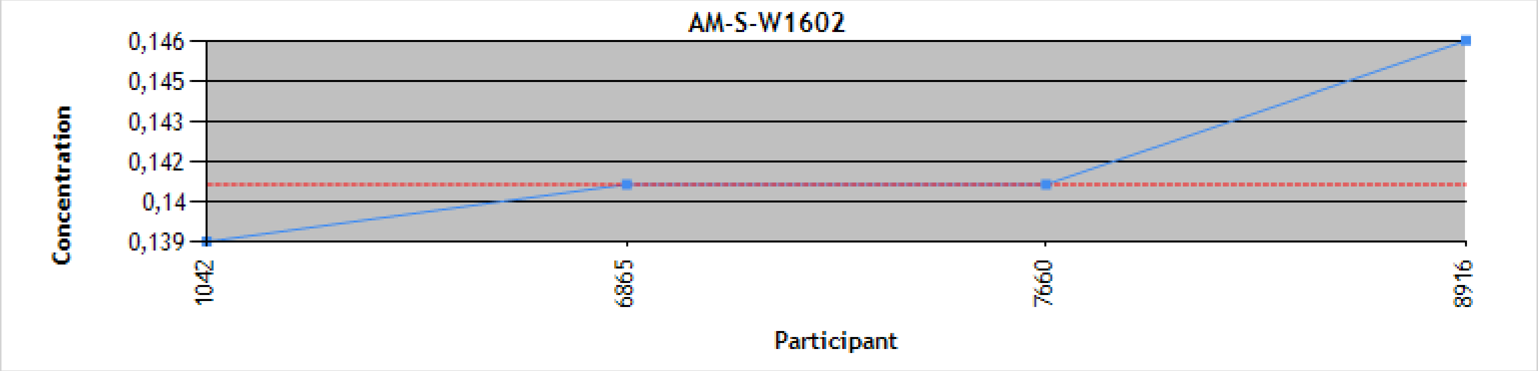

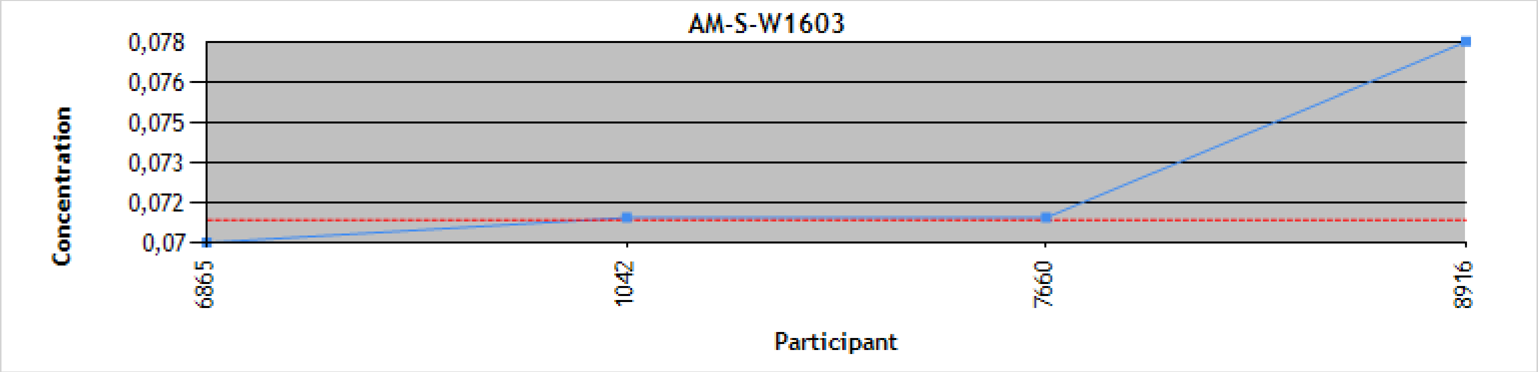

Individual results

Serum Toxaphene Parlar # 50 (µg/L)

Round #2016-01

Participant	AM-S-W1601	z'-score	AM-S-W1602	z'-score	AM-S-W1603	z'-score	Method
1042	0.172	0.21	0.0882	0.02	0.434	0.65	GC-MS CI
6865	0.170	0.12	0.0955	0.53	0.336	-0.81	ND
7660	0.166	-0.04	0.0833	-0.32	0.424	0.51	GC-MS CI
8916	0.153	-0.54	0.0870	-0.06	0.366	-0.36	GC

	Assigned value	Standard uncertainty	σ pt	Acceptable range	K-S (Lilliefors)	Species
AM-S-W1601	0.167	0.00357	0.0258	0.115 - 0.219	Accepted	---
AM-S-W1602	0.0879	0.00283	0.0142	0.0590 - 0.117	Accepted	---
AM-S-W1603	0.390	0.0332	0.0582	0.256 - 0.524	Accepted	---

Statistics

Serum Toxaphene Parlar # 50 (µg/L)

All methods	AM-S-W1601	AM-S-W1602	AM-S-W1603
N	4	4	4
Robust mean Algo A	0.167	0.0879	0.390
Robust STDev	0.00570	0.00453	0.0531
Median	0.168	0.0876	0.395
STDev from MAD	0.00474	0.00360	0.0503
Arithmetic mean	0.165	0.0885	0.390
STDev	0.00864	0.00511	0.0468
Comparison AM-S-W1505	0.168		
	0.00449		
CV or Variability	3.4%	5.2%	13.6%

When fewer than 20 results were considered for statistical treatment of all or a sub-sample of results, the accuracy of statistical data may be questionable.

Distribution

Serum Toxaphene Parlar # 50 (µg/L)

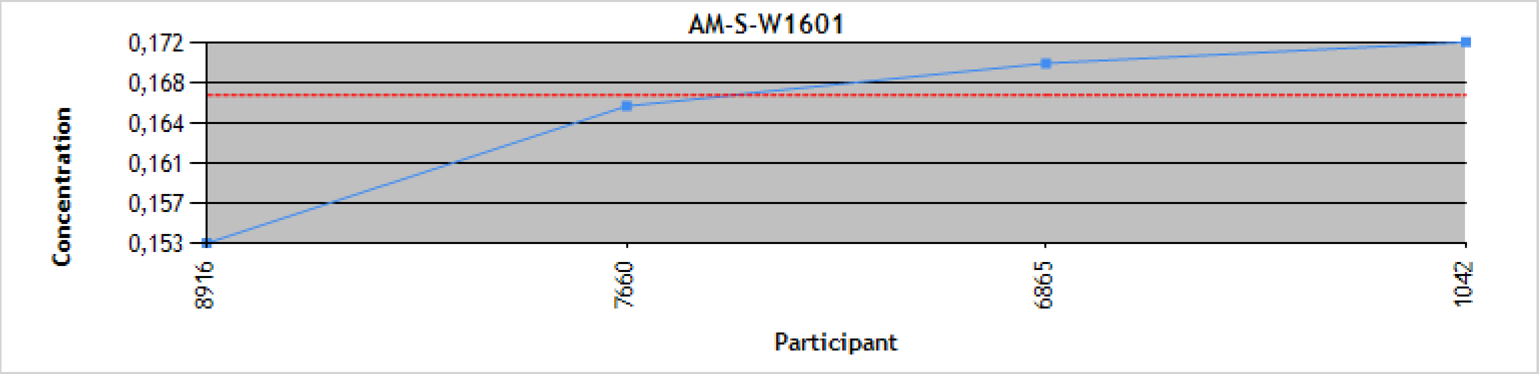

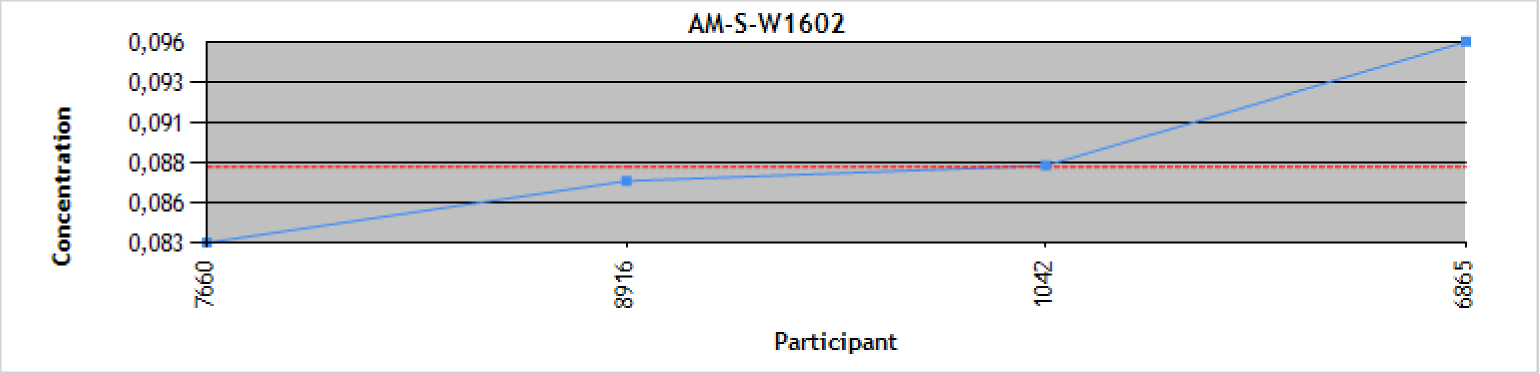

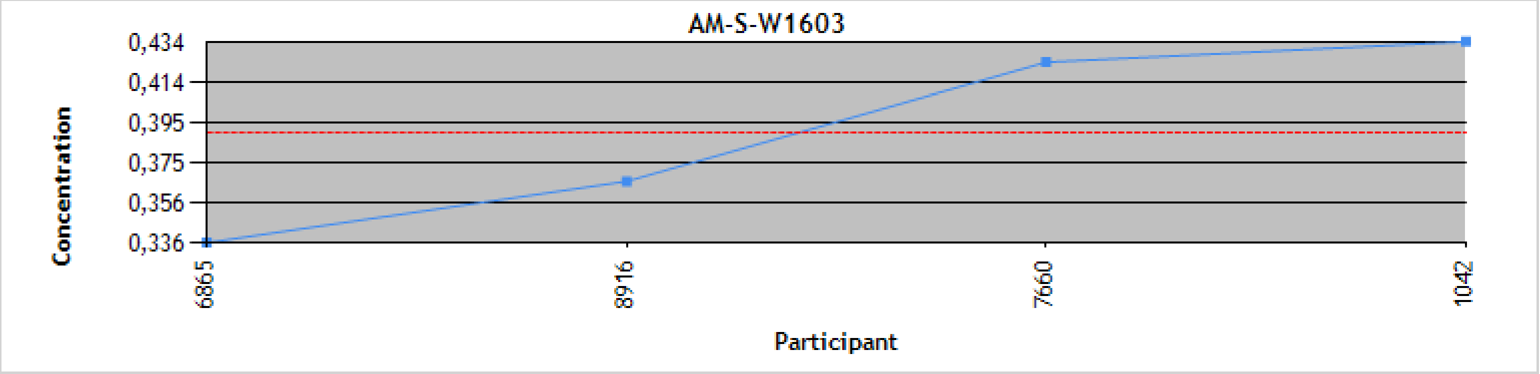

Individual results

Serum trans-Nonachlor (µg/L)

Round #2016-01

Participant	AM-S-W1601	z'-score	AM-S-W1602	z'-score	AM-S-W1603	z'-score	Method
1003	2.62	-0.45	1.69	0.39	3.56	-0.24	GC-MS-MS
1042	2.77	0.08	1.63	0.08	3.66	0.02	GC-MS CI
1629	2.98	0.79	2.01	**2.26**	5.00	**3.58**	GC-MS-MS
2446	2.76	0.03	1.66	0.23	3.82	0.45	ND
3112	2.75	-0.02	1.54	-0.47	3.76	0.29	GC-MS CI
4635	2.98	0.79	1.69	0.41	3.77	0.32	GC-MS EI
6865	2.62	-0.45	1.59	-0.18	3.40	-0.67	ND
7660	2.69	-0.22	1.59	-0.17	3.49	-0.43	GC-MS CI
8559	2.63	-0.42	1.60	-0.12	3.25	-1.07	ND
8916	2.37	-1.33	1.38	-1.43	3.07	-1.55	GC
9301	2.98	0.80	1.57	-0.29	3.73	0.21	GC-MS-MS
9880	---	---	---	---	3.79	0.37	GC-MS-MS

	Assigned value	Standard uncertainty	σ pt	Acceptable range	K-S (Lilliefors)	Species
AM-S-W1601	2.75	0.0727	0.279	2.17 - 3.33	Accepted	---
AM-S-W1602	1.62	0.0303	0.168	1.28 - 1.96	Rejected[1]	---
AM-S-W1603	3.65	0.0781	0.367	2.90 - 4.40	Rejected[1]	---

Statistics
Serum trans-Nonachlor (µg/L)

All methods	AM-S-W1601	AM-S-W1602	AM-S-W1603
N	11	11	12
Robust mean Algo A	2.75	1.62	3.65
Robust STDev	0.193	0.0804	0.216
Median	2.75	1.60	3.69
STDev from MAD	0.185	0.0890	0.193
Arithmetic mean	2.74	1.63	3.69
STDev	0.188	0.152	0.473
Comparison AM-S-W1505	2.72		
	0.223		
CV or Variability	7.0%	5.0%	5.9%

GC-MS CI	AM-S-W1601	AM-S-W1602	AM-S-W1603
N	3	3	3
Robust mean Algo A	2.73	1.59	3.63
Robust STDev	0.0512	0.0532	0.154
Median	2.75	1.59	3.66
STDev from MAD	0.0431	0.0639	0.150
Arithmetic mean	2.73	1.59	3.63
STDev	0.0451	0.0470	0.136
CV or Variability	1.9%	3.4%	4.2%

GC-MS-MS	AM-S-W1601	AM-S-W1602	AM-S-W1603
N	3	3	4
Robust mean Algo A	2.98	1.73	3.77
Robust STDev	0.00562	0.217	0.214
Median	2.98	1.69	3.76
STDev from MAD	0.00445	0.172	0.171
Arithmetic mean	2.86	1.75	4.02
STDev	0.207	0.226	0.658
CV or Variability	0.2%	12.5%	5.7%

When fewer than 20 results were considered for statistical treatment of all or a sub-sample of results, the accuracy of statistical data may be questionable.

Distribution

Serum trans-Nonachlor (µg/L)

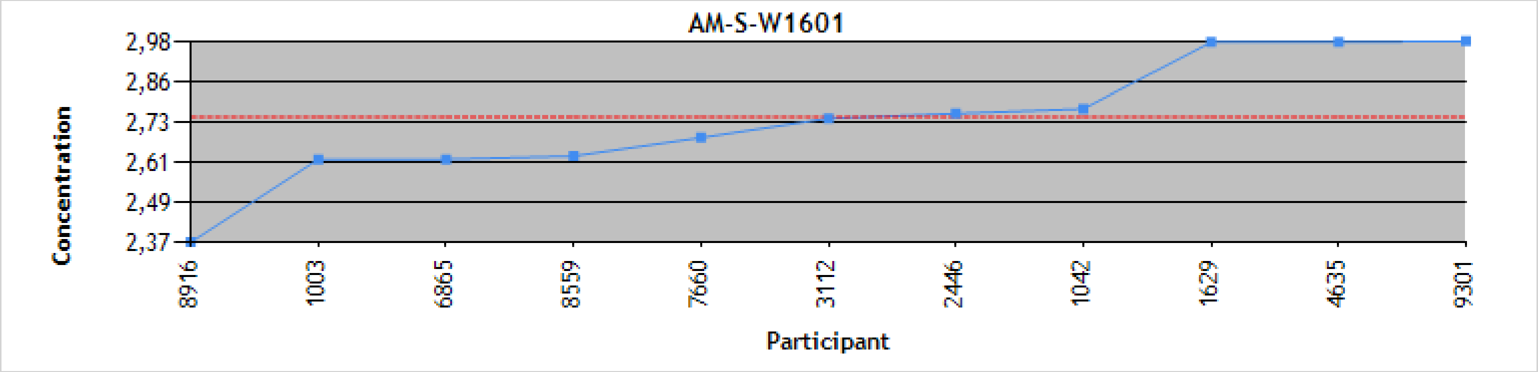

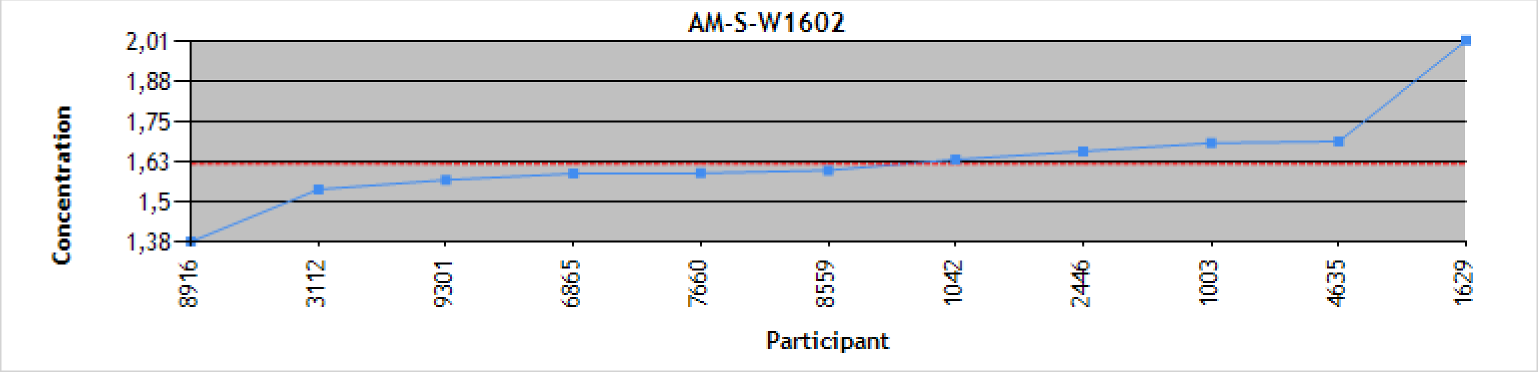

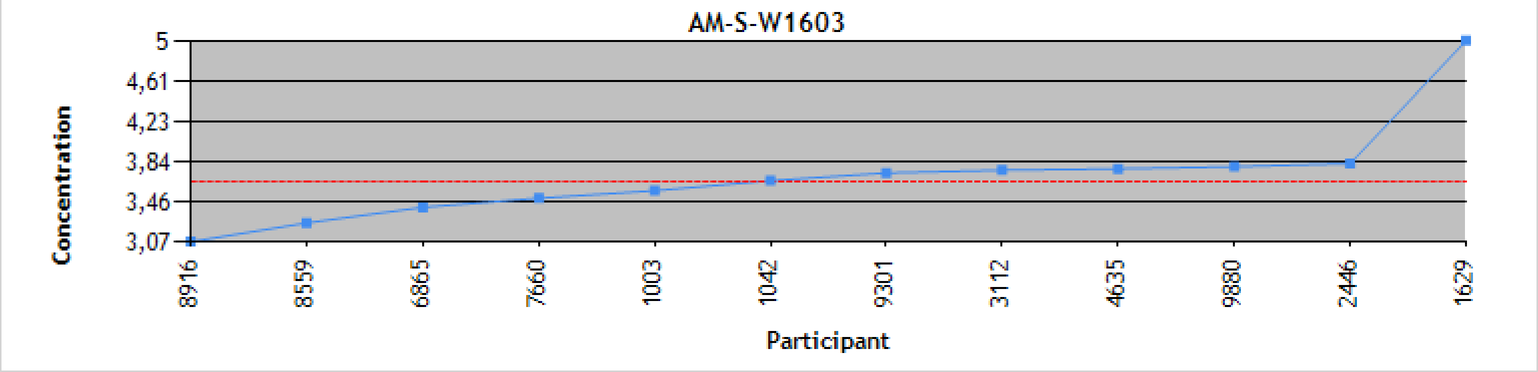

Individual results

Serum Triglycerides (g/L)

Round #2016-01

Participant	AM-S-W1601	z'-score	AM-S-W1602	z'-score	AM-S-W1603	z'-score	Method
**1042**	1.04	-0.06	1.06	0.05	1.13	0.24	ND
**2446**	0.950	-1.25	0.940	-1.85	0.990	-1.89	ND
**4635**	1.13	1.28	1.13	1.00	1.17	0.88	ND
**7660**	1.05	0.07	1.05	-0.09	1.10	-0.19	ND

	Assigned value	Standard uncertainty	σ pt	Acceptable range	K-S (Lilliefors)	Species
**AM-S-W1601**	1.04	0.0527	0.0489	0.896 - 1.18	Accepted	---
**AM-S-W1602**	1.06	0.0422	0.0492	0.930 - 1.19	Accepted	---
**AM-S-W1603**	1.11	0.0387	0.0503	0.983 - 1.24	Accepted	---

Triglycerides is not included in the scope of our accreditation.

Statistics
Serum Triglycerides (g/L)

All methods	AM-S-W1601	AM-S-W1602	AM-S-W1603
N	4	4	4
Robust mean Algo A	1.04	1.06	1.11
Robust STDev	0.0844	0.0676	0.0619
Median	1.04	1.06	1.11
STDev from MAD	0.0704	0.0526	0.0504
Arithmetic mean	1.04	1.05	1.09
STDev	0.0744	0.0771	0.0752
Comparison AM-S-W1505	1.11		
	0.0837		
CV or Variability	8.1%	6.4%	5.6%

When fewer than 20 results were considered for statistical treatment of all or a sub-sample of results, the accuracy of statistical data may be questionable.

Distribution
Serum Triglycerides (g/L)

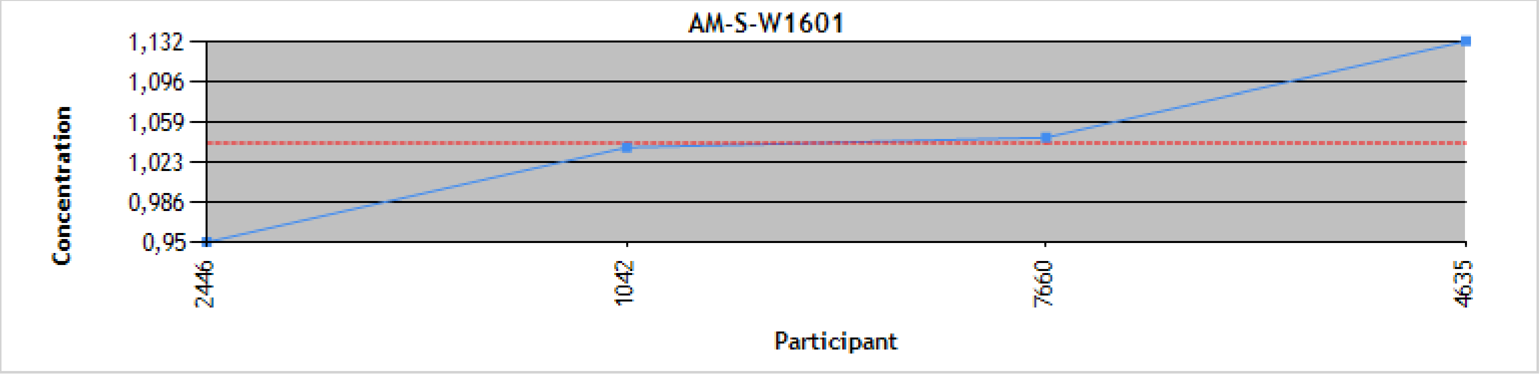

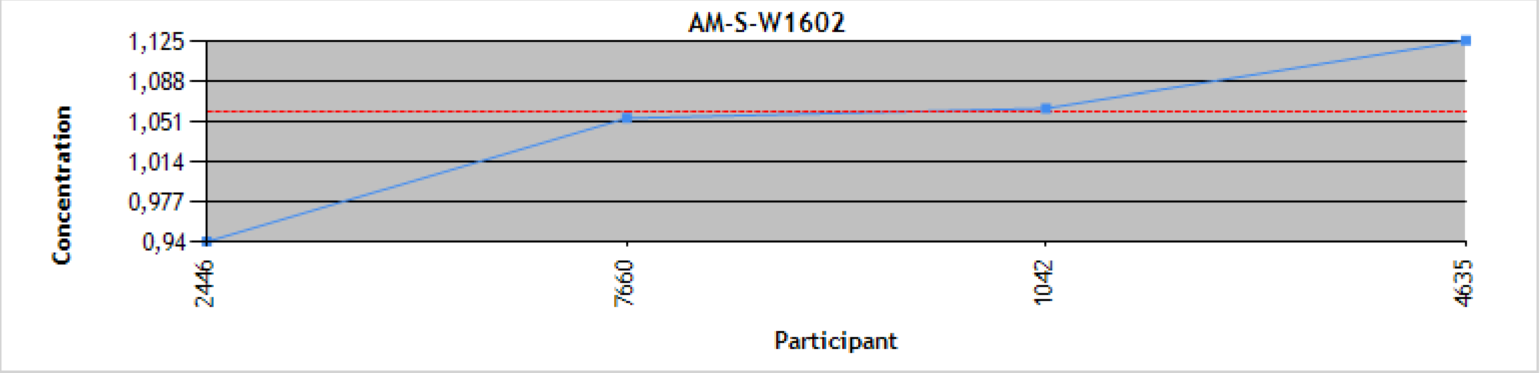

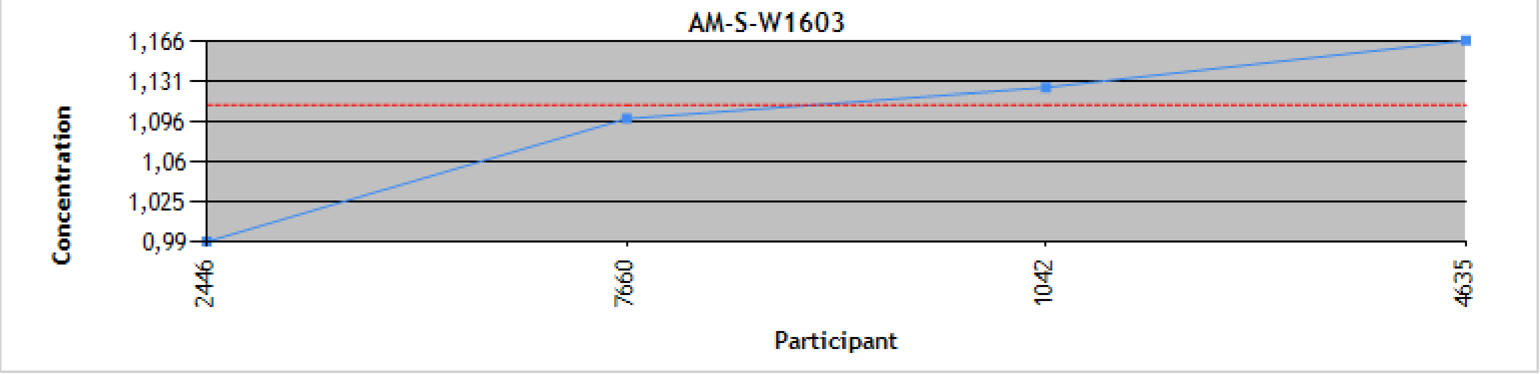

AMAP Ring Test for Persistent Organic Pollutants in Human Serum

ASSIGNED VALUES

ROUND: 2016-01

SHIPPED ON: 2016-01-25

DEADLINE: 2016-03-11

MATRIX	ANALYTE	UNIT	PTM	ASSIGNED VALUE	PTM	ASSIGNED VALUE	PTM	ASSIGNED VALUE
Serum	Dieldrin	µg/L	AM-S-W1601	---	AM-S-W1602	1.63	AM-S-W1603	0.644
	ß-HCH	µg/L	AM-S-W1601	1.49	AM-S-W1602	0.880	AM-S-W1603	1.80
	Heptachlor	µg/L	AM-S-W1601	1.16	AM-S-W1602	1.37	AM-S-W1603	0.768
	Heptachlor epoxide	µg/L	AM-S-W1601	1.57	AM-S-W1602	1.19	AM-S-W1603	0.260
	Hexachlorobenzene	µg/L	AM-S-W1601	1.54	AM-S-W1602	1.07	AM-S-W1603	0.732
	Oxychlordane	µg/L	AM-S-W1601	1.68	AM-S-W1602	0.299	AM-S-W1603	1.51
	p,p'-DDE	µg/L	AM-S-W1601	6.26	AM-S-W1602	5.22	AM-S-W1603	1.51
	p,p'-DDT	µg/L	AM-S-W1601	1.51	AM-S-W1602	0.744	AM-S-W1603	1.32
	PBDE IUPAC # 28	µg/L	AM-S-W1601	0.0970	AM-S-W1602	0.451	AM-S-W1603	0.0762
	PBDE IUPAC # 47	µg/L	AM-S-W1601	0.272	AM-S-W1602	1.46	AM-S-W1603	0.881
	PBDE IUPAC # 99	µg/L	AM-S-W1601	0.361	AM-S-W1602	0.159	AM-S-W1603	0.658
	PBDE IUPAC # 100	µg/L	AM-S-W1601	0.0927	AM-S-W1602	0.263	AM-S-W1603	0.617
	PBDE IUPAC # 153	µg/L	AM-S-W1601	0.490	AM-S-W1602	0.103	AM-S-W1603	0.940
	PBDE IUPAC # 154	µg/L	AM-S-W1601	0.314	AM-S-W1602	0.813	AM-S-W1603	0.143
	PBDE IUPAC # 183	µg/L	AM-S-W1601	0.520	AM-S-W1602	0.353	AM-S-W1603	0.716
	PBDE IUPAC # 209	µg/L	AM-S-W1601	1.42	AM-S-W1602	1.15	AM-S-W1603	0.276
	PCB IUPAC # 28	µg/L	AM-S-W1601	0.862	AM-S-W1602	0.253	AM-S-W1603	0.769
	PCB IUPAC # 52	µg/L	AM-S-W1601	0.0284	AM-S-W1602	0.779	AM-S-W1603	0.398
	PCB IUPAC # 74	µg/L	AM-S-W1601	0.567	AM-S-W1602	0.864	AM-S-W1603	0.451
	PCB IUPAC # 99	µg/L	AM-S-W1601	0.394	AM-S-W1602	0.133	AM-S-W1603	0.659
	PCB IUPAC # 101	µg/L	AM-S-W1601	0.0649	AM-S-W1602	0.609	AM-S-W1603	0.306

Centre de toxicologie/INSPQ
945, Ave Wolfe
Québec, QC
Canada, G1V 5B3
Phone: (418) 650-5115 Ext. 5254
Fax : (418) 654-2148
E-mail : ctq@inspq.qc.ca
http://www.inspq.qc.ca/ctq

Institut national de santé publique Québec
Centre de toxicologie

AMAP Ring Test for Persistent Organic Pollutants in Human Serum

ASSIGNED VALUES

ROUND: 2016-03
SHIPPED ON: 2016-01-25
DEADLINE: 2016-03-11

MATRIX	ANALYTE	UNIT	PTM	ASSIGNED VALUE	PTM	ASSIGNED VALUE	PTM	ASSIGNED VALUE
Serum	PCB IUPAC # 105	µg/L	AM-S-W1601	0.235	AM-S-W1602	0.422	AM-S-W1603	0.132
	PCB IUPAC # 118	µg/L	AM-S-W1601	0.141	AM-S-W1602	0.313	AM-S-W1603	0.750
	PCB IUPAC # 138	µg/L	AM-S-W1601	0.679	AM-S-W1602	1.92	AM-S-W1603	0.232
	PCB IUPAC # 153	µg/L	AM-S-W1601	1.70	AM-S-W1602	0.846	AM-S-W1603	0.916
	PCB IUPAC # 170	µg/L	AM-S-W1601	0.769	AM-S-W1602	0.262	AM-S-W1603	0.652
	PCB IUPAC # 180	µg/L	AM-S-W1601	1.14	AM-S-W1602	0.733	AM-S-W1603	1.77
	PCB IUPAC # 183	µg/L	AM-S-W1601	0.00645	AM-S-W1602	0.168	AM-S-W1603	0.800
	PCB IUPAC # 187	µg/L	AM-S-W1601	0.0293	AM-S-W1602	0.969	AM-S-W1603	1.74
	PFHxA	µg/L	AM-S-Y1601	6.78	AM-S-Y1602	4.58	AM-S-Y1603	1.03
	PFHxS	µg/L	AM-S-Y1601	12.1	AM-S-Y1602	22.0	AM-S-Y1603	5.58
	PFNA	µg/L	AM-S-Y1601	1.79	AM-S-Y1602	3.04	AM-S-Y1603	0.741
	PFOA	µg/L	AM-S-Y1601	25.1	AM-S-Y1602	14.6	AM-S-Y1603	2.00
	PFOS	µg/L	AM-S-Y1601	92.2	AM-S-Y1602	145	AM-S-Y1603	44.6
	PFUdA	µg/L	AM-S-Y1601	2.02	AM-S-Y1602	3.56	AM-S-Y1603	0.378
	Total Cholesterol	g/L	AM-S-W1601	1.93	AM-S-W1602	1.92	AM-S-W1603	1.67
	Total lipids	g/L	AM-S-W1601	5.62	AM-S-W1602	5.54	AM-S-W1603	5.32
	Toxaphene Parlar # 26	µg/L	AM-S-W1601	0.351	AM-S-W1602	0.141	AM-S-W1603	0.0709
	Toxaphene Parlar # 50	µg/L	AM-S-W1601	0.167	AM-S-W1602	0.0879	AM-S-W1603	0.390
	trans-nonachlor	µg/L	AM-S-W1601	2.75	AM-S-W1602	1.62	AM-S-W1603	3.65
	Triglycerides	g/L	AM-S-W1601	1.04	AM-S-W1602	1.06	AM-S-W1603	1.11

Centre de toxicologie/INSPQ
945, Ave Wolfe
Québec, QC
Canada, G1V 5B3
Phone: (418) 650-5115 Ext. 5254
Fax : (418) 654-2148
E-mail : ctq@inspq.qc.ca
http://www.inspq.qc.ca/ctq

GROUPING OF ANALYTICAL METHODS FOR STATISTICS

METHODS GROUPING CODE	METHODS GROUPING	METHODS INCLUDED	METHODS CODE
GC	GC	GC-ECD	GC-ECD
		GC-FID	GC-FID
GC-MS EI	GC-MS EI	GC-HRMS	GC-HRMS
		GC-MS EI	GC-MS EI
GC-MS CI	GC-MS CI	GC-MS NCI	GC-MS NCI
GC-MS-MS	GC-MS-MS	GC-MS-MS EI	GC-MS-MS EI
		GC-MS-MS NCI	GC-MS-MS NCI
LC-MS	LC-MS	LC-MS APCI	LC-MS APCI
		LC-MS ESI	LC-MS ESI
LC-MS-MS	LC-MS-MS	LC-MS-MS APCI	LC-MS-MS APCI
		LC-MS-MS ESI	LC-MS-MS ESI

1. Kolmogorov-Smirnov (Lilliefors) test

The Kolmogorov-Smirnov (Lilliefors) statistical test is a tool used to evaluate the normality of a distribution of results. The test can be performed with N ≥ 4.

The power of the test increases rapidly with the number of observations. For N ≥ 30, the test will be sensitive to account for small deviations from normality even with the presence of only one outlier.

For a small sample size (N ≤ 10), the hypothesis that the observations follow a normal distribution will generally be accepted. However, if normality is rejected for N ≤ 10, this indicates that the observations are far from following a normal distribution and that one or more observations are most likely outliers.

When the normality hypothesis is rejected, the use of robust methods for the determination of the AV by consensus such as the Algorithm A allows to obtain a better estimation of the AV. Outliers are retained except if their deviation from the other observations can be clearly explained as indicated in section 1.1.

The normality hypothesis is rejected when the statistical value is too weak according to the Kolmogorov-Smirnov (Lilliefors) critical value table. The result of the normality of the distribution of each set of observations is provided in the report whether the test is "Accepted" or "Rejected" under the column labeled "KS (Lilliefors)".

***** END OF REPORT *****

Institut national de santé publique Québec

Centre de toxicologie

AMAP RING TEST FOR PERSISTENT ORGANIC POLLUTANTS IN HUMAN SERUM (AMAP)

FINAL REPORT

**REPORT FOR ROUND:**	**2017-02**
**PTMS SHIPPING DATE:**	**2017-05-01**
**DATE OF PUBLICATION:**	**2017-07-14**

SCC Accredited PTP

PSEA Accrédité CCN TM

PCB IUPAC # 52, PCB IUPAC # 101, PCB IUPAC # 183, PCB IUPAC # 187, TOTAL CHOLESTEROL, TOTAL LIPIDS AND TRIGLYCERIDES ARE NOT INCLUDED IN THE SCOPE OF OUR ACCREDITATION.

THE ASSIGNED VALUE OBTAINED FOR PCB IUPAC # 74 IN MATERIAL AM-S-W1705 EXCEEDS THE RANGE OF CONCENTRATION FROM OUR SCOPE OF ACCREDITATION.

Centre de toxicologie/INSPQ

945, Wolfe Avenue
Quebec, QC
Canada, G1V 5B3

Phone: (418) 650-5115, ext. 5254
Fax: (418) 654-2148
E-mail: ctq@inspq.qc.ca
http://www.inspq.qc.ca/ctq

TABLE OF CONTENTS

INTRODUCTION 4

SERUM

Dieldrin 5

ß-HCH 8

Heptachlor 11

Heptachlor epoxide 14

Hexachlorobenzene 17

Oxychlordane 20

p,p'-DDE 23

p,p'-DDT 26

PBDE IUPAC # 28 29

PBDE IUPAC # 47 32

PBDE IUPAC # 99 35

PBDE IUPAC # 100 38

PBDE IUPAC # 153 41

PBDE IUPAC # 154 44

PBDE IUPAC # 183 47

PBDE IUPAC # 209 50

PCB IUPAC # 28 53

PCB IUPAC # 52 56

PCB IUPAC # 74 59

PCB IUPAC # 99 62

PCB IUPAC # 101 65

PCB IUPAC # 105 68

PCB IUPAC # 118 71

PCB IUPAC # 138 74

PCB IUPAC # 153 77

PCB IUPAC # 170 80
PCB IUPAC # 180 83
PCB IUPAC # 183 86
PCB IUPAC # 187 89
PFHxA 92
PFHxS 95
PFNA 98
PFOA 101
PFOS 104
PFUdA 107
Total Cholesterol 110
Total lipids 113
Toxaphene Parlar # 26 116
Toxaphene Parlar # 50 119
trans-nonachlor 122
Triglycerides 125

ASSIGNED VALUES 128

GROUPING OF ANALYTICAL METHODS FOR STATISTICS 130

END OF REPORT 131

INTRODUCTION

Dear AMAP participants:

This report includes the results and performance evaluations of round 2017-02.

Participating laboratories are identified only by their unique subscription number. Identity of participants will be kept strictly confidential by the AMAP organizer.

For this PT exercise, all analytes meet the homogeneity criteria as per ISO/CEI 17043 and 13528 guidelines with the exception of toxaphene parlar #26 and toxaphene parlar #50 in material AM-S-W1706. A note regarding homogeneity has been included in the corresponding sections.

A study had been previously performed to demonstrate that all the analytes are stable for the duration of the PT exercise and meet the stability criteria according to ISO/CEI 17043 and 13528 guidelines. This study is currently being updated.

We are available to assist you at any time. If you have any questions or concerns regarding our program, do not hesitate to contact us. Your comments help us enhance the quality of our schemes.

Best regards,

David Bisson, M.Sc. Chemist
Program Coordinators
External Quality Assessment Schemes and Reference Materials

Centre de toxicologie du Québec / INSPQ
Tel.: (418) 650-5115, extension 4649
E-mail: david.bisson@inspq.qc.ca
Web Site: www.inspq.qc.ca/CTQ/paqe

Individual results

Serum Dieldrin (µg/L)

Round #2017-02

Participant	AM-S-W1704	z'-score	AM-S-W1705	z'-score	AM-S-W1706	z'-score	Method
**1042**	0.135	0.23	0.681	-0.06	1.49	-0.45	GC-MS CI
**1629**	0.164	1.96	0.870	**2.18**	1.57	-0.05	GC-MS-MS
**2446**	0.125	-0.36	0.639	-0.56	1.48	-0.50	GC-MS EI
**4336**	<LD	---	0.666	-0.24	1.96	1.86	ND
**7828**	0.124	-0.42	0.660	-0.31	1.62	0.21	GC-MS EI
**8420**	<LQ	---	0.720	0.40	1.74	0.78	ND
**8559**	<LD	---	0.703	0.20	1.31	-1.31	GC
**9426**	<LD	---	---	---	---	---	GC-MS-MS

	Assigned value	Standard uncertainty	σ pt	Acceptable range	K-S (Lilliefors)	Species
**AM-S-W1704**	0.131	0.00609	0.0158	0.0972 - 0.165	Accepted	---
**AM-S-W1705**	0.686	0.0179	0.0823	0.518 - 0.854	Accepted	---
**AM-S-W1706**	1.58	0.0796	0.189	1.17 - 1.99	Accepted	---

Statistics
Serum Dieldrin (µg/L)

All methods	AM-S-W1704	AM-S-W1705	AM-S-W1706
N	4	7	7
Robust mean Algo A	0.131	0.686	1.58
Robust STDev	0.00975	0.0378	0.168
Median	0.130	0.681	1.57
STDev from MAD	0.00808	0.0326	0.138
Arithmetic mean	0.137	0.706	1.60
STDev	0.0187	0.0774	0.210
CV or Variability	7.4%	5.5%	10.7%

When fewer than 20 results were considered for statistical treatment of all or a sub-sample of results, the accuracy of statistical data may be questionable.

Distribution

Serum Dieldrin (µg/L)

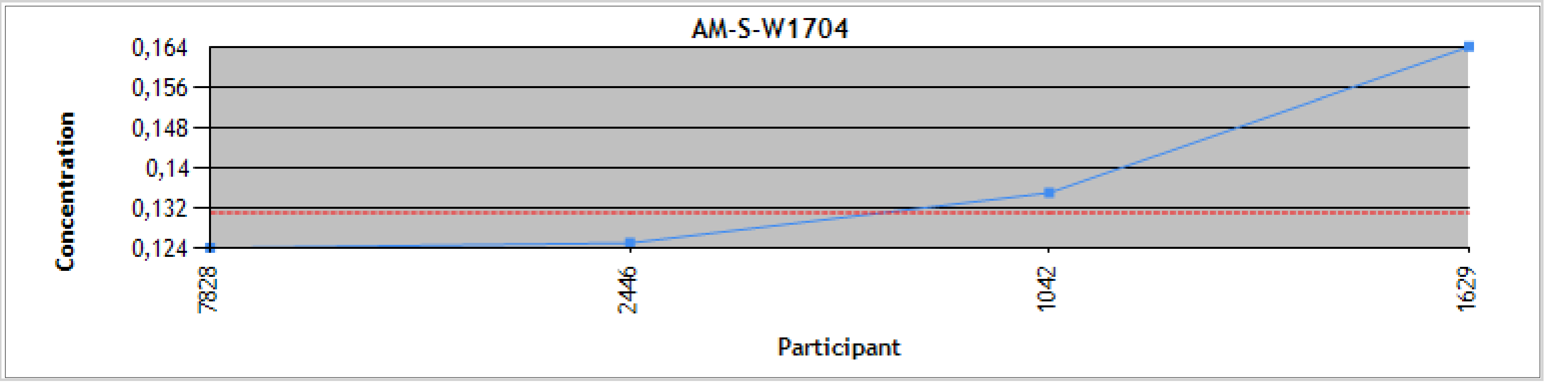

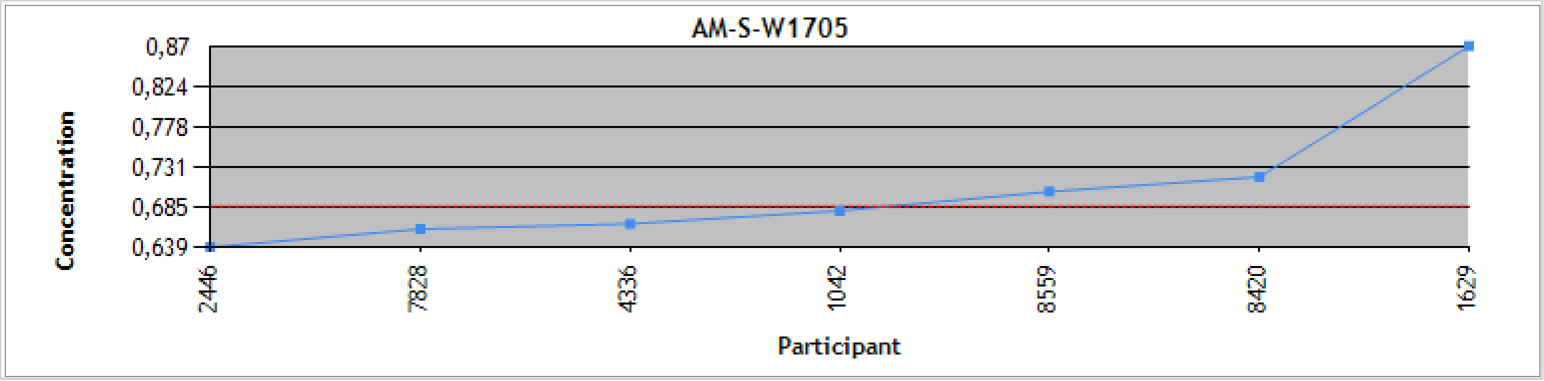

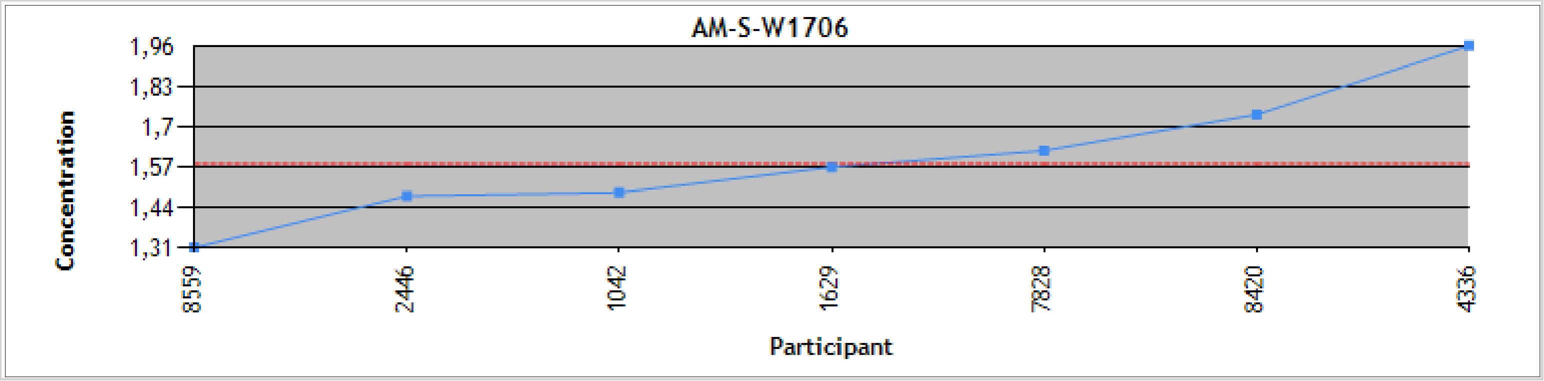

Individual results

Serum ß-HCH (µg/L)

Round #2017-02

Participant	AM-S-W1704	z'-score	AM-S-W1705	z'-score	AM-S-W1706	z'-score	Method
**748**	0.266	0.00	0.831	-0.85	0.535	0.01	ND
**1003**	0.260	-0.13	0.973	-0.02	0.543	0.10	GC-MS-MS
**1042**	0.262	-0.08	1.01	0.17	0.546	0.13	GC-MS CI
**1629**	0.446	3.82	0.921	-0.32	0.541	0.08	GC-MS-MS
**2446**	0.274	0.17	1.01	0.17	0.575	0.44	GC-MS EI
**3112**	0.258	-0.17	0.945	-0.19	0.516	-0.19	GC-MS CI
**4336**	0.182	-1.78	1.14	0.94	0.501	-0.36	ND
**4635**	0.197	-1.46	0.801	-1.02	0.467	-0.72	GC-MS EI
**6865**	0.309	0.91	1.18	1.18	0.553	0.20	GC
**7660**	0.265	-0.02	1.03	0.32	0.580	0.50	GC-MS CI
**7828**	0.281	0.32	1.07	0.56	0.576	0.45	GC-MS EI
**8420**	0.250	-0.34	0.880	-0.56	0.530	-0.04	ND
**8559**	0.258	-0.17	0.901	-0.44	0.416	-1.27	ND
**8916**	0.308	0.89	1.22	1.42	0.651	1.26	GC-MS EI
**9301**	0.220	-0.98	0.786	-1.11	0.440	-1.01	GC-MS-MS
**9426**	1.30	21.92	<LD	---	---	---	GC-MS-MS

	Assigned value	Standard uncertainty	σ pt	Acceptable range	K-S (Lilliefors)	Species
**AM-S-W1704**	0.266	0.00864	0.0464	0.172 - 0.360	Rejected[1]	---
**AM-S-W1705**	0.977	0.0470	0.166	0.632 - 1.32	Accepted	---
**AM-S-W1706**	0.534	0.0161	0.0915	0.348 - 0.720	Accepted	---

Statistics

Serum ß-HCH (µg/L)

All methods	AM-S-W1704	AM-S-W1705	AM-S-W1706
N	16	15	15
Robust mean Algo A	0.266	0.977	0.534
Robust STDev	0.0277	0.146	0.0499
Median	0.264	0.973	0.541
STDev from MAD	0.0230	0.138	0.0504
Arithmetic mean	0.334	0.980	0.531
STDev	0.264	0.134	0.0589
CV or Variability	10.4%	14.9%	9.4%

GC-MS CI	AM-S-W1704	AM-S-W1705	AM-S-W1706
N	3	3	3
Robust mean Algo A	0.262	0.997	0.547
Robust STDev	0.00400	0.0468	0.0365
Median	0.262	1.01	0.546
STDev from MAD	0.00400	0.0371	0.0445
Arithmetic mean	0.262	0.995	0.547
STDev	0.00353	0.0448	0.0322
CV or Variability	1.5%	4.7%	6.7%

GC-MS EI	AM-S-W1704	AM-S-W1705	AM-S-W1706
N	4	4	4
Robust mean Algo A	0.276	1.03	0.573
Robust STDev	0.0319	0.199	0.0741
Median	0.278	1.04	0.576
STDev from MAD	0.0252	0.161	0.0564
Arithmetic mean	0.265	1.03	0.567
STDev	0.0476	0.175	0.0757
CV or Variability	11.6%	19.4%	12.9%

GC-MS-MS	AM-S-W1704	AM-S-W1705	AM-S-W1706
N	4	3	3
Robust mean Algo A	0.383	0.900	0.540
Robust STDev	0.201	0.0973	0.00374
Median	0.353	0.921	0.541
STDev from MAD	0.168	0.0771	0.00297
Arithmetic mean	0.557	0.893	0.508
STDev	0.505	0.0965	0.0589
CV or Variability	52.7%	10.8%	0.7%

When fewer than 20 results were considered for statistical treatment of all or a sub-sample of results, the accuracy of statistical data may be questionable.

Distribution

Serum ß-HCH (µg/L)

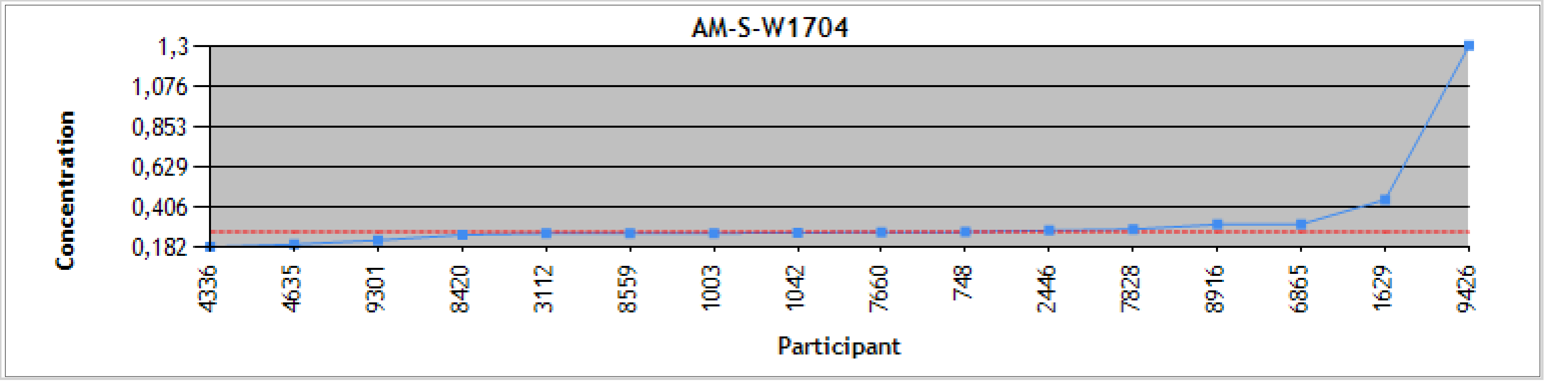

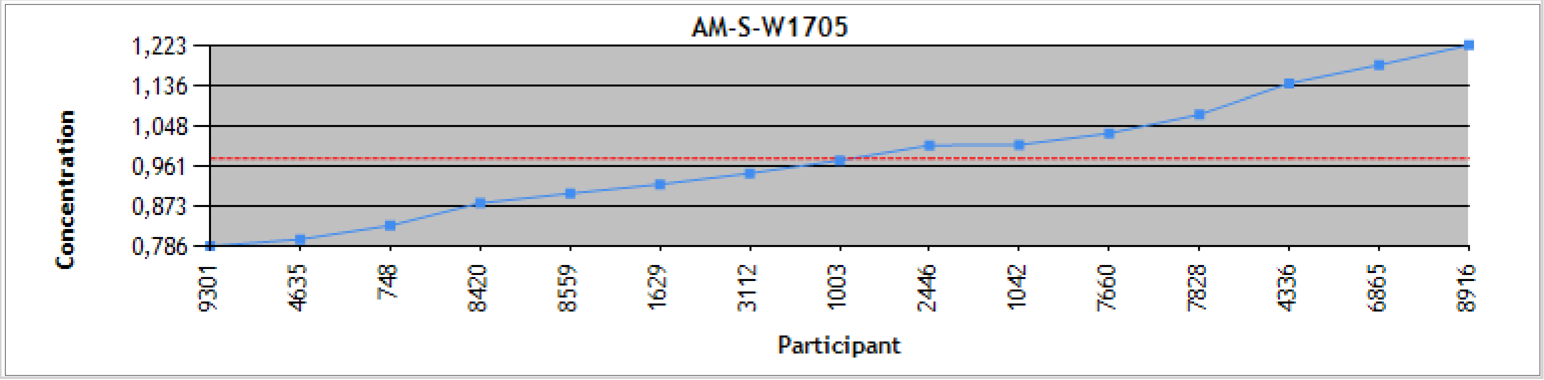

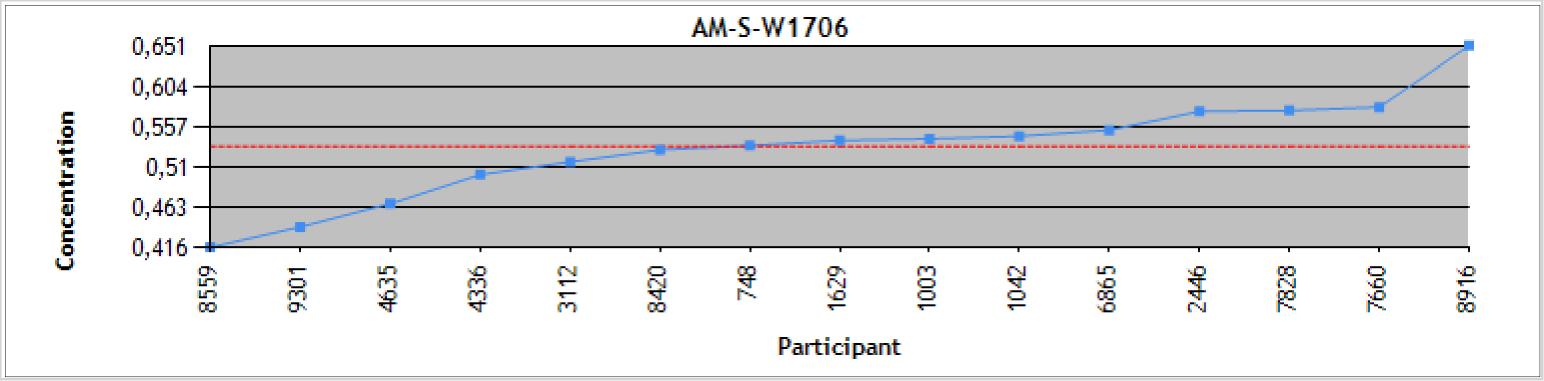

Individual results

Serum Heptachlor (µg/L)

Round #2017-02

Participant	AM-S-W1704	z'-score	AM-S-W1705	z'-score	AM-S-W1706	z'-score	Method
**1042**	0.0771	0.23	0.710	0.53	0.531	0.68	GC-MS CI
**1629**	0.152	**4.27**	1.23	**4.09**	1.25	**7.32**	GC-MS-MS
**2446**	0.0630	-0.53	0.647	0.10	0.463	0.06	GC-MS EI
**3112**	0.0660	-0.37	0.506	-0.87	0.343	-1.05	GC-MS CI
**4336**	0.0220	**-2.75**	0.268	**-2.50**	0.240	-2.00	ND
**7828**	0.0880	0.82	0.717	0.58	0.523	0.61	GC-MS EI
**8559**	<LD	---	0.560	-0.50	0.413	-0.41	ND
**9426**	<LD	---	<LD	---	<LD	---	GC-MS-MS

	Assigned value	Standard uncertainty	σ pt	Acceptable range	K-S (Lilliefors)	Species
**AM-S-W1704**	0.0729	0.0114	0.0146	0.0359 - 0.110	Accepted	---
**AM-S-W1705**	0.633	0.0725	0.127	0.341 - 0.925	Accepted	---
**AM-S-W1706**	0.457	0.0581	0.0914	0.240 - 0.674	Rejected[1]	---

Statistics

Serum Heptachlor (µg/L)

All methods	AM-S-W1704	AM-S-W1705	AM-S-W1706
N	6	7	7
Robust mean Algo A	0.0729	0.633	0.457
Robust STDev	0.0223	0.154	0.123
Median	0.0716	0.647	0.463
STDev from MAD	0.0185	0.129	0.101
Arithmetic mean	0.0780	0.663	0.538
STDev	0.0426	0.294	0.330
CV or Variability	30.7%	24.2%	26.9%

When fewer than 20 results were considered for statistical treatment of all or a sub-sample of results, the accuracy of statistical data may be questionable.

Distribution

Serum Heptachlor (µg/L)

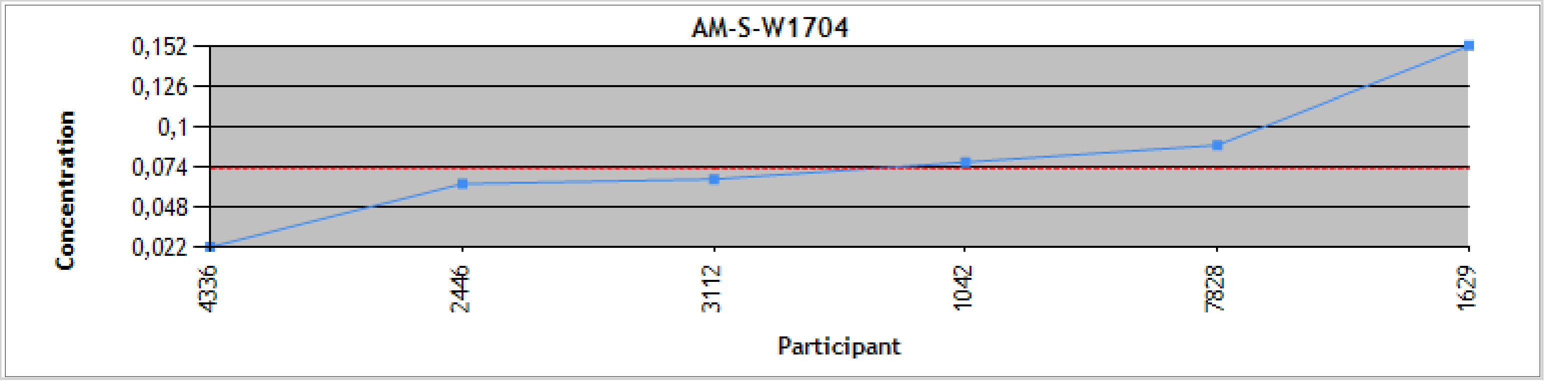

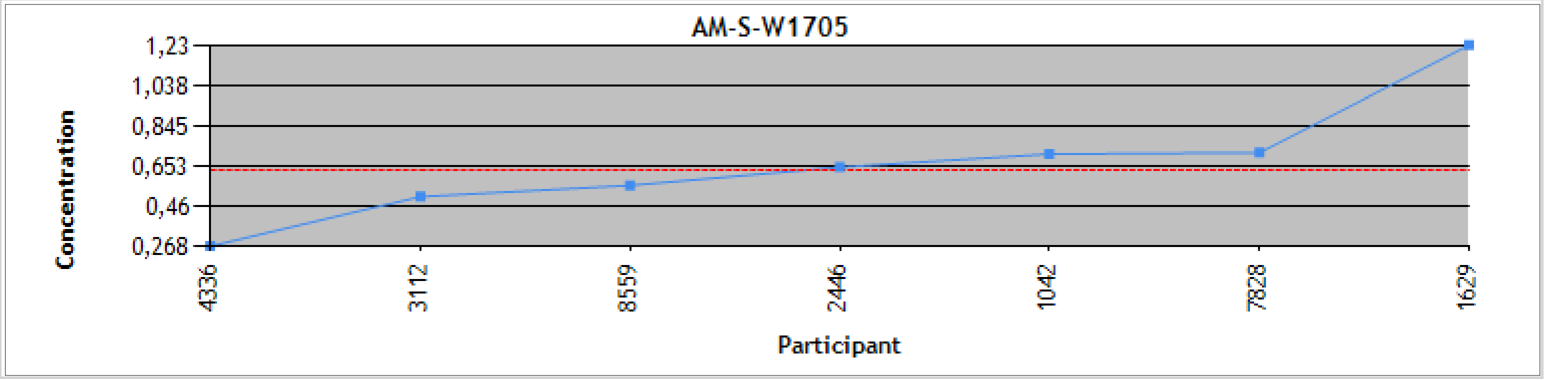

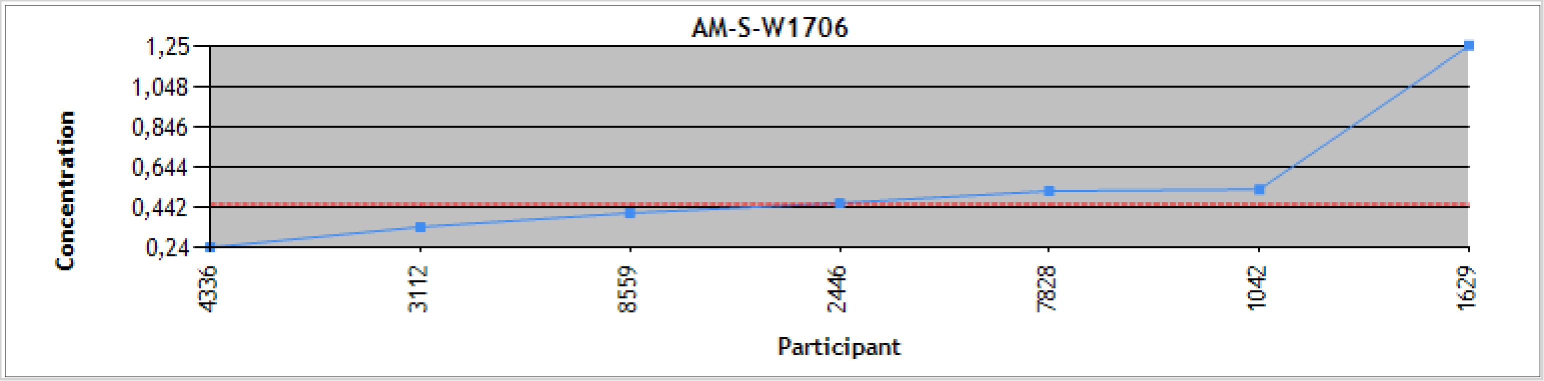

Individual results

Serum Heptachlor epoxide (µg/L)

Round #2017-02

Participant	AM-S-W1704	z'-score	AM-S-W1705	z'-score	AM-S-W1706	z'-score	Method
**1042**	0.113	0.43	1.21	0.12	0.678	0.02	GC-MS CI
**1629**	<LD	---	***8.03***	37.21	---	---	GC-MS-MS
**2446**	0.101	-0.22	1.08	-0.58	0.641	-0.33	GC-MS EI
**4336**	0.0850	-1.08	1.15	-0.24	0.679	0.03	ND
**7828**	0.102	-0.16	1.25	0.30	0.698	0.21	GC-MS EI
**8420**	0.210	5.67	1.46	1.47	0.750	0.71	ND
**8559**	<LD	---	1.15	-0.22	0.471	-1.96	ND
**9426**	<LD	---	<LD	---	<LD	---	GC-MS-MS

	Assigned value	Standard uncertainty	σ pt	Acceptable range	K-S (Lilliefors)	Species
**AM-S-W1704**	0.105	0.00976	0.0158	0.0678 - 0.142	Rejected[1]	---
**AM-S-W1705**	1.19	0.0438	0.178	0.823 - 1.56	Accepted	---
**AM-S-W1706**	0.676	0.0256	0.101	0.467 - 0.885	Accepted	---

Statistics
Serum Heptachlor epoxide (µg/L)

All methods	AM-S-W1704	AM-S-W1705	AM-S-W1706
**N**	5	6	6
**Robust mean Algo A**	0.105	1.19	0.676
**Robust STDev**	0.0175	0.0859	0.0501
**Median**	0.102	1.18	0.679
**STDev from MAD**	0.0163	0.0734	0.0423
**Arithmetic mean**	0.122	1.22	0.653
**STDev**	0.0501	0.132	0.0959
**CV or Variability**	16.6%	7.2%	7.4%

When fewer than 20 results were considered for statistical treatment of all or a sub-sample of results, the accuracy of statistical data may be questionable.

Distribution

Serum Heptachlor epoxide (µg/L)

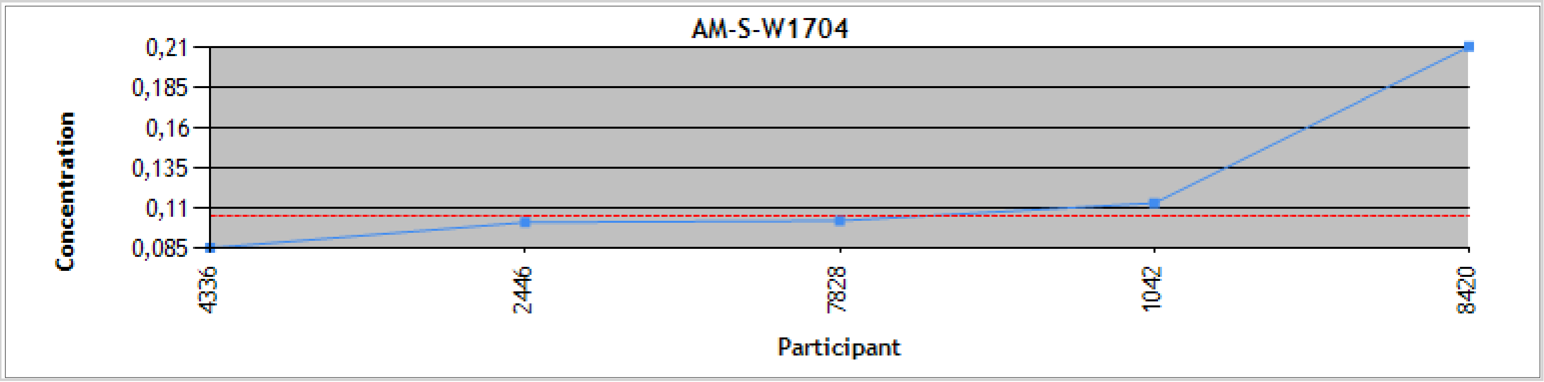

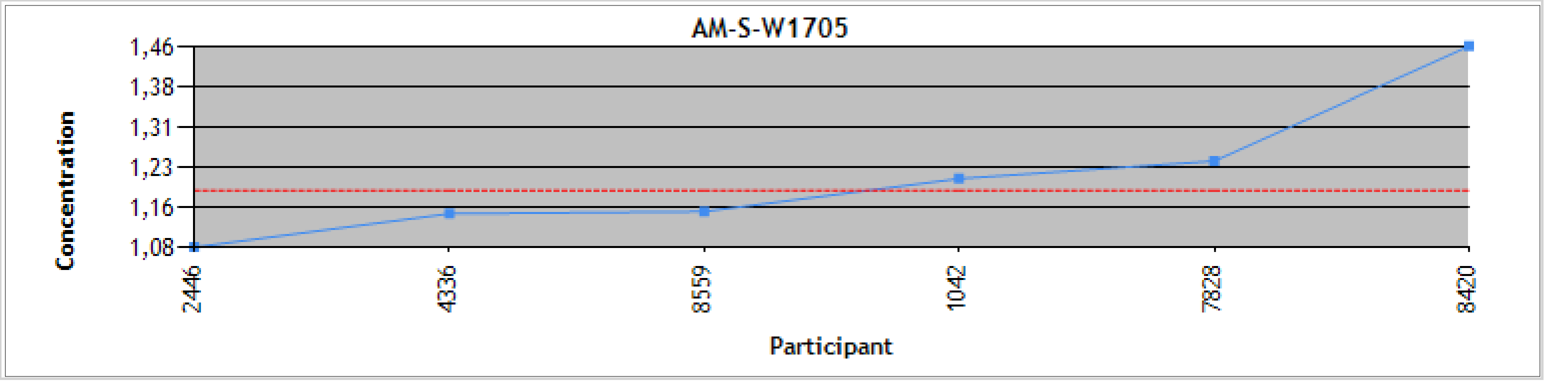

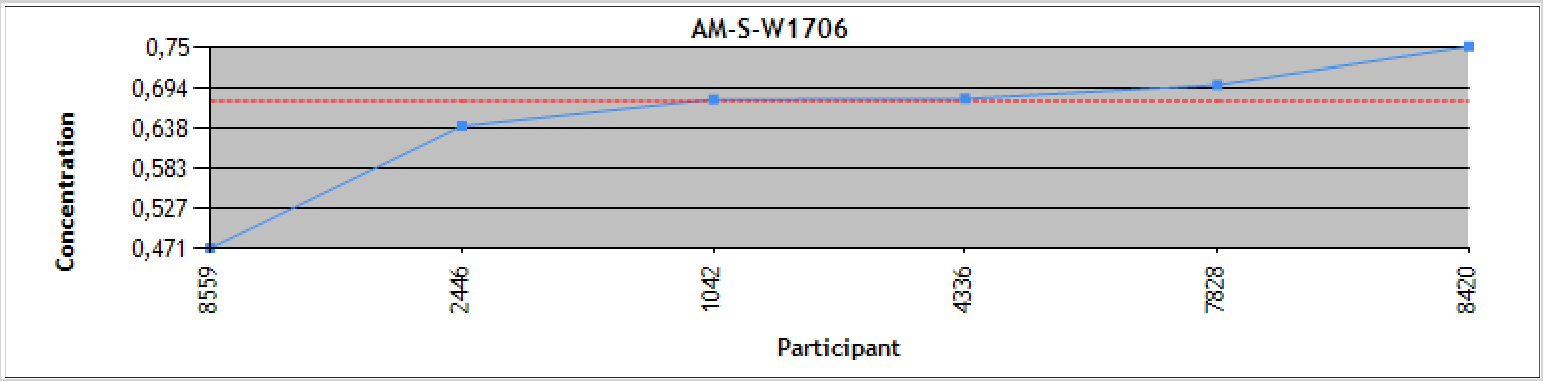

Individual results

Serum Hexachlorobenzene (µg/L)

Round #2017-02

Participant	AM-S-W1704	z'-score	AM-S-W1705	z'-score	AM-S-W1706	z'-score	Method
**748**	0.281	-0.60	0.936	-1.45	0.713	-0.27	ND
**1003**	0.327	0.11	1.30	0.13	0.760	0.07	GC-MS-MS
**1042**	0.343	0.36	1.36	0.39	0.788	0.28	GC-MS CI
**1629**	0.410	1.39	1.37	0.44	0.782	0.24	GC-MS-MS
**2446**	0.317	-0.05	1.20	-0.30	0.726	-0.18	GC-MS EI
**3112**	0.195	-1.93	0.768	**-2.19**	0.436	**-2.31**	GC-MS CI
**4336**	0.232	-1.36	0.194	**-4.68**	0.0580	**-5.10**	ND
**4635**	0.376	0.87	1.44	0.74	0.778	0.21	GC-MS EI
**6865**	0.378	0.90	1.57	1.30	0.897	1.08	GC
**7660**	0.341	0.33	1.38	0.49	0.794	0.32	GC-MS CI
**7828**	0.370	0.77	1.44	0.75	0.847	0.71	GC-MS EI
**8420**	0.380	0.93	1.40	0.57	0.850	0.74	ND
**8559**	0.254	-1.02	1.18	-0.39	0.510	-1.77	ND
**8916**	0.332	0.19	1.30	0.12	0.723	-0.20	GC-MS EI
**9301**	0.184	**-2.10**	0.693	**-2.51**	0.394	**-2.62**	GC-MS-MS

	Assigned value	Standard uncertainty	σ pt	Acceptable range	K-S (Lilliefors)	Species
**AM-S-W1704**	0.320	0.0223	0.0607	0.191 - 0.449	Accepted	---
**AM-S-W1705**	1.27	0.0644	0.221	0.810 - 1.73	Accepted	---
**AM-S-W1706**	0.750	0.0270	0.133	0.479 - 1.02	Accepted	---

Statistics
Serum Hexachlorobenzene (µg/L)

All methods	AM-S-W1704	AM-S-W1705	AM-S-W1706
N	15	15	15
Robust mean Algo A	0.320	1.27	0.750
Robust STDev	0.0690	0.200	0.0837
Median	0.332	1.30	0.760
STDev from MAD	0.0682	0.178	0.0697
Arithmetic mean	0.315	1.17	0.670
STDev	0.0703	0.369	0.226
CV or Variability	21.6%	15.7%	11.2%

GC-MS CI	AM-S-W1704	AM-S-W1705	AM-S-W1706
N	3	3	3
Robust mean Algo A	0.340	1.35	0.785
Robust STDev	0.00356	0.0430	0.0118
Median	0.341	1.36	0.788
STDev from MAD	0.00282	0.0341	0.00934
Arithmetic mean	0.293	1.17	0.673
STDev	0.0849	0.349	0.205
CV or Variability	1.0%	3.2%	1.5%

GC-MS EI	AM-S-W1704	AM-S-W1705	AM-S-W1706
N	4	4	4
Robust mean Algo A	0.349	1.35	0.760
Robust STDev	0.0326	0.130	0.0493
Median	0.351	1.37	0.752
STDev from MAD	0.0326	0.107	0.0408
Arithmetic mean	0.349	1.35	0.769
STDev	0.0288	0.117	0.0581
CV or Variability	9.4%	9.6%	6.5%

GC-MS-MS	AM-S-W1704	AM-S-W1705	AM-S-W1706
N	3	3	3
Robust mean Algo A	0.307	1.27	0.751
Robust STDev	0.130	0.131	0.0412
Median	0.327	1.30	0.760
STDev from MAD	0.123	0.104	0.0326
Arithmetic mean	0.307	1.12	0.645
STDev	0.114	0.372	0.218
CV or Variability	42.2%	10.3%	5.5%

When fewer than 20 results were considered for statistical treatment of all or a sub-sample of results, the accuracy of statistical data may be questionable.

Distribution

Serum Hexachlorobenzene (µg/L)

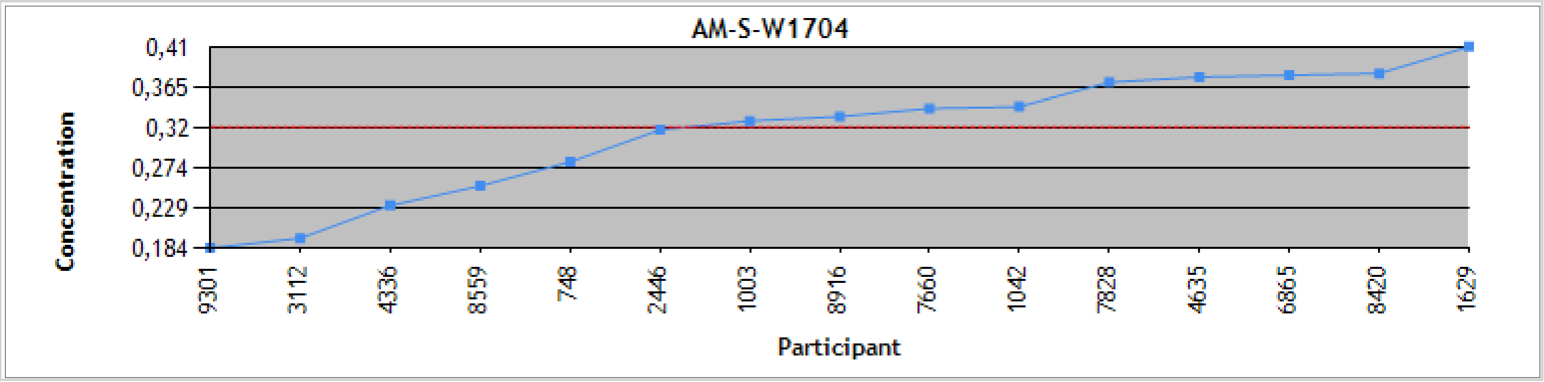

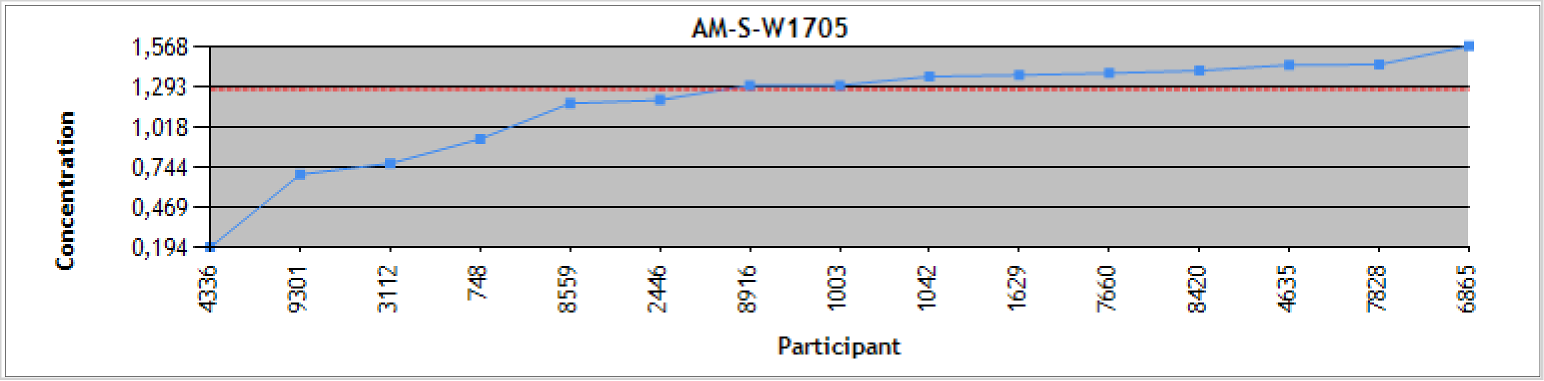

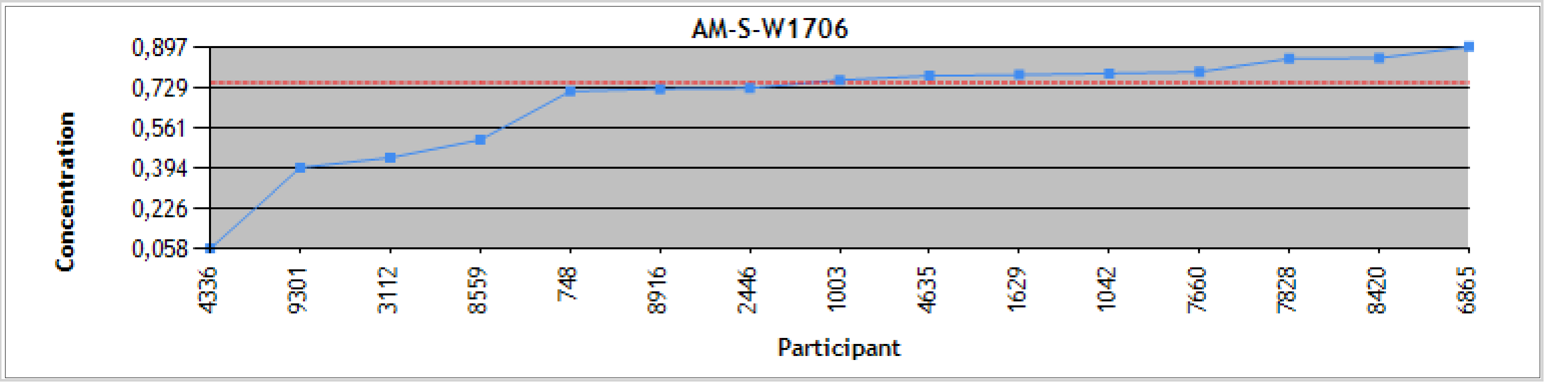

Individual results

Serum Oxychlordane (µg/L)

Round #2017-02

Participant	AM-S-W1704	z'-score	AM-S-W1705	z'-score	AM-S-W1706	z'-score	Method
**1003**	0.175	-0.64	0.761	0.09	1.56	0.21	GC-MS-MS
**1042**	0.216	0.42	0.796	0.40	1.62	0.51	GC-MS CI
**2446**	0.180	-0.52	0.676	-0.67	1.26	-1.35	GC-MS EI
**3112**	0.198	-0.05	0.662	-0.79	1.39	-0.69	GC-MS CI
**4635**	0.222	0.57	0.894	1.27	1.61	0.45	GC-MS EI
**6865**	0.299	**2.55**	0.953	1.80	1.59	0.37	GC
**7660**	0.193	-0.18	0.747	-0.04	1.48	-0.19	GC-MS CI
**7828**	0.228	0.72	0.932	1.61	1.84	1.66	GC-MS EI
**8420**	0.250	1.29	0.760	0.08	1.53	0.05	ND
**8559**	0.128	-1.85	0.397	**-3.15**	1.01	**-2.65**	ND
**9301**	0.108	**-2.37**	0.430	**-2.86**	0.785	**-3.81**	GC-MS-MS
**9426**	<LD	---	<LD	---	3.50	**10.27**	GC-MS-MS

	Assigned value	Standard uncertainty	σ pt	Acceptable range	K-S (Lilliefors)	Species
**AM-S-W1704**	0.200	0.0159	0.0354	0.122 - 0.278	Accepted	---
**AM-S-W1705**	0.751	0.0600	0.0949	0.526 - 0.976	Accepted	---
**AM-S-W1706**	1.52	0.0743	0.178	1.13 - 1.91	Rejected[1]	---

Statistics

Serum Oxychlordane (µg/L)

All methods	AM-S-W1704	AM-S-W1705	AM-S-W1706
N	11	11	12
Robust mean Algo A	0.200	0.751	1.52
Robust STDev	0.0423	0.159	0.206
Median	0.198	0.760	1.55
STDev from MAD	0.0356	0.145	0.171
Arithmetic mean	0.200	0.728	1.60
STDev	0.0534	0.182	0.665
CV or Variability	21.1%	21.2%	13.6%

GC-MS CI	AM-S-W1704	AM-S-W1705	AM-S-W1706
N	3	3	3
Robust mean Algo A	0.200	0.735	1.50
Robust STDev	0.00936	0.0767	0.132
Median	0.198	0.747	1.48
STDev from MAD	0.00742	0.0724	0.142
Arithmetic mean	0.202	0.735	1.50
STDev	0.0123	0.0677	0.116
CV or Variability	4.7%	10.4%	8.8%

GC-MS EI	AM-S-W1704	AM-S-W1705	AM-S-W1706
N	3	3	3
Robust mean Algo A	0.220	0.878	1.57
Robust STDev	0.0112	0.0711	0.331
Median	0.222	0.894	1.61
STDev from MAD	0.00890	0.0564	0.349
Arithmetic mean	0.210	0.834	1.57
STDev	0.0262	0.138	0.292
CV or Variability	5.1%	8.1%	21.1%

GC-MS-MS	AM-S-W1704	AM-S-W1705	AM-S-W1706
N	NA	NA	3
Robust mean Algo A	NA	NA	1.88
Robust STDev	NA	NA	1.45
Median	NA	NA	1.56
STDev from MAD	NA	NA	1.15
Arithmetic mean	NA	NA	1.95
STDev	NA	NA	1.40
CV or Variability	NA	NA	77.3%

When fewer than 20 results were considered for statistical treatment of all or a sub-sample of results, the accuracy of statistical data may be questionable.

Distribution

Serum Oxychlordane (µg/L)

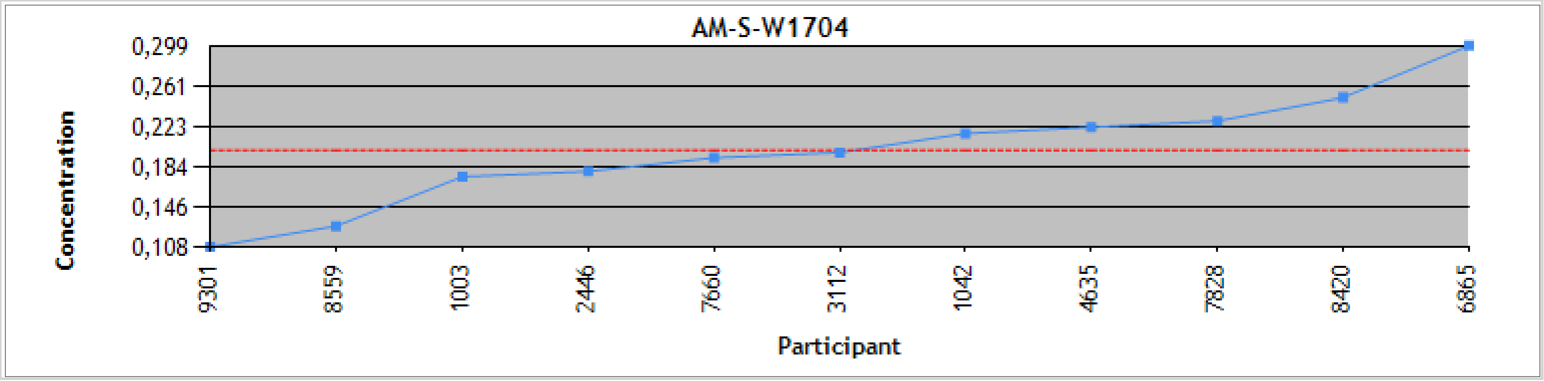

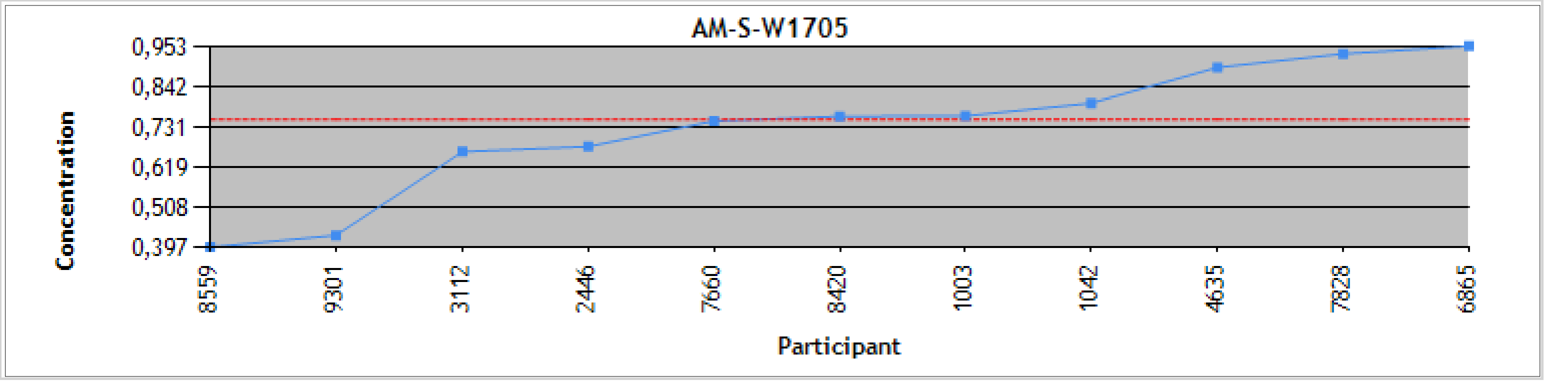

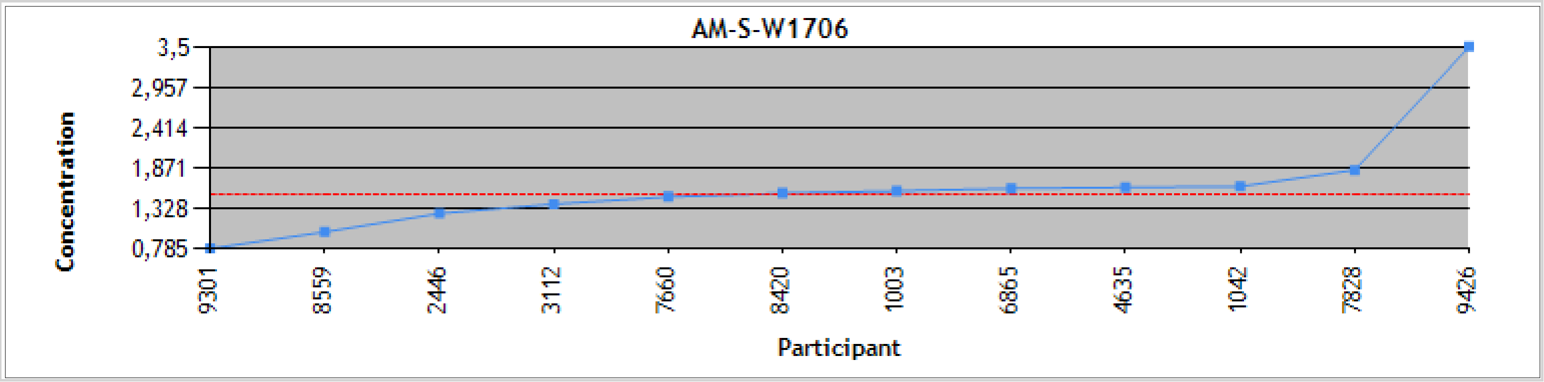

Individual results

Serum p,p'-DDE (µg/L)

Round #2017-02

Participant	AM-S-W1704	z'-score	AM-S-W1705	z'-score	AM-S-W1706	z'-score	Method
**748**	1.54	-0.16	3.87	-0.93	9.74	1.41	ND
**1003**	1.54	-0.16	4.38	0.17	8.39	-0.11	GC-MS-MS
**1042**	1.64	0.39	4.71	0.87	8.93	0.50	GC-MS CI
**1629**	1.21	-1.90	3.94	-0.78	7.86	-0.71	GC-MS-MS
**2446**	1.47	-0.54	3.88	-0.90	7.70	-0.89	GC-MS EI
**3112**	2.11	**2.84**	6.16	**4.02**	11.9	**3.87**	GC-MS CI
**4336**	1.46	-0.60	4.57	0.58	9.20	0.80	ND
**4635**	1.60	0.15	4.26	-0.08	8.40	-0.11	GC-MS EI
**6865**	1.69	0.63	4.43	0.28	9.01	0.59	GC
**7660**	1.65	0.42	4.78	1.03	8.95	0.52	GC-MS CI
**7828**	1.72	0.79	4.65	0.75	8.95	0.52	GC-MS EI
**8420**	1.46	-0.58	3.67	-1.36	7.32	-1.32	ND
**8559**	1.71	0.74	4.51	0.45	9.23	0.84	ND
**8916**	1.59	0.08	4.70	0.87	7.76	-0.83	GC-MS EI
**9301**	0.784	**-4.15**	2.24	**-4.44**	4.41	**-4.61**	GC-MS-MS
**9426**	<LD	---	2.50	**-3.88**	3.80	**-5.30**	GC-MS-MS

	Assigned value	Standard uncertainty	σ pt	Acceptable range	K-S (Lilliefors)	Species
**AM-S-W1704**	1.57	0.0521	0.182	1.19 - 1.95	Accepted	---
**AM-S-W1705**	4.30	0.162	0.435	3.37 - 5.23	Accepted	---
**AM-S-W1706**	8.49	0.325	0.823	6.72 - 10.3	Accepted	---

Statistics

Serum p,p'-DDE (µg/L)

All methods	AM-S-W1704	AM-S-W1705	AM-S-W1706
N	15	16	16
Robust mean Algo A	1.57	4.30	8.49
Robust STDev	0.161	0.517	1.04
Median	1.59	4.41	8.66
STDev from MAD	0.174	0.498	1.02
Arithmetic mean	1.54	4.20	8.22
STDev	0.284	0.913	1.93
CV or Variability	10.3%	12.0%	12.2%

GC-MS CI	AM-S-W1704	AM-S-W1705	AM-S-W1706
N	3	3	3
Robust mean Algo A	1.65	4.80	8.95
Robust STDev	0.00936	0.133	0.0243
Median	1.65	4.78	8.95
STDev from MAD	0.00742	0.105	0.0193
Arithmetic mean	1.80	5.22	9.93
STDev	0.266	0.823	1.72
CV or Variability	0.6%	2.8%	0.3%

GC-MS EI	AM-S-W1704	AM-S-W1705	AM-S-W1706
N	4	4	4
Robust mean Algo A	1.59	4.40	8.17
Robust STDev	0.116	0.393	0.619
Median	1.59	4.46	8.08
STDev from MAD	0.0971	0.326	0.512
Arithmetic mean	1.59	4.37	8.20
STDev	0.103	0.383	0.591
CV or Variability	7.3%	8.9%	7.6%

GC-MS-MS	AM-S-W1704	AM-S-W1705	AM-S-W1706
N	3	4	4
Robust mean Algo A	1.18	3.27	6.12
Robust STDev	0.430	1.20	2.66
Median	1.21	3.22	6.14
STDev from MAD	0.489	1.26	2.95
Arithmetic mean	1.18	3.27	6.12
STDev	0.379	1.05	2.34
CV or Variability	36.5%	36.6%	43.5%

When fewer than 20 results were considered for statistical treatment of all or a sub-sample of results, the accuracy of statistical data may be questionable.

Distribution

Serum p,p'-DDE (µg/L)

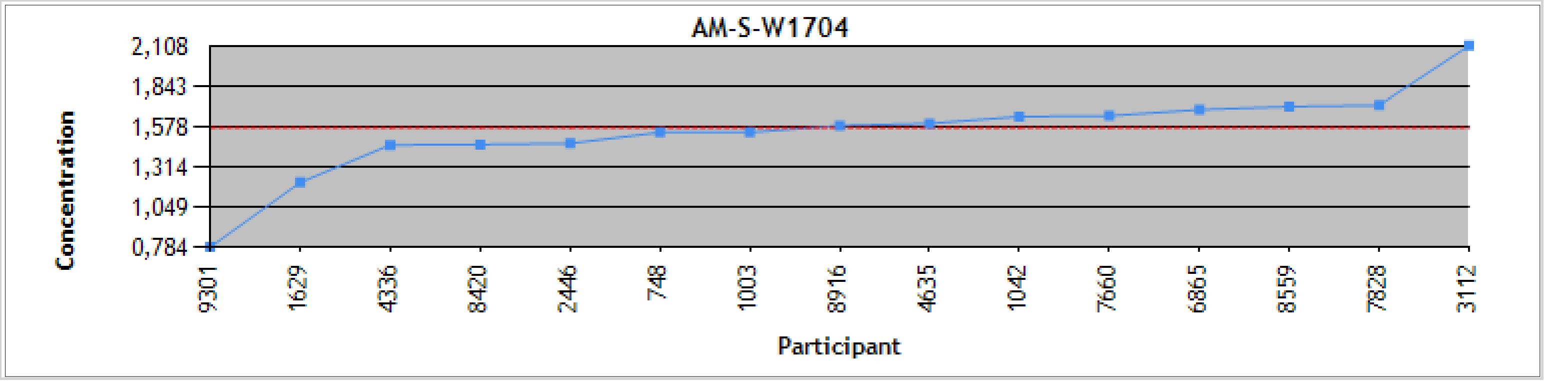

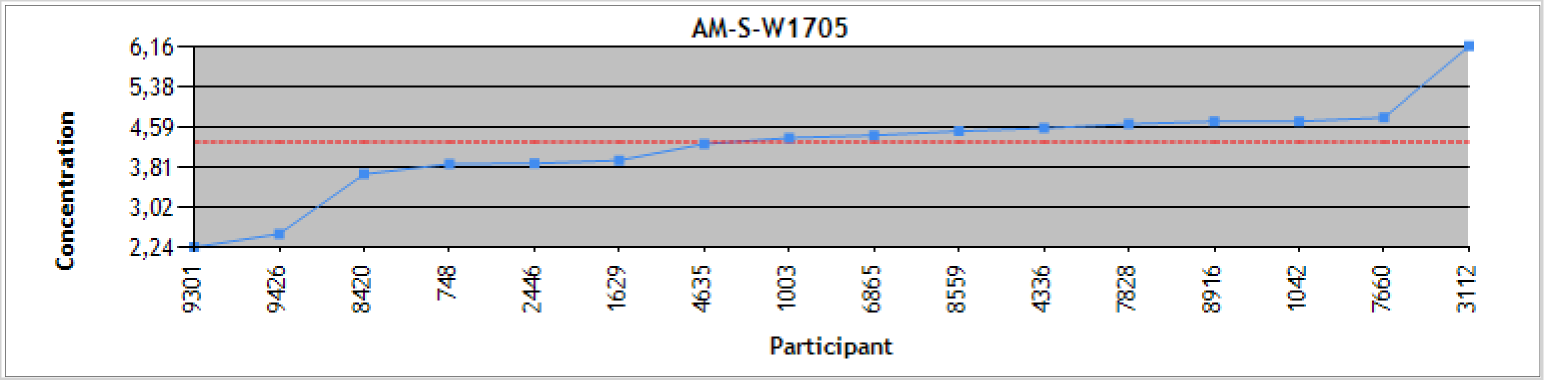

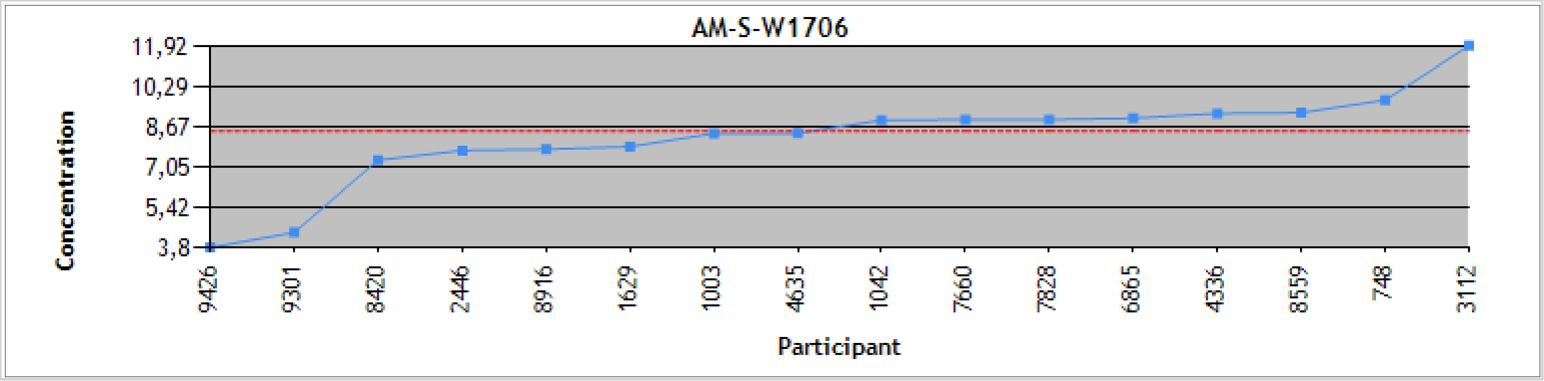

Individual results
Serum p,p'-DDT (µg/L)
Round #2017-02

Participant	AM-S-W1704	z'-score	AM-S-W1705	z'-score	AM-S-W1706	z'-score	Method
**748**	0.167	-0.60	0.381	-1.47	0.862	-0.57	ND
**1003**	0.207	0.20	0.582	0.27	0.985	0.09	GC-MS-MS
**1042**	0.195	-0.04	0.561	0.09	0.960	-0.04	GC-MS CI
**1629**	0.243	0.92	0.566	0.13	0.965	-0.02	GC-MS-MS
**2446**	0.177	-0.40	0.447	-0.90	0.873	-0.51	GC-MS EI
**3112**	0.335	**2.77**	0.912	**3.12**	1.60	**3.41**	GC-MS CI
**4336**	0.216	0.38	0.637	0.74	1.08	0.62	ND
**4635**	0.199	0.04	0.599	0.42	0.984	0.09	GC-MS EI
**6865**	0.231	0.68	0.634	0.72	1.01	0.21	GC
**7660**	0.211	0.29	0.642	0.79	1.10	0.71	GC-MS CI
**7828**	0.186	-0.22	0.565	0.12	0.935	-0.18	GC-MS EI
**8559**	0.126	-1.43	0.283	**-2.32**	1.00	0.17	ND
**8916**	0.184	-0.26	0.530	-0.18	0.846	-0.66	ND
**9301**	0.0795	**-2.36**	0.269	**-2.44**	0.483	**-2.62**	GC-MS-MS
**9426**	<LD	---	<LD	---	<LD	---	GC-MS-MS

	Assigned value	Standard uncertainty	σ pt	Acceptable range	K-S (Lilliefors)	Species
**AM-S-W1704**	0.197	0.0110	0.0486	0.0974 - 0.297	Accepted	---
**AM-S-W1705**	0.551	0.0372	0.109	0.320 - 0.782	Accepted	---
**AM-S-W1706**	0.968	0.0362	0.181	0.598 - 1.34	Rejected[1]	---

Statistics

Serum p,p'-DDT (µg/L)

All methods	AM-S-W1704	AM-S-W1705	AM-S-W1706
N	14	14	14
Robust mean Algo A	0.197	0.551	0.968
Robust STDev	0.0329	0.111	0.108
Median	0.197	0.566	0.975
STDev from MAD	0.0289	0.104	0.105
Arithmetic mean	0.197	0.543	0.977
STDev	0.0578	0.163	0.232
CV or Variability	16.7%	20.2%	11.2%

GC-MS CI	AM-S-W1704	AM-S-W1705	AM-S-W1706
N	3	3	3
Robust mean Algo A	0.218	0.676	1.16
Robust STDev	0.0309	0.152	0.263
Median	0.211	0.642	1.10
STDev from MAD	0.0245	0.121	0.208
Arithmetic mean	0.247	0.705	1.22
STDev	0.0766	0.184	0.336
CV or Variability	14.2%	22.6%	22.7%

GC-MS EI	AM-S-W1704	AM-S-W1705	AM-S-W1706
N	3	3	3
Robust mean Algo A	0.187	0.551	0.931
Robust STDev	0.0125	0.0636	0.0631
Median	0.186	0.565	0.935
STDev from MAD	0.0133	0.0504	0.0727
Arithmetic mean	0.187	0.537	0.931
STDev	0.0111	0.0798	0.0556
CV or Variability	6.7%	11.5%	6.8%

GC-MS-MS	AM-S-W1704	AM-S-W1705	AM-S-W1706
N	3	3	3
Robust mean Algo A	0.192	0.559	0.957
Robust STDev	0.0674	0.0299	0.0374
Median	0.207	0.566	0.965
STDev from MAD	0.0534	0.0237	0.0297
Arithmetic mean	0.177	0.472	0.811
STDev	0.0859	0.176	0.284
CV or Variability	35.0%	5.4%	3.9%

When fewer than 20 results were considered for statistical treatment of all or a sub-sample of results, the accuracy of statistical data may be questionable.

Distribution

Serum p,p'-DDT (µg/L)

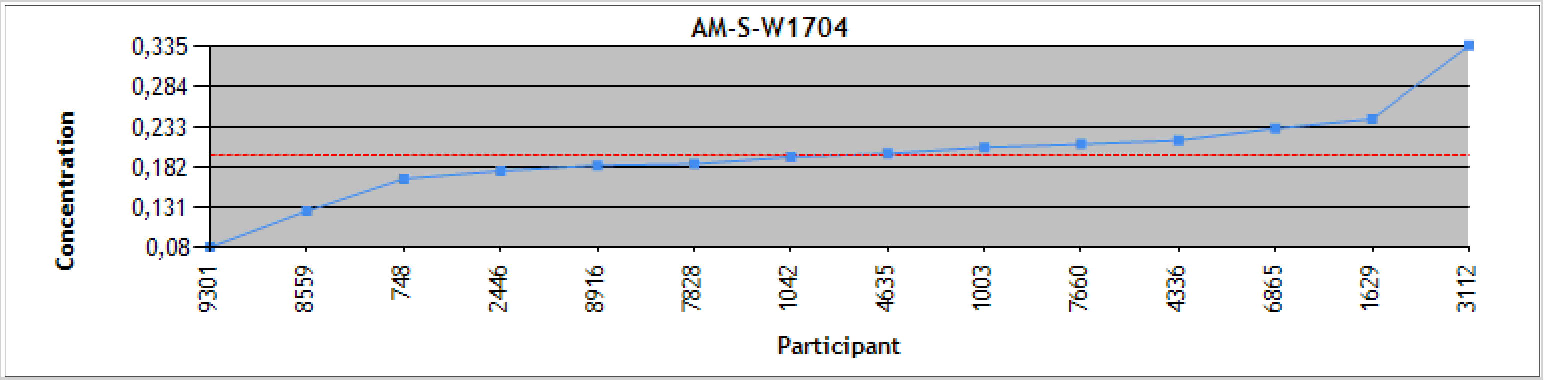

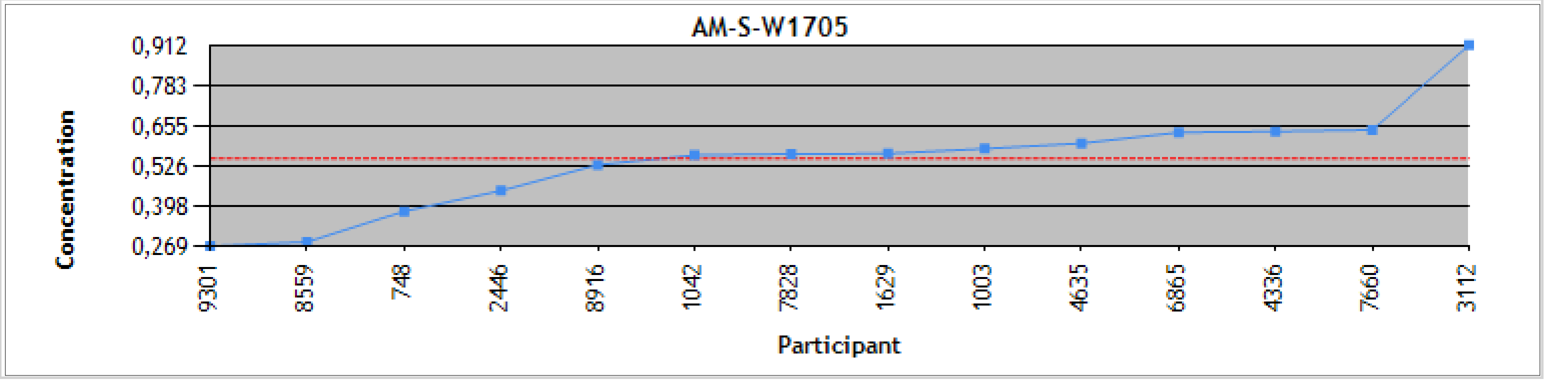

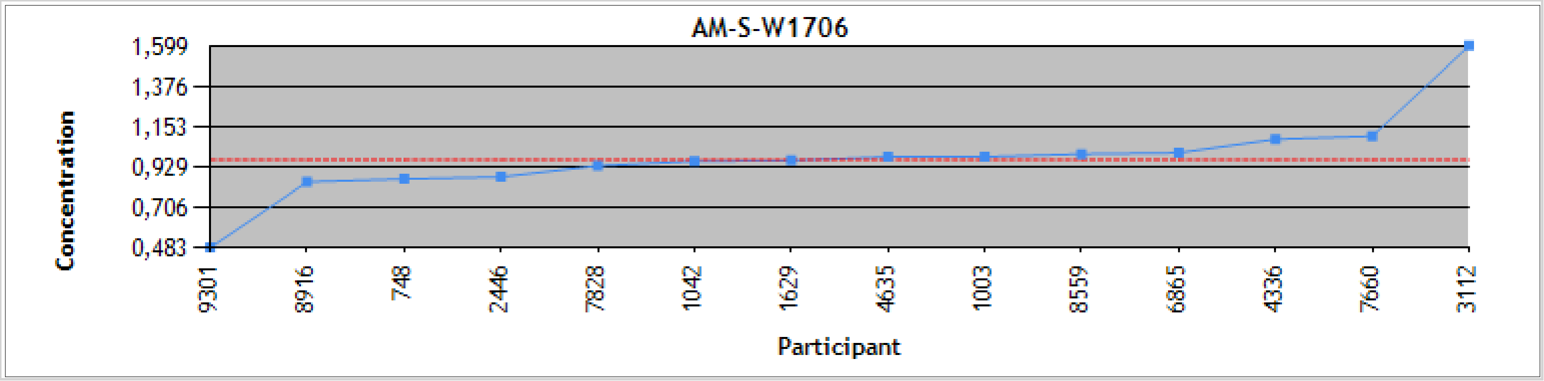

Individual results

Serum PBDE IUPAC # 28 (µg/L)

Round #2017-02

Participant	AM-S-W1704	z'-score	AM-S-W1705	z'-score	AM-S-W1706	z'-score	Method
**1042**	0.0998	0.35	0.459	0.57	0.230	0.08	GC-MS CI
**1629**	0.0850	-0.73	0.421	-0.13	0.230	0.06	GC-MS EI
**2446**	0.115	1.45	0.516	1.60	0.271	1.38	GC-MS EI
**3112**	0.0950	0.00	0.436	0.15	0.242	0.45	GC-MS CI
**4635**	0.0970	0.15	0.440	0.22	0.236	0.26	GC-MS EI
**4728**	0.0890	-0.44	0.405	-0.42	0.207	-0.68	GC-MS-MS
**7336**	0.154	**4.28**	0.513	1.54	0.326	**3.16**	GC-MS CI
**7660**	0.0954	0.03	0.409	-0.35	0.217	-0.35	GC-MS CI
**8916**	0.0850	-0.73	0.355	-1.32	0.182	-1.48	GC-MS EI
**9301**	0.0495	**-3.30**	0.223	**-3.72**	0.117	**-3.57**	GC-MS EI

	Assigned value	Standard uncertainty	σ pt	Acceptable range	K-S (Lilliefors)	Species
**AM-S-W1704**	0.0950	0.00532	0.0127	0.0674 - 0.123	Accepted	---
**AM-S-W1705**	0.428	0.0193	0.0517	0.318 - 0.538	Accepted	---
**AM-S-W1706**	0.228	0.0129	0.0283	0.166 - 0.290	Accepted	---

Statistics

Serum PBDE IUPAC # 28 (µg/L)

All methods	AM-S-W1704	AM-S-W1705	AM-S-W1706
N	10	10	10
Robust mean Algo A	0.0950	0.428	0.228
Robust STDev	0.0135	0.0488	0.0325
Median	0.0952	0.429	0.230
STDev from MAD	0.0122	0.0402	0.0270
Arithmetic mean	0.0965	0.418	0.226
STDev	0.0262	0.0839	0.0544
CV or Variability	14.2%	11.4%	14.2%

GC-MS CI	AM-S-W1704	AM-S-W1705	AM-S-W1706
N	4	4	4
Robust mean Algo A	0.0983	0.452	0.238
Robust STDev	0.00430	0.0458	0.0226
Median	0.0976	0.448	0.236
STDev from MAD	0.00356	0.0376	0.0185
Arithmetic mean	0.111	0.454	0.254
STDev	0.0287	0.0444	0.0492
CV or Variability	4.4%	10.1%	9.5%

GC-MS EI	AM-S-W1704	AM-S-W1705	AM-S-W1706
N	5	5	5
Robust mean Algo A	0.0874	0.401	0.212
Robust STDev	0.0223	0.104	0.0585
Median	0.0850	0.421	0.230
STDev from MAD	0.0178	0.0979	0.0608
Arithmetic mean	0.0863	0.391	0.207
STDev	0.0240	0.110	0.0596
CV or Variability	25.5%	25.8%	27.6%

When fewer than 20 results were considered for statistical treatment of all or a sub-sample of results, the accuracy of statistical data may be questionable.

Distribution

Serum PBDE IUPAC # 28 (µg/L)

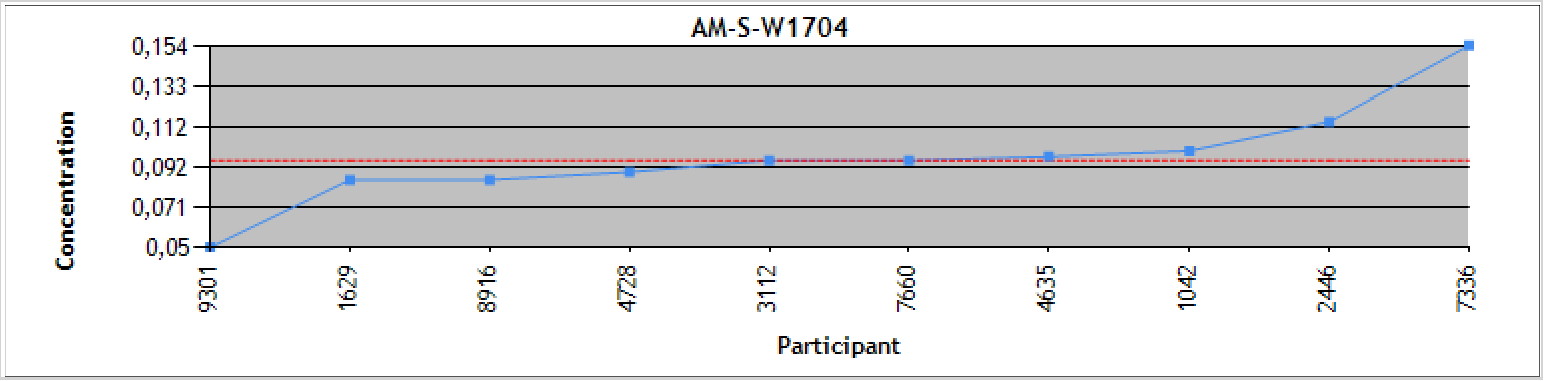

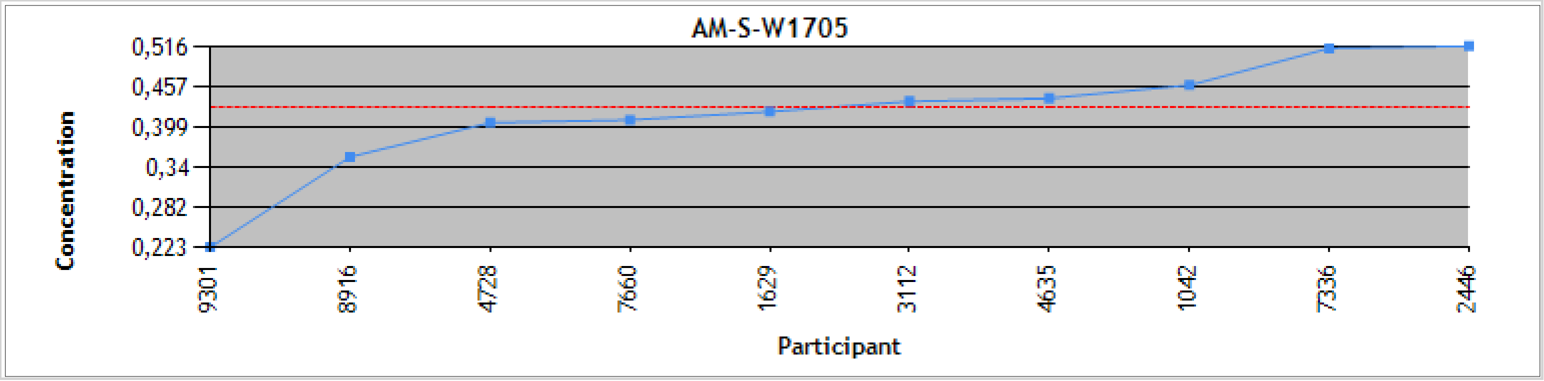

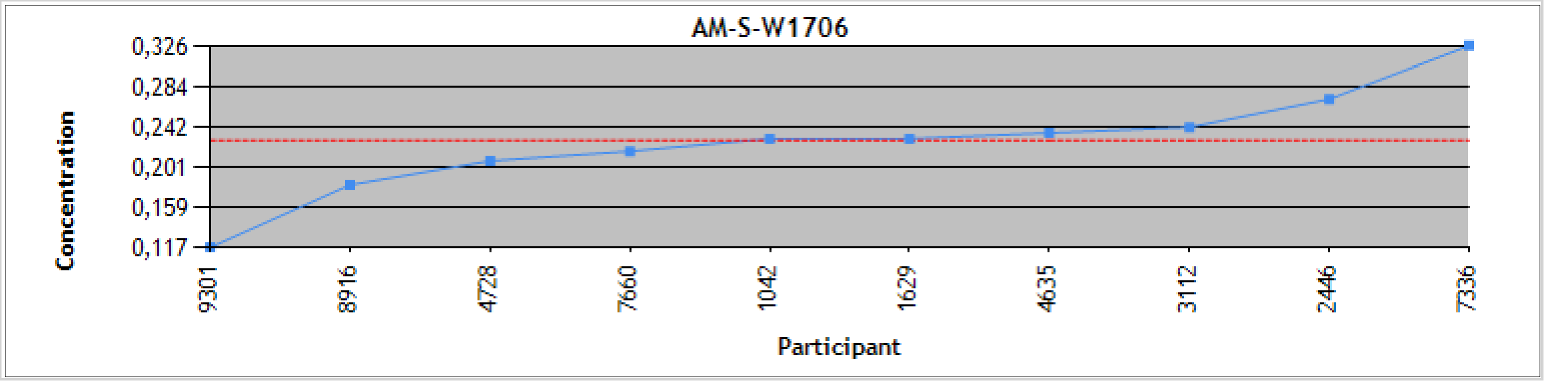

Individual results

Serum PBDE IUPAC # 47 (µg/L)

Round #2017-02

Participant	AM-S-W1704	z'-score	AM-S-W1705	z'-score	AM-S-W1706	z'-score	Method
**1003**	0.492	0.13	1.15	0.23	0.643	0.23	GC-MS-MS
**1042**	0.529	0.73	1.20	0.60	0.646	0.27	GC-MS CI
**1629**	0.473	-0.18	1.12	0.00	0.650	0.33	GC-MS EI
**2446**	0.490	0.10	1.07	-0.41	0.623	-0.03	GC-MS EI
**3112**	0.501	0.28	1.18	0.44	0.679	0.70	GC-MS CI
**4635**	0.531	0.76	1.17	0.37	0.675	0.65	GC-MS EI
**4728**	0.453	-0.50	1.05	-0.52	0.574	-0.67	GC-MS-MS
**6865**	0.414	-1.13	1.07	-0.39	0.565	-0.78	GC
**7336**	0.839	**5.75**	1.30	1.37	0.716	1.19	GC-MS CI
**7660**	0.516	0.52	1.15	0.21	0.632	0.09	GC-MS CI
**8916**	0.437	-0.76	0.991	-0.99	0.538	-1.14	GC-MS EI
**9301**	0.274	**-3.40**	0.608	**-3.93**	0.346	**-3.64**	GC-MS EI

	Assigned value	Standard uncertainty	σ pt	Acceptable range	K-S (Lilliefors)	Species
**AM-S-W1704**	0.484	0.0203	0.0584	0.360 - 0.608	Rejected[1]	---
**AM-S-W1705**	1.12	0.0339	0.125	0.860 - 1.38	Accepted	---
**AM-S-W1706**	0.625	0.0223	0.0733	0.472 - 0.778	Accepted	---

Statistics

Serum PBDE IUPAC # 47 (µg/L)

All methods	AM-S-W1704	AM-S-W1705	AM-S-W1706
N	12	12	12
Robust mean Algo A	0.484	1.12	0.625
Robust STDev	0.0561	0.0938	0.0619
Median	0.491	1.13	0.637
STDev from MAD	0.0563	0.0957	0.0587
Arithmetic mean	0.496	1.09	0.607
STDev	0.129	0.171	0.0967
CV or Variability	11.6%	8.4%	9.9%

GC-MS CI	AM-S-W1704	AM-S-W1705	AM-S-W1706
N	4	4	4
Robust mean Algo A	0.525	1.19	0.668
Robust STDev	0.0252	0.0455	0.0420
Median	0.522	1.19	0.662
STDev from MAD	0.0207	0.0371	0.0349
Arithmetic mean	0.596	1.21	0.668
STDev	0.162	0.0650	0.0375
CV or Variability	4.8%	3.8%	6.3%

GC-MS EI	AM-S-W1704	AM-S-W1705	AM-S-W1706
N	5	5	5
Robust mean Algo A	0.465	1.05	0.599
Robust STDev	0.0595	0.121	0.0823
Median	0.473	1.07	0.623
STDev from MAD	0.0534	0.113	0.0771
Arithmetic mean	0.441	0.991	0.566
STDev	0.0993	0.224	0.134
CV or Variability	12.8%	11.6%	13.8%

When fewer than 20 results were considered for statistical treatment of all or a sub-sample of results, the accuracy of statistical data may be questionable.

Distribution
Serum PBDE IUPAC # 47 (µg/L)

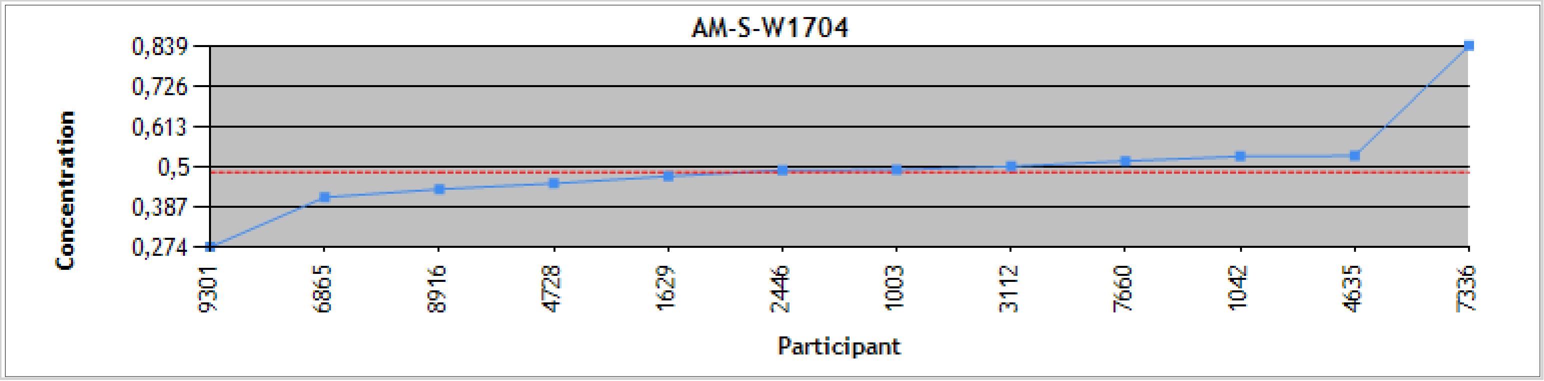

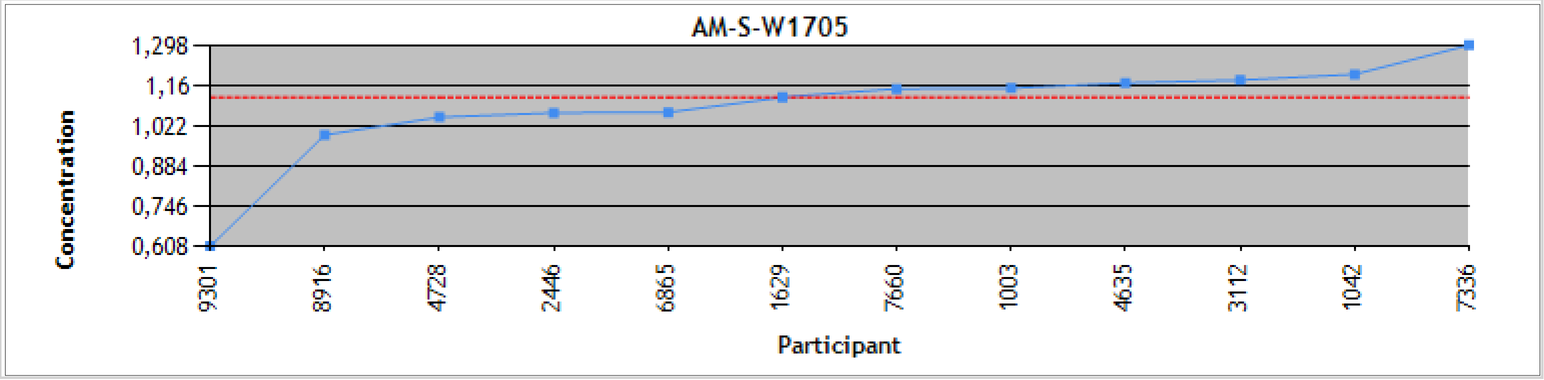

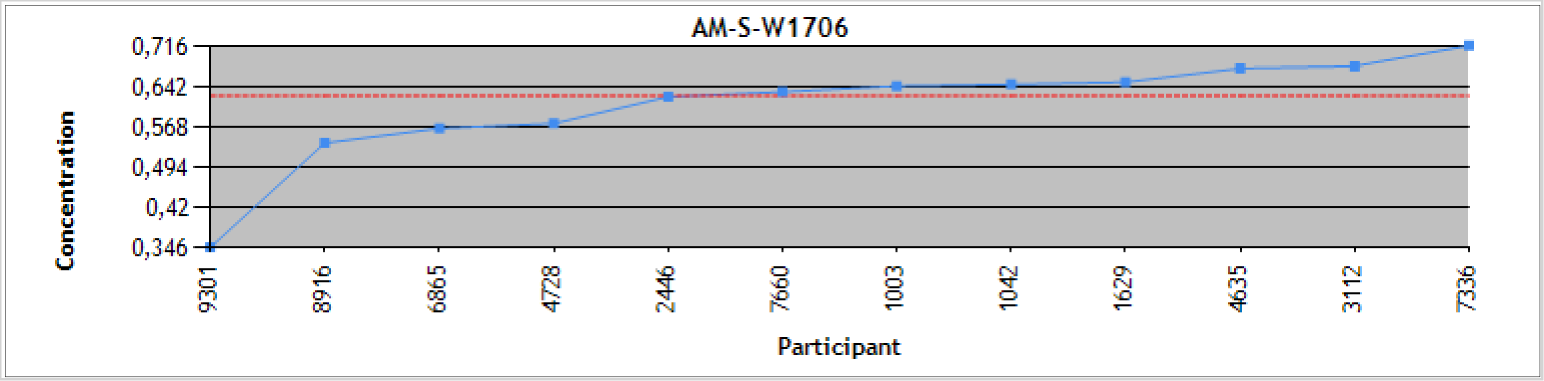

Individual results

Serum PBDE IUPAC # 99 (µg/L)

Round #2017-02

Participant	AM-S-W1704	z'-score	AM-S-W1705	z'-score	AM-S-W1706	z'-score	Method
**1003**	0.102	-0.28	0.462	0.37	0.229	0.19	GC-MS-MS
**1042**	0.102	-0.30	0.443	0.00	0.227	0.14	GC-MS CI
**1629**	0.0980	-0.47	0.439	-0.08	0.229	0.19	GC-MS EI
**2446**	0.100	-0.38	0.385	-1.13	0.210	-0.42	GC-MS EI
**3112**	0.120	0.57	0.513	1.36	0.257	1.09	GC-MS CI
**4635**	0.112	0.19	0.468	0.49	0.241	0.58	GC-MS EI
**4728**	0.105	-0.14	0.452	0.17	0.209	-0.45	GC-MS-MS
**6865**	0.127	0.90	0.465	0.43	0.237	0.45	GC
**7336**	0.141	1.55	0.420	-0.45	0.210	-0.42	GC-MS CI
**7660**	0.0903	-0.84	0.444	0.01	0.224	0.02	GC-MS CI
**8916**	0.142	1.60	0.433	-0.19	0.212	-0.35	GC-MS EI
**9301**	0.0635	**-2.10**	0.231	**-4.12**	0.117	**-3.39**	GC-MS EI

	Assigned value	Standard uncertainty	σ pt	Acceptable range	K-S (Lilliefors)	Species
**AM-S-W1704**	0.108	0.00624	0.0203	0.0656 - 0.150	Accepted	---
**AM-S-W1705**	0.443	0.0112	0.0502	0.340 - 0.546	Accepted	---
**AM-S-W1706**	0.223	0.00643	0.0306	0.161 - 0.285	Accepted	---

Statistics
Serum PBDE IUPAC # 99 (µg/L)

All methods	AM-S-W1704	AM-S-W1705	AM-S-W1706
N	12	12	12
Robust mean Algo A	0.108	0.443	0.223
Robust STDev	0.0173	0.0310	0.0178
Median	0.104	0.443	0.225
STDev from MAD	0.0161	0.0300	0.0214
Arithmetic mean	0.109	0.430	0.217
STDev	0.0220	0.0696	0.0346
CV or Variability	16.0%	7.0%	8.0%

GC-MS CI	AM-S-W1704	AM-S-W1705	AM-S-W1706
N	4	4	4
Robust mean Algo A	0.113	0.444	0.226
Robust STDev	0.0252	0.0230	0.0162
Median	0.111	0.443	0.225
STDev from MAD	0.0221	0.0176	0.0128
Arithmetic mean	0.113	0.455	0.229
STDev	0.0222	0.0403	0.0198
CV or Variability	22.2%	5.2%	7.1%

GC-MS EI	AM-S-W1704	AM-S-W1705	AM-S-W1706
N	5	5	5
Robust mean Algo A	0.102	0.416	0.213
Robust STDev	0.0223	0.0513	0.0287
Median	0.100	0.433	0.212
STDev from MAD	0.0178	0.0519	0.0252
Arithmetic mean	0.103	0.391	0.202
STDev	0.0283	0.0944	0.0491
CV or Variability	21.9%	12.3%	13.4%

When fewer than 20 results were considered for statistical treatment of all or a sub-sample of results, the accuracy of statistical data may be questionable.

Distribution

Serum PBDE IUPAC # 99 (µg/L)

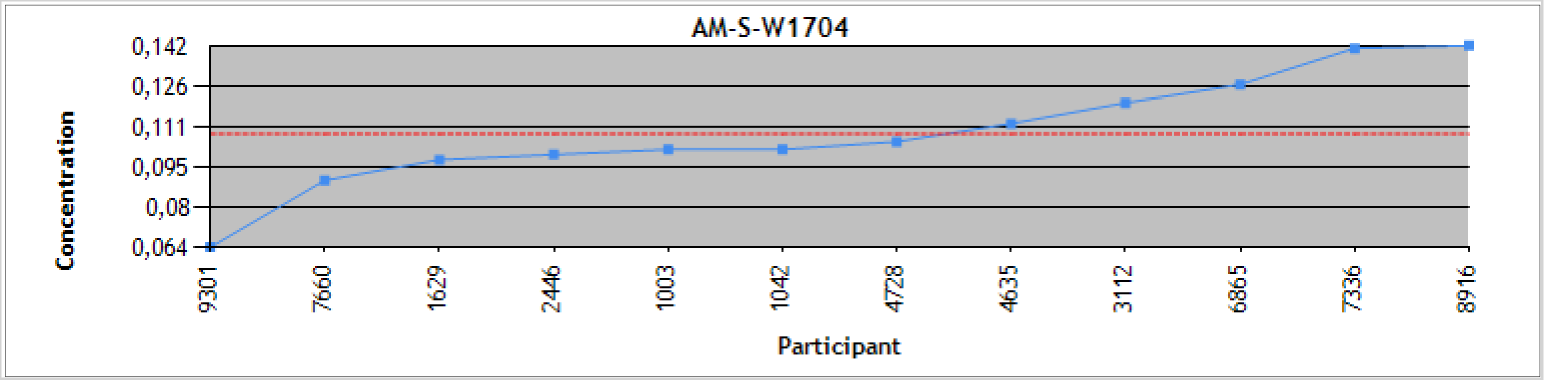

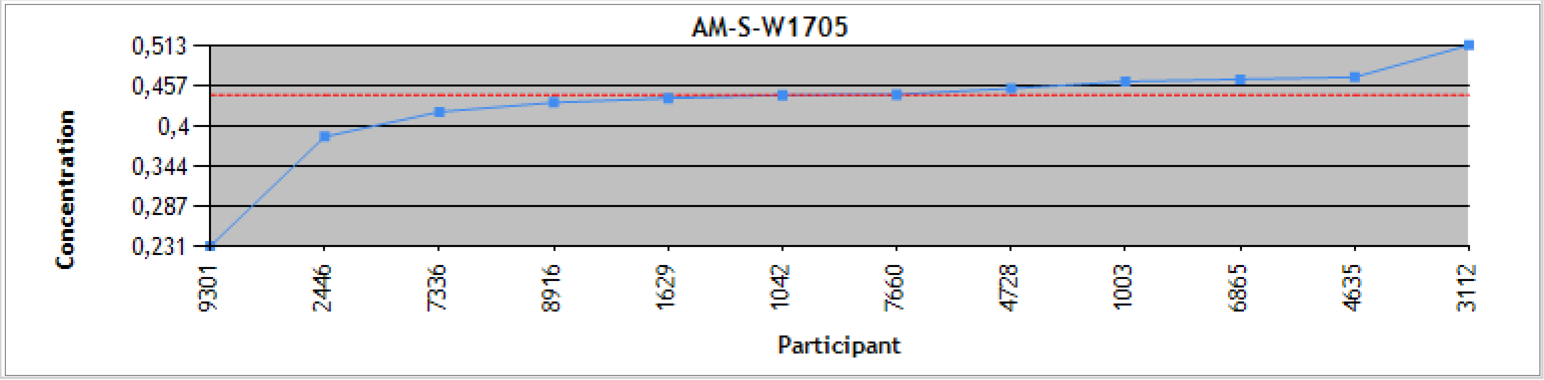

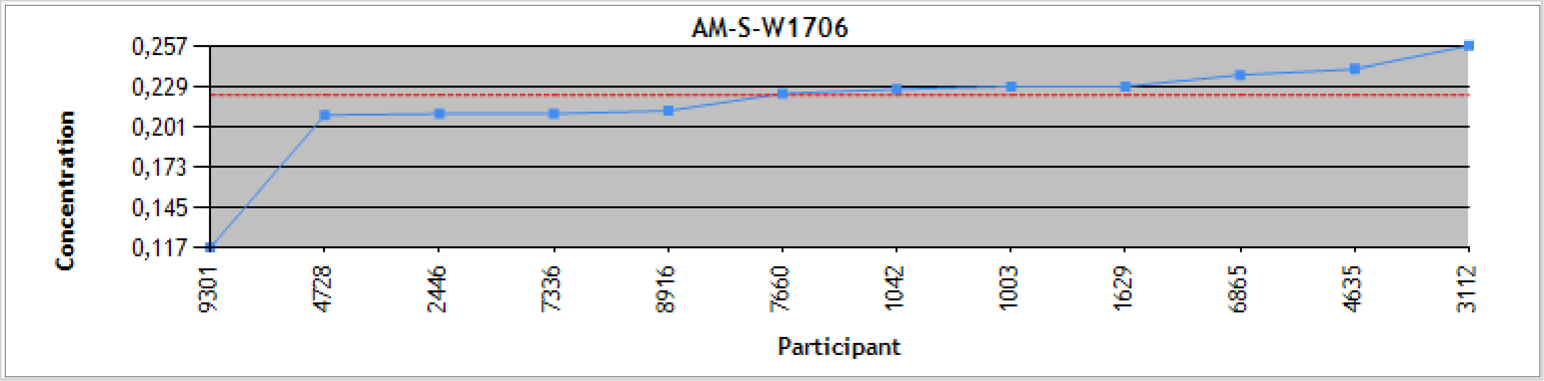

Individual results

Serum PBDE IUPAC # 100 (µg/L)

Round #2017-02

Participant	AM-S-W1704	z'-score	AM-S-W1705	z'-score	AM-S-W1706	z'-score	Method
**1042**	0.167	0.15	0.340	0.36	0.474	0.17	GC-MS CI
**1629**	0.156	-0.36	0.321	-0.10	0.469	0.07	GC-MS EI
**2446**	0.147	-0.76	0.302	-0.55	0.457	-0.15	GC-MS EI
**3112**	0.163	-0.04	0.329	0.10	0.469	0.07	GC-MS CI
**4635**	0.182	0.81	0.368	1.03	0.509	0.81	GC-MS EI
**4728**	0.163	-0.04	0.337	0.29	0.459	-0.11	GC-MS-MS
**6865**	0.189	1.12	0.377	1.25	0.494	0.53	GC
**7336**	0.187	1.03	0.265	-1.44	0.441	-0.44	GC-MS CI
**7660**	0.164	-0.02	0.357	0.77	0.491	0.47	GC-MS CI
**8916**	0.160	-0.18	0.307	-0.43	0.432	-0.61	GC-MS EI
**9301**	0.0855	**-3.52**	0.177	**-3.56**	0.251	**-3.93**	GC-MS EI

	Assigned value	Standard uncertainty	σ pt	Acceptable range	K-S (Lilliefors)	Species
**AM-S-W1704**	0.164	0.00483	0.0218	0.119 - 0.209	Accepted	---
**AM-S-W1705**	0.325	0.0155	0.0386	0.242 - 0.408	Accepted	---
**AM-S-W1706**	0.465	0.0115	0.0532	0.356 - 0.574	Accepted	---

Statistics

Serum PBDE IUPAC # 100 (µg/L)

All methods	AM-S-W1704	AM-S-W1705	AM-S-W1706
N	11	11	11
Robust mean Algo A	0.164	0.325	0.465
Robust STDev	0.0128	0.0411	0.0305
Median	0.163	0.329	0.469
STDev from MAD	0.0104	0.0400	0.0319
Arithmetic mean	0.160	0.316	0.450
STDev	0.0281	0.0561	0.0696
CV or Variability	7.8%	12.6%	6.6%

GC-MS CI	AM-S-W1704	AM-S-W1705	AM-S-W1706
N	4	4	4
Robust mean Algo A	0.166	0.332	0.470
Robust STDev	0.00384	0.0255	0.0201
Median	0.165	0.335	0.472
STDev from MAD	0.00319	0.0208	0.0159
Arithmetic mean	0.170	0.323	0.469
STDev	0.0114	0.0402	0.0206
CV or Variability	2.3%	7.7%	4.3%

GC-MS EI	AM-S-W1704	AM-S-W1705	AM-S-W1706
N	5	5	5
Robust mean Algo A	0.155	0.309	0.454
Robust STDev	0.0169	0.0263	0.0458
Median	0.156	0.307	0.457
STDev from MAD	0.0133	0.0208	0.0371
Arithmetic mean	0.146	0.295	0.424
STDev	0.0362	0.0709	0.100
CV or Variability	10.9%	8.5%	10.1%

When fewer than 20 results were considered for statistical treatment of all or a sub-sample of results, the accuracy of statistical data may be questionable.

Distribution
Serum PBDE IUPAC # 100 (µg/L)

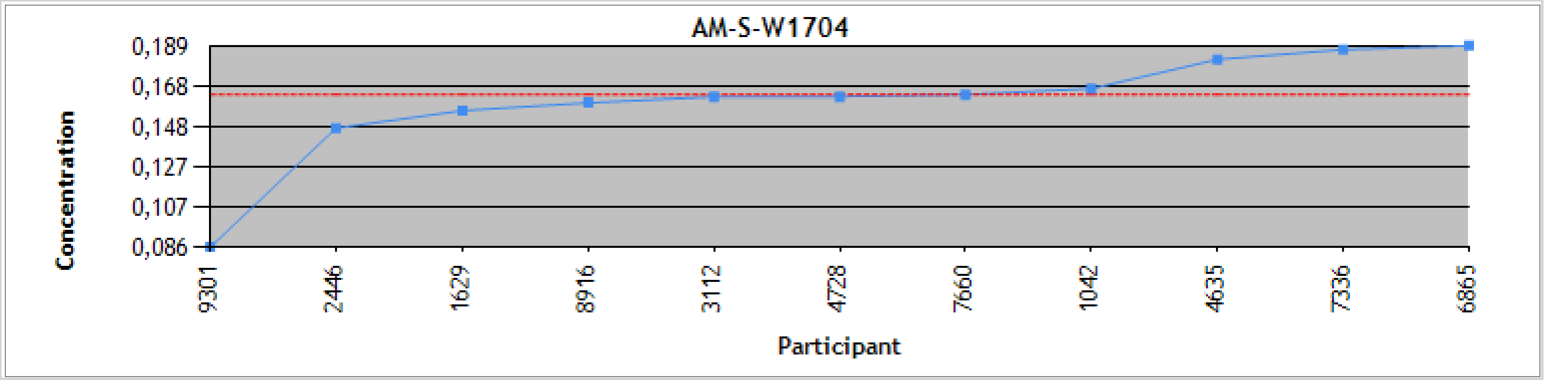

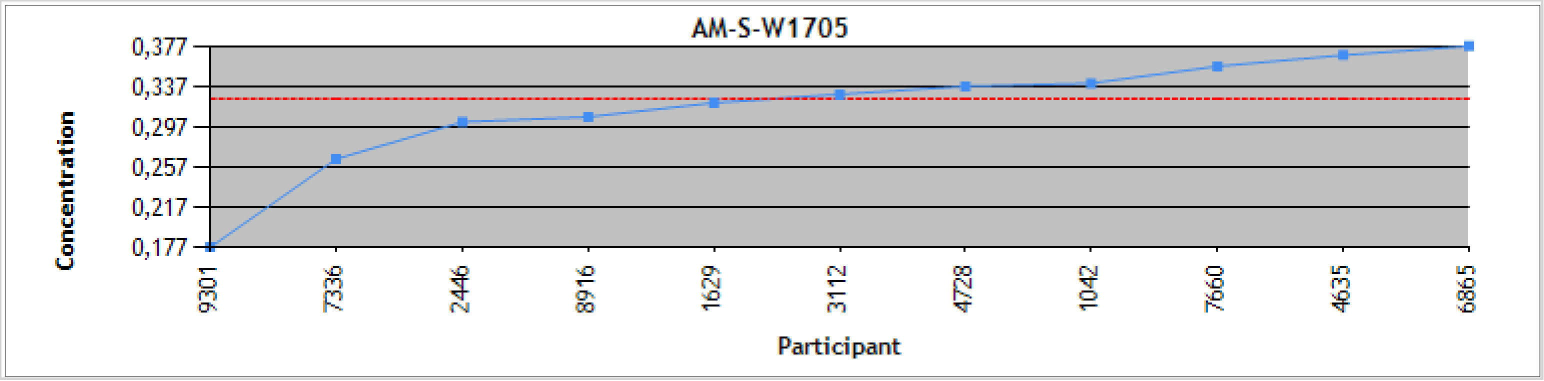

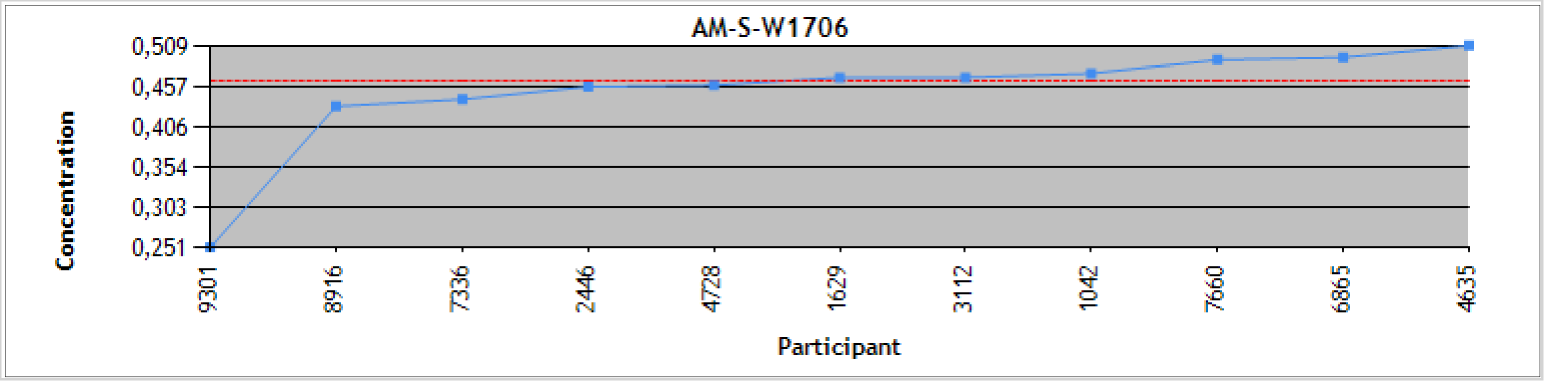

Individual results

Serum PBDE IUPAC # 153 (µg/L)

Round #2017-02

Participant	AM-S-W1704	z'-score	AM-S-W1705	z'-score	AM-S-W1706	z'-score	Method
**1003**	0.223	0.15	0.544	0.37	0.0840	0.06	GC-MS-MS
**1042**	0.238	0.62	0.543	0.36	0.0885	0.35	GC-MS CI
**1629**	0.224	0.19	0.530	0.17	0.0860	0.19	GC-MS EI
**2446**	0.210	-0.25	0.474	-0.63	0.0830	-0.01	GC-MS EI
**3112**	0.239	0.65	0.577	0.85	0.0890	0.39	GC-MS CI
**4635**	0.245	0.83	0.569	0.73	0.0880	0.32	GC-MS EI
**4728**	0.220	0.06	0.527	0.13	0.0730	-0.66	GC-MS-MS
**6865**	0.207	-0.34	0.451	-0.96	0.100	1.11	GC
**7336**	0.186	-0.99	0.305	**-3.06**	0.0520	**-2.05**	GC-MS CI
**7660**	0.200	-0.57	0.531	0.18	0.0736	-0.63	GC-MS CI
**8916**	0.237	0.59	0.522	0.06	0.0850	0.13	GC-MS EI
**9301**	0.119	**-3.06**	0.256	**-3.76**	0.0460	**-2.44**	GC-MS EI

	Assigned value	Standard uncertainty	σ pt	Acceptable range	K-S (Lilliefors)	Species
**AM-S-W1704**	0.218	0.00836	0.0313	0.153 - 0.283	Accepted	---
**AM-S-W1705**	0.518	0.0164	0.0676	0.379 - 0.657	Accepted	---
**AM-S-W1706**	0.0831	0.00268	0.0150	0.0527 - 0.114	Accepted	---

Statistics

Serum PBDE IUPAC # 153 (µg/L)

All methods	AM-S-W1704	AM-S-W1705	AM-S-W1706
N	12	12	12
Robust mean Algo A	0.218	0.518	0.0831
Robust STDev	0.0232	0.0456	0.00743
Median	0.222	0.529	0.0845
STDev from MAD	0.0238	0.0415	0.00627
Arithmetic mean	0.212	0.486	0.0790
STDev	0.0343	0.103	0.0157
CV or Variability	10.6%	8.8%	8.9%

GC-MS CI	AM-S-W1704	AM-S-W1705	AM-S-W1706
N	4	4	4
Robust mean Algo A	0.216	0.534	0.0787
Robust STDev	0.0306	0.0430	0.0138
Median	0.219	0.537	0.0810
STDev from MAD	0.0293	0.0343	0.0114
Arithmetic mean	0.216	0.489	0.0758
STDev	0.0270	0.124	0.0174
CV or Variability	14.2%	8.1%	17.6%

GC-MS EI	AM-S-W1704	AM-S-W1705	AM-S-W1706
N	5	5	5
Robust mean Algo A	0.222	0.502	0.0845
Robust STDev	0.0237	0.0661	0.00324
Median	0.224	0.522	0.0850
STDev from MAD	0.0208	0.0697	0.00297
Arithmetic mean	0.207	0.470	0.0776
STDev	0.0510	0.124	0.0178
CV or Variability	10.7%	13.2%	3.8%

When fewer than 20 results were considered for statistical treatment of all or a sub-sample of results, the accuracy of statistical data may be questionable.

Distribution

Serum PBDE IUPAC # 153 (µg/L)

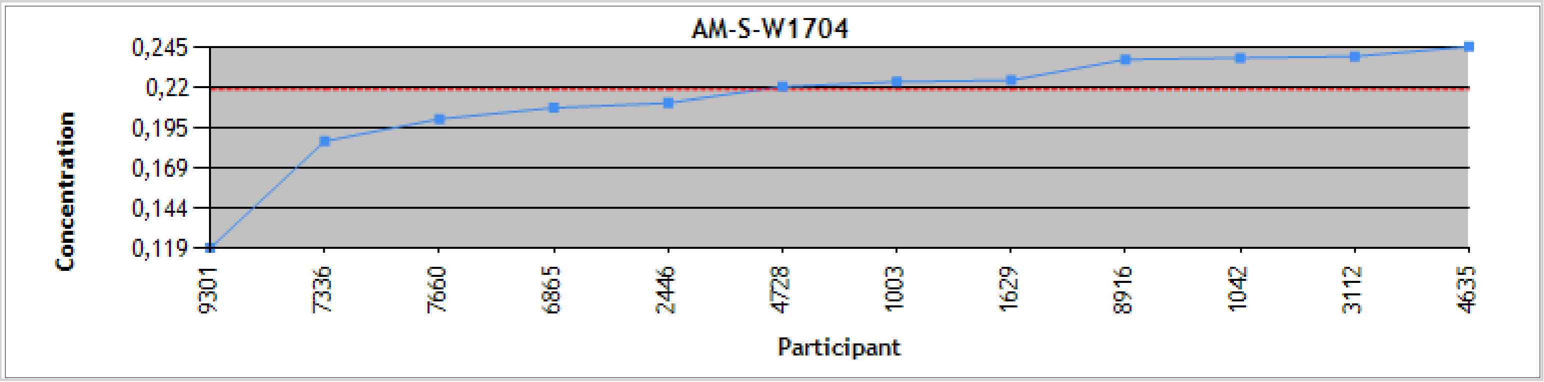

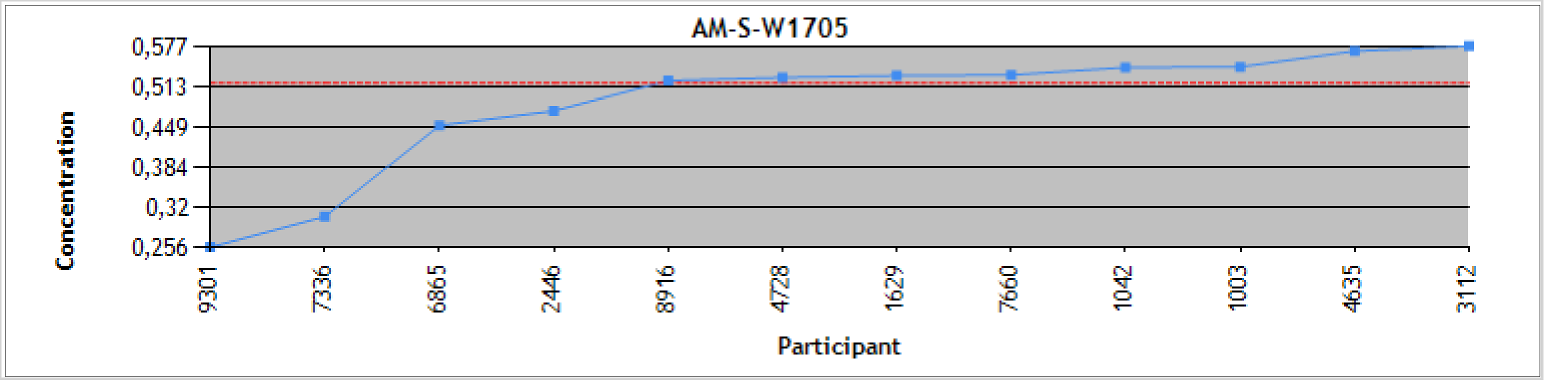

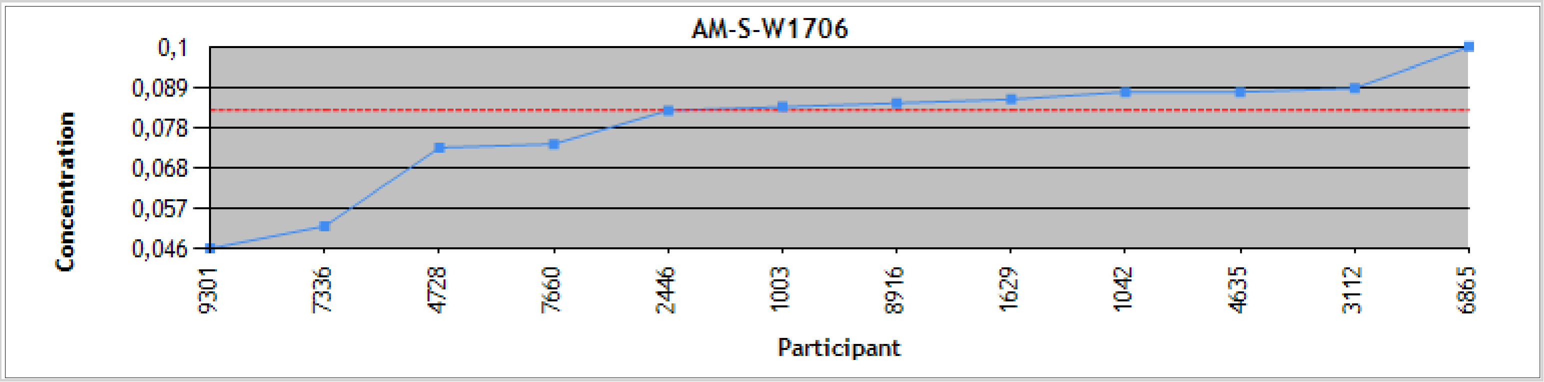

Individual results

Serum PBDE IUPAC # 154 (µg/L)

Round #2017-02

Participant	AM-S-W1704	z'-score	AM-S-W1705	z'-score	AM-S-W1706	z'-score	Method
**1042**	0.0742	-0.11	0.604	0.45	0.368	0.49	GC-MS CI
**1629**	0.0710	-0.30	0.608	0.52	0.384	0.84	GC-MS EI
**2446**	0.0700	-0.36	0.548	-0.32	0.357	0.26	GC-MS EI
**3112**	0.0800	0.24	0.609	0.53	0.367	0.47	GC-MS CI
**4635**	0.0830	0.42	0.662	1.27	0.407	1.33	GC-MS EI
**4728**	0.0840	0.48	0.672	1.41	0.385	0.86	GC-MS-MS
**6865**	0.0952	1.16	0.513	-0.81	0.300	-0.96	GC
**7336**	0.0760	0.00	0.388	**-2.56**	0.285	-1.28	GC-MS CI
**8916**	0.0720	-0.24	0.556	-0.21	0.307	-0.81	GC-MS EI
**9301**	0.0370	**-2.36**	0.293	**-3.89**	0.178	**-3.58**	GC-MS EI

	Assigned value	Standard uncertainty	σ pt	Acceptable range	K-S (Lilliefors)	Species
**AM-S-W1704**	0.0760	0.00320	0.0162	0.0429 - 0.109	Accepted	---
**AM-S-W1705**	0.571	0.0323	0.0638	0.428 - 0.714	Accepted	---
**AM-S-W1706**	0.345	0.0203	0.0420	0.252 - 0.438	Accepted	---

Statistics

Serum PBDE IUPAC # 154 (µg/L)

All methods	AM-S-W1704	AM-S-W1705	AM-S-W1706
N	10	10	10
Robust mean Algo A	0.0760	0.571	0.345
Robust STDev	0.00809	0.0818	0.0514
Median	0.0751	0.580	0.362
STDev from MAD	0.00742	0.0730	0.0504
Arithmetic mean	0.0742	0.545	0.334
STDev	0.0152	0.120	0.0682
CV or Variability	10.6%	14.3%	14.9%

GC-MS CI	AM-S-W1704	AM-S-W1705	AM-S-W1706
N	3	3	3
Robust mean Algo A	0.0767	0.601	0.367
Robust STDev	0.00339	0.0103	0.00206
Median	0.0760	0.604	0.367
STDev from MAD	0.00273	0.00816	0.00163
Arithmetic mean	0.0767	0.534	0.340
STDev	0.00299	0.126	0.0477
CV or Variability	4.4%	1.7%	0.6%

GC-MS EI	AM-S-W1704	AM-S-W1705	AM-S-W1706
N	5	5	5
Robust mean Algo A	0.0710	0.563	0.340
Robust STDev	0.00196	0.0934	0.0732
Median	0.0710	0.556	0.357
STDev from MAD	0.00148	0.0771	0.0742
Arithmetic mean	0.0666	0.533	0.327
STDev	0.0174	0.142	0.0910
CV or Variability	2.8%	16.6%	21.5%

When fewer than 20 results were considered for statistical treatment of all or a sub-sample of results, the accuracy of statistical data may be questionable.

Distribution

Serum PBDE IUPAC # 154 (µg/L)

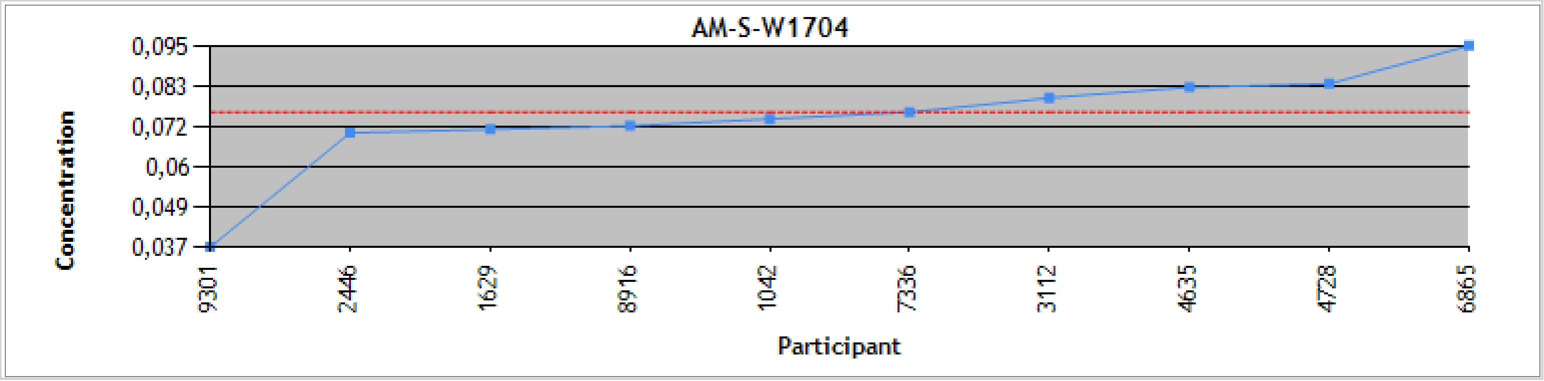

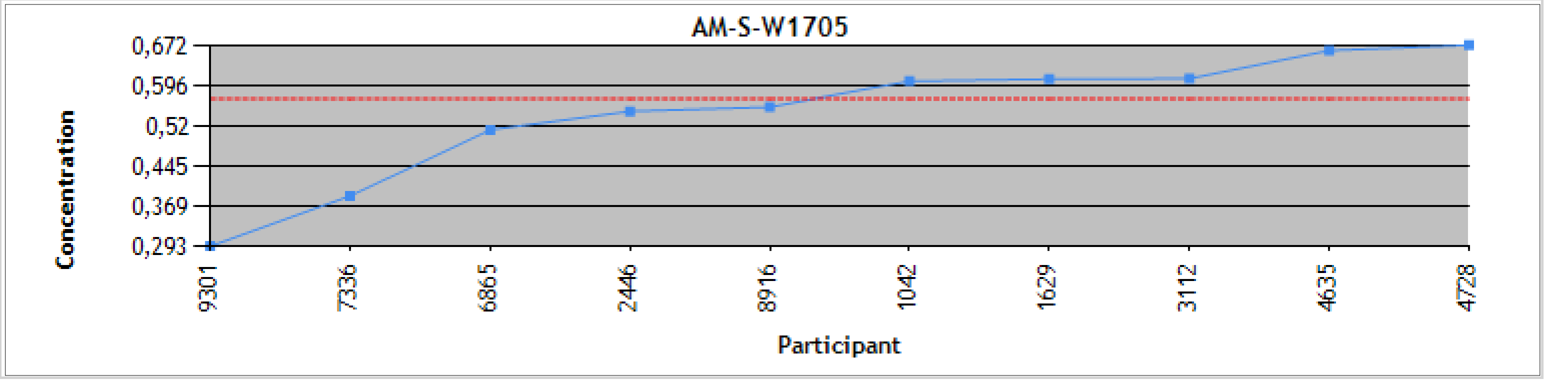

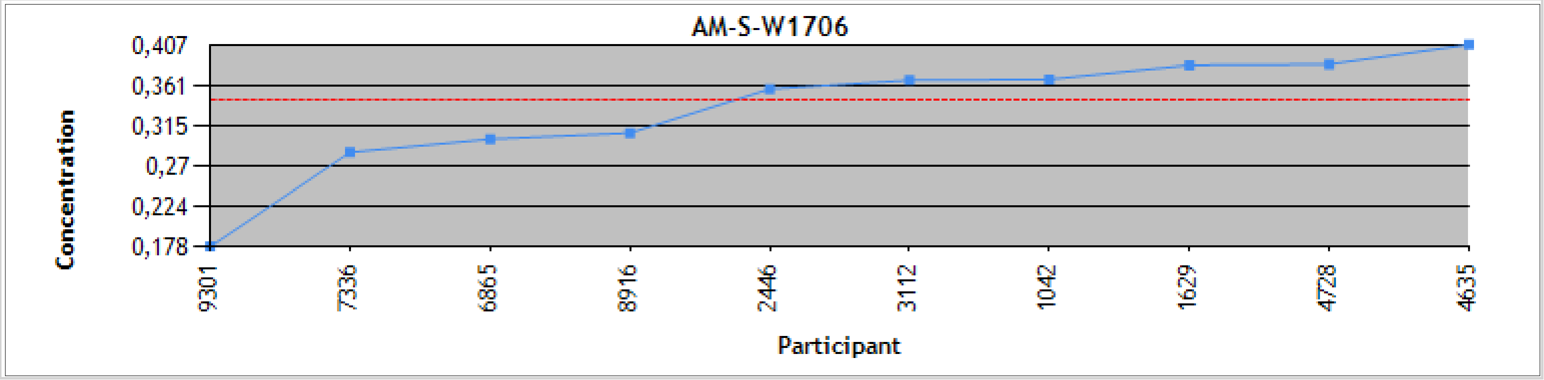

Individual results

Serum PBDE IUPAC # 183 (µg/L)

Round #2017-02

Participant	AM-S-W1704	z'-score	AM-S-W1705	z'-score	AM-S-W1706	z'-score	Method
**1042**	0.236	0.40	0.393	0.55	0.129	0.94	GC-MS CI
**1629**	0.226	0.18	0.377	0.33	0.119	0.56	GC-MS EI
**2446**	0.240	0.48	0.364	0.14	0.111	0.24	GC-MS EI
**3112**	0.215	-0.07	0.372	0.26	0.0980	-0.28	GC-MS CI
**4635**	0.242	0.53	0.397	0.61	0.115	0.40	GC-MS EI
**4728**	0.205	-0.29	0.353	-0.01	0.0970	-0.32	GC-MS-MS
**7336**	0.164	-1.19	0.200	**-2.18**	0.0730	-1.27	GC-MS CI
**8916**	0.224	0.13	0.329	-0.35	0.106	0.04	GC-MS EI
**9301**	0.119	**-2.18**	0.206	**-2.10**	0.0595	-1.80	GC-MS EI

	Assigned value	Standard uncertainty	σ pt	Acceptable range	K-S (Lilliefors)	Species
**AM-S-W1704**	0.218	0.00977	0.0444	0.127 - 0.309	Accepted	---
**AM-S-W1705**	0.354	0.0174	0.0684	0.213 - 0.495	Accepted	---
**AM-S-W1706**	0.105	0.00671	0.0243	0.0546 - 0.155	Accepted	---

Statistics

Serum PBDE IUPAC # 183 (µg/L)

All methods	AM-S-W1704	AM-S-W1705	AM-S-W1706
N	9	9	9
Robust mean Algo A	0.218	0.354	0.105
Robust STDev	0.0234	0.0417	0.0161
Median	0.224	0.364	0.106
STDev from MAD	0.0237	0.0429	0.0133
Arithmetic mean	0.208	0.332	0.101
STDev	0.0410	0.0761	0.0222
CV or Variability	10.7%	11.8%	15.3%

GC-MS CI	AM-S-W1704	AM-S-W1705	AM-S-W1706
N	3	3	3
Robust mean Algo A	0.206	0.363	0.0999
Robust STDev	0.0397	0.0391	0.0317
Median	0.215	0.372	0.0980
STDev from MAD	0.0314	0.0310	0.0371
Arithmetic mean	0.205	0.322	0.0999
STDev	0.0371	0.106	0.0280
CV or Variability	19.2%	10.8%	31.7%

GC-MS EI	AM-S-W1704	AM-S-W1705	AM-S-W1706
N	5	5	5
Robust mean Algo A	0.225	0.352	0.110
Robust STDev	0.0214	0.0478	0.00851
Median	0.226	0.364	0.111
STDev from MAD	0.0208	0.0489	0.00742
Arithmetic mean	0.210	0.335	0.102
STDev	0.0516	0.0760	0.0243
CV or Variability	9.5%	13.6%	7.7%

When fewer than 20 results were considered for statistical treatment of all or a sub-sample of results, the accuracy of statistical data may be questionable.

Distribution
Serum PBDE IUPAC # 183 (µg/L)

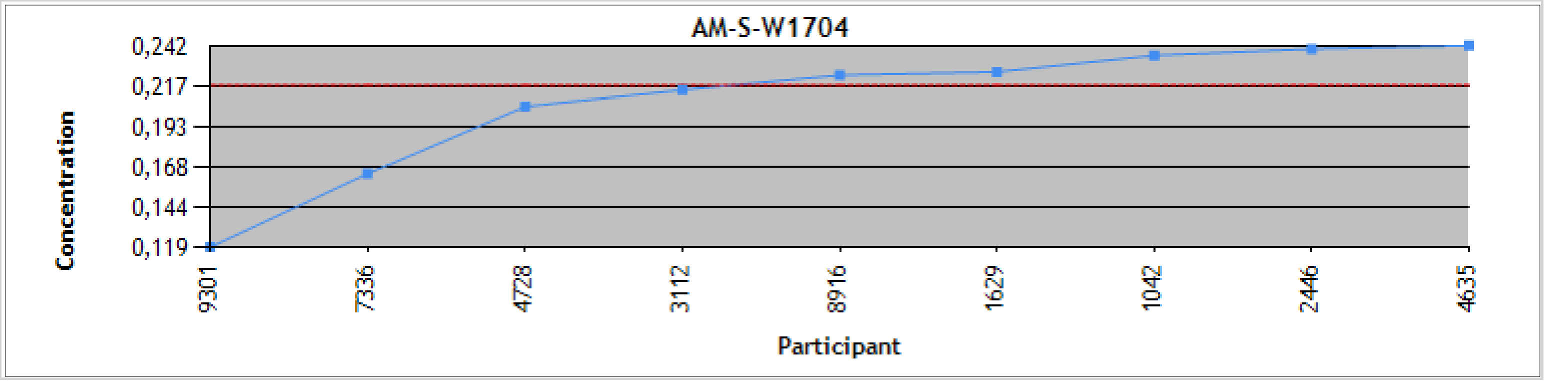

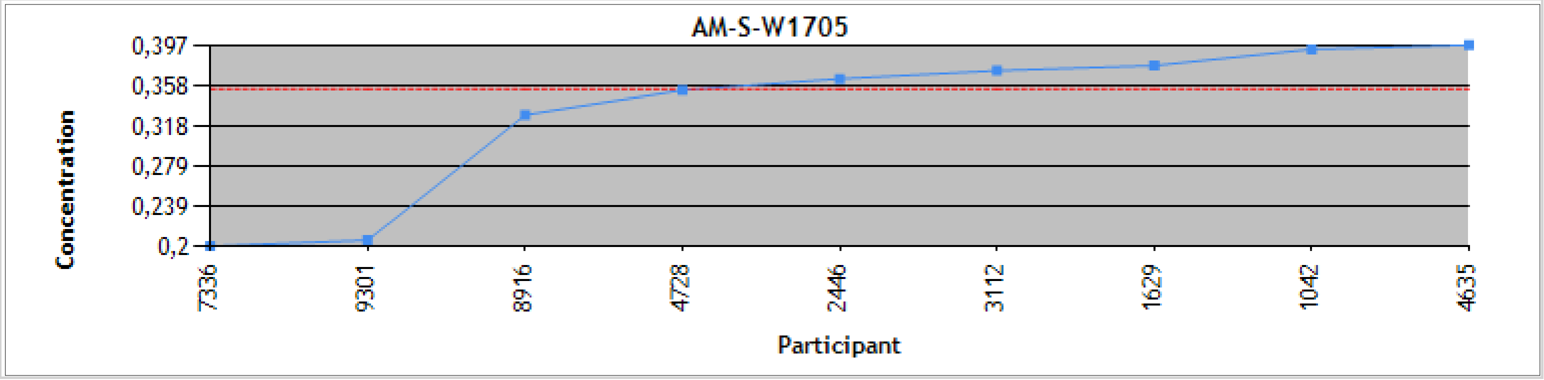

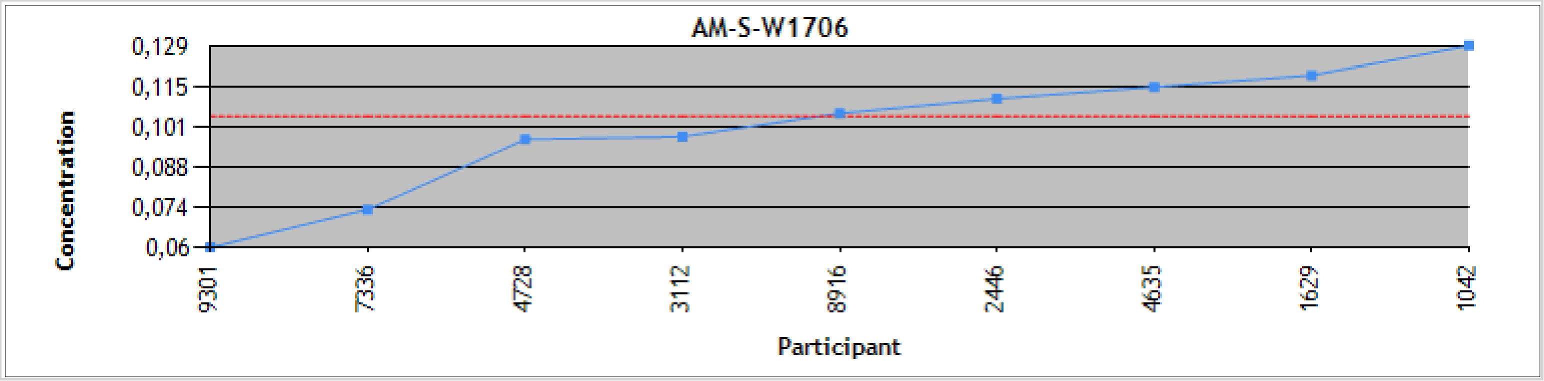

Individual results

Serum PBDE IUPAC # 209 (µg/L)

Round #2017-02

Participant	AM-S-W1704	z'-score	AM-S-W1705	z'-score	AM-S-W1706	z'-score	Method
**1042**	0.277	0.80	1.13	0.31	0.667	0.44	GC-MS CI
**1629**	0.214	-0.21	1.60	1.99	0.752	1.06	GC-MS EI
**2446**	0.195	-0.51	0.762	-0.99	0.376	-1.67	GC-MS EI
**2724**	0.168	-0.95	0.556	-1.72	0.301	**-2.21**	GC-MS CI
**3112**	0.255	0.45	1.30	0.93	0.697	0.66	GC-MS CI
**4635**	0.326	1.59	1.34	1.08	0.763	1.14	GC-MS EI
**9301**	0.166	-0.98	0.622	-1.48	0.390	-1.57	GC-MS EI

	Assigned value	Standard uncertainty	σ pt	Acceptable range	K-S (Lilliefors)	Species
**AM-S-W1704**	0.227	0.0306	0.0541	0.103 - 0.351	Accepted	---
**AM-S-W1705**	1.04	0.215	0.183	0.476 - 1.60	Accepted	---
**AM-S-W1706**	0.606	0.0782	0.114	0.330 - 0.882	Accepted	---

Statistics

Serum PBDE IUPAC # 209 (µg/L)

All methods	AM-S-W1704	AM-S-W1705	AM-S-W1706
N	7	7	7
Robust mean Algo A	0.227	1.04	0.606
Robust STDev	0.0649	0.455	0.165
Median	0.214	1.13	0.667
STDev from MAD	0.0682	0.541	0.142
Arithmetic mean	0.229	1.04	0.564
STDev	0.0598	0.402	0.199
CV or Variability	28.5%	43.6%	27.3%

GC-MS CI	AM-S-W1704	AM-S-W1705	AM-S-W1706
N	3	3	3
Robust mean Algo A	0.246	1.06	0.655
Robust STDev	0.0406	0.328	0.0562
Median	0.255	1.13	0.667
STDev from MAD	0.0322	0.260	0.0445
Arithmetic mean	0.233	0.995	0.555
STDev	0.0575	0.390	0.220
CV or Variability	16.5%	31.0%	8.6%

GC-MS EI	AM-S-W1704	AM-S-W1705	AM-S-W1706
N	4	4	4
Robust mean Algo A	0.208	1.08	0.570
Robust STDev	0.0437	0.528	0.245
Median	0.205	1.05	0.571
STDev from MAD	0.0356	0.535	0.277
Arithmetic mean	0.225	1.08	0.570
STDev	0.0700	0.466	0.216
CV or Variability	21.0%	48.8%	43.0%

When fewer than 20 results were considered for statistical treatment of all or a sub-sample of results, the accuracy of statistical data may be questionable.

Distribution

Serum PBDE IUPAC # 209 (µg/L)

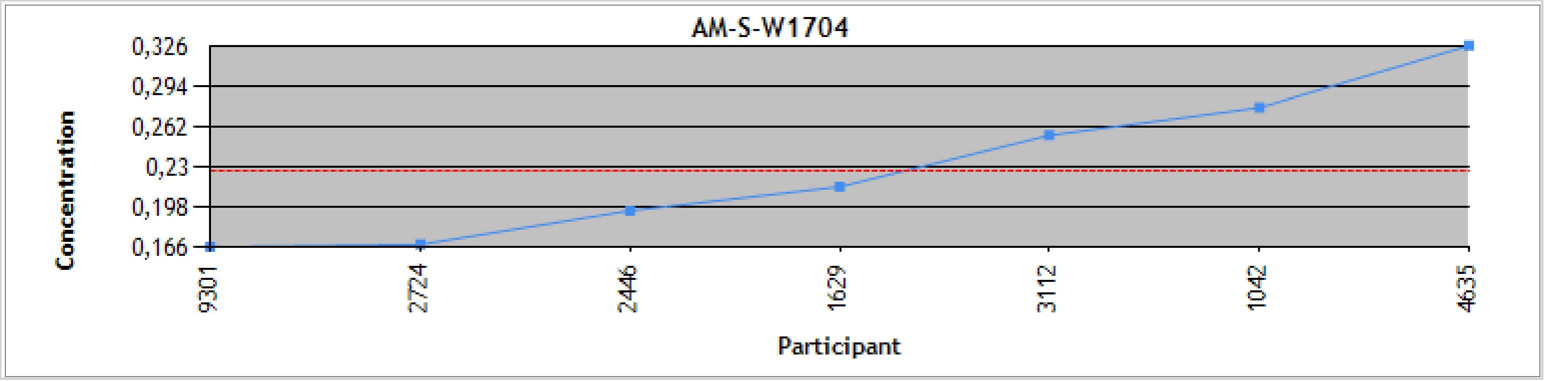

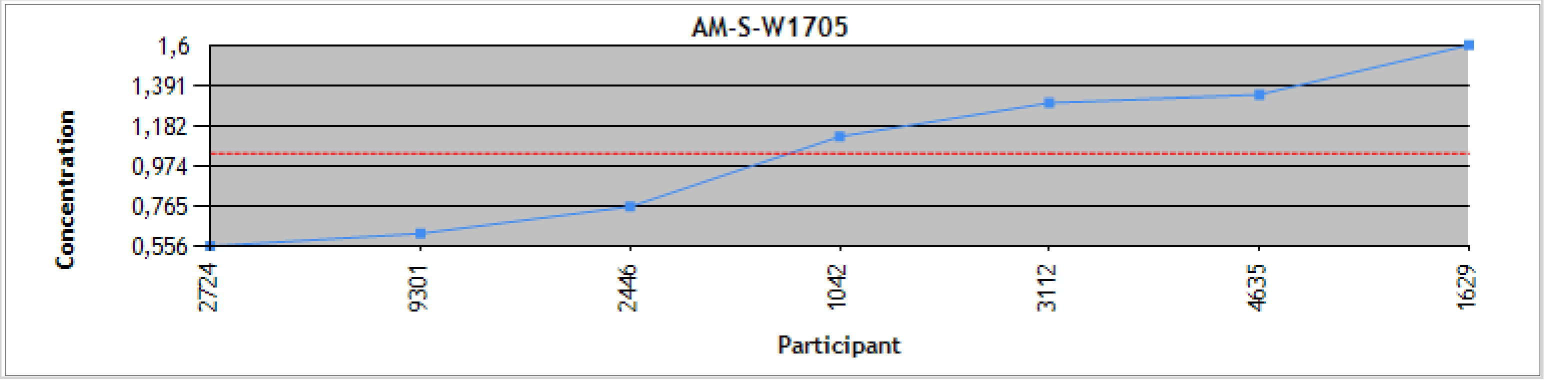

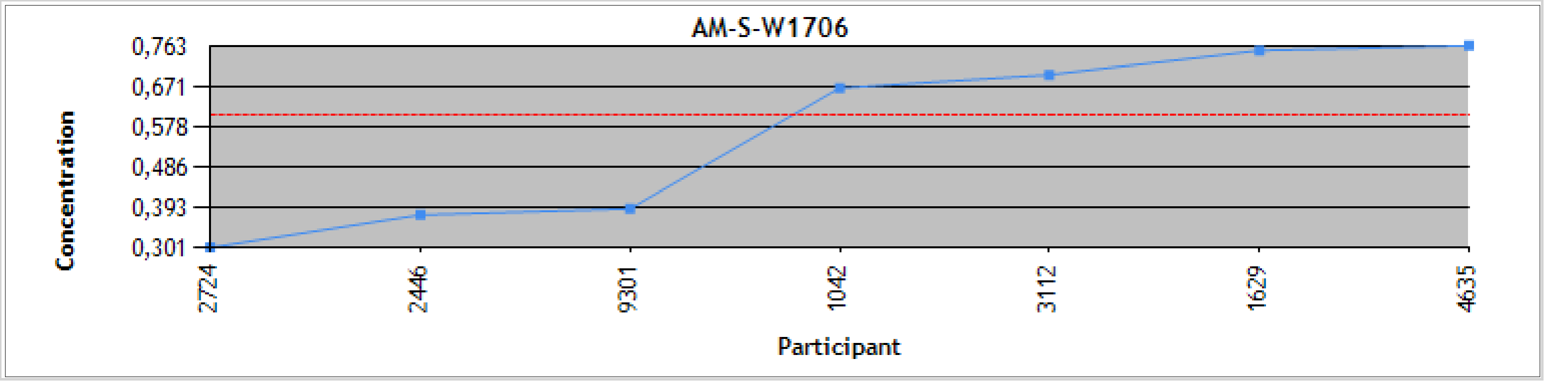

Individual results

Serum PCB IUPAC # 28 (µg/L)

Round #2017-02

Participant	AM-S-W1704	z'-score	AM-S-W1705	z'-score	AM-S-W1706	z'-score	Method
**270**	0.120	-1.71	0.390	**-2.49**	0.205	**-2.37**	GC-MS EI
**332**	0.203	0.00	0.756	0.12	0.389	-0.12	ND
**748**	0.194	-0.19	0.667	-0.51	0.470	0.87	ND
**1042**	0.200	-0.05	0.712	-0.20	0.373	-0.32	GC-MS CI
**1629**	0.227	0.49	0.863	0.88	0.477	0.95	GC-MS-MS
**2446**	0.196	-0.14	0.667	-0.51	0.354	-0.55	GC-MS EI
**3112**	0.113	-1.85	---	---	---	---	GC-MS CI
**4635**	0.220	0.35	0.847	0.77	0.432	0.40	GC-MS EI
**6865**	0.249	0.95	0.744	0.04	0.422	0.28	GC
**7660**	0.191	-0.24	0.741	0.02	0.404	0.06	GC-MS CI
**7828**	0.211	0.16	0.768	0.21	0.417	0.22	GC-MS EI
**8559**	0.201	-0.04	0.643	-0.69	0.344	-0.67	ND
**8916**	0.220	0.35	0.869	0.93	0.390	-0.11	GC-MS EI

	Assigned value	Standard uncertainty	σ pt	Acceptable range	K-S (Lilliefors)	Species
**AM-S-W1704**	0.203	0.00612	0.0482	0.106 - 0.300	Accepted	---
**AM-S-W1705**	0.739	0.0368	0.135	0.459 - 1.02	Accepted	---
**AM-S-W1706**	0.399	0.0179	0.0799	0.235 - 0.563	Accepted	---

Statistics

Serum PCB IUPAC # 28 (µg/L)

All methods	AM-S-W1704	AM-S-W1705	AM-S-W1706
N	13	12	12
Robust mean Algo A	0.203	0.739	0.399
Robust STDev	0.0177	0.102	0.0495
Median	0.201	0.743	0.397
STDev from MAD	0.0148	0.112	0.0444
Arithmetic mean	0.196	0.722	0.390
STDev	0.0387	0.129	0.0711
CV or Variability	8.7%	13.8%	12.4%

GC-MS CI	AM-S-W1704	AM-S-W1705	AM-S-W1706
N	3	NA	NA
Robust mean Algo A	0.188	NA	NA
Robust STDev	0.0168	NA	NA
Median	0.191	NA	NA
STDev from MAD	0.0133	NA	NA
Arithmetic mean	0.168	NA	NA
STDev	0.0481	NA	NA
CV or Variability	9.0%	NA	NA

GC-MS EI	AM-S-W1704	AM-S-W1705	AM-S-W1706
N	5	5	5
Robust mean Algo A	0.208	0.739	0.381
Robust STDev	0.0153	0.153	0.0560
Median	0.211	0.768	0.390
STDev from MAD	0.0133	0.150	0.0534
Arithmetic mean	0.193	0.708	0.360
STDev	0.0422	0.195	0.0914
CV or Variability	7.4%	20.7%	14.7%

When fewer than 20 results were considered for statistical treatment of all or a sub-sample of results, the accuracy of statistical data may be questionable.

Distribution

Serum PCB IUPAC # 28 (µg/L)

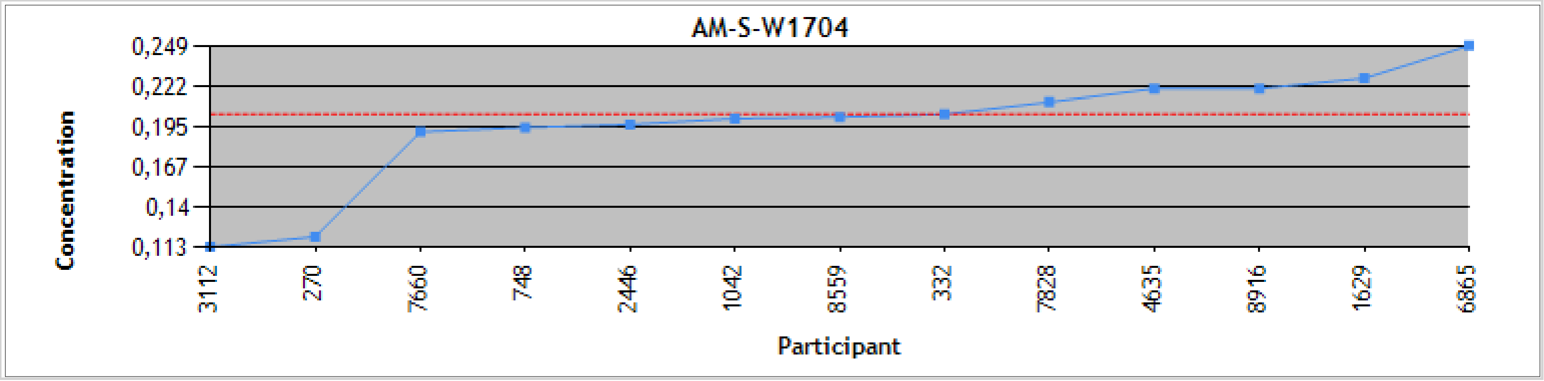

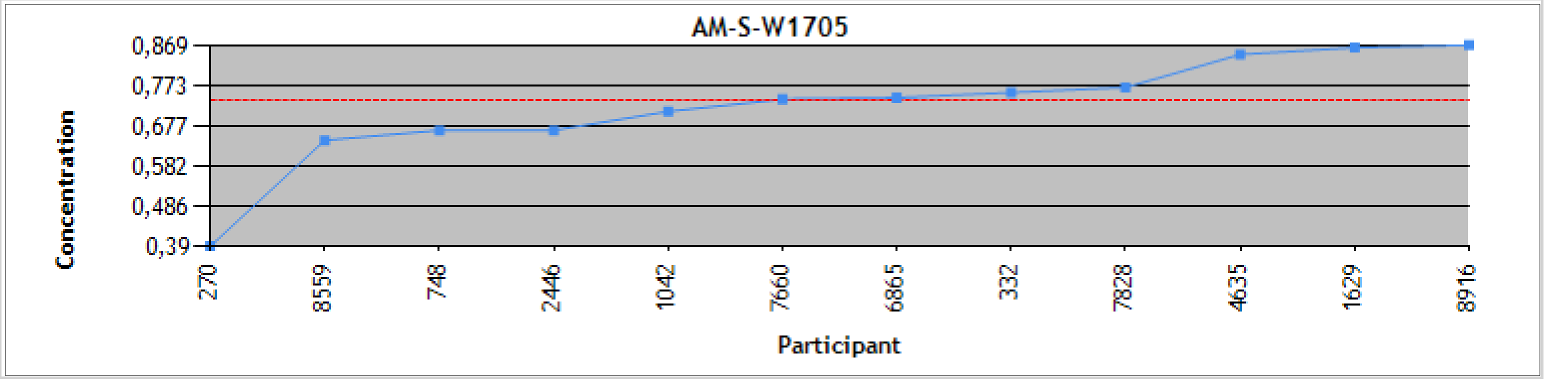

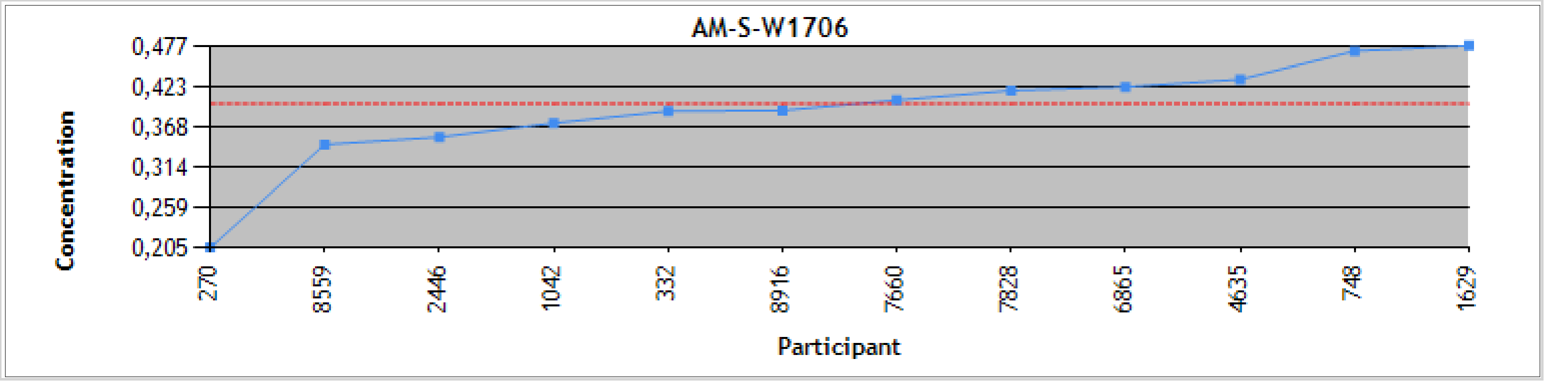

Individual results

Serum PCB IUPAC # 52 (µg/L)

Round #2017-02

Participant	AM-S-W1704	z'-score	AM-S-W1705	z'-score	AM-S-W1706	z'-score	Method
**270**	0.121	0.00	0.310	0.47	0.578	0.22	GC-MS EI
**332**	0.0970	-0.97	0.250	-0.57	0.540	-0.13	ND
**748**	0.123	0.08	0.324	0.71	0.697	1.28	ND
**1042**	<LD	---	0.275	-0.14	0.521	-0.29	GC-MS CI
**1629**	0.124	0.12	0.284	0.02	0.606	0.47	GC-MS-MS
**2446**	0.119	-0.08	0.259	-0.42	0.549	-0.04	GC-MS EI
**6865**	0.138	0.68	0.305	0.38	0.570	0.14	GC
**7660**	<LD	---	0.310	0.47	0.555	0.01	GC-MS CI
**7828**	0.113	-0.32	0.252	-0.54	0.534	-0.18	GC-MS EI
**8559**	0.138	0.68	0.274	-0.16	0.527	-0.24	ND
**8916**	0.110	-0.44	0.269	-0.24	0.528	-0.23	GC-MS EI
**9426**	<LD	---	<LD	---	<LD	---	GC-MS-MS

	Assigned value	Standard uncertainty	σ pt	Acceptable range	K-S (Lilliefors)	Species
**AM-S-W1704**	0.121	0.00553	0.0242	0.0713 - 0.171	Accepted	---
**AM-S-W1705**	0.283	0.0110	0.0566	0.168 - 0.398	Accepted	---
**AM-S-W1706**	0.554	0.0116	0.111	0.331 - 0.777	Accepted	---

PCB IUPAC # 52 is not included in the scope of our accreditation.

Statistics

Serum PCB IUPAC # 52 (µg/L)

All methods	AM-S-W1704	AM-S-W1705	AM-S-W1706
**N**	9	11	11
**Robust mean Algo A**	0.121	0.283	0.554
**Robust STDev**	0.0133	0.0291	0.0309
**Median**	0.121	0.275	0.549
**STDev from MAD**	0.0119	0.0341	0.0311
**Arithmetic mean**	0.120	0.283	0.564
**STDev**	0.0130	0.0257	0.0509
**CV or Variability**	11.0%	10.3%	5.6%

GC-MS EI	AM-S-W1704	AM-S-W1705	AM-S-W1706
**N**	8	4	4
**Robust mean Algo A**	0.116	0.266	0.544
**Robust STDev**	0.00538	0.0152	0.0187
**Median**	0.116	0.264	0.542
**STDev from MAD**	0.00593	0.0126	0.0156
**Arithmetic mean**	0.116	0.273	0.547
**STDev**	0.00474	0.0260	0.0223
**CV or Variability**	4.6%	5.7%	3.4%

When fewer than 20 results were considered for statistical treatment of all or a sub-sample of results, the accuracy of statistical data may be questionable.

Distribution
Serum PCB IUPAC # 52 (µg/L)

Distribution graph for PCB IUPAC # 52 in material AM-S-W1704 is not available.

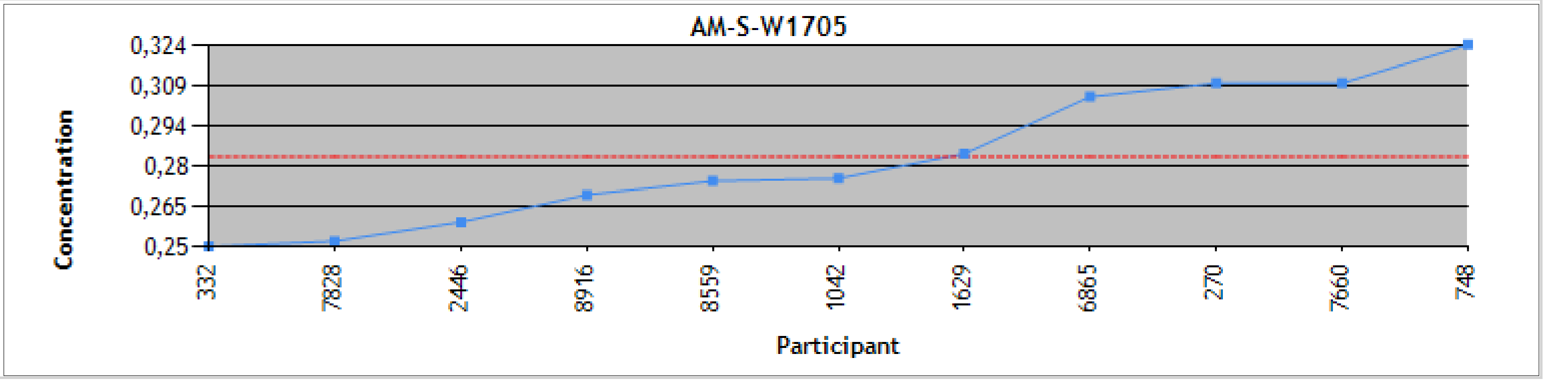

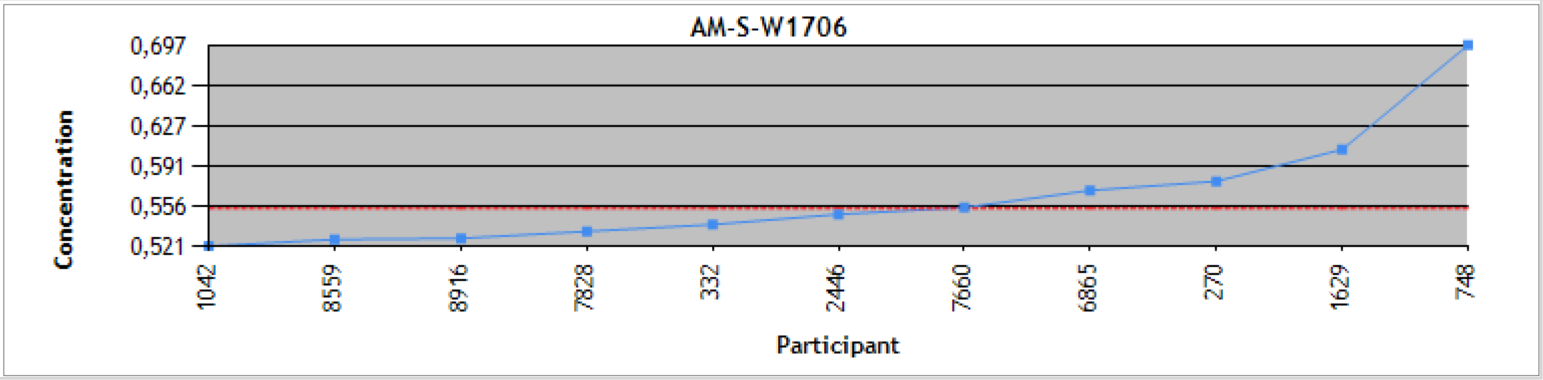

Individual results

Serum PCB IUPAC # 74 (µg/L)

Round #2017-02

Participant	AM-S-W1704	z'-score	AM-S-W1705	z'-score	AM-S-W1706	z'-score	Method
**332**	0.138	-0.79	0.739	-1.48	0.238	-1.17	ND
**1003**	0.152	-0.29	0.831	-0.43	0.281	-0.05	GC-MS-MS
**1042**	0.169	0.30	0.934	0.73	0.305	0.57	GC-MS CI
**1629**	0.166	0.21	0.899	0.34	0.314	0.81	GC-MS-MS
**3112**	0.263	**3.69**	1.42	**6.21**	0.464	**4.70**	GC-MS CI
**4635**	0.149	-0.39	0.937	0.77	0.315	0.83	GC-MS EI
**6865**	0.197	1.32	0.918	0.56	0.290	0.18	GC
**7660**	0.156	-0.16	0.842	-0.30	0.272	-0.28	GC-MS CI
**7828**	0.169	0.32	0.899	0.34	0.293	0.26	GC-MS EI
**8559**	0.156	-0.14	0.665	**-2.32**	0.245	-0.99	ND
**8916**	0.165	0.18	0.868	-0.01	0.273	-0.26	GC-MS EI
**9301**	0.0626	**-3.49**	0.332	**-6.10**	0.107	**-4.57**	GC-MS-MS

	Assigned value	Standard uncertainty	σ pt	Acceptable range	K-S (Lilliefors)	Species
**AM-S-W1704**	0.160	0.00533	0.0274	0.104 - 0.216	Rejected[1]	---
**AM-S-W1705**	0.869*	0.0305	0.0826	0.693 - 1.05	Rejected[1]	---
**AM-S-W1706**	0.283	0.0135	0.0360	0.206 - 0.360	Rejected[1]	---

* The assigned value obtained exceeds the range of concentration from our scope of accreditation.

Statistics
Serum PCB IUPAC # 74 (µg/L)

All methods	AM-S-W1704	AM-S-W1705	AM-S-W1706
N	12	12	12
Robust mean Algo A	0.160	0.869	0.283
Robust STDev	0.0148	0.0845	0.0374
Median	0.161	0.884	0.286
STDev from MAD	0.0126	0.0760	0.0355
Arithmetic mean	0.162	0.857	0.283
STDev	0.0451	0.245	0.0798
CV or Variability	9.2%	9.7%	13.2%

GC-MS CI	AM-S-W1704	AM-S-W1705	AM-S-W1706
N	3	3	3
Robust mean Algo A	0.174	0.971	0.318
Robust STDev	0.0243	0.171	0.0608
Median	0.169	0.934	0.305
STDev from MAD	0.0193	0.135	0.0482
Arithmetic mean	0.196	1.06	0.347
STDev	0.0587	0.308	0.103
CV or Variability	14.0%	17.6%	19.1%

GC-MS EI	AM-S-W1704	AM-S-W1705	AM-S-W1706
N	3	3	3
Robust mean Algo A	0.163	0.901	0.294
Robust STDev	0.00749	0.0392	0.0238
Median	0.165	0.899	0.293
STDev from MAD	0.00593	0.0460	0.0297
Arithmetic mean	0.161	0.901	0.294
STDev	0.0106	0.0346	0.0210
CV or Variability	4.6%	4.3%	8.1%

GC-MS-MS	AM-S-W1704	AM-S-W1705	AM-S-W1706
N	3	3	3
Robust mean Algo A	0.146	0.803	0.268
Robust STDev	0.0262	0.127	0.0618
Median	0.152	0.831	0.281
STDev from MAD	0.0208	0.101	0.0489
Arithmetic mean	0.127	0.687	0.234
STDev	0.0561	0.310	0.111
CV or Variability	17.9%	15.8%	23.1%

When fewer than 20 results were considered for statistical treatment of all or a sub-sample of results, the accuracy of statistical data may be questionable.

Distribution

Serum PCB IUPAC # 74 (µg/L)

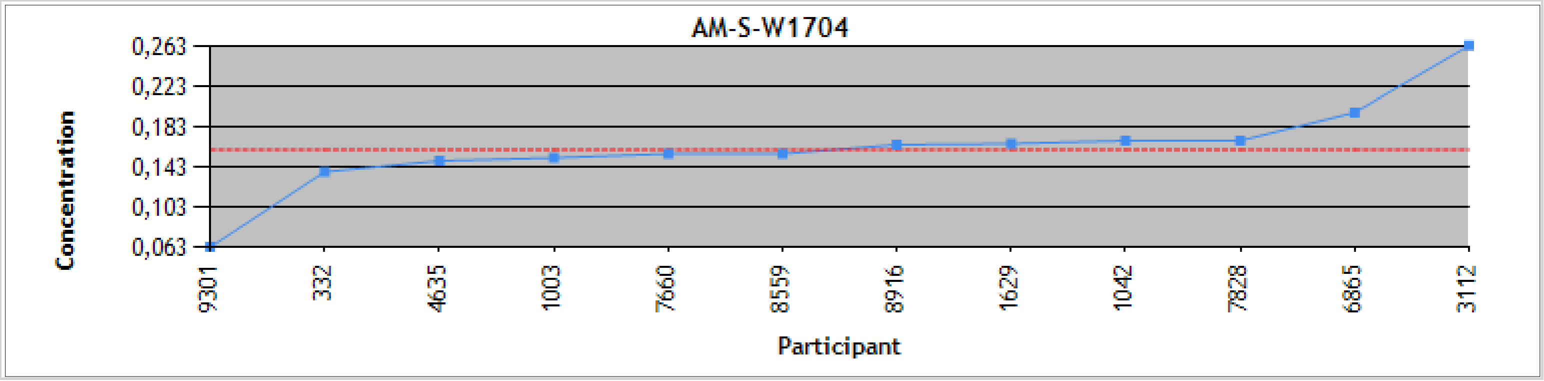

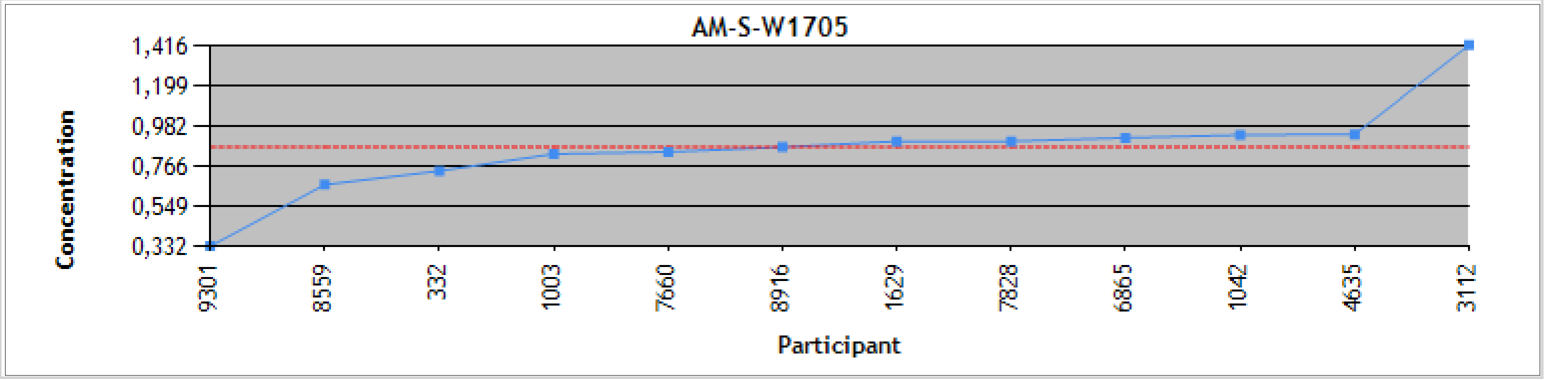

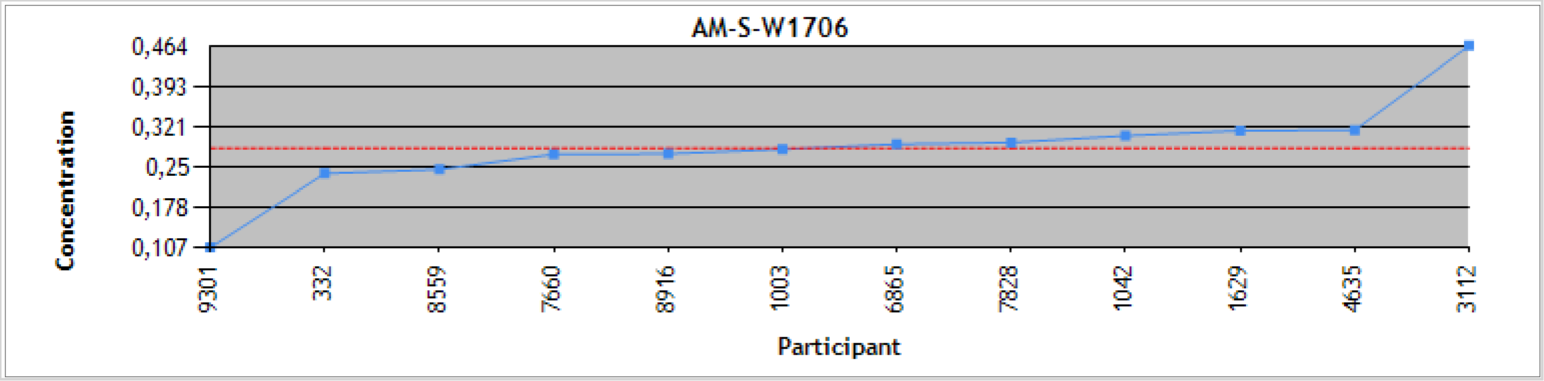

Individual results

Serum PCB IUPAC # 99 (µg/L)

Round #2017-02

Participant	AM-S-W1704	z'-score	AM-S-W1705	z'-score	AM-S-W1706	z'-score	Method
**270**	0.105	-0.92	0.735	0.57	0.281	0.02	GC-MS EI
**332**	0.111	-0.63	0.652	-0.36	0.262	-0.44	ND
**1003**	0.129	0.24	0.680	-0.04	0.321	1.01	GC-MS-MS
**1042**	0.125	0.03	0.701	0.19	0.271	-0.22	GC-MS CI
**1629**	0.116	-0.39	0.611	-0.82	0.241	-0.96	GC-MS-MS
**3112**	0.199	**3.63**	1.01	**3.63**	0.469	**4.67**	GC-MS CI
**4635**	0.127	0.15	0.697	0.15	0.279	-0.02	GC-MS EI
**6865**	0.123	-0.05	0.775	1.02	0.313	0.81	GC
**7660**	0.122	-0.08	0.654	-0.34	0.260	-0.50	GC-MS CI
**7828**	0.148	1.16	0.817	1.49	0.331	1.26	GC-MS EI
**8559**	0.153	1.40	0.644	-0.45	0.287	0.17	ND
**8916**	0.106	-0.87	0.582	-1.14	0.233	-1.16	GC-MS EI
**9301**	0.0552	**-3.33**	0.267	**-4.68**	0.113	**-4.12**	GC-MS-MS

	Assigned value	Standard uncertainty	σ pt	Acceptable range	K-S (Lilliefors)	Species
**AM-S-W1704**	0.124	0.00683	0.0194	0.0828 - 0.165	Accepted	---
**AM-S-W1705**	0.684	0.0310	0.0835	0.506 - 0.862	Accepted	---
**AM-S-W1706**	0.280	0.0158	0.0373	0.199 - 0.361	Accepted	---

Statistics

Serum PCB IUPAC # 99 (µg/L)

All methods	AM-S-W1704	AM-S-W1705	AM-S-W1706
N	13	13	13
Robust mean Algo A	0.124	0.684	0.280
Robust STDev	0.0197	0.0894	0.0454
Median	0.123	0.680	0.279
STDev from MAD	0.0178	0.0816	0.0504
Arithmetic mean	0.125	0.679	0.282
STDev	0.0326	0.165	0.0783
CV or Variability	15.9%	13.1%	16.2%

GC-MS CI	AM-S-W1704	AM-S-W1705	AM-S-W1706
N	3	3	3
Robust mean Algo A	0.126	0.720	0.276
Robust STDev	0.00430	0.0876	0.0215
Median	0.125	0.701	0.271
STDev from MAD	0.00341	0.0694	0.0171
Arithmetic mean	0.149	0.787	0.333
STDev	0.0436	0.192	0.118
CV or Variability	3.4%	12.2%	7.8%

GC-MS EI	AM-S-W1704	AM-S-W1705	AM-S-W1706
N	4	4	4
Robust mean Algo A	0.120	0.708	0.281
Robust STDev	0.0197	0.110	0.0454
Median	0.117	0.716	0.280
STDev from MAD	0.0163	0.0890	0.0356
Arithmetic mean	0.122	0.708	0.281
STDev	0.0204	0.0977	0.0400
CV or Variability	16.5%	15.6%	16.2%

GC-MS-MS	AM-S-W1704	AM-S-W1705	AM-S-W1706
N	3	3	3
Robust mean Algo A	0.111	0.583	0.225
Robust STDev	0.0243	0.129	0.119
Median	0.116	0.611	0.241
STDev from MAD	0.0193	0.102	0.119
Arithmetic mean	0.100	0.519	0.225
STDev	0.0394	0.221	0.105
CV or Variability	22.0%	22.2%	52.9%

When fewer than 20 results were considered for statistical treatment of all or a sub-sample of results, the accuracy of statistical data may be questionable.

Distribution

Serum PCB IUPAC # 99 (µg/L)

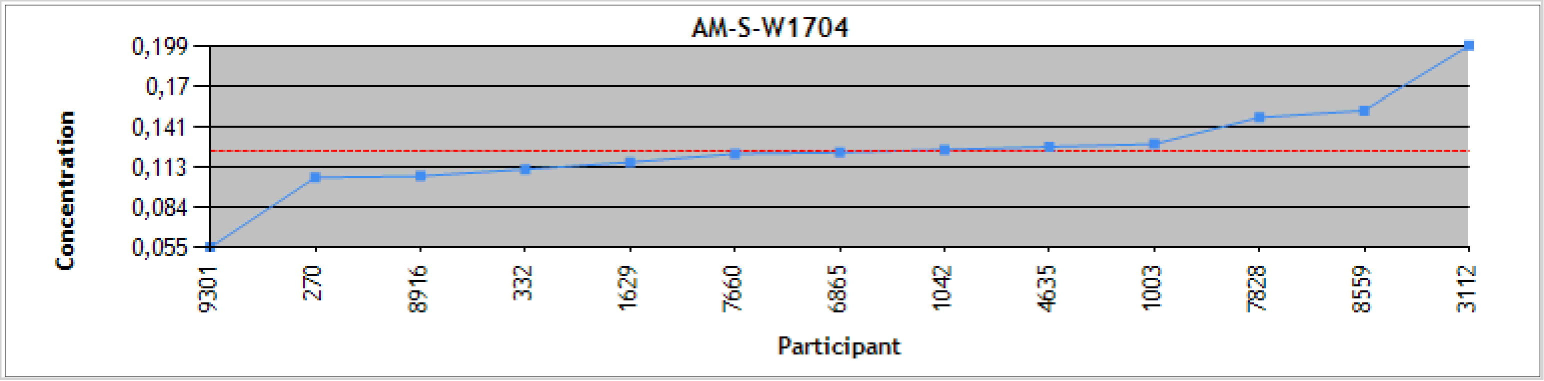

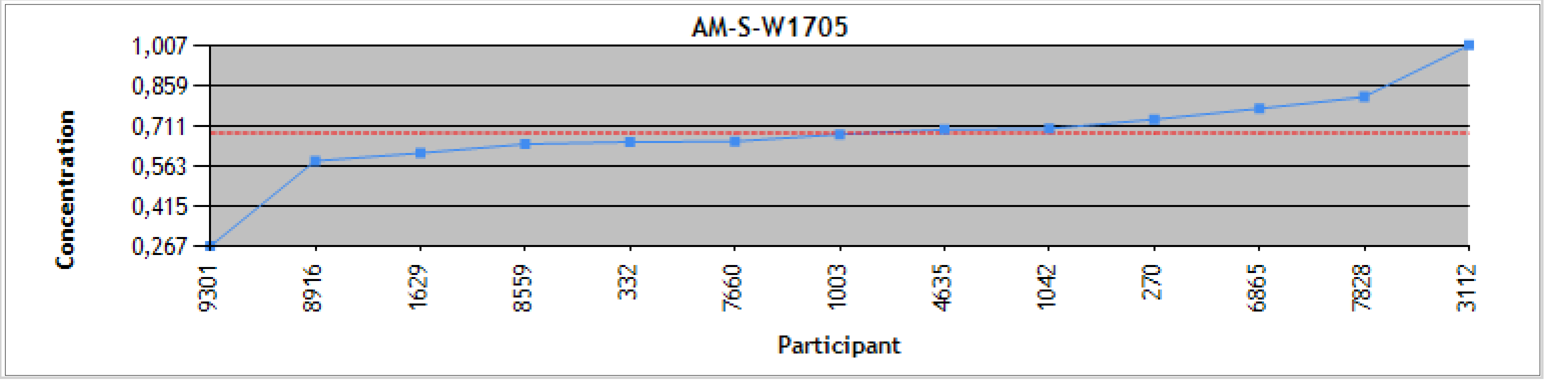

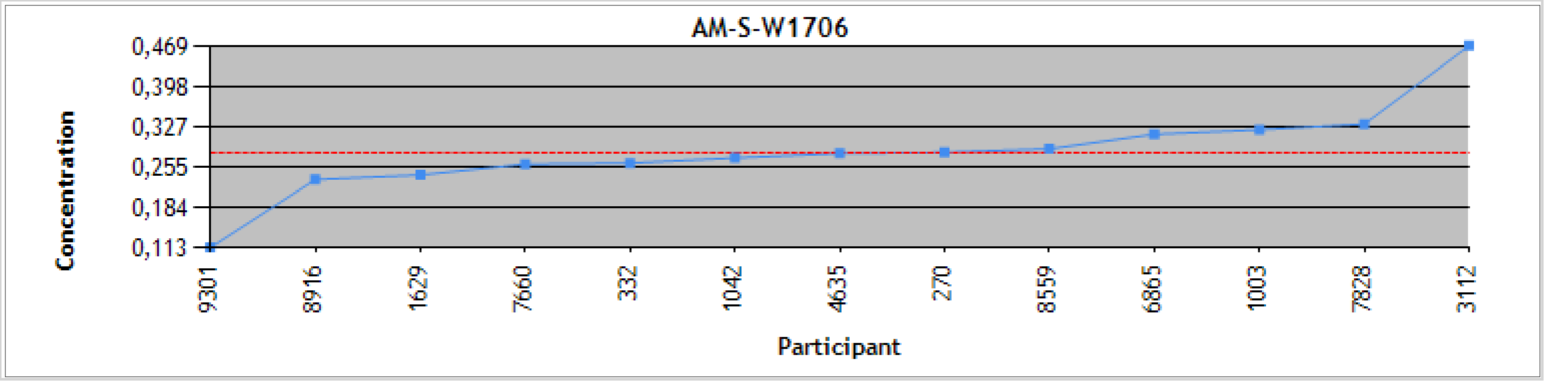

Individual results

Serum PCB IUPAC # 101 (µg/L)

Round #2017-02

Participant	AM-S-W1704	z'-score	AM-S-W1705	z'-score	AM-S-W1706	z'-score	Method
**270**	0.279	0.67	0.655	0.57	1.34	0.69	GC-MS EI
**332**	0.207	-0.75	0.560	-0.23	1.16	-0.04	ND
**748**	0.280	0.69	0.548	-0.33	1.27	0.41	ND
**1042**	0.254	0.17	0.634	0.39	1.23	0.24	GC-MS CI
**1629**	0.226	-0.38	0.544	-0.36	0.995	-0.73	GC-MS-MS
**2446**	0.231	-0.28	0.541	-0.38	1.12	-0.22	GC-MS EI
**3112**	0.334	1.76	0.817	1.92	1.67	**2.05**	GC-MS CI
**6865**	0.268	0.45	0.633	0.38	1.14	-0.12	GC
**7660**	0.239	-0.12	0.599	0.10	1.16	-0.03	GC-MS CI
**7828**	0.244	-0.02	0.611	0.20	1.23	0.25	GC-MS EI
**8559**	0.262	0.34	0.540	-0.39	1.00	-0.70	ND
**8916**	0.212	-0.65	0.514	-0.61	1.04	-0.53	GC-MS EI
**9301**	0.0980	**-2.91**	0.238	**-2.91**	0.486	**-2.84**	GC-MS-MS
**9426**	<LD	---	2.10	**12.62**	2.20	**4.27**	GC-MS-MS

	Assigned value	Standard uncertainty	σ pt	Acceptable range	K-S (Lilliefors)	Species
**AM-S-W1704**	0.245	0.0124	0.0491	0.144 - 0.346	Accepted	---
**AM-S-W1705**	0.587	0.0244	0.117	0.347 - 0.827	Rejected[1]	---
**AM-S-W1706**	1.17	0.0586	0.235	0.686 - 1.65	Rejected[1]	---

PCB IUPAC # 101 is not included in the scope of our accreditation.

Statistics
Serum PCB IUPAC # 101 (µg/L)

All methods	AM-S-W1704	AM-S-W1705	AM-S-W1706
N	13	14	14
Robust mean Algo A	0.245	0.587	1.17
Robust STDev	0.0359	0.0729	0.175
Median	0.244	0.579	1.16
STDev from MAD	0.0356	0.0690	0.168
Arithmetic mean	0.241	0.681	1.22
STDev	0.0546	0.426	0.379
CV or Variability	14.6%	12.4%	15.0%

GC-MS CI	AM-S-W1704	AM-S-W1705	AM-S-W1706
N	6	3	3
Robust mean Algo A	0.260	0.649	1.26
Robust STDev	0.0248	0.0664	0.125
Median	0.254	0.634	1.23
STDev from MAD	0.0219	0.0526	0.0994
Arithmetic mean	0.275	0.683	1.35
STDev	0.0458	0.117	0.273
CV or Variability	9.5%	10.2%	10.0%

GC-MS EI	AM-S-W1704	AM-S-W1705	AM-S-W1706
N	8	4	4
Robust mean Algo A	0.240	0.580	1.18
Robust STDev	0.0269	0.0731	0.146
Median	0.238	0.576	1.17
STDev from MAD	0.0237	0.0719	0.139
Arithmetic mean	0.242	0.580	1.18
STDev	0.0261	0.0645	0.129
CV or Variability	11.2%	12.6%	12.4%

GC-MS-MS	AM-S-W1704	AM-S-W1705	AM-S-W1706
N	4	3	3
Robust mean Algo A	0.162	0.669	1.20
Robust STDev	0.0838	0.573	0.953
Median	0.162	0.544	0.995
STDev from MAD	0.0949	0.454	0.755
Arithmetic mean	0.162	0.961	1.23
STDev	0.0739	0.998	0.880
CV or Variability	51.7%	85.6%	79.2%

When fewer than 20 results were considered for statistical treatment of all or a sub-sample of results, the accuracy of statistical data may be questionable.

Distribution

Serum PCB IUPAC # 101 (µg/L)

Distribution graph for PCB IUPAC # 101 in material AM-S-W1704 is not available.

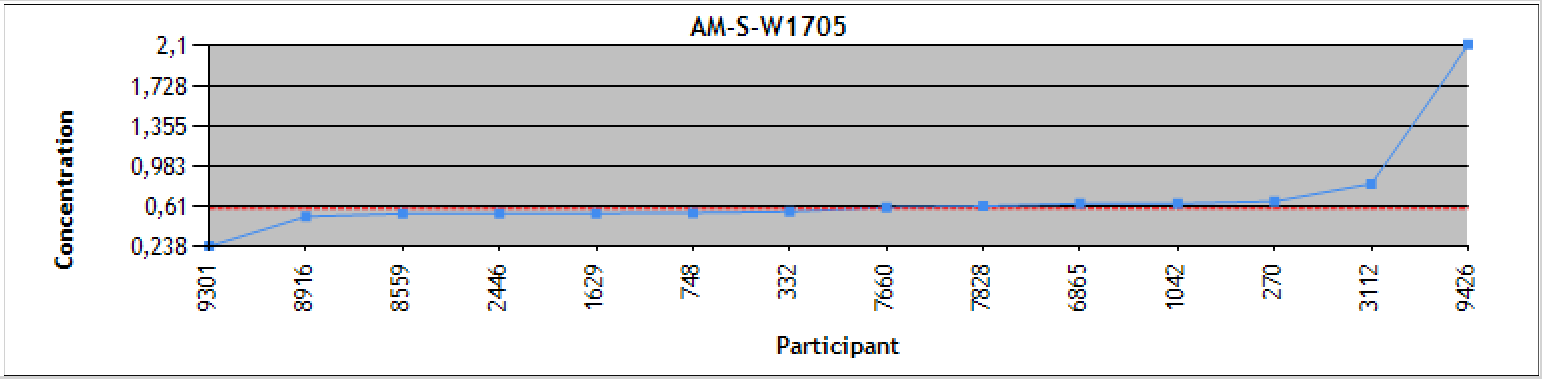

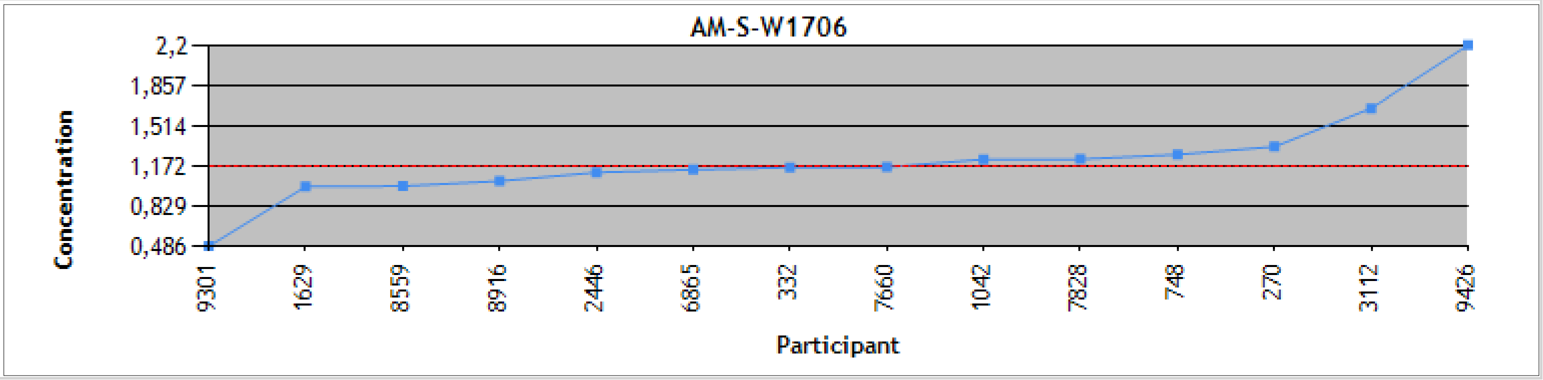

Individual results

Serum PCB IUPAC # 105 (µg/L)

Round #2017-02

Participant	AM-S-W1704	z'-score	AM-S-W1705	z'-score	AM-S-W1706	z'-score	Method
**270**	0.0810	0.82	0.0280	**-6.52**	0.102	-1.06	GC-MS EI
**332**	0.0690	-0.16	0.224	0.43	0.124	0.11	ND
**1042**	0.0742	0.27	0.239	0.94	0.129	0.39	GC-MS CI
**1629**	0.0690	-0.16	0.248	1.28	0.136	0.74	GC-MS-MS
**2446**	0.0640	-0.56	0.179	-1.17	0.106	-0.85	GC-MS EI
**3112**	0.0510	-1.62	0.159	-1.88	0.0890	-1.75	GC-MS CI
**4635**	0.0720	0.09	0.227	0.53	0.130	0.42	GC-MS EI
**6865**	0.0911	1.65	0.242	1.06	0.159	1.97	GC
**7660**	0.0705	-0.04	0.235	0.83	0.127	0.25	GC-MS CI
**7828**	0.0700	-0.07	0.224	0.43	0.125	0.16	GC-MS EI
**8559**	0.0740	0.25	0.179	-1.17	0.110	-0.64	ND
**8916**	0.0770	0.50	0.250	1.35	0.192	**3.72**	GC-MS EI
**9301**	0.0313	**-3.23**	0.103	**-3.86**	0.0545	**-3.59**	GC-MS-MS

	Assigned value	Standard uncertainty	σ pt	Acceptable range	K-S (Lilliefors)	Species
**AM-S-W1704**	0.0709	0.00226	0.0120	0.0464 - 0.0954	Accepted	---
**AM-S-W1705**	0.212	0.0128	0.0252	0.156 - 0.268	Accepted	---
**AM-S-W1706**	0.122	0.00852	0.0168	0.0843 - 0.160	Accepted	---

Statistics

Serum PCB IUPAC # 105 (µg/L)

All methods	AM-S-W1704	AM-S-W1705	AM-S-W1706
N	13	13	13
Robust mean Algo A	0.0709	0.212	0.122
Robust STDev	0.00652	0.0369	0.0246
Median	0.0705	0.224	0.125
STDev from MAD	0.00559	0.0356	0.0222
Arithmetic mean	0.0688	0.195	0.122
STDev	0.0145	0.0661	0.0330
CV or Variability	9.2%	17.4%	20.1%

GC-MS CI	AM-S-W1704	AM-S-W1705	AM-S-W1706
N	3	3	3
Robust mean Algo A	0.0689	0.234	0.126
Robust STDev	0.00706	0.00599	0.00505
Median	0.0705	0.235	0.127
STDev from MAD	0.00559	0.00475	0.00400
Arithmetic mean	0.0652	0.211	0.115
STDev	0.0125	0.0451	0.0226
CV or Variability	10.2%	2.6%	4.0%

GC-MS EI	AM-S-W1704	AM-S-W1705	AM-S-W1706
N	5	5	5
Robust mean Algo A	0.0728	0.209	0.126
Robust STDev	0.00741	0.0400	0.0294
Median	0.0720	0.224	0.125
STDev from MAD	0.00742	0.0386	0.0282
Arithmetic mean	0.0728	0.182	0.131
STDev	0.00653	0.0896	0.0361
CV or Variability	10.2%	19.1%	23.4%

When fewer than 20 results were considered for statistical treatment of all or a sub-sample of results, the accuracy of statistical data may be questionable.

Distribution

Serum PCB IUPAC # 105 (µg/L)

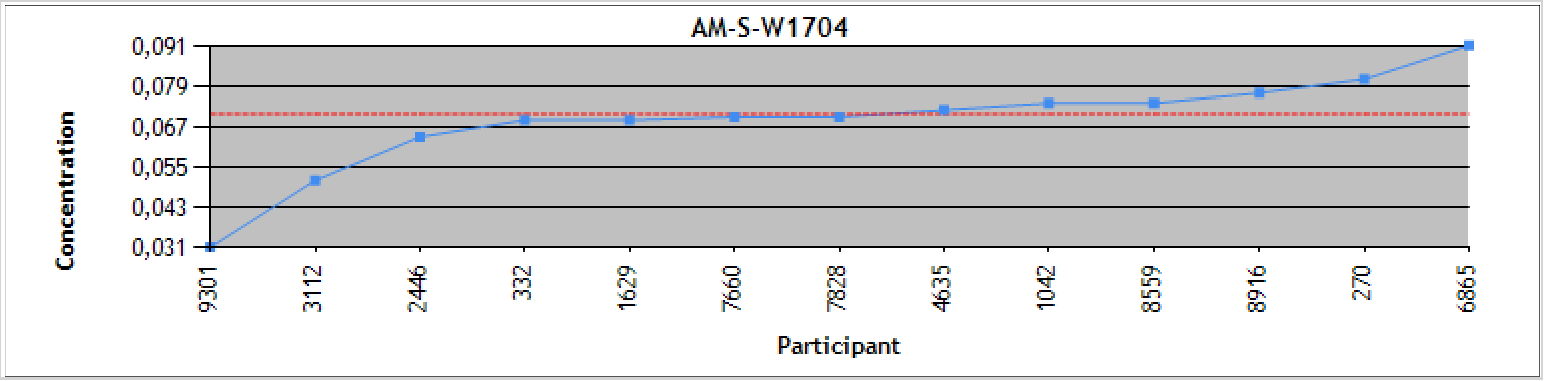

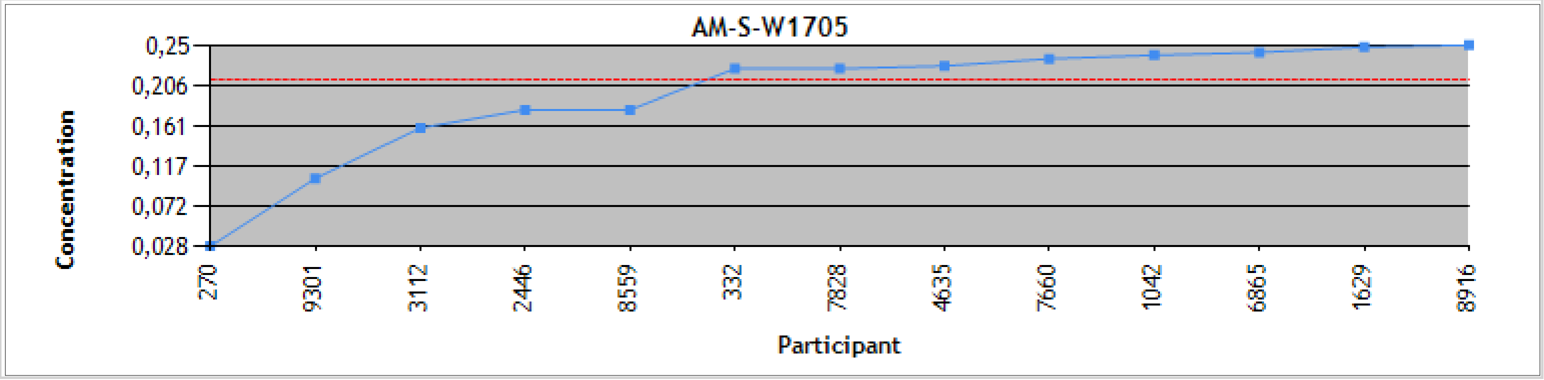

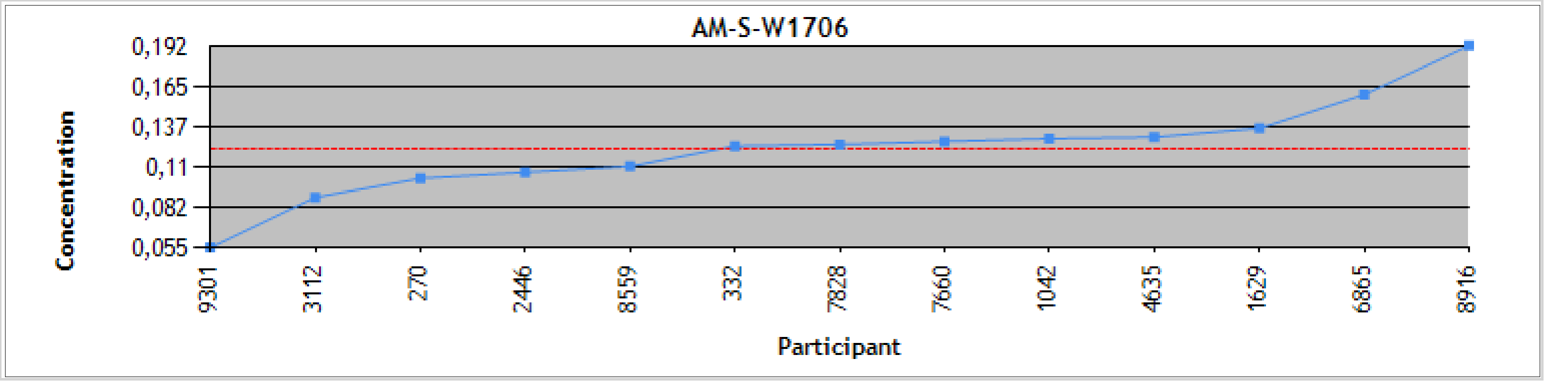

Individual results

Serum PCB IUPAC # 118 (µg/L)

Round #2017-02

Participant	AM-S-W1704	z'-score	AM-S-W1705	z'-score	AM-S-W1706	z'-score	Method
**270**	0.121	-0.26	0.416	-0.25	0.314	1.17	GC-MS EI
**332**	0.121	-0.26	0.440	0.15	0.258	-0.29	ND
**748**	0.141	0.79	0.405	-0.43	0.339	1.82	ND
**1003**	0.139	0.69	0.487	0.92	0.295	0.68	GC-MS-MS
**1042**	0.132	0.31	0.461	0.49	0.276	0.19	GC-MS CI
**1629**	0.116	-0.53	0.472	0.67	0.286	0.44	GC-MS-MS
**2446**	0.113	-0.69	0.338	-1.52	0.229	-1.04	GC-MS EI
**3112**	0.0750	**-2.69**	0.251	**-2.95**	0.152	**-3.04**	GC-MS CI
**4635**	0.131	0.26	0.449	0.29	0.282	0.34	GC-MS EI
**6865**	0.150	1.26	0.478	0.77	0.287	0.47	GC
**7660**	0.130	0.19	0.461	0.49	0.275	0.15	GC-MS CI
**7828**	0.134	0.42	0.458	0.44	0.282	0.34	GC-MS EI
**8420**	<LQ	---	0.500	1.13	0.220	-1.27	ND
**8559**	0.137	0.58	0.405	-0.43	0.258	-0.29	ND
**8916**	0.114	-0.63	0.368	-1.03	0.219	-1.30	GC-MS EI
**9301**	0.0419	**-4.43**	0.170	**-4.27**	0.103	**-4.32**	GC-MS-MS
**9426**	<LD	---	<LD	---	<LD	---	GC-MS-MS

	Assigned value	Standard uncertainty	σ pt	Acceptable range	K-S (Lilliefors)	Species
**AM-S-W1704**	0.126	0.00459	0.0184	0.0880 - 0.164	Accepted	---
**AM-S-W1705**	0.431	0.0169	0.0586	0.309 - 0.553	Accepted	---
**AM-S-W1706**	0.269	0.0101	0.0371	0.192 - 0.346	Accepted	---

Statistics
Serum PCB IUPAC # 118 (µg/L)

All methods	AM-S-W1704	AM-S-W1705	AM-S-W1706
N	15	16	16
Robust mean Algo A	0.126	0.431	0.269
Robust STDev	0.0142	0.0540	0.0324
Median	0.130	0.445	0.276
STDev from MAD	0.0139	0.0541	0.0274
Arithmetic mean	0.120	0.410	0.255
STDev	0.0277	0.0903	0.0596
CV or Variability	11.3%	12.5%	12.0%

GC-MS CI	AM-S-W1704	AM-S-W1705	AM-S-W1706
N	3	3	3
Robust mean Algo A	0.129	0.461	0.274
Robust STDev	0.00430	0.000562	0.00299
Median	0.130	0.461	0.275
STDev from MAD	0.00341	0.000445	0.00237
Arithmetic mean	0.112	0.391	0.234
STDev	0.0322	0.121	0.0714
CV or Variability	3.3%	0.1%	1.1%

GC-MS EI	AM-S-W1704	AM-S-W1705	AM-S-W1706
N	5	5	5
Robust mean Algo A	0.123	0.406	0.265
Robust STDev	0.0109	0.0587	0.0453
Median	0.121	0.416	0.282
STDev from MAD	0.0119	0.0623	0.0475
Arithmetic mean	0.123	0.406	0.265
STDev	0.00961	0.0518	0.0400
CV or Variability	8.9%	14.5%	17.1%

GC-MS-MS	AM-S-W1704	AM-S-W1705	AM-S-W1706
N	3	3	3
Robust mean Algo A	0.107	0.466	0.282
Robust STDev	0.0430	0.0281	0.0168
Median	0.116	0.472	0.286
STDev from MAD	0.0341	0.0222	0.0133
Arithmetic mean	0.0990	0.376	0.228
STDev	0.0507	0.179	0.108
CV or Variability	40.4%	6.0%	6.0%

When fewer than 20 results were considered for statistical treatment of all or a sub-sample of results, the accuracy of statistical data may be questionable.

Distribution

Serum PCB IUPAC # 118 (µg/L)

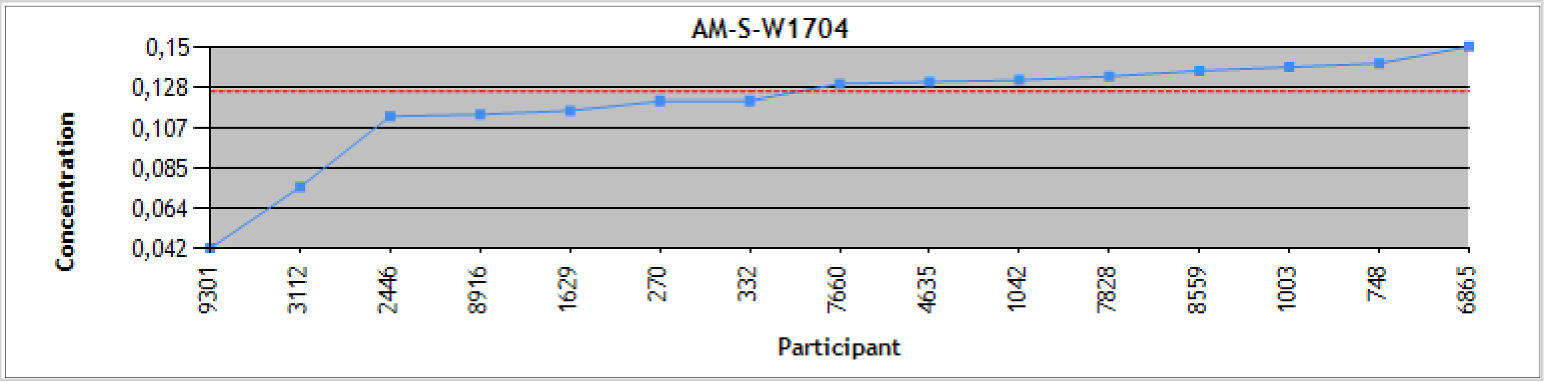

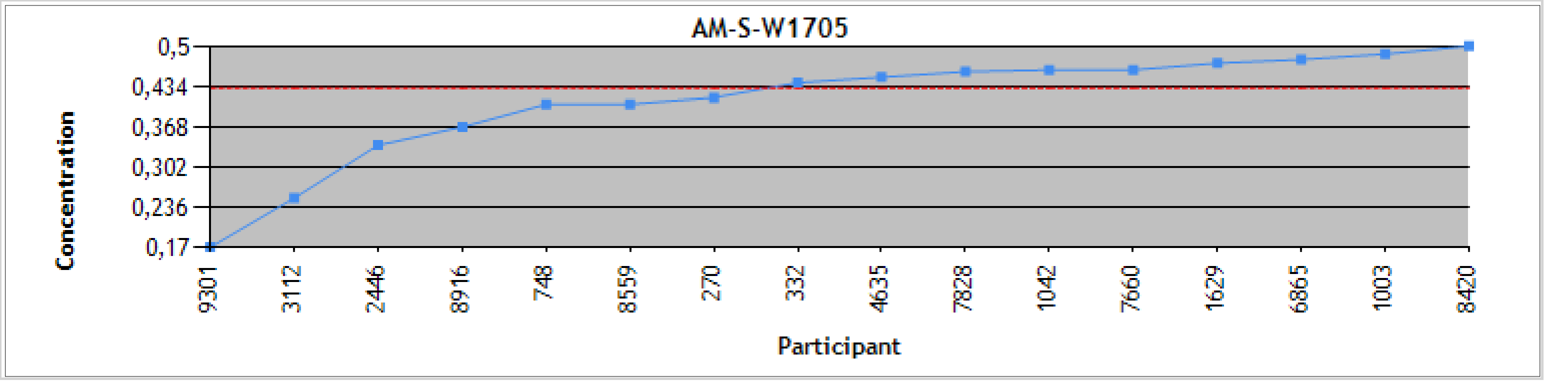

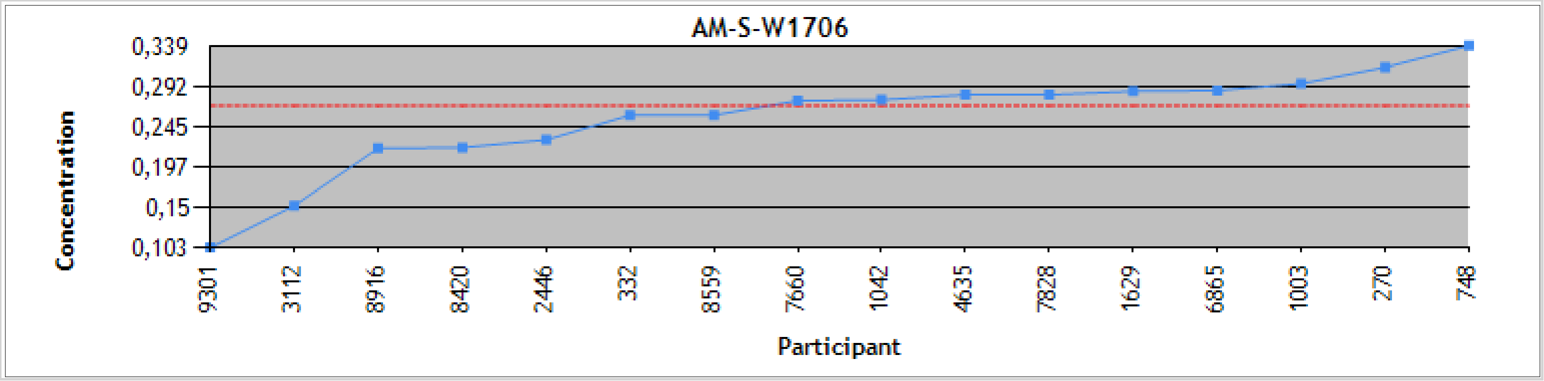

Individual results

Serum PCB IUPAC # 138 (µg/L)

Round #2017-02

Participant	AM-S-W1704	z'-score	AM-S-W1705	z'-score	AM-S-W1706	z'-score	Method
**270**	0.500	**2.57**	2.62	**3.62**	1.29	**3.71**	GC-MS EI
**332**	0.471	**2.13**	2.30	**2.48**	1.10	**2.39**	ND
**748**	0.374	0.64	1.54	-0.21	0.944	1.28	ND
**1003**	0.337	0.08	1.75	0.53	0.822	0.41	GC-MS-MS
**1042**	0.309	-0.35	1.47	-0.45	0.673	-0.65	GC-MS CI
**1629**	0.285	-0.72	1.35	-0.89	0.683	-0.59	GC-MS-MS
**2446**	0.270	-0.95	1.24	-1.27	0.626	-0.99	GC-MS EI
**3112**	0.251	-1.24	1.10	-1.77	0.524	-1.72	GC-MS CI
**4635**	0.323	-0.14	1.50	-0.35	0.745	-0.14	GC-MS EI
**6865**	0.322	-0.15	1.71	0.39	0.770	0.04	GC
**7660**	0.299	-0.51	1.65	0.18	0.748	-0.12	GC-MS CI
**8420**	0.280	-0.80	1.29	-1.10	0.550	-1.53	ND
**8559**	0.414	1.25	2.28	**2.41**	0.867	0.73	ND
**8916**	0.534	**3.09**	2.58	**3.49**	1.19	**3.00**	GC-MS EI
**9301**	0.118	**-3.27**	0.549	**-3.73**	0.233	**-3.80**	GC-MS-MS
**9426**	<LD	---	<ld	---	<ld	---	GC-MS-MS

	Assigned value	Standard uncertainty	σ pt	Acceptable range	K-S (Lilliefors)	Species
**AM-S-W1704**	0.332	0.0267	0.0597	0.201 - 0.463	Accepted	---
**AM-S-W1705**	1.60	0.137	0.246	1.04 - 2.16	Accepted	---
**AM-S-W1706**	0.765	0.0663	0.123	0.485 - 1.05	Accepted	---

PCB IUPAC # 163 was added to all PTM with the following levels: AM-S-W1704 = 0.10 µg/L
AM-S-W1705 = 0.56 µg/L
AM-S-W1706 = 0.26 µg/L

Statistics
Serum PCB IUPAC # 138 (µg/L)

All methods	AM-S-W1704	AM-S-W1705	AM-S-W1706
N	15	15	15
Robust mean Algo A	0.332	1.60	0.765
Robust STDev	0.0827	0.425	0.205
Median	0.322	1.54	0.748
STDev from MAD	0.0771	0.371	0.180
Arithmetic mean	0.339	1.66	0.784
STDev	0.107	0.574	0.270
CV or Variability	24.9%	26.6%	26.8%

GC-MS CI	AM-S-W1704	AM-S-W1705	AM-S-W1706
N	3	3	3
Robust mean Algo A	0.295	1.41	0.648
Robust STDev	0.0185	0.317	0.129
Median	0.299	1.47	0.673
STDev from MAD	0.0147	0.261	0.110
Arithmetic mean	0.286	1.41	0.648
STDev	0.0309	0.280	0.114
CV or Variability	6.3%	22.5%	19.9%

GC-MS EI	AM-S-W1704	AM-S-W1705	AM-S-W1706
N	4	4	4
Robust mean Algo A	0.407	1.99	0.960
Robust STDev	0.147	0.814	0.367
Median	0.412	2.04	0.965
STDev from MAD	0.156	0.830	0.400
Arithmetic mean	0.407	1.99	0.960
STDev	0.130	0.718	0.324
CV or Variability	36.2%	41.0%	38.2%

GC-MS-MS	AM-S-W1704	AM-S-W1705	AM-S-W1706
N	3	3	3
Robust mean Algo A	0.264	1.22	0.626
Robust STDev	0.0973	0.694	0.260
Median	0.285	1.35	0.683
STDev from MAD	0.0771	0.593	0.206
Arithmetic mean	0.247	1.22	0.579
STDev	0.114	0.612	0.308
CV or Variability	36.9%	57.0%	41.5%

When fewer than 20 results were considered for statistical treatment of all or a sub-sample of results, the accuracy of statistical data may be questionable.

Distribution

Serum PCB IUPAC # 138 (µg/L)

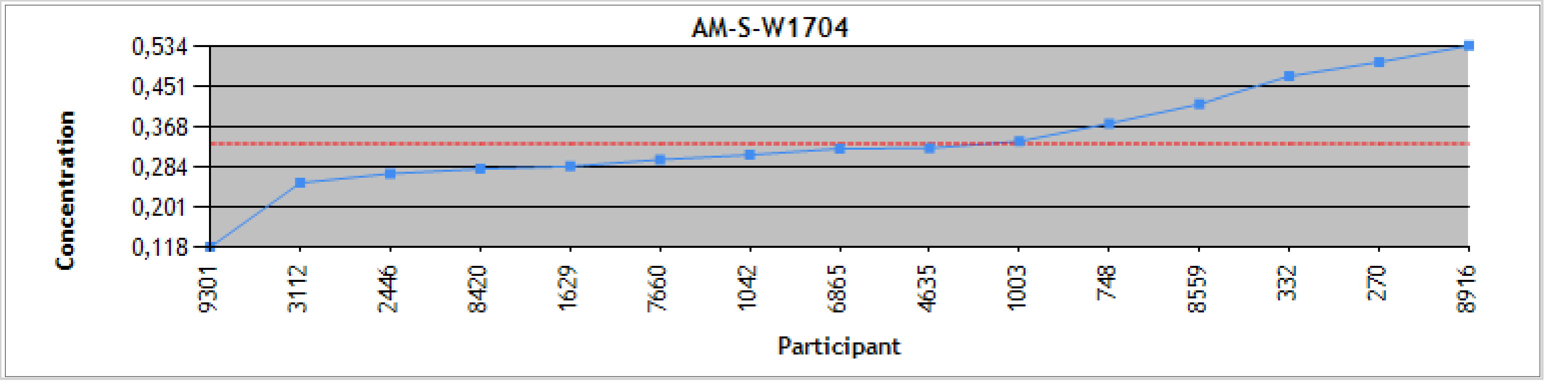

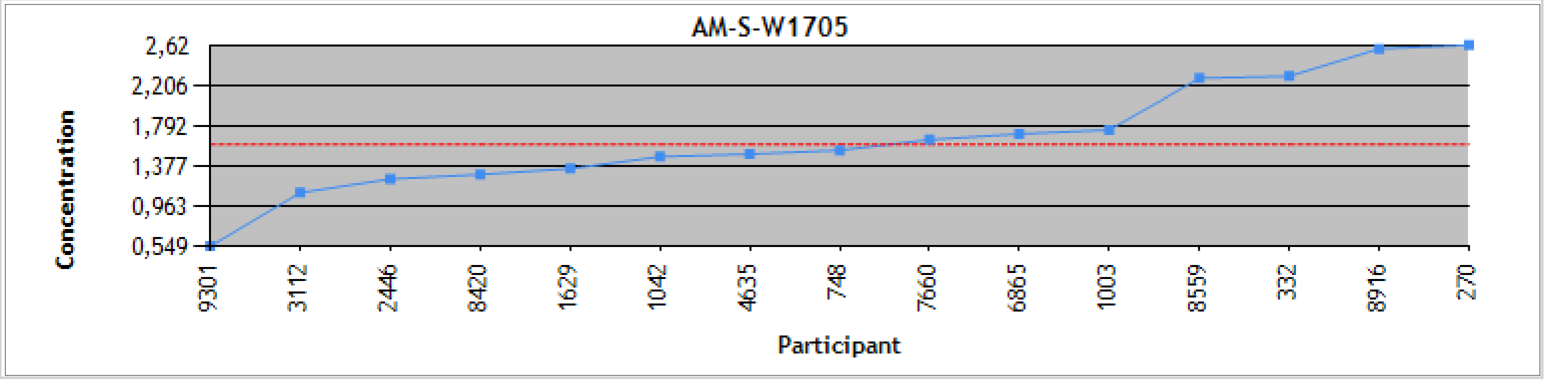

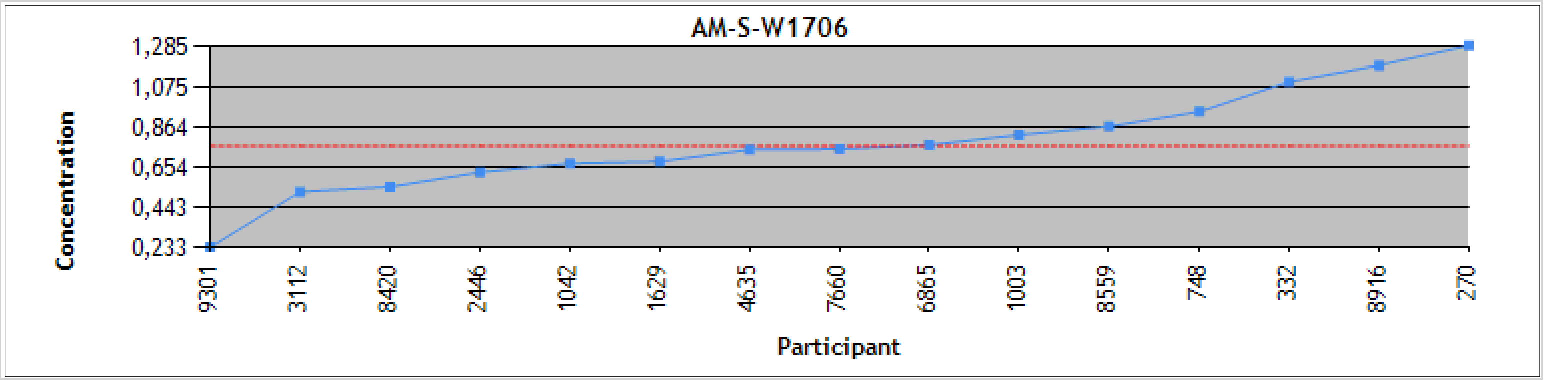

Individual results

Serum PCB IUPAC # 153 (µg/L)

Round #2017-02

Participant	AM-S-W1704	z'-score	AM-S-W1705	z'-score	AM-S-W1706	z'-score	Method
**270**	0.334	1.25	1.18	1.52	2.06	1.06	GC-MS EI
**332**	0.278	-0.07	0.875	-0.58	1.74	-0.15	ND
**748**	0.283	0.05	0.690	-1.86	1.67	-0.42	ND
**1003**	0.263	-0.42	0.922	-0.25	1.77	-0.04	GC-MS-MS
**1042**	0.291	0.23	1.01	0.33	1.88	0.40	GC-MS CI
**1629**	0.261	-0.47	0.882	-0.53	1.65	-0.50	GC-MS-MS
**2446**	0.260	-0.50	0.828	-0.90	1.63	-0.56	GC-MS EI
**3112**	0.187	**-2.22**	0.603	**-2.46**	1.13	**-2.52**	GC-MS CI
**4635**	0.295	0.33	1.02	0.46	1.98	0.77	GC-MS EI
**6865**	0.311	0.71	0.997	0.27	1.69	-0.34	GC
**7660**	0.269	-0.29	0.960	0.01	1.83	0.19	GC-MS CI
**7828**	0.257	-0.57	0.876	-0.57	1.63	-0.58	GC-MS EI
**8420**	0.320	0.92	1.02	0.43	1.90	0.46	ND
**8559**	0.291	0.24	1.18	1.54	1.76	-0.08	ND
**8916**	0.441	**3.77**	1.49	**3.69**	2.70	**3.53**	GC-MS EI
**9301**	0.119	**-3.82**	0.436	**-3.62**	0.750	**-3.97**	GC-MS-MS
**9426**	***2.00***	**40.55**	4.00	**21.11**	4.00	**8.56**	GC-MS-MS

	Assigned value	Standard uncertainty	σ pt	Acceptable range	K-S (Lilliefors)	Species
**AM-S-W1704**	0.281	0.0103	0.0412	0.196 - 0.366	Accepted	---
**AM-S-W1705**	0.958	0.0469	0.136	0.670 - 1.25	Rejected[1]	---
**AM-S-W1706**	1.78	0.0628	0.251	1.26 - 2.30	Rejected[1]	---

PCB IUPAC # 132 was added to all PTM with the following levels: AM-S-W1704 = 0.10 µg/L
AM-S-W1705 = 0.32 µg/L
AM-S-W1706 = 0.74 µg/L

Statistics

Serum PCB IUPAC # 153 (µg/L)

All methods	AM-S-W1704	AM-S-W1705	AM-S-W1706
N	16	17	17
Robust mean Algo A	0.281	0.958	1.78
Robust STDev	0.0329	0.155	0.207
Median	0.281	0.960	1.76
STDev from MAD	0.0297	0.125	0.187
Arithmetic mean	0.279	1.12	1.87
STDev	0.0674	0.780	0.678
CV or Variability	11.7%	16.1%	11.6%

GC-MS CI	AM-S-W1704	AM-S-W1705	AM-S-W1706
N	3	3	3
Robust mean Algo A	0.259	0.941	1.81
Robust STDev	0.0414	0.0852	0.105
Median	0.269	0.960	1.83
STDev from MAD	0.0328	0.0675	0.0830
Arithmetic mean	0.249	0.856	1.61
STDev	0.0546	0.220	0.422
CV or Variability	15.9%	9.1%	5.8%

GC-MS EI	AM-S-W1704	AM-S-W1705	AM-S-W1706
N	5	5	5
Robust mean Algo A	0.305	1.05	2.00
Robust STDev	0.0590	0.250	0.494
Median	0.295	1.02	1.98
STDev from MAD	0.0564	0.227	0.513
Arithmetic mean	0.317	1.08	2.00
STDev	0.0758	0.267	0.436
CV or Variability	19.3%	23.8%	24.7%

GC-MS-MS	AM-S-W1704	AM-S-W1705	AM-S-W1706
N	3	4	4
Robust mean Algo A	0.260	0.921	1.75
Robust STDev	0.00374	0.467	0.973
Median	0.261	0.902	1.71
STDev from MAD	0.00297	0.360	0.756
Arithmetic mean	0.214	1.56	2.04
STDev	0.0826	1.64	1.38
CV or Variability	1.4%	50.7%	55.5%

When fewer than 20 results were considered for statistical treatment of all or a sub-sample of results, the accuracy of statistical data may be questionable.

Distribution
Serum PCB IUPAC # 153 (µg/L)

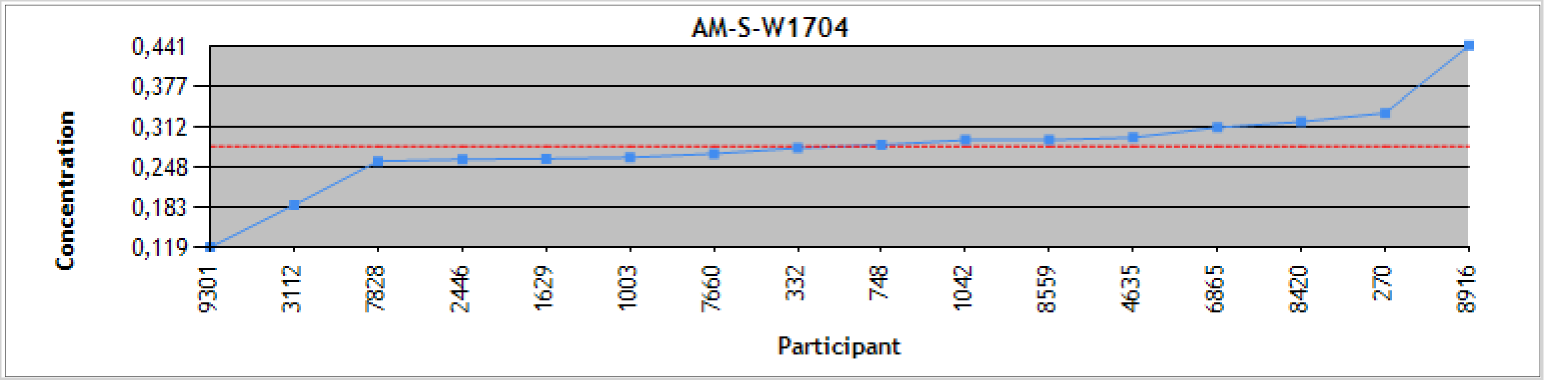

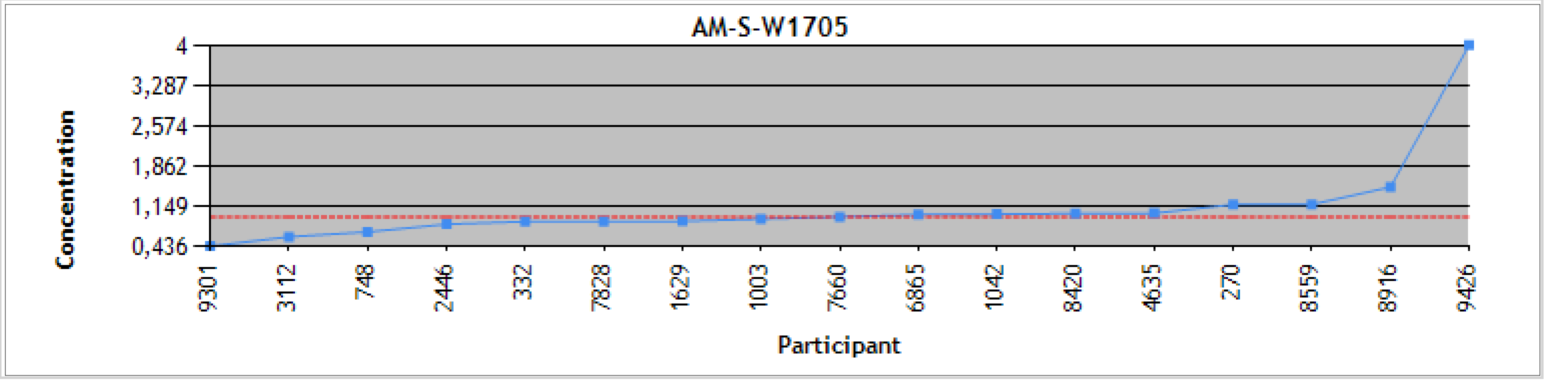

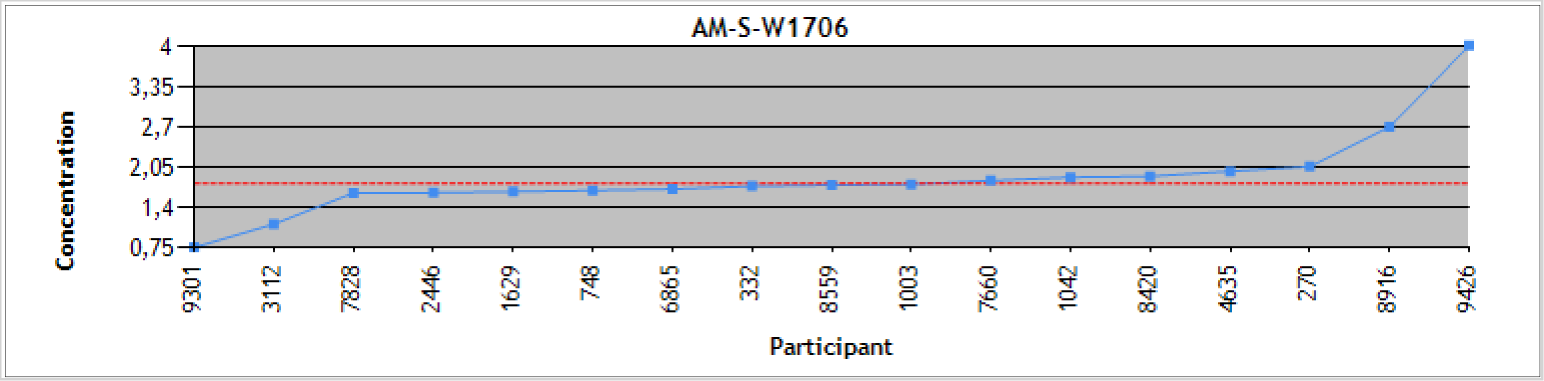

Individual results

Serum PCB IUPAC # 170 (µg/L)

Round #2017-02

Participant	AM-S-W1704	z'-score	AM-S-W1705	z'-score	AM-S-W1706	z'-score	Method
**332**	0.190	0.29	0.488	-0.14	0.291	-0.10	ND
**1003**	0.188	0.22	0.514	0.20	0.308	0.25	GC-MS-MS
**1042**	0.182	0.03	0.507	0.10	0.297	0.02	GC-MS CI
**1629**	0.172	-0.29	0.468	-0.41	0.297	0.02	GC-MS-MS
**3112**	0.167	-0.45	0.455	-0.58	0.272	-0.50	GC-MS CI
**4635**	0.182	0.03	0.501	0.03	0.293	-0.06	GC-MS EI
**6865**	0.196	0.48	0.560	0.80	0.323	0.56	GC
**7660**	0.180	-0.04	0.515	0.21	0.301	0.10	GC-MS CI
**7828**	0.171	-0.32	0.461	-0.50	0.271	-0.52	GC-MS EI
**8559**	0.205	0.77	0.662	**2.13**	0.415	**2.48**	ND
**8916**	0.160	-0.67	0.423	-1.00	0.243	-1.11	GC-MS EI
**9301**	0.0700	**-3.55**	0.183	**-4.14**	0.115	**-3.78**	GC-MS-MS
**9426**	0.900	**23.00**	1.00	**6.56**	0.500	**4.26**	GC-MS-MS

	Assigned value	Standard uncertainty	σ pt	Acceptable range	K-S (Lilliefors)	Species
**AM-S-W1704**	0.181	0.00616	0.0307	0.118 - 0.244	Rejected[1]	---
**AM-S-W1705**	0.499	0.0223	0.0730	0.346 - 0.652	Rejected[1]	---
**AM-S-W1706**	0.296	0.0136	0.0459	0.200 - 0.392	Rejected[1]	---

Statistics
Serum PCB IUPAC # 170 (µg/L)

All methods	AM-S-W1704	AM-S-W1705	AM-S-W1706
N	13	13	13
Robust mean Algo A	0.181	0.499	0.296
Robust STDev	0.0178	0.0643	0.0392
Median	0.182	0.501	0.297
STDev from MAD	0.0163	0.0593	0.0371
Arithmetic mean	0.228	0.518	0.302
STDev	0.205	0.180	0.0880
CV or Variability	9.8%	12.9%	13.3%

GC-MS CI	AM-S-W1704	AM-S-W1705	AM-S-W1706
N	3	3	3
Robust mean Algo A	0.179	0.504	0.296
Robust STDev	0.00393	0.0146	0.00730
Median	0.180	0.507	0.297
STDev from MAD	0.00311	0.0116	0.00578
Arithmetic mean	0.176	0.492	0.290
STDev	0.00812	0.0325	0.0157
CV or Variability	2.2%	2.9%	2.5%

GC-MS EI	AM-S-W1704	AM-S-W1705	AM-S-W1706
N	3	3	3
Robust mean Algo A	0.171	0.462	0.269
Robust STDev	0.0125	0.0442	0.0284
Median	0.171	0.461	0.271
STDev from MAD	0.0163	0.0564	0.0326
Arithmetic mean	0.171	0.462	0.269
STDev	0.0110	0.0390	0.0251
CV or Variability	7.3%	9.6%	10.6%

GC-MS-MS	AM-S-W1704	AM-S-W1705	AM-S-W1706
N	4	4	4
Robust mean Algo A	0.185	0.506	0.305
Robust STDev	0.112	0.314	0.178
Median	0.180	0.491	0.303
STDev from MAD	0.0875	0.245	0.143
Arithmetic mean	0.333	0.541	0.305
STDev	0.382	0.339	0.157
CV or Variability	60.5%	62.1%	58.5%

When fewer than 20 results were considered for statistical treatment of all or a sub-sample of results, the accuracy of statistical data may be questionable.

Distribution

Serum PCB IUPAC # 170 (µg/L)

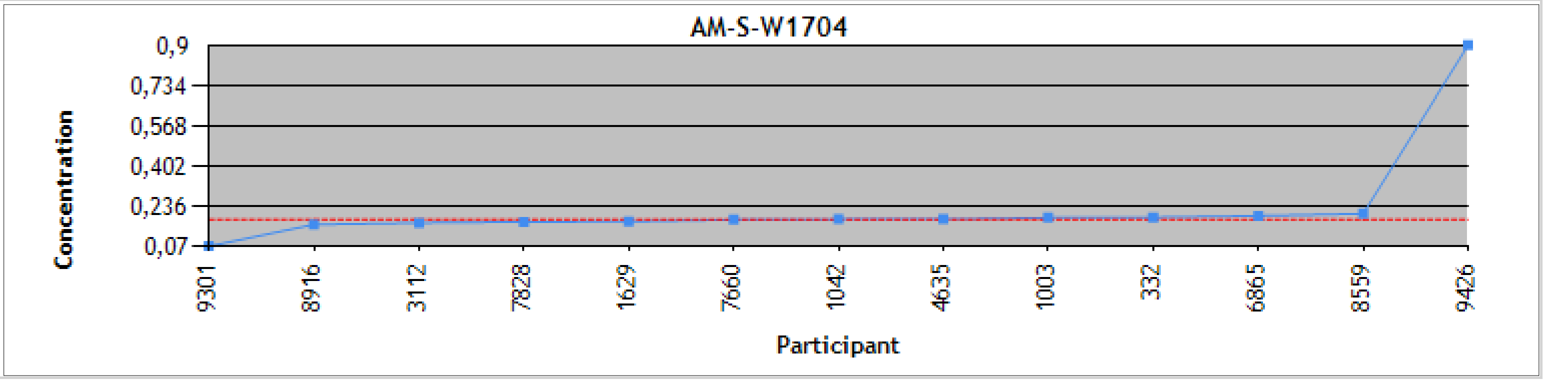

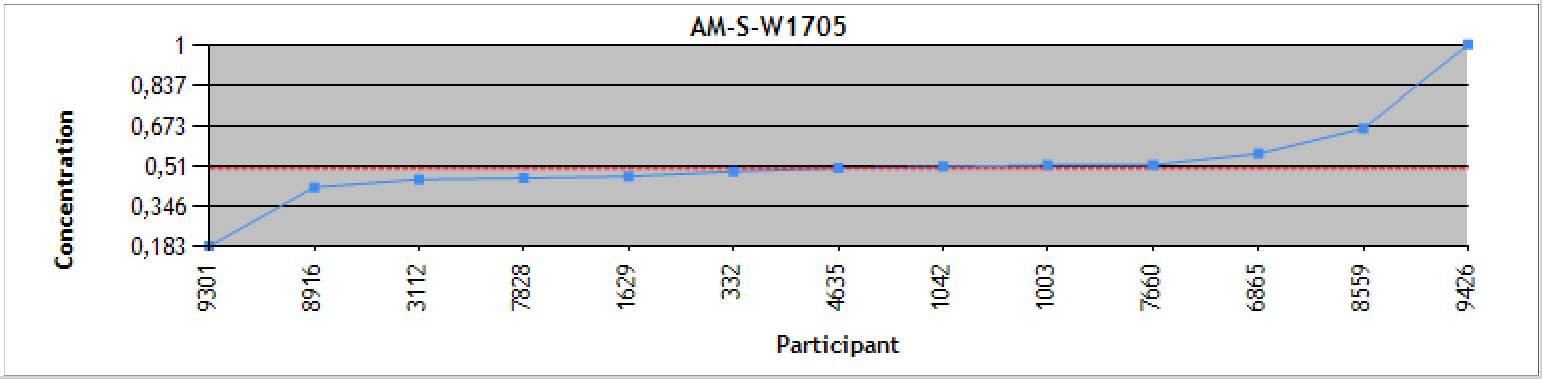

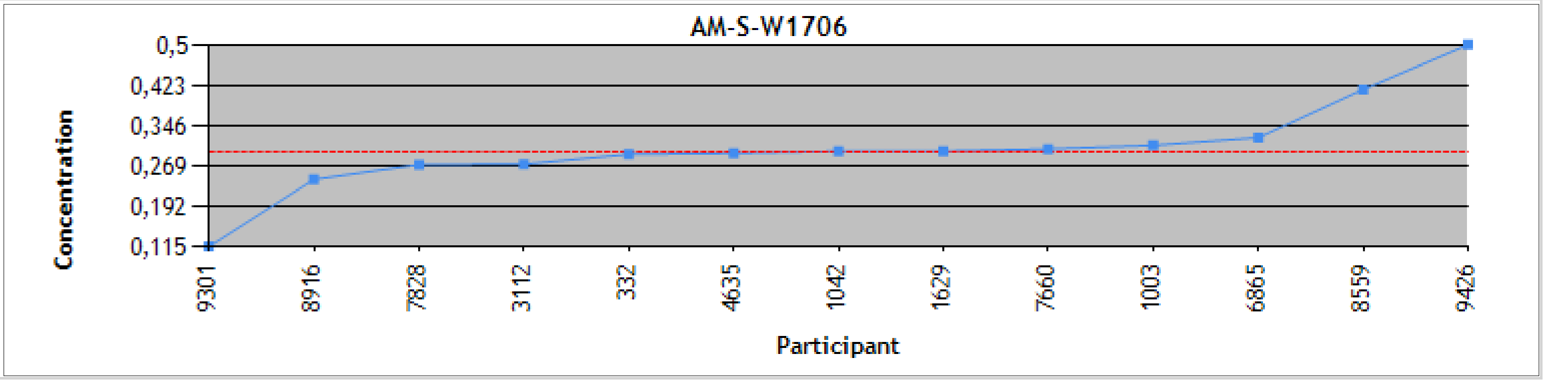

Individual results

Serum PCB IUPAC # 180 (µg/L)

Round #2017-02

Participant	AM-S-W1704	z'-score	AM-S-W1705	z'-score	AM-S-W1706	z'-score	Method
**270**	0.260	-0.08	0.604	0.21	0.757	0.84	GC-MS EI
**332**	0.275	0.31	0.602	0.18	0.693	0.20	ND
**748**	0.198	-1.67	0.354	**-2.67**	0.551	-1.22	ND
**1003**	0.249	-0.36	0.553	-0.38	0.653	-0.20	GC-MS-MS
**1042**	0.273	0.27	0.598	0.13	0.705	0.32	GC-MS CI
**1629**	0.253	-0.26	0.585	-0.01	0.697	0.24	GC-MS-MS
**2446**	0.241	-0.56	0.485	-1.16	0.594	-0.79	GC-MS EI
**3112**	0.248	-0.38	0.525	-0.70	0.633	-0.40	GC-MS CI
**4635**	0.246	-0.44	0.549	-0.43	0.627	-0.46	GC-MS EI
**6865**	0.281	0.46	0.633	0.54	0.698	0.25	GC
**7660**	0.275	0.29	0.612	0.29	0.717	0.44	GC-MS CI
**7828**	0.278	0.38	0.614	0.32	0.697	0.24	GC-MS EI
**8420**	0.300	0.95	0.620	0.39	0.760	0.87	ND
**8559**	0.266	0.08	0.670	0.97	0.729	0.56	ND
**8916**	0.265	0.05	0.565	-0.24	0.639	-0.34	GC-MS EI
**9301**	0.101	**-4.15**	0.231	**-4.09**	0.273	**-4.00**	GC-MS-MS
**9426**	1.20	**24.01**	1.30	**8.22**	<LD	---	GC-MS-MS

	Assigned value	Standard uncertainty	σ pt	Acceptable range	K-S (Lilliefors)	Species
**AM-S-W1704**	0.263	0.00720	0.0383	0.185 - 0.341	Rejected[1]	---
**AM-S-W1705**	0.586	0.0172	0.0851	0.412 - 0.760	Rejected[1]	---
**AM-S-W1706**	0.673	0.0204	0.0978	0.473 - 0.873	Accepted	---

Statistics
Serum PCB IUPAC # 180 (µg/L)

All methods	AM-S-W1704	AM-S-W1705	AM-S-W1706
N	17	17	16
Robust mean Algo A	0.263	0.586	0.673
Robust STDev	0.0237	0.0567	0.0652
Median	0.265	0.598	0.695
STDev from MAD	0.0237	0.0526	0.0727
Arithmetic mean	0.306	0.594	0.651
STDev	0.235	0.212	0.116
CV or Variability	9.0%	9.7%	9.7%

GC-MS CI	AM-S-W1704	AM-S-W1705	AM-S-W1706
N	3	3	3
Robust mean Algo A	0.273	0.592	0.700
Robust STDev	0.00206	0.0264	0.0223
Median	0.273	0.598	0.705
STDev from MAD	0.00163	0.0209	0.0176
Arithmetic mean	0.265	0.578	0.685
STDev	0.0150	0.0465	0.0455
CV or Variability	0.8%	4.5%	3.2%

GC-MS EI	AM-S-W1704	AM-S-W1705	AM-S-W1706
N	5	5	5
Robust mean Algo A	0.258	0.563	0.659
Robust STDev	0.0169	0.0583	0.0659
Median	0.260	0.565	0.639
STDev from MAD	0.0208	0.0578	0.0667
Arithmetic mean	0.258	0.563	0.663
STDev	0.0149	0.0514	0.0645
CV or Variability	6.5%	10.3%	10.0%

GC-MS-MS	AM-S-W1704	AM-S-W1705	AM-S-W1706
N	4	4	3
Robust mean Algo A	0.256	0.583	0.635
Robust STDev	0.148	0.340	0.0824
Median	0.251	0.569	0.653
STDev from MAD	0.113	0.262	0.0653
Arithmetic mean	0.451	0.667	0.541
STDev	0.504	0.451	0.233
CV or Variability	57.8%	58.3%	13.0%

When fewer than 20 results were considered for statistical treatment of all or a sub-sample of results, the accuracy of statistical data may be questionable.

Distribution
Serum PCB IUPAC # 180 (µg/L)

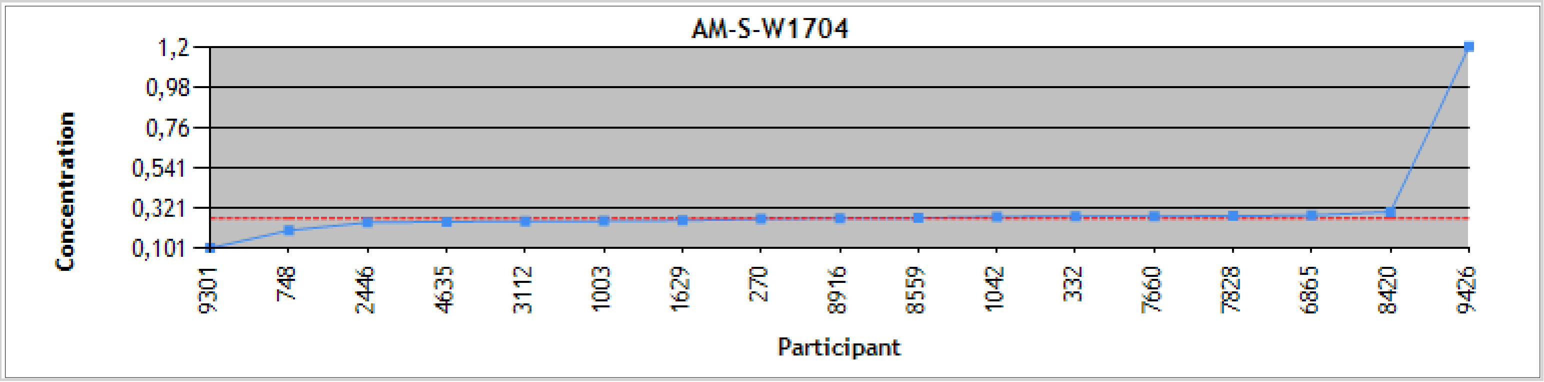

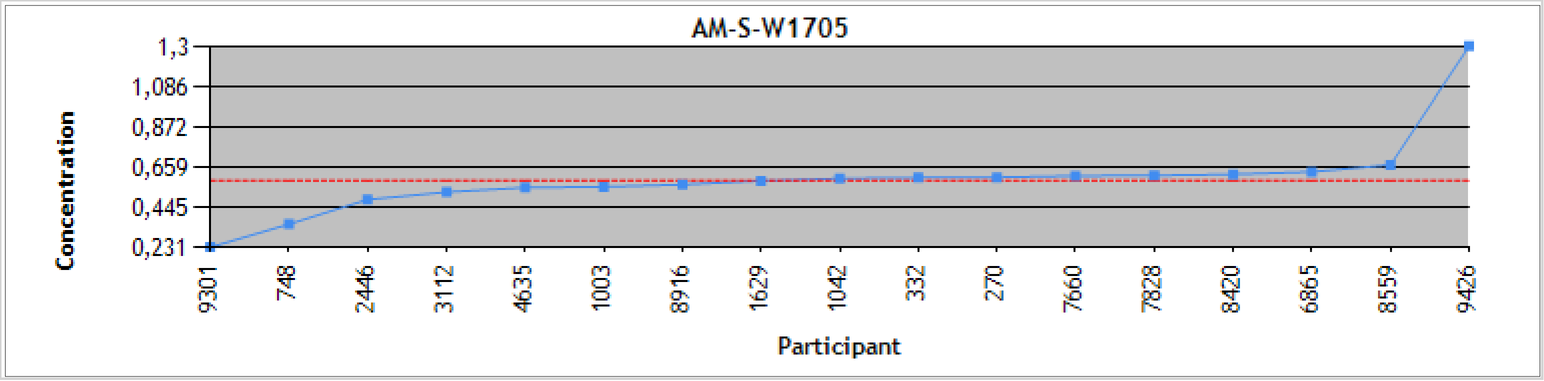

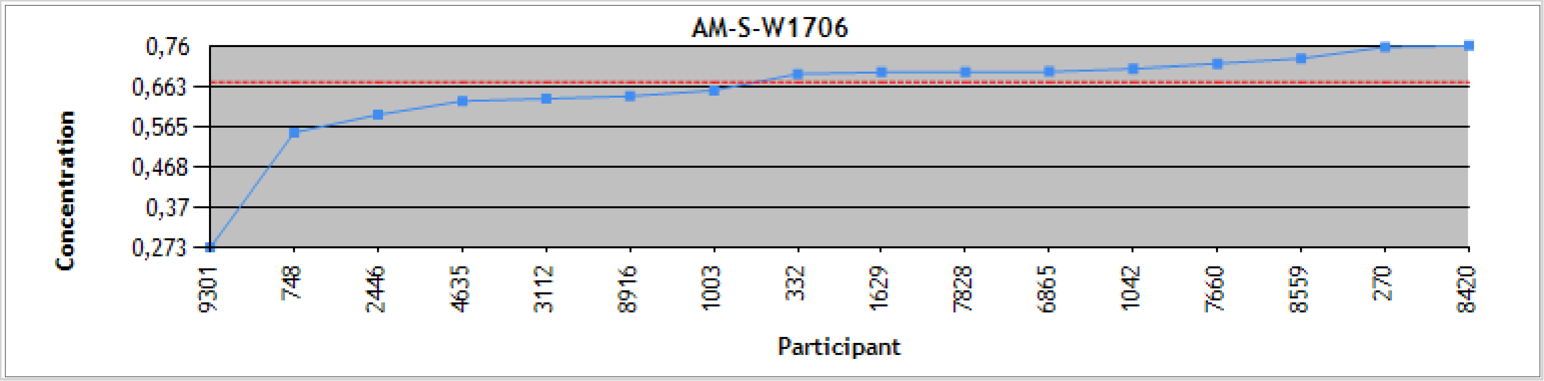

Individual results

Serum PCB IUPAC # 183 (µg/L)

Round #2017-02

Participant	AM-S-W1704	z'-score	AM-S-W1705	z'-score	AM-S-W1706	z'-score	Method
**270**	0.205	-0.81	0.516	-0.99	1.06	-0.22	GC-MS EI
**332**	0.257	0.22	0.646	-0.01	1.15	0.18	ND
**1003**	0.259	0.26	0.679	0.23	1.24	0.57	GC-MS-MS
**1042**	0.256	0.12	0.660	0.09	1.20	0.38	GC-MS CI
**1629**	0.236	-0.20	0.603	-0.34	1.15	0.18	GC-MS-MS
**3112**	0.104	**-2.85**	0.260	**-2.90**	0.480	**-2.78**	GC-MS CI
**4635**	0.286	0.79	0.697	0.37	1.14	0.14	GC-MS EI
**6865**	0.280	0.67	0.739	0.68	1.25	0.59	GC
**7660**	0.259	0.17	0.691	0.32	1.27	0.71	GC-MS CI
**7828**	0.264	0.36	0.681	0.25	1.25	0.62	GC-MS EI
**8559**	0.251	0.10	0.740	0.69	1.08	-0.13	ND
**8916**	0.227	-0.45	0.581	-0.50	1.01	-0.43	GC-MS EI
**9301**	0.0910	**-3.07**	0.226	**-3.15**	0.444	**-2.93**	GC-MS-MS
**9426**	1.70	**28.75**	1.40	**5.61**	0.700	-1.81	GC-MS-MS

	Assigned value	Standard uncertainty	σ pt	Acceptable range	K-S (Lilliefors)	Species
**AM-S-W1704**	0.250	0.0112	0.0500	0.148 - 0.352	Rejected[1]	---
**AM-S-W1705**	0.648	0.0339	0.130	0.380 - 0.916	Rejected[1]	---
**AM-S-W1706**	1.11	0.0471	0.223	0.655 - 1.57	Accepted	---

PCB IUPAC # 183 is not included in the scope of our accreditation.

Statistics

Serum PCB IUPAC # 183 (µg/L)

All methods	AM-S-W1704	AM-S-W1705	AM-S-W1706
N	14	14	14
Robust mean Algo A	0.250	0.648	1.11
Robust STDev	0.0334	0.101	0.141
Median	0.256	0.669	1.15
STDev from MAD	0.0326	0.101	0.143
Arithmetic mean	0.334	0.651	1.03
STDev	0.398	0.269	0.281
CV or Variability	13.4%	15.6%	12.7%

GC-MS CI	AM-S-W1704	AM-S-W1705	AM-S-W1706
N	6	3	3
Robust mean Algo A	0.255	0.647	1.17
Robust STDev	0.00435	0.0580	0.140
Median	0.256	0.660	1.20
STDev from MAD	0.00386	0.0460	0.111
Arithmetic mean	0.206	0.537	0.983
STDev	0.0791	0.240	0.437
CV or Variability	1.7%	9.0%	12.0%

GC-MS EI	AM-S-W1704	AM-S-W1705	AM-S-W1706
N	9	4	4
Robust mean Algo A	0.239	0.619	1.12
Robust STDev	0.0353	0.0971	0.116
Median	0.227	0.631	1.10
STDev from MAD	0.0326	0.0860	0.0964
Arithmetic mean	0.241	0.619	1.12
STDev	0.0343	0.0856	0.104
CV or Variability	14.8%	15.7%	10.4%

GC-MS-MS	AM-S-W1704	AM-S-W1705	AM-S-W1706
N	8	4	4
Robust mean Algo A	0.255	0.663	0.884
Robust STDev	0.148	0.428	0.427
Median	0.248	0.641	0.925
STDev from MAD	0.125	0.336	0.400
Arithmetic mean	0.572	0.727	0.884
STDev	0.700	0.490	0.376
CV or Variability	57.9%	64.5%	48.3%

When fewer than 20 results were considered for statistical treatment of all or a sub-sample of results, the accuracy of statistical data may be questionable.

Distribution

Serum PCB IUPAC # 183 (µg/L)

Distribution graph for PCB IUPAC # 183 in material AM-S-W1704 is not available.

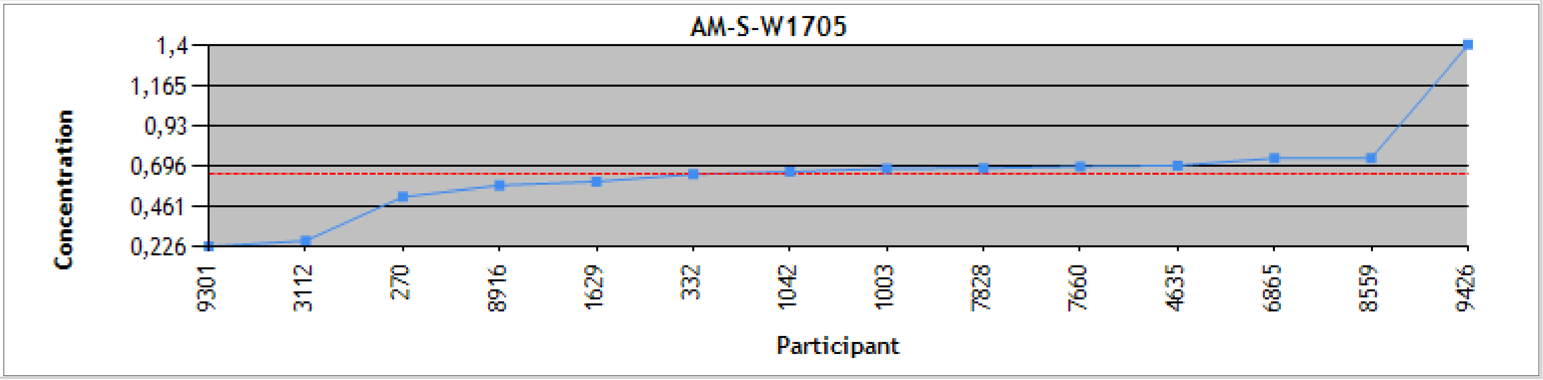

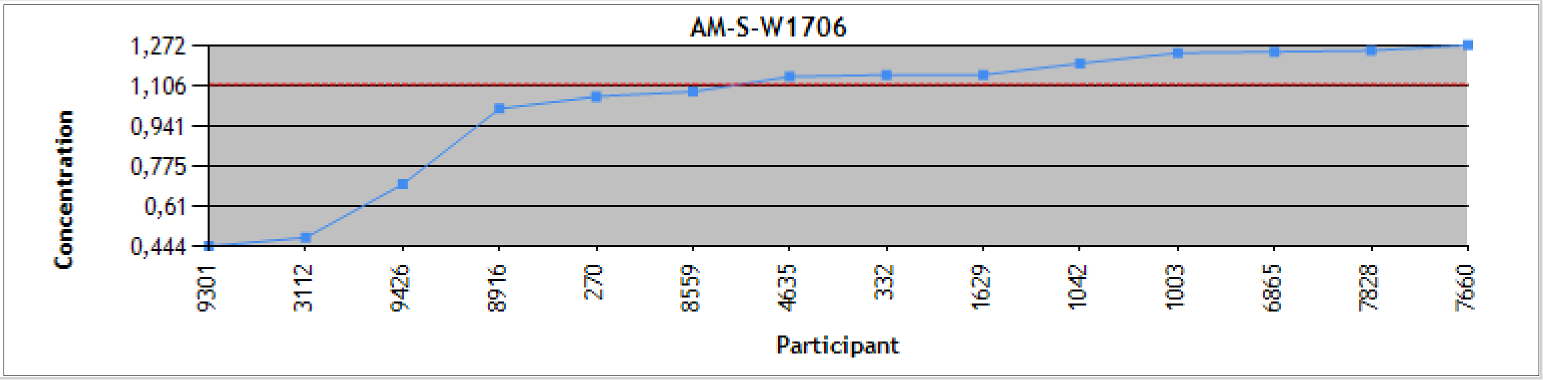

Individual results
Serum PCB IUPAC # 187 (µg/L)
Round #2017-02

Participant	AM-S-W1704	z'-score	AM-S-W1705	z'-score	AM-S-W1706	z'-score	Method
**270**	0.163	-0.66	0.768	-0.52	0.528	-0.39	GC-MS EI
**332**	0.180	-0.21	0.804	-0.32	0.543	-0.26	ND
**1003**	0.192	0.10	0.871	0.06	0.592	0.16	GC-MS-MS
**1042**	0.200	0.31	0.897	0.21	0.601	0.24	GC-MS CI
**1629**	0.189	0.03	0.827	-0.19	0.598	0.22	GC-MS-MS
**3112**	0.0590	**-3.38**	0.252	**-3.46**	0.172	**-3.47**	GC-MS CI
**4635**	0.230	1.10	0.925	0.37	0.570	-0.03	GC-MS EI
**6865**	0.215	0.71	1.07	1.18	0.610	0.32	GC
**7660**	0.189	0.03	0.863	0.02	0.593	0.17	GC-MS CI
**7828**	0.202	0.37	0.891	0.18	0.601	0.24	GC-MS EI
**8559**	0.187	-0.03	0.933	0.42	0.571	-0.02	ND
**8916**	0.186	-0.05	0.798	-0.35	0.517	-0.49	GC-MS EI
**9301**	0.0711	**-3.06**	0.304	**-3.17**	0.226	**-3.01**	GC-MS-MS
**9426**	***1.90***	**44.88**	1.90	**5.92**	1.50	**8.03**	GC-MS-MS

	Assigned value	Standard uncertainty	σ pt	Acceptable range	K-S (Lilliefors)	Species
**AM-S-W1704**	0.188	0.00643	0.0376	0.112 - 0.264	Accepted	---
**AM-S-W1705**	0.860	0.0350	0.172	0.509 - 1.21	Rejected[1]	---
**AM-S-W1706**	0.573	0.0137	0.115	0.342 - 0.804	Rejected[1]	---

PCB IUPAC # 187 is not included in the scope of our accreditation.

Statistics

Serum PCB IUPAC # 187 (µg/L)

All methods	AM-S-W1704	AM-S-W1705	AM-S-W1706
N	13	14	14
Robust mean Algo A	0.188	0.860	0.573
Robust STDev	0.0185	0.105	0.0411
Median	0.189	0.867	0.582
STDev from MAD	0.0159	0.0957	0.0356
Arithmetic mean	0.174	0.864	0.587
STDev	0.0511	0.375	0.297
CV or Variability	9.9%	12.2%	7.2%

GC-MS CI	AM-S-W1704	AM-S-W1705	AM-S-W1706
N	6	3	3
Robust mean Algo A	0.185	0.849	0.590
Robust STDev	0.0176	0.0636	0.0142
Median	0.189	0.863	0.593
STDev from MAD	0.0156	0.0504	0.0113
Arithmetic mean	0.149	0.671	0.455
STDev	0.0701	0.363	0.245
CV or Variability	9.5%	7.5%	2.4%

GC-MS EI	AM-S-W1704	AM-S-W1705	AM-S-W1706
N	8	4	4
Robust mean Algo A	0.195	0.846	0.554
Robust STDev	0.0296	0.0845	0.0440
Median	0.194	0.845	0.549
STDev from MAD	0.0289	0.0912	0.0393
Arithmetic mean	0.195	0.846	0.554
STDev	0.0261	0.0745	0.0388
CV or Variability	15.1%	10.0%	7.9%

GC-MS-MS	AM-S-W1704	AM-S-W1705	AM-S-W1706
N	6	4	4
Robust mean Algo A	0.188	0.870	0.606
Robust STDev	0.00502	0.545	0.363
Median	0.189	0.849	0.595
STDev from MAD	0.00445	0.420	0.276
Arithmetic mean	0.151	0.976	0.729
STDev	0.0617	0.668	0.543
CV or Variability	2.7%	62.7%	59.8%

When fewer than 20 results were considered for statistical treatment of all or a sub-sample of results, the accuracy of statistical data may be questionable.

Distribution
Serum PCB IUPAC # 187 (µg/L)

Distribution graph for PCB IUPAC # 187 in material AM-S-W1704 is not available.

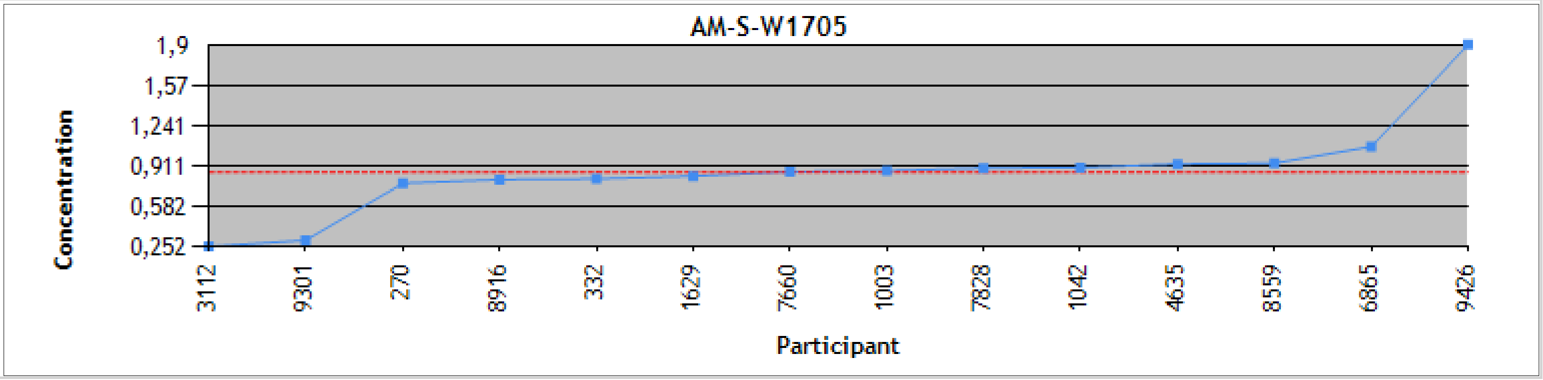

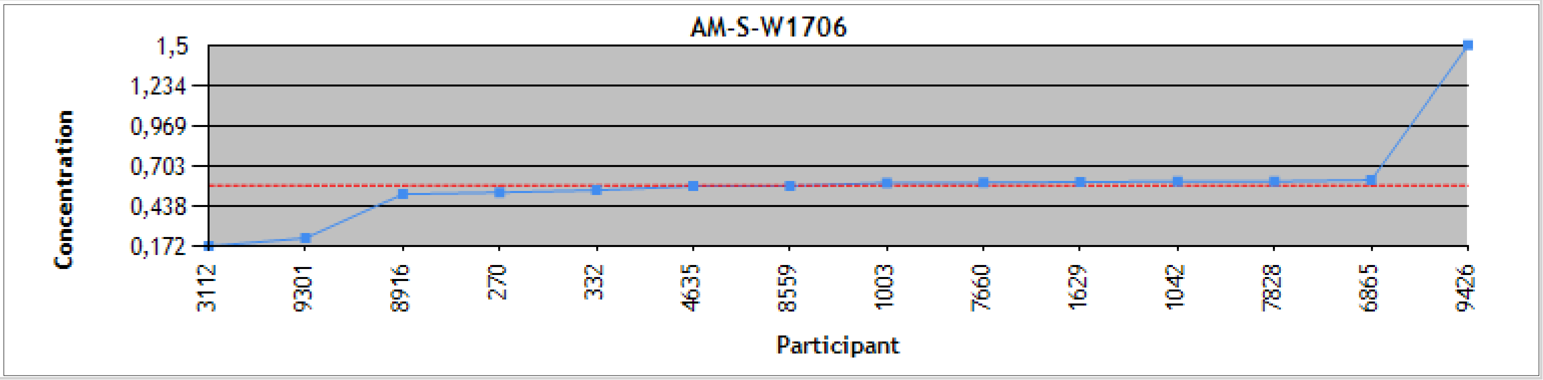

Individual results

Serum PFHxA (µg/L)

Round #2017-02

Participant	AM-S-Y1704	z'-score	AM-S-Y1705	z'-score	AM-S-Y1706	z'-score	Method
**270**	3.55	0.43	1.49	-0.49	4.69	0.25	LC-MS
**1003**	3.20	-0.23	1.41	-0.79	4.17	-0.48	LC-MS
**1042**	3.62	0.56	1.75	0.48	4.81	0.43	LC-MS
**1382**	2.98	-0.64	1.48	-0.53	4.07	-0.62	LC-MS
**1629**	2.86	-0.87	1.80	0.68	4.02	-0.69	LC-MS
**2446**	3.41	0.17	1.72	0.38	4.57	0.08	LC-MS
**2791**	3.76	0.83	1.76	0.53	5.15	0.90	LC-MS
**3367**	4.20	1.66	2.10	1.80	5.80	1.81	LC-MS
**5786**	3.84	0.98	1.91	1.09	5.15	0.90	LC-MS
**7828**	2.53	-1.49	1.24	-1.43	4.06	-0.63	LC-MS
**8916**	2.44	-1.65	1.12	-1.86	3.27	-1.74	LC-MS
**9301**	3.49	0.32	1.85	0.86	4.85	0.48	LC-MS
**9756**	3.12	-0.39	1.31	-1.16	4.10	-0.57	LC-MS

	Assigned value	Standard uncertainty	σ pt	Acceptable range	K-S (Lilliefors)	Species
**AM-S-Y1704**	3.32	0.183	0.499	2.26 - 4.38	Accepted	---
**AM-S-Y1705**	1.62	0.109	0.243	1.09 - 2.15	Accepted	---
**AM-S-Y1706**	4.51	0.222	0.677	3.08 - 5.94	Accepted	---

Statistics

Serum PFHxA (µg/L)

All methods	AM-S-Y1704	AM-S-Y1705	AM-S-Y1706
**N**	13	13	13
**Robust mean Algo A**	3.32	1.62	4.51
**Robust STDev**	0.529	0.315	0.641
**Median**	3.41	1.72	4.57
**STDev from MAD**	0.519	0.341	0.742
**Arithmetic mean**	3.31	1.61	4.52
**STDev**	0.516	0.290	0.657
**CV or Variability**	15.9%	19.5%	14.2%

LC-MS	AM-S-Y1704	AM-S-Y1705	AM-S-Y1706
**N**	13	13	13
**Robust mean Algo A**	3.33	1.62	4.51
**Robust STDev**	0.533	0.316	0.665
**Median**	3.41	1.72	4.57
**STDev from MAD**	0.519	0.341	0.742
**Arithmetic mean**	3.31	1.61	4.52
**STDev**	0.516	0.290	0.657
**CV or Variability**	16.0%	19.5%	14.7%

When fewer than 20 results were considered for statistical treatment of all or a sub-sample of results, the accuracy of statistical data may be questionable.

Distribution

Serum PFHxA (µg/L)

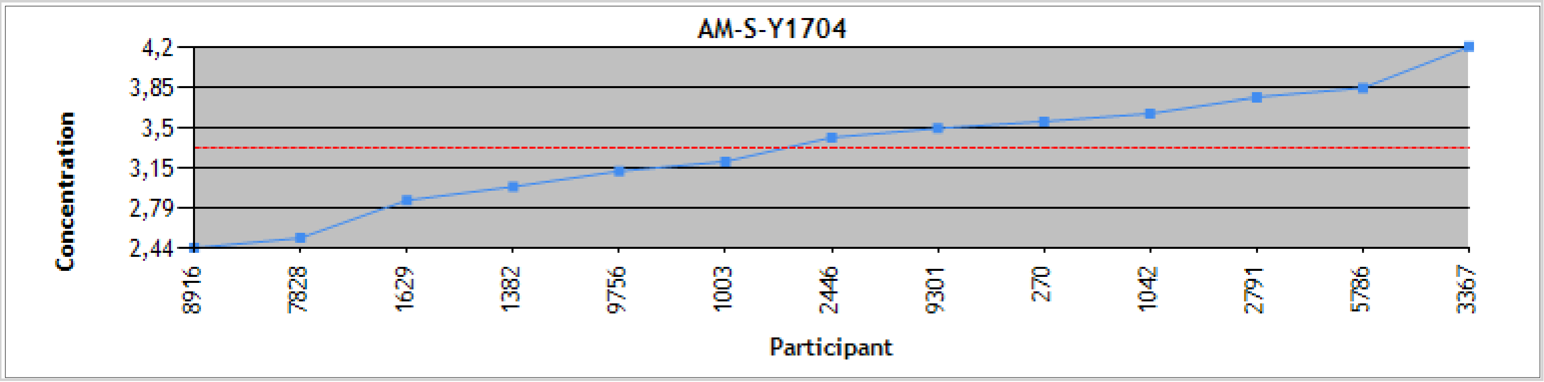

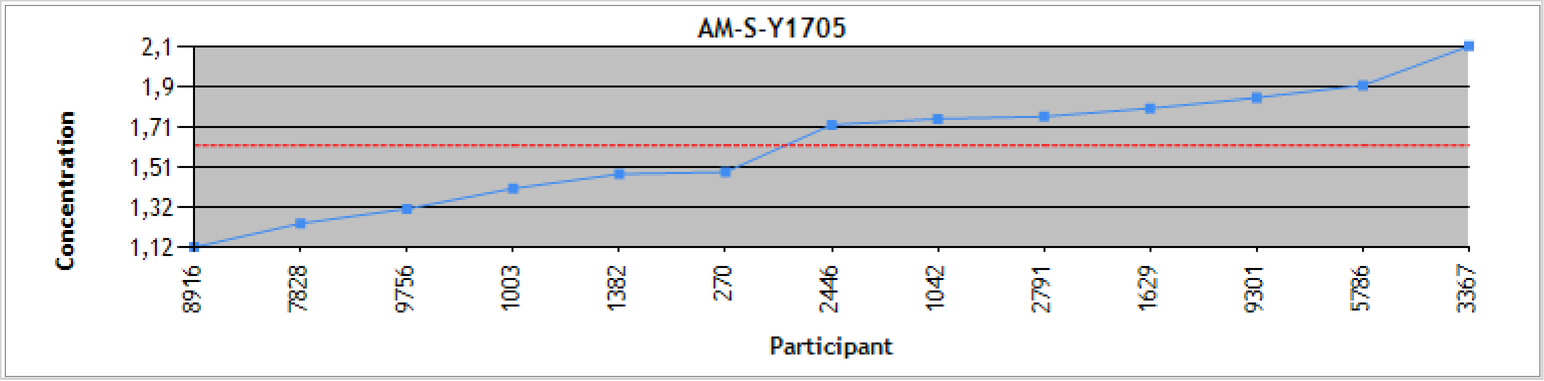

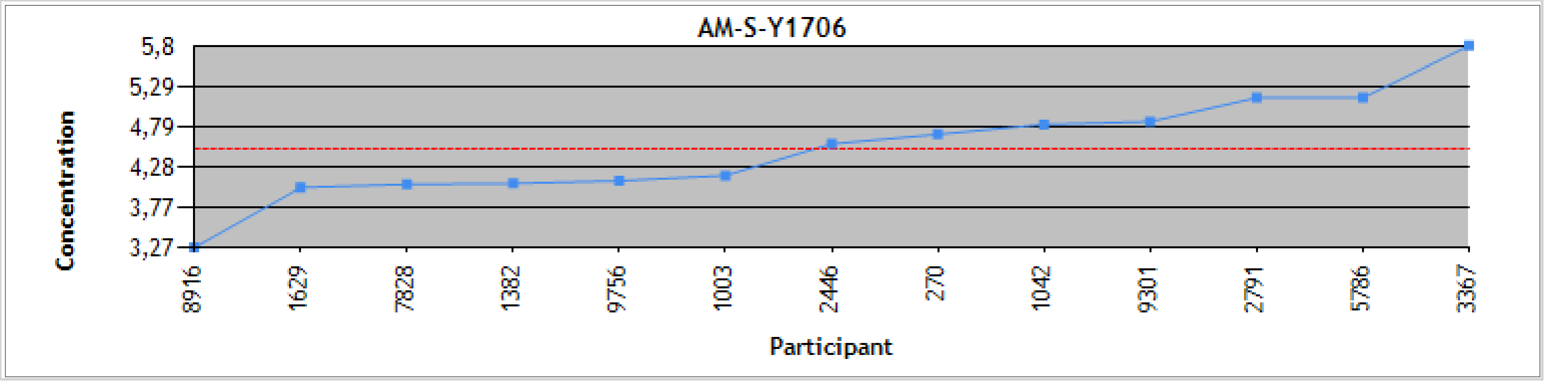

Individual results

Serum PFHxS (µg/L)

Round #2017-02

Participant	AM-S-Y1704	z'-score	AM-S-Y1705	z'-score	AM-S-Y1706	z'-score	Method
**270**	0.380	**-3.03**	10.8	-0.53	6.19	0.32	LC-MS
**1003**	0.520	-1.79	9.41	-1.31	4.60	-1.43	LC-MS
**1042**	0.724	0.03	13.2	0.79	6.18	0.30	LC-MS
**1382**	0.678	-0.38	10.8	-0.55	5.03	-0.95	LC-MS
**1629**	1.25	**4.70**	12.3	0.27	5.63	-0.30	LC-MS
**2446**	0.710	-0.10	10.7	-0.58	5.41	-0.54	LC-MS
**2791**	0.747	0.23	12.3	0.27	5.94	0.04	LC-MS
**3367**	0.800	0.70	14.4	1.43	6.80	0.99	LC-MS
**4087**	0.700	-0.19	11.1	-0.38	6.20	0.33	ND
**5786**	0.720	-0.01	11.6	-0.11	6.05	0.16	LC-MS
**7828**	0.862	1.25	11.0	-0.44	5.51	-0.43	LC-MS
**8916**	0.693	-0.25	11.9	0.05	5.44	-0.50	LC-MS
**9301**	0.821	0.89	12.1	0.16	6.30	0.44	LC-MS
**9756**	0.642	-0.70	13.7	1.05	6.88	1.08	LC-MS

	Assigned value	Standard uncertainty	σ pt	Acceptable range	K-S (Lilliefors)	Species
**AM-S-Y1704**	0.721	0.0308	0.108	0.496 - 0.946	Accepted	---
**AM-S-Y1705**	11.8	0.430	1.77	8.16 - 15.4	Accepted	---
**AM-S-Y1706**	5.90	0.219	0.885	4.08 - 7.72	Accepted	---

Statistics
Serum PFHxS (µg/L)

All methods	AM-S-Y1704	AM-S-Y1705	AM-S-Y1706
**N**	14	14	14
**Robust mean Algo A**	0.721	11.8	5.90
**Robust STDev**	0.0923	1.29	0.654
**Median**	0.715	11.7	6.00
**STDev from MAD**	0.0814	1.22	0.630
**Arithmetic mean**	0.732	11.8	5.87
**STDev**	0.192	1.33	0.640
**CV or Variability**	12.8%	10.9%	11.1%

LC-MS	AM-S-Y1704	AM-S-Y1705	AM-S-Y1706
**N**	13	13	13
**Robust mean Algo A**	0.722	11.9	5.87
**Robust STDev**	0.121	1.47	0.685
**Median**	0.720	11.9	5.94
**STDev from MAD**	0.115	1.55	0.638
**Arithmetic mean**	0.734	11.9	5.84
**STDev**	0.200	1.37	0.658
**CV or Variability**	16.7%	12.4%	11.7%

When fewer than 20 results were considered for statistical treatment of all or a sub-sample of results, the accuracy of statistical data may be questionable.

Distribution
Serum PFHxS (µg/L)

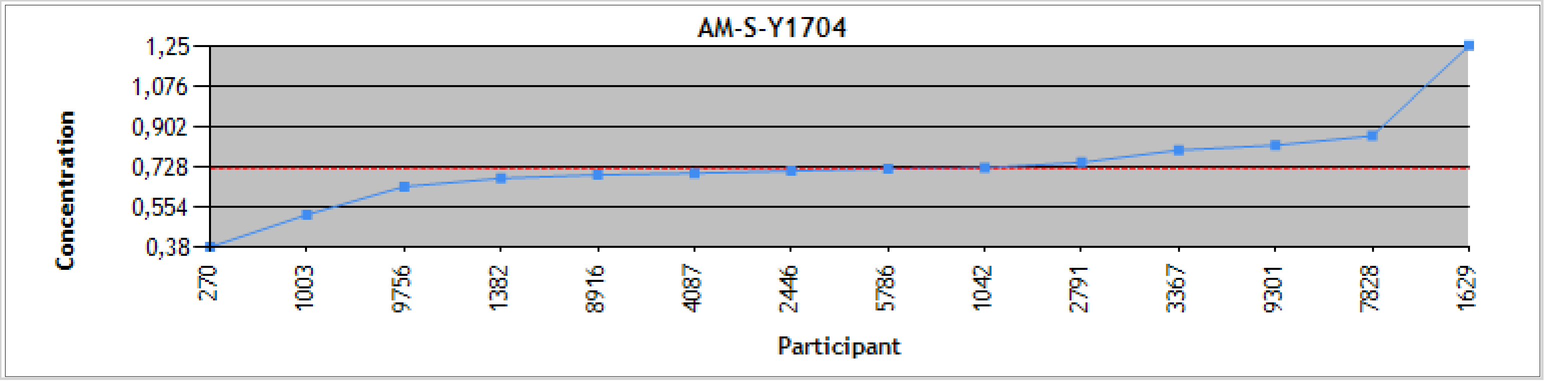

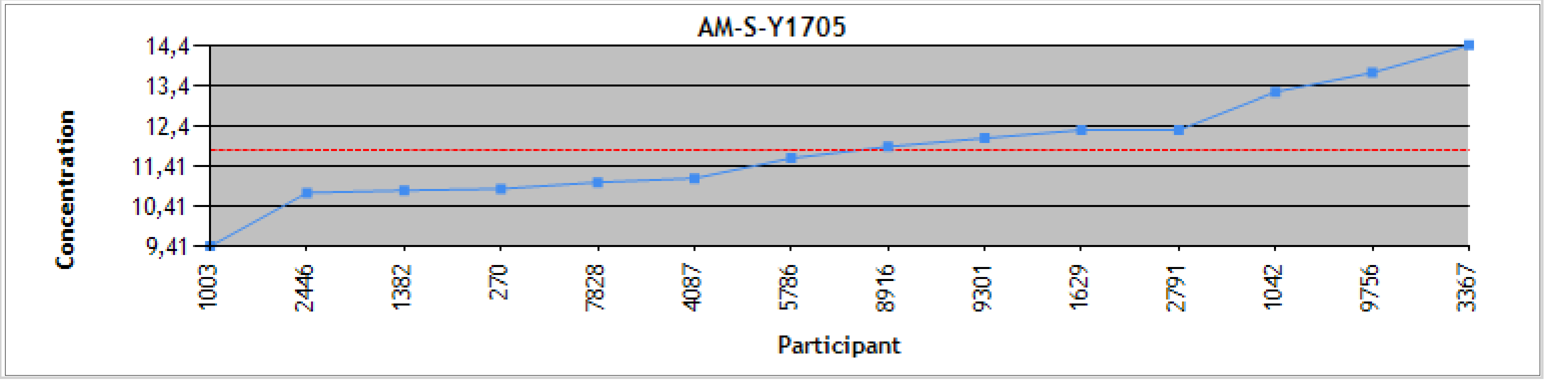

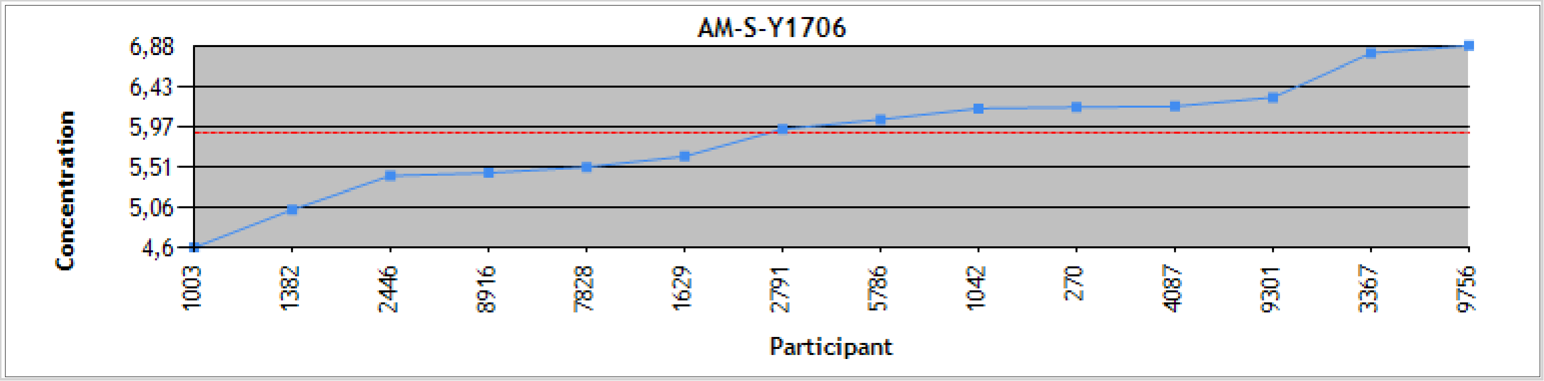

Individual results

Serum PFNA (µg/L)

Round #2017-02

Participant	AM-S-Y1704	z'-score	AM-S-Y1705	z'-score	AM-S-Y1706	z'-score	Method
**270**	0.680	-0.04	2.01	-1.44	1.40	0.19	LC-MS
**1003**	0.620	-0.59	2.45	-0.35	1.17	-0.92	LC-MS
**1042**	0.680	-0.04	2.70	0.28	1.41	0.24	LC-MS
**1382**	0.600	-0.78	2.30	-0.72	1.21	-0.73	LC-MS
**1629**	0.872	1.74	2.19	-1.00	0.953	-1.97	LC-MS
**2446**	0.670	-0.13	2.63	0.10	1.33	-0.15	LC-MS
**2791**	0.763	0.73	3.17	1.44	1.63	1.31	LC-MS
**3367**	0.900	2.00	3.10	1.27	1.70	1.65	LC-MS
**4087**	0.800	1.07	2.80	0.52	1.40	0.19	ND
**5786**	0.630	-0.50	2.75	0.40	1.33	-0.15	LC-MS
**7828**	0.559	-1.16	2.61	0.05	1.40	0.19	LC-MS
**8916**	0.559	-1.16	2.13	-1.14	1.09	-1.29	LC-MS
**9301**	0.732	0.44	2.57	-0.05	1.35	-0.05	LC-MS
**9756**	0.644	-0.37	2.79	0.50	1.43	0.35	LC-MS

	Assigned value	Standard uncertainty	σ pt	Acceptable range	K-S (Lilliefors)	Species
**AM-S-Y1704**	0.684	0.0344	0.103	0.467 - 0.901	Accepted	---
**AM-S-Y1705**	2.59	0.103	0.389	1.78 - 3.40	Accepted	---
**AM-S-Y1706**	1.36	0.0309	0.204	0.947 - 1.77	Accepted	---

Statistics
Serum PFNA (µg/L)

All methods	AM-S-Y1704	AM-S-Y1705	AM-S-Y1706
N	14	14	14
Robust mean Algo A	0.684	2.59	1.36
Robust STDev	0.103	0.309	0.0924
Median	0.675	2.62	1.38
STDev from MAD	0.0979	0.260	0.0758
Arithmetic mean	0.693	2.59	1.34
STDev	0.107	0.343	0.195
CV or Variability	15.0%	11.9%	6.8%

LC-MS	AM-S-Y1704	AM-S-Y1705	AM-S-Y1706
N	13	13	13
Robust mean Algo A	0.673	2.57	1.34
Robust STDev	0.0954	0.321	0.147
Median	0.670	2.61	1.35
STDev from MAD	0.0919	0.266	0.122
Arithmetic mean	0.685	2.57	1.34
STDev	0.107	0.352	0.202
CV or Variability	14.2%	12.5%	10.9%

When fewer than 20 results were considered for statistical treatment of all or a sub-sample of results, the accuracy of statistical data may be questionable.

Distribution

Serum PFNA (µg/L)

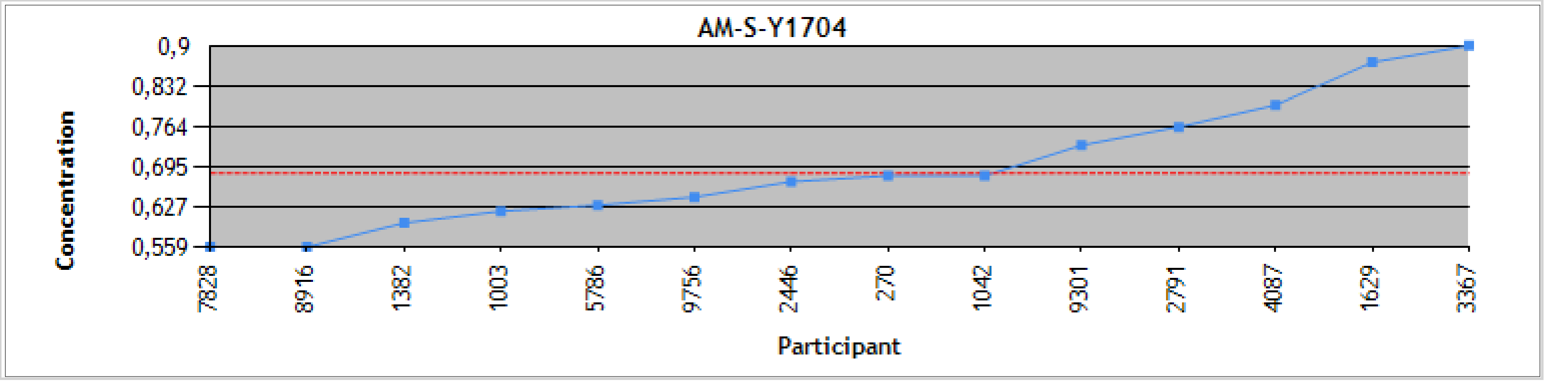

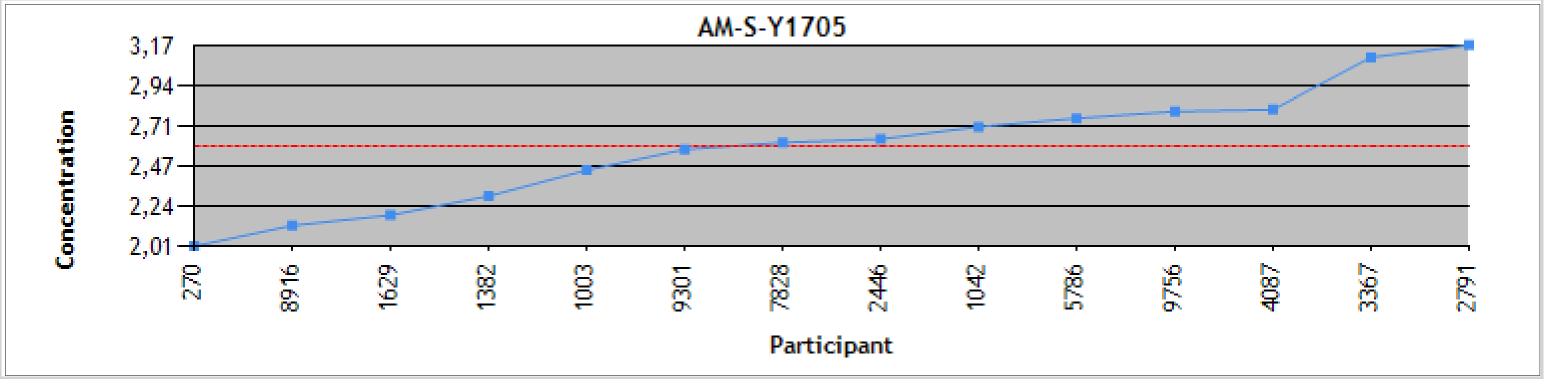

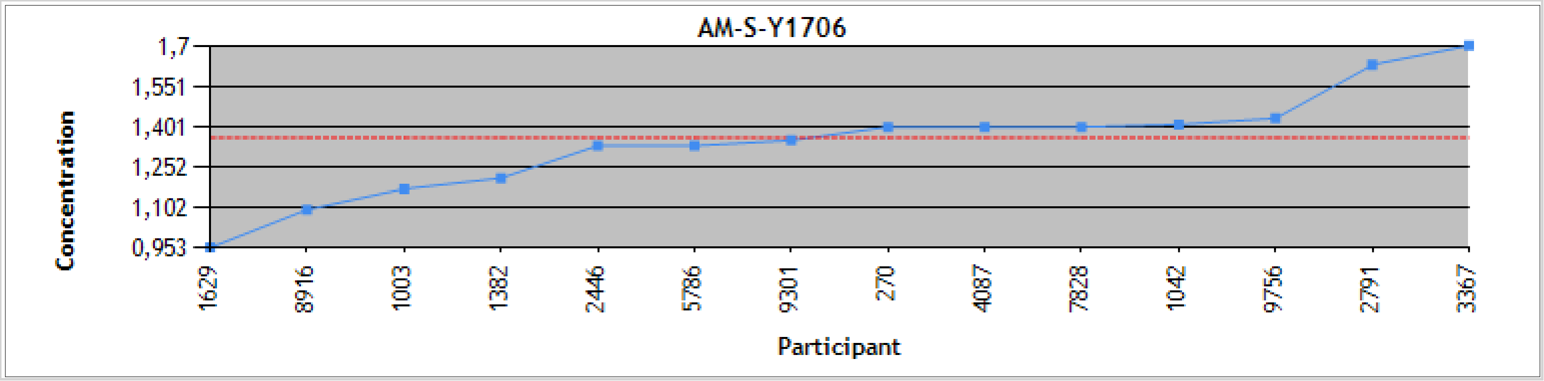

Individual results

Serum PFOA (µg/L)

Round #2017-02

Participant	AM-S-Y1704	z'-score	AM-S-Y1705	z'-score	AM-S-Y1706	z'-score	Method
**1003**	12.9	-0.84	2.65	-1.30	5.43	-1.34	LC-MS
**1042**	14.6	0.15	3.28	0.08	6.63	0.13	LC-MS
**1382**	11.9	-1.44	2.84	-0.88	5.55	-1.19	LC-MS
**1629**	16.1	1.08	2.46	-1.71	7.21	0.83	LC-MS
**2446**	13.7	-0.34	3.23	-0.02	6.37	-0.19	LC-MS
**2791**	16.7	1.44	4.00	1.67	7.79	1.53	LC-MS
**3367**	15.8	0.90	3.70	1.01	7.10	0.69	LC-MS
**4087**	14.7	0.24	3.40	0.35	6.70	0.21	ND
**5786**	14.9	0.36	3.12	-0.26	5.50	-1.25	LC-MS
**7828**	13.3	-0.60	3.18	-0.13	6.56	0.04	LC-MS
**7944**	13.5	-0.46	3.59	0.77	6.31	-0.27	LC-MS
**8916**	14.6	0.16	3.33	0.19	6.74	0.25	LC-MS
**9301**	14.2	-0.06	3.34	0.22	7.20	0.81	LC-MS
**9756**	13.9	-0.26	3.01	-0.50	6.22	-0.37	LC-MS

	Assigned value	Standard uncertainty	σ pt	Acceptable range	K-S (Lilliefors)	Species
**AM-S-Y1704**	14.3	0.410	1.62	11.0 - 17.6	Accepted	---
**AM-S-Y1705**	3.24	0.112	0.441	2.33 - 4.15	Accepted	---
**AM-S-Y1706**	6.53	0.236	0.789	4.88 - 8.18	Accepted	---

Statistics

Serum PFOA (µg/L)

All methods	AM-S-Y1704	AM-S-Y1705	AM-S-Y1706
N	14	14	14
Robust mean Algo A	14.3	3.24	6.53
Robust STDev	1.23	0.336	0.707
Median	14.4	3.25	6.60
STDev from MAD	1.09	0.287	0.651
Arithmetic mean	14.3	3.22	6.52
STDev	1.29	0.406	0.696
CV or Variability	8.6%	10.4%	10.8%

LC-MS	AM-S-Y1704	AM-S-Y1705	AM-S-Y1706
N	13	13	13
Robust mean Algo A	14.3	3.22	6.50
Robust STDev	1.21	0.371	0.810
Median	14.2	3.23	6.56
STDev from MAD	1.04	0.322	0.801
Arithmetic mean	14.3	3.21	6.51
STDev	1.34	0.419	0.723
CV or Variability	8.5%	11.5%	12.5%

When fewer than 20 results were considered for statistical treatment of all or a sub-sample of results, the accuracy of statistical data may be questionable.

Distribution
Serum PFOA (µg/L)

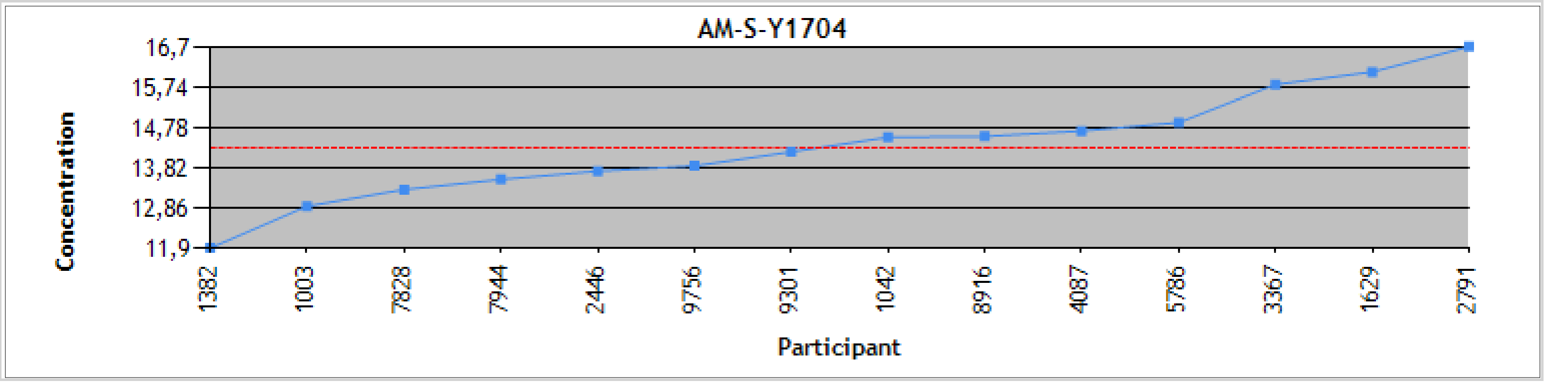

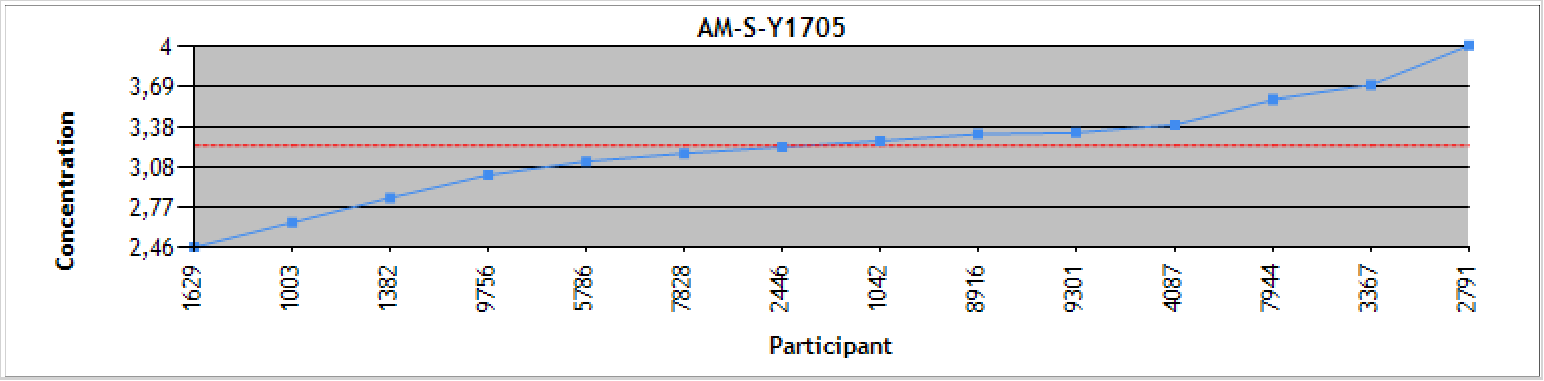

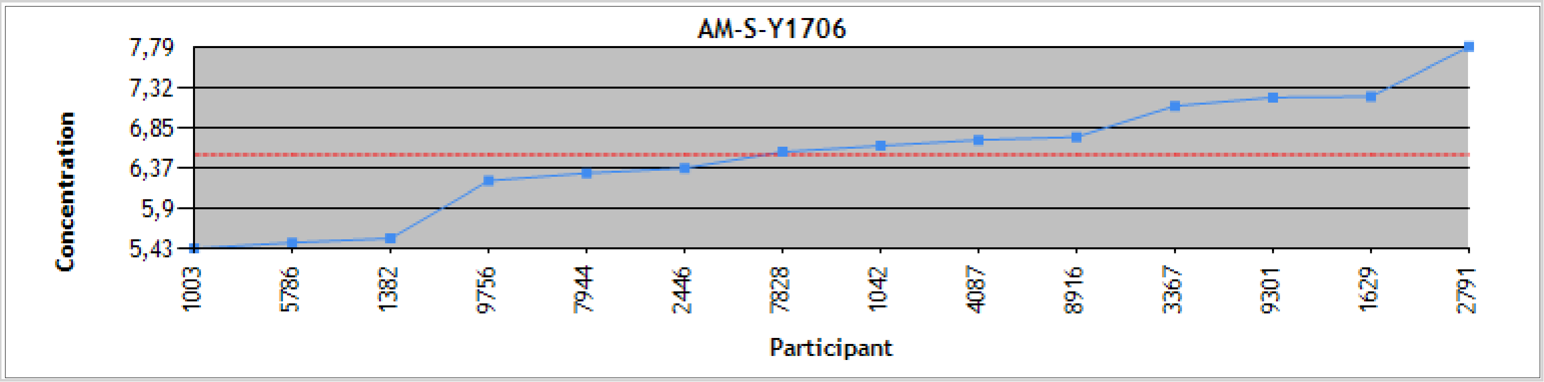

Individual results
Serum PFOS (µg/L)
Round #2017-02

Participant	AM-S-Y1704	z'-score	AM-S-Y1705	z'-score	AM-S-Y1706	z'-score	Method
**270**	97.1	0.07	31.1	-0.60	170	-0.27	LC-MS
**1003**	104	0.55	35.7	0.26	195	0.69	LC-MS
**1042**	91.5	-0.32	33.1	-0.23	162	-0.57	LC-MS
**1382**	87.7	-0.59	32.3	-0.37	163	-0.54	LC-MS
**1629**	101	0.34	39.0	0.86	192	0.58	LC-MS
**2446**	103	0.51	37.6	0.61	194	0.67	LC-MS
**2791**	95.9	-0.01	34.8	0.09	173	-0.15	LC-MS
**3367**	86.4	-0.68	33.3	-0.18	162	-0.56	LC-MS
**4087**	92.1	-0.28	33.7	-0.11	165	-0.45	ND
**5786**	97.5	0.10	34.4	0.02	176	-0.04	LC-MS
**7828**	110	0.97	39.6	0.97	202	0.96	LC-MS
**7944**	76.8	-1.35	29.5	-0.88	144	-1.25	LC-MS
**8916**	90.5	-0.39	32.2	-0.39	169	-0.32	LC-MS
**9301**	109	0.90	43.5	1.68	194	0.65	LC-MS
**9756**	94.7	-0.10	33.8	-0.08	197	0.78	LC-MS

	Assigned value	Standard uncertainty	σ pt	Acceptable range	K-S (Lilliefors)	Species
**AM-S-Y1704**	96.1	2.77	14.1	67.4 - 125	Accepted	---
**AM-S-Y1705**	34.3	0.887	5.39	23.4 - 45.2	Accepted	---
**AM-S-Y1706**	177	5.85	25.4	125 - 229	Accepted	---

Statistics

Serum PFOS (µg/L)

All methods	AM-S-Y1704	AM-S-Y1705	AM-S-Y1706
N	15	15	15
Robust mean Algo A	96.1	34.3	177
Robust STDev	8.59	2.75	18.1
Median	95.9	33.8	173
STDev from MAD	7.99	2.44	16.0
Arithmetic mean	95.8	34.9	177
STDev	8.90	3.66	17.2
CV or Variability	8.9%	8.0%	10.2%

LC-MS	AM-S-Y1704	AM-S-Y1705	AM-S-Y1706
N	14	14	14
Robust mean Algo A	96.5	34.5	178
Robust STDev	9.50	3.16	19.2
Median	96.5	34.1	175
STDev from MAD	9.60	2.78	22.1
Arithmetic mean	96.1	35.0	178
STDev	9.18	3.79	17.5
CV or Variability	9.8%	9.2%	10.8%

When fewer than 20 results were considered for statistical treatment of all or a sub-sample of results, the accuracy of statistical data may be questionable.

Distribution

Serum PFOS (µg/L)

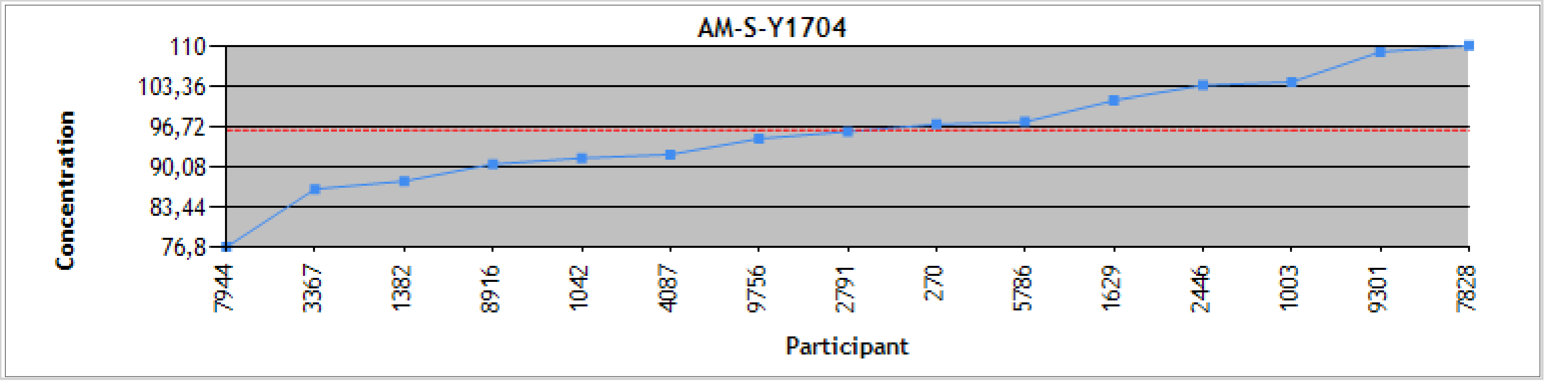

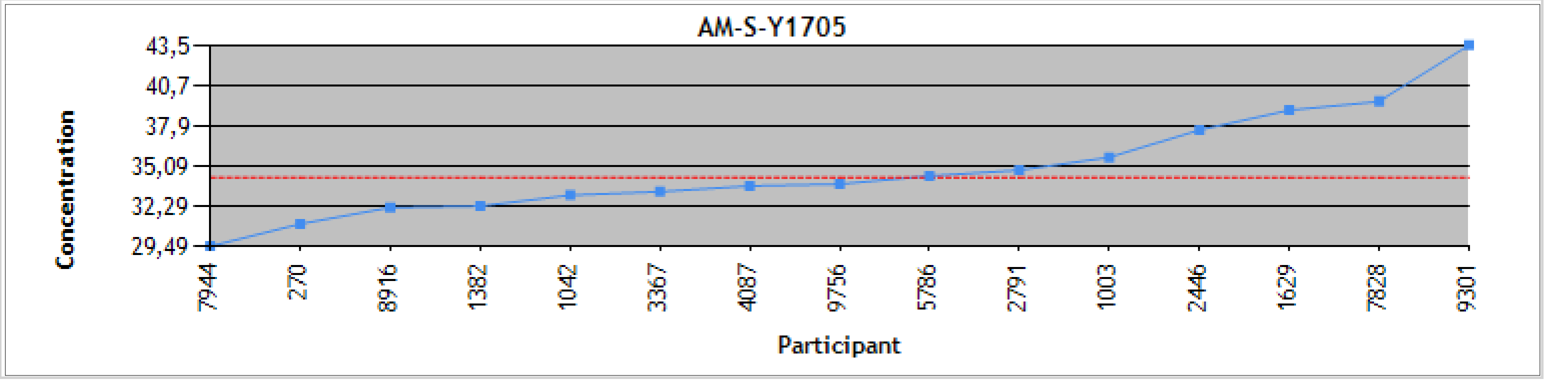

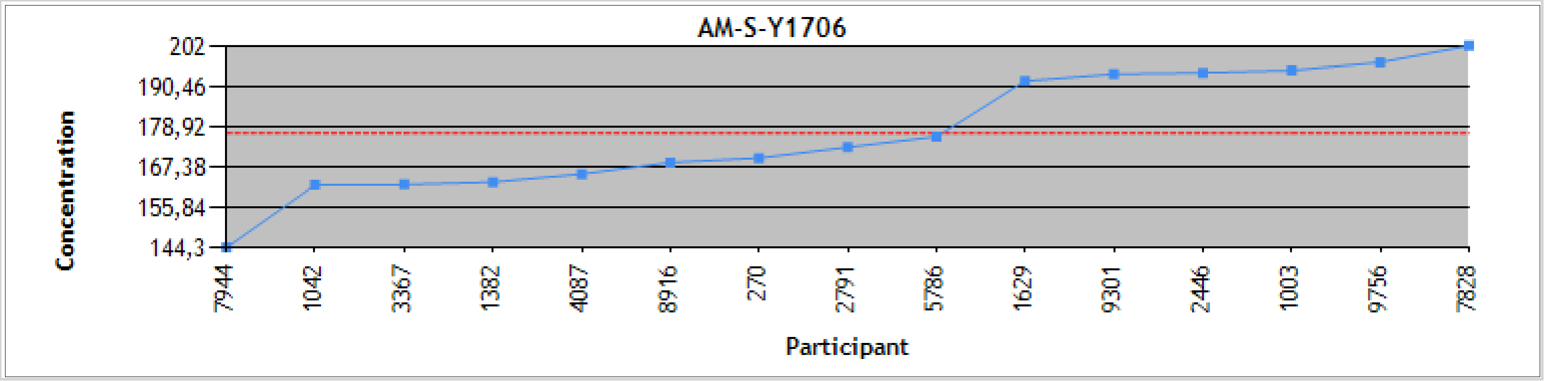

Individual results
Serum PFUdA (µg/L)
Round #2017-02

Participant	AM-S-Y1704	z'-score	AM-S-Y1705	z'-score	AM-S-Y1706	z'-score	Method
**270**	0.600	-0.43	1.99	-0.89	1.56	0.26	LC-MS
**1003**	0.540	-1.02	2.18	-0.36	1.45	-0.22	LC-MS
**1042**	0.693	0.48	2.46	0.40	1.58	0.34	LC-MS
**1382**	0.549	-0.93	2.01	-0.84	1.26	-1.05	LC-MS
**1629**	0.961	**3.11**	2.21	-0.28	1.17	-1.44	LC-MS
**2446**	0.640	-0.04	2.35	0.11	1.53	0.13	LC-MS
**3367**	1.00	**3.49**	3.40	**3.04**	2.20	**3.05**	LC-MS
**4087**	0.600	-0.43	2.50	0.53	1.50	0.00	ND
**5786**	0.640	-0.04	2.30	-0.03	1.52	0.09	LC-MS
**7828**	0.687	0.42	2.44	0.36	1.57	0.31	LC-MS
**8916**	0.547	-0.95	2.10	-0.58	1.36	-0.60	LC-MS
**9301**	0.841	1.93	2.69	1.06	2.02	**2.27**	LC-MS
**9756**	0.584	-0.59	2.12	-0.54	1.37	-0.59	LC-MS

	Assigned value	Standard uncertainty	σ pt	Acceptable range	K-S (Lilliefors)	Species
**AM-S-Y1704**	0.644	0.0329	0.0966	0.440 - 0.848	Rejected[1]	---
**AM-S-Y1705**	2.31	0.0933	0.347	1.59 - 3.03	Accepted	---
**AM-S-Y1706**	1.50	0.0445	0.225	1.04 - 1.96	Rejected[1]	---

Statistics
Serum PFUdA (µg/L)

All methods	AM-S-Y1704	AM-S-Y1705	AM-S-Y1706
N	13	13	13
Robust mean Algo A	0.644	2.31	1.50
Robust STDev	0.0950	0.269	0.128
Median	0.640	2.30	1.52
STDev from MAD	0.0828	0.270	0.104
Arithmetic mean	0.683	2.37	1.55
STDev	0.155	0.373	0.282
CV or Variability	14.8%	11.6%	8.5%

LC-MS	AM-S-Y1704	AM-S-Y1705	AM-S-Y1706
N	12	12	12
Robust mean Algo A	0.658	2.28	1.50
Robust STDev	0.115	0.250	0.197
Median	0.640	2.26	1.53
STDev from MAD	0.109	0.250	0.174
Arithmetic mean	0.690	2.35	1.55
STDev	0.159	0.387	0.294
CV or Variability	17.5%	11.0%	13.1%

When fewer than 20 results were considered for statistical treatment of all or a sub-sample of results, the accuracy of statistical data may be questionable.

Distribution
Serum PFUdA (µg/L)

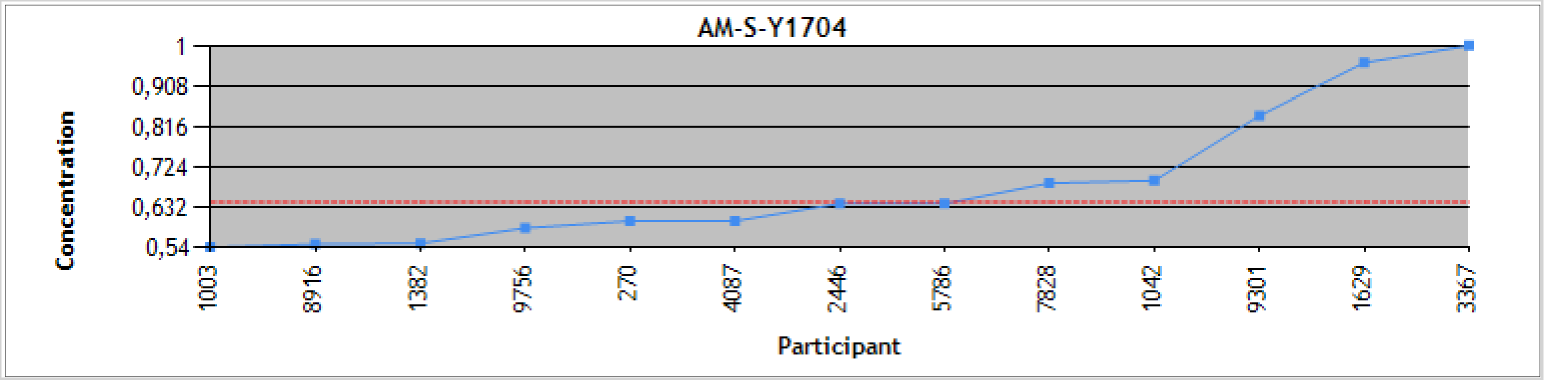

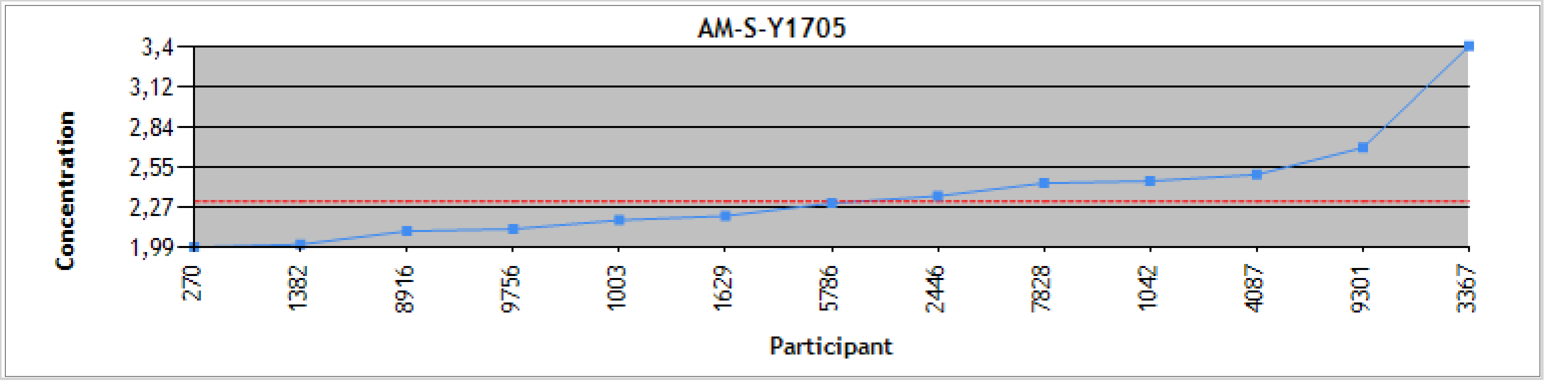

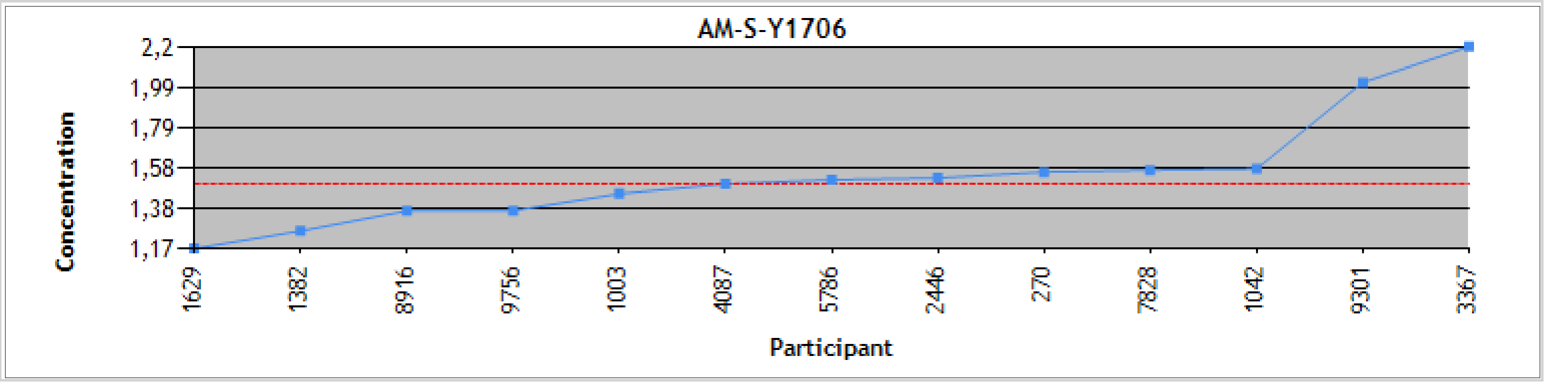

Individual results

Serum Total Cholesterol (g/L)

Round #2017-02

Participant	AM-S-W1704	z'-score	AM-S-W1705	z'-score	AM-S-W1706	z'-score	Method
**1042**	2.52	-0.12	1.81	-0.10	1.93	0.17	ND
**2446**	2.56	0.41	1.88	1.04	1.93	0.17	ND
**4635**	2.48	-0.66	1.79	-0.45	1.91	-0.15	ND
**7336**	2.51	-0.28	1.82	0.00	1.92	0.00	ND
**7660**	2.56	0.36	1.81	-0.10	1.93	0.10	ND

	Assigned value	Standard uncertainty	σ pt	Acceptable range	K-S (Lilliefors)	Species
**AM-S-W1704**	2.53	0.0207	0.0693	2.39 - 2.67	Accepted	---
**AM-S-W1705**	1.82	0.00622	0.0574	1.70 - 1.94	Rejected[1]	---
**AM-S-W1706**	1.92	0.00372	0.0592	1.80 - 2.04	Accepted	---

Total cholesterol is not included in the scope of our accreditation.

Statistics

Serum Total Cholesterol (g/L)

All methods	AM-S-W1704	AM-S-W1705	AM-S-W1706
N	5	5	5
Robust mean Algo A	2.53	1.82	1.92
Robust STDev	0.0371	0.0111	0.00665
Median	2.52	1.81	1.93
STDev from MAD	0.0519	0.00890	0.00593
Arithmetic mean	2.53	1.82	1.92
STDev	0.0327	0.0326	0.00805
CV or Variability	1.5%	0.6%	0.3%

When fewer than 20 results were considered for statistical treatment of all or a sub-sample of results, the accuracy of statistical data may be questionable.

Distribution

Serum Total Cholesterol (g/L)

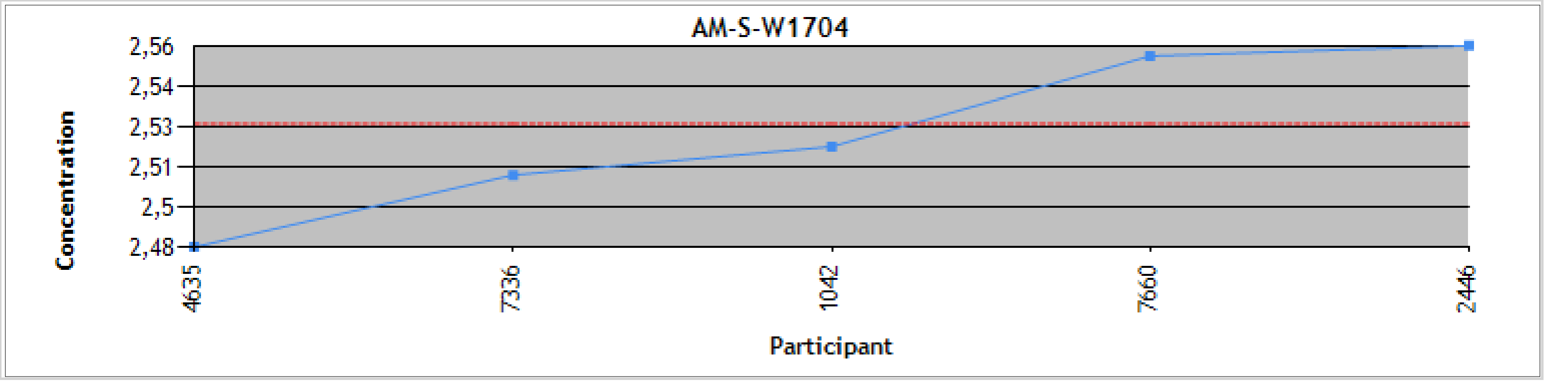

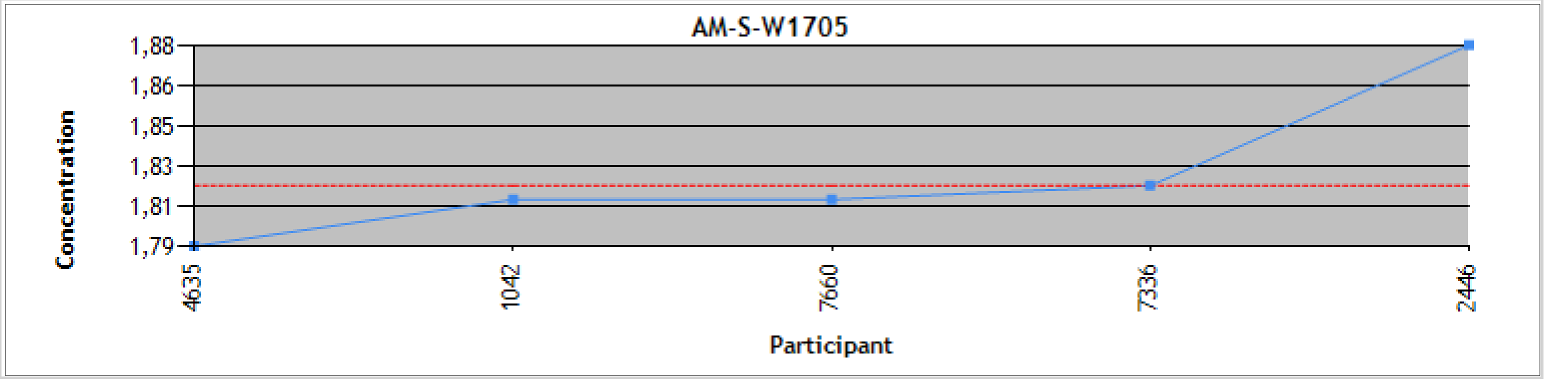

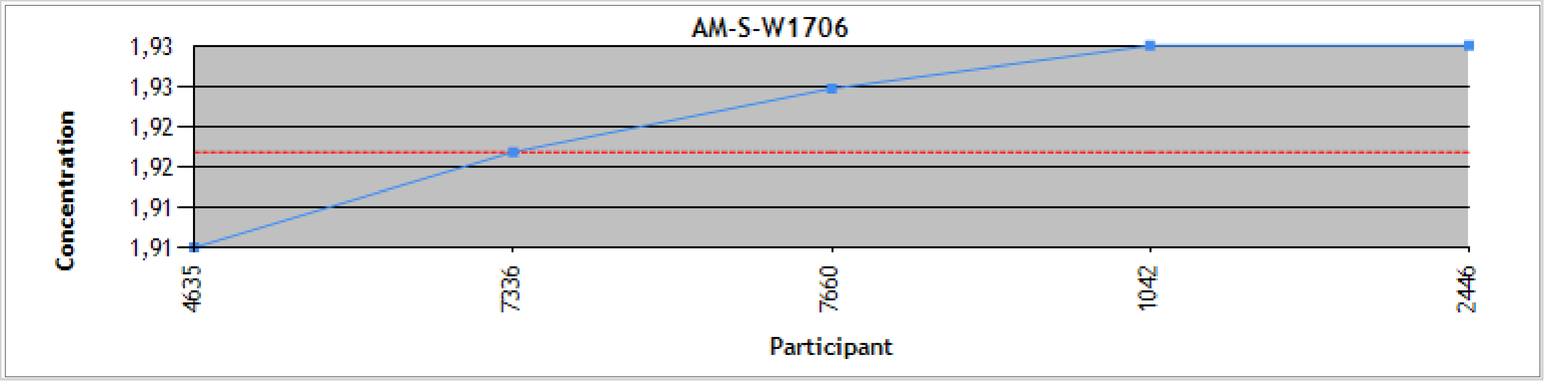

Individual results

Serum Total lipids (g/L)

Round #2017-02

Participant	AM-S-W1704	z'-score	AM-S-W1705	z'-score	AM-S-W1706	z'-score	Method
**1042**	8.78	0.86	7.39	0.39	7.08	0.86	ND
**2446**	8.29	0.31	7.24	0.18	6.68	0.30	ND
**3112**	6.00	**-2.30**	5.20	**-2.66**	4.76	**-2.40**	ND
**4635**	8.29	0.31	7.30	0.26	6.85	0.54	ND
**7660**	8.85	0.94	7.44	0.46	7.10	0.89	ND
**8559**	7.75	-0.31	6.50	-0.85	6.00	-0.66	ND
**9880**	6.64	-1.57	6.79	-0.45	5.84	-0.89	GC

	Assigned value	Standard uncertainty	σ pt	Acceptable range	K-S (Lilliefors)	Species
**AM-S-W1704**	8.02	0.393	0.787	6.26 - 9.78	Accepted	---
**AM-S-W1705**	7.11	0.160	0.699	5.68 - 8.54	Accepted	---
**AM-S-W1706**	6.47	0.317	0.637	5.05 - 7.89	Accepted	---

Total lipids are not included in the scope of our accreditation.

Statistics

Serum Total lipids (g/L)

All methods	AM-S-W1704	AM-S-W1705	AM-S-W1706
N	7	7	7
Robust mean Algo A	8.02	7.11	6.47
Robust STDev	0.831	0.339	0.671
Median	8.29	7.24	6.68
STDev from MAD	0.801	0.297	0.623
Arithmetic mean	7.80	6.84	6.33
STDev	1.09	0.800	0.853
CV or Variability	10.4%	4.8%	10.4%

When fewer than 20 results were considered for statistical treatment of all or a sub-sample of results, the accuracy of statistical data may be questionable.

Distribution

Serum Total lipids (g/L)

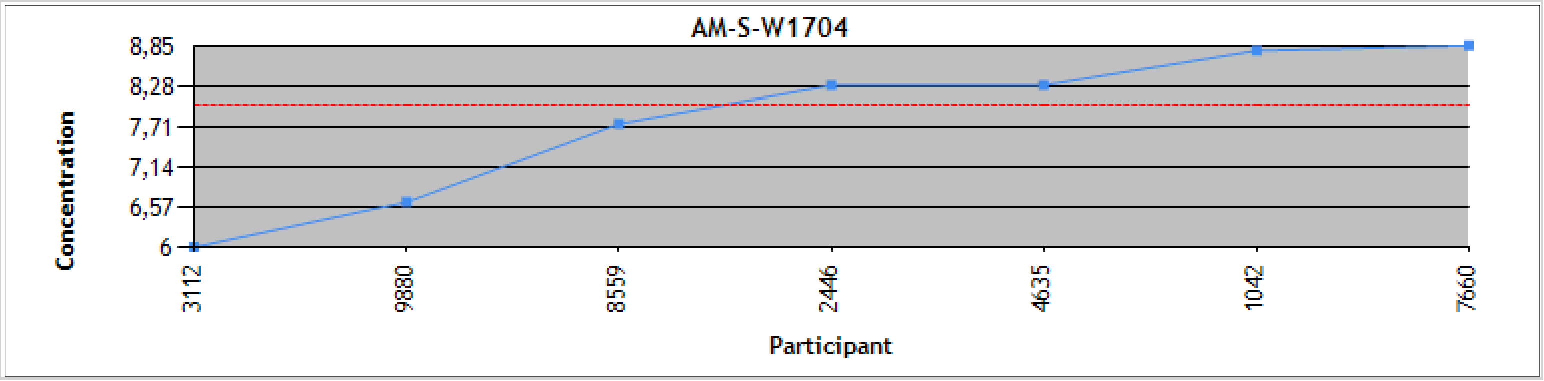

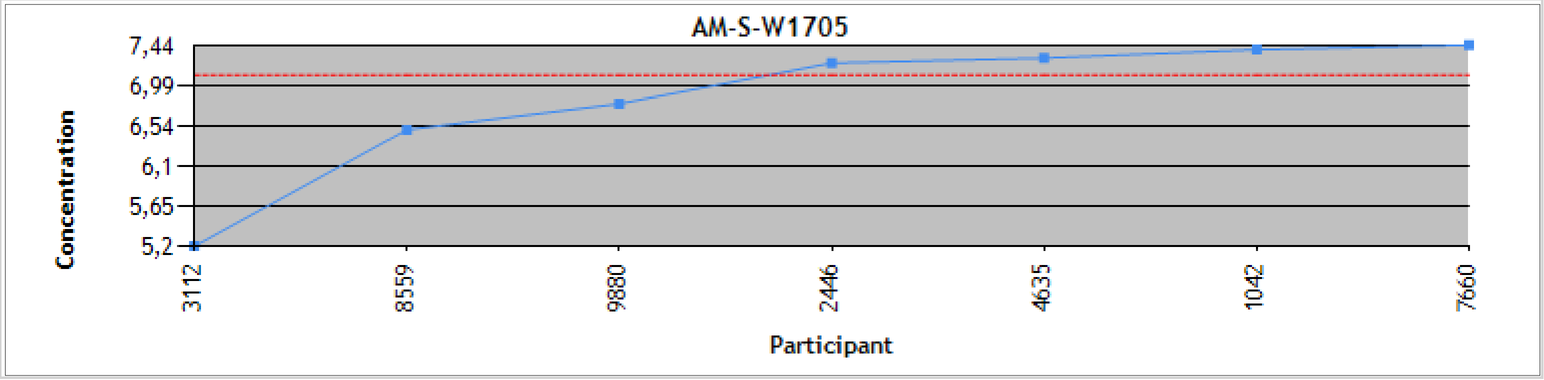

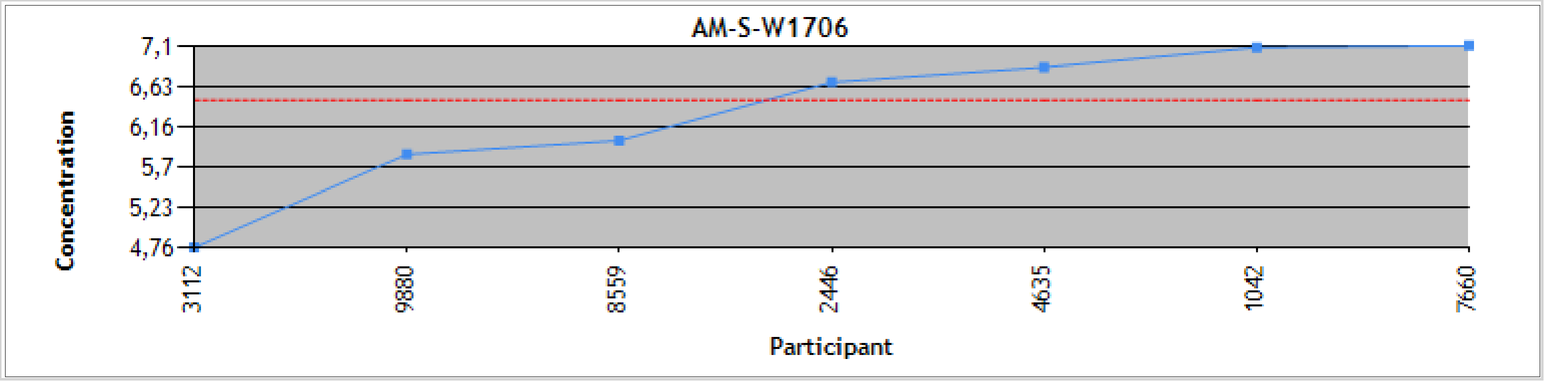

Individual results

Serum Toxaphene Parlar # 26 (µg/L)

Round #2017-02

Participant	AM-S-W1704	z'-score	AM-S-W1705	z'-score	AM-S-W1706	z'-score	Method
**1042**	0.0456	-0.08	0.389	0.21	0.146	-0.10	GC-MS CI
**6865**	0.0641	**2.04**	0.377	0.00	0.172	1.00	GC
**7660**	0.0439	-0.28	0.365	-0.21	0.140	-0.33	GC-MS CI

	Assigned value	Standard uncertainty	σ pt	Acceptable range	K-S (Lilliefors)	Species
**AM-S-W1704**	0.0463	0.00238	0.00838	0.0289 - 0.0637	Accepted	---
**AM-S-W1705**	0.377	0.00949	0.0553	0.265 - 0.489	Accepted	---
**AM-S-W1706**	0.148	0.00716	0.0228	0.100 - 0.196	Accepted	---

Criteria of homogeneity: according to ISO/CEI 17043 criteria, toxaphene parlar #26 in material AM-S-W1706 doesn't respect acceptable heterogeneity. The statistical data are produced as an indication only.

Statistics
Serum Toxaphene Parlar # 26 (µg/L)

All methods	AM-S-W1704	AM-S-W1705	AM-S-W1706
N	3	3	3
Robust mean Algo A	0.0463	0.377	0.148
Robust STDev	0.00329	0.0132	0.00992
Median	0.0456	0.377	0.146
STDev from MAD	0.00261	0.0172	0.00786
Arithmetic mean	0.0512	0.377	0.153
STDev	0.0112	0.0116	0.0170
CV or Variability	7.1%	3.5%	6.7%

When fewer than 20 results were considered for statistical treatment of all or a sub-sample of results, the accuracy of statistical data may be questionable.

Distribution

Serum Toxaphene Parlar # 26 (µg/L)

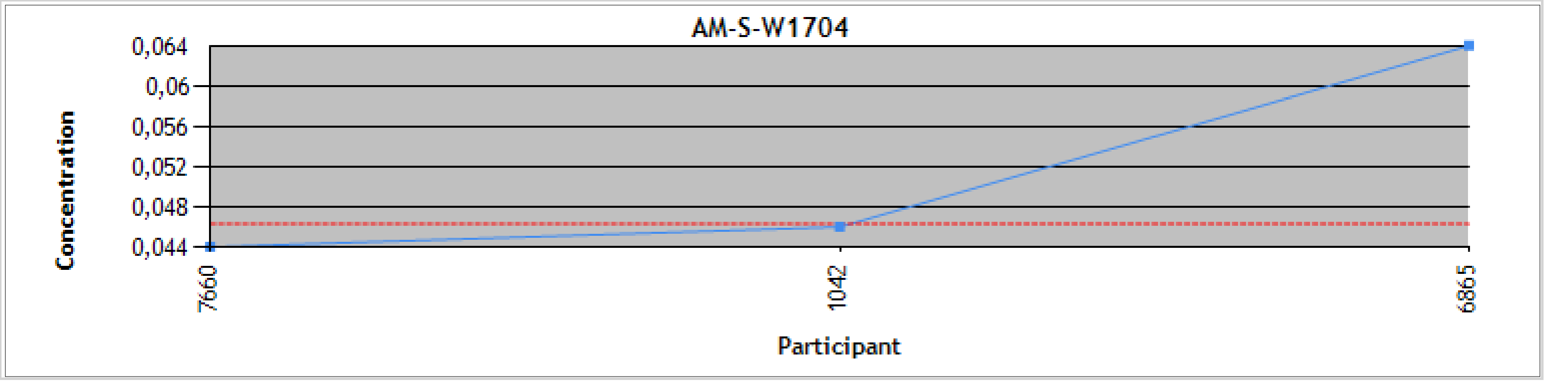

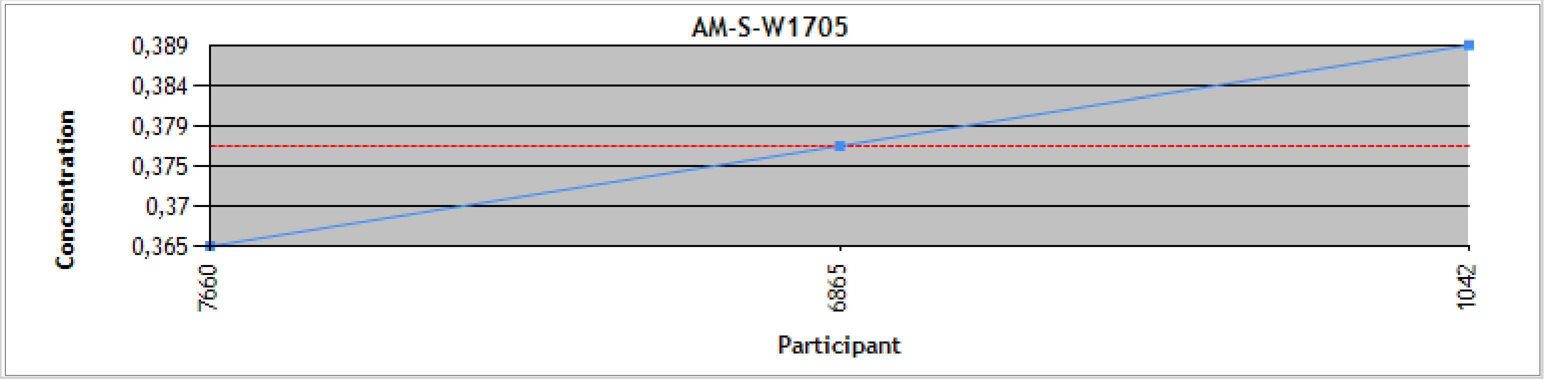

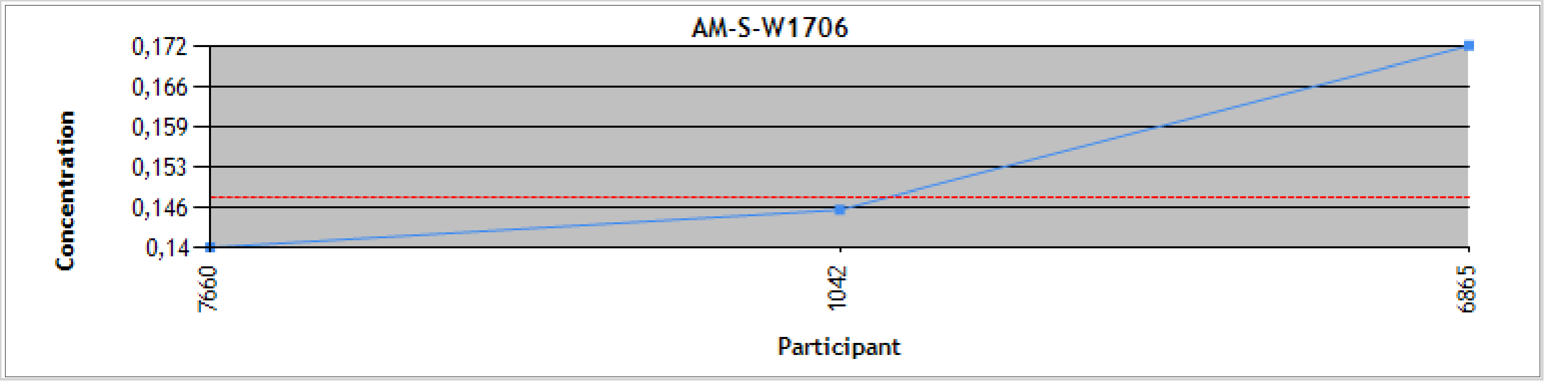

Individual results

Serum Toxaphene Parlar # 50 (µg/L)

Round #2017-02

Participant	AM-S-W1704	z'-score	AM-S-W1705	z'-score	AM-S-W1706	z'-score	Method
**1042**	0.0770	0.02	0.252	-0.10	0.103	0.22	GC-MS CI
**6865**	0.0775	0.06	0.290	0.83	0.0960	-0.19	GC
**7660**	0.0751	-0.14	0.242	-0.34	0.0984	-0.03	GC-MS CI

	Assigned value	Standard uncertainty	σ pt	Acceptable range	K-S (Lilliefors)	Species
**AM-S-W1704**	0.0768	0.000635	0.0126	0.0517 - 0.102	Accepted	---
**AM-S-W1705**	0.256	0.0134	0.0387	0.174 - 0.338	Accepted	---
**AM-S-W1706**	0.0990	0.00269	0.0158	0.0670 - 0.131	Accepted	---

Criteria of homogeneity: according to ISO/CEI 17043 criteria, toxaphene parlar #50 in material AM-S-W1706 doesn't respect acceptable heterogeneity. The statistical data are produced as an indication only.

Statistics
Serum Toxaphene Parlar # 50 (µg/L)

All methods	AM-S-W1704	AM-S-W1705	AM-S-W1706
N	3	3	3
Robust mean Algo A	0.0768	0.256	0.0990
Robust STDev	0.000880	0.0185	0.00372
Median	0.0770	0.252	0.0984
STDev from MAD	0.000697	0.0147	0.00362
Arithmetic mean	0.0765	0.261	0.0990
STDev	0.00128	0.0254	0.00328
CV or Variability	1.1%	7.2%	3.8%

When fewer than 20 results were considered for statistical treatment of all or a sub-sample of results, the accuracy of statistical data may be questionable.

Distribution

Serum Toxaphene Parlar # 50 (µg/L)

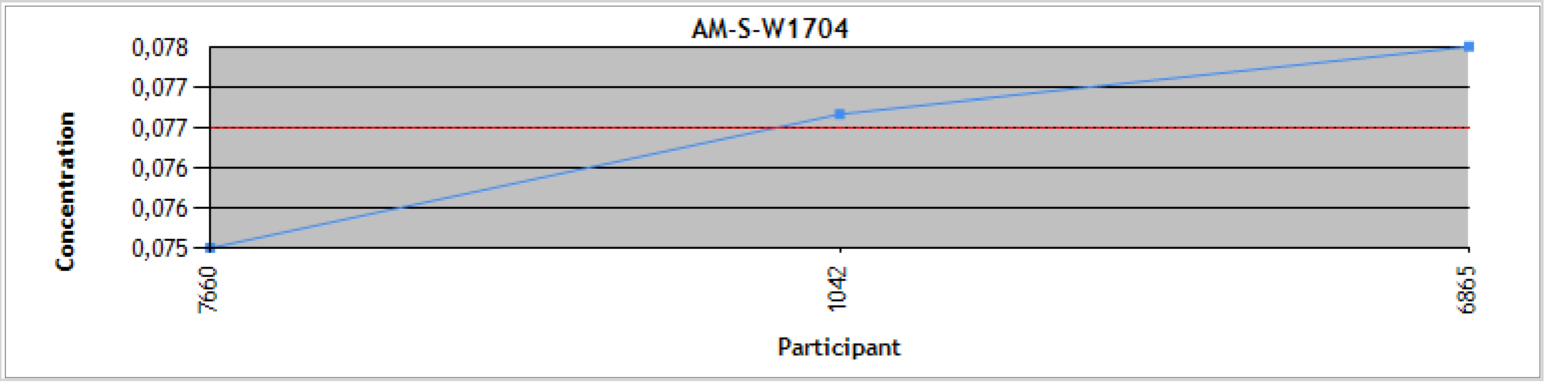

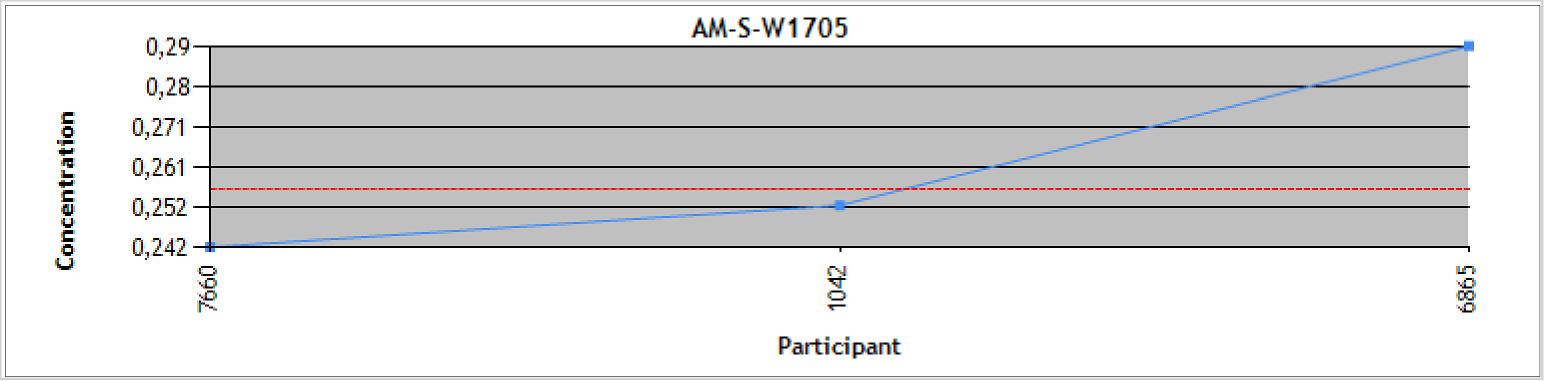

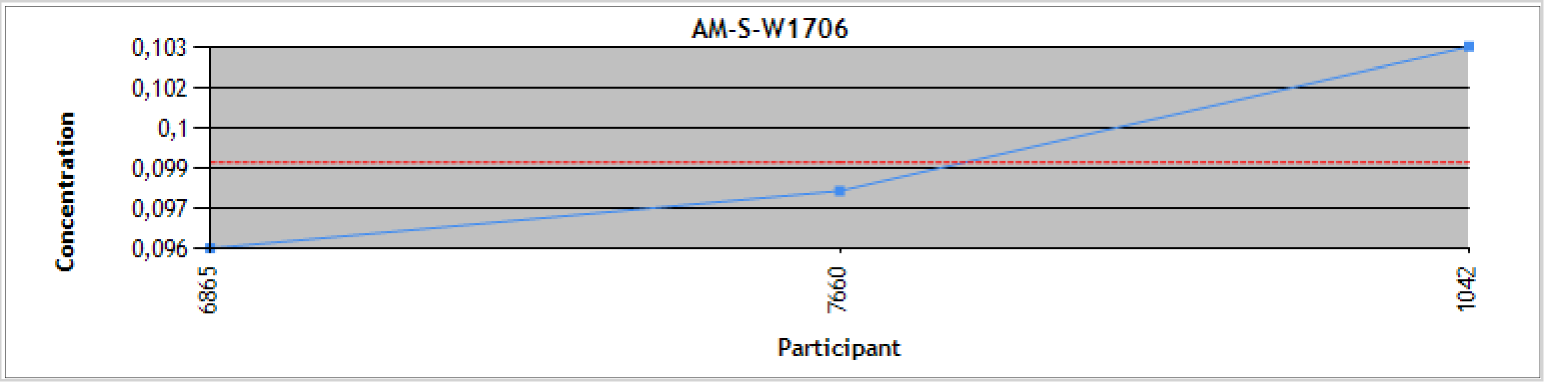

Individual results

Serum trans-Nonachlor (µg/L)

Round #2017-02

Participant	AM-S-W1704	z'-score	AM-S-W1705	z'-score	AM-S-W1706	z'-score	Method
**1003**	0.544	0.16	1.58	0.00	3.19	0.69	GC-MS-MS
**1042**	0.546	0.19	1.60	0.12	3.05	0.27	GC-MS CI
**2446**	0.514	-0.31	1.50	-0.50	2.94	-0.05	GC-MS EI
**3112**	0.467	-1.04	1.35	-1.38	2.72	-0.72	GC-MS CI
**4635**	0.569	0.54	1.68	0.57	3.23	0.82	GC-MS EI
**6865**	0.640	1.65	1.66	0.46	3.66	**2.10**	GC
**7660**	0.540	0.09	1.62	0.21	3.17	0.64	GC-MS CI
**7828**	0.614	1.24	1.81	1.37	3.52	1.69	GC-MS EI
**8420**	0.530	-0.06	1.62	0.24	2.95	-0.03	ND
**8559**	0.496	-0.59	1.54	-0.24	2.46	-1.50	ND
**8916**	0.528	-0.09	1.54	-0.26	2.93	-0.10	GC-MS EI
**9301**	0.287	**-3.83**	0.692	**-5.28**	1.42	**-4.63**	GC-MS-MS
**9426**	<LD	---	<LD	---	2.00	**-2.89**	GC-MS-MS

	Assigned value	Standard uncertainty	σ pt	Acceptable range	K-S (Lilliefors)	Species
**AM-S-W1704**	0.534	0.0167	0.0622	0.405 - 0.663	Accepted	---
**AM-S-W1705**	1.58	0.0353	0.165	1.24 - 1.92	Accepted	---
**AM-S-W1706**	2.96	0.145	0.299	2.29 - 3.63	Accepted	---

Statistics
Serum trans-Nonachlor (µg/L)

All methods	AM-S-W1704	AM-S-W1705	AM-S-W1706
N	12	12	13
Robust mean Algo A	0.534	1.58	2.96
Robust STDev	0.0464	0.0979	0.418
Median	0.535	1.59	2.95
STDev from MAD	0.0408	0.0905	0.356
Arithmetic mean	0.523	1.51	2.87
STDev	0.0879	0.282	0.611
CV or Variability	8.7%	6.2%	14.1%

GC-MS CI	AM-S-W1704	AM-S-W1705	AM-S-W1706
N	3	3	3
Robust mean Algo A	0.537	1.59	3.00
Robust STDev	0.0118	0.0299	0.234
Median	0.540	1.60	3.05
STDev from MAD	0.00934	0.0237	0.185
Arithmetic mean	0.518	1.52	2.98
STDev	0.0441	0.151	0.234
CV or Variability	2.2%	1.9%	7.8%

GC-MS EI	AM-S-W1704	AM-S-W1705	AM-S-W1706
N	4	4	4
Robust mean Algo A	0.555	1.63	3.13
Robust STDev	0.0490	0.160	0.274
Median	0.549	1.61	3.09
STDev from MAD	0.0408	0.133	0.227
Arithmetic mean	0.556	1.63	3.16
STDev	0.0450	0.143	0.282
CV or Variability	8.8%	9.8%	8.8%

GC-MS-MS	AM-S-W1704	AM-S-W1705	AM-S-W1706
N	NA	NA	3
Robust mean Algo A	NA	NA	2.20
Robust STDev	NA	NA	1.02
Median	NA	NA	2.00
STDev from MAD	NA	NA	0.860
Arithmetic mean	NA	NA	2.20
STDev	NA	NA	0.902
CV or Variability	NA	NA	46.4%

When fewer than 20 results were considered for statistical treatment of all or a sub-sample of results, the accuracy of statistical data may be questionable.

Distribution

Serum trans-Nonachlor (µg/L)

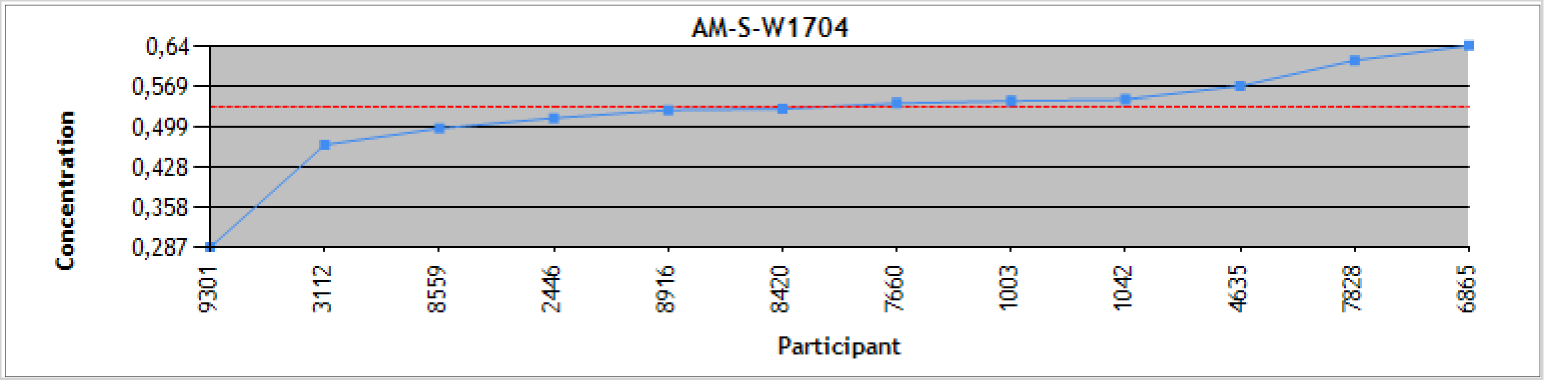

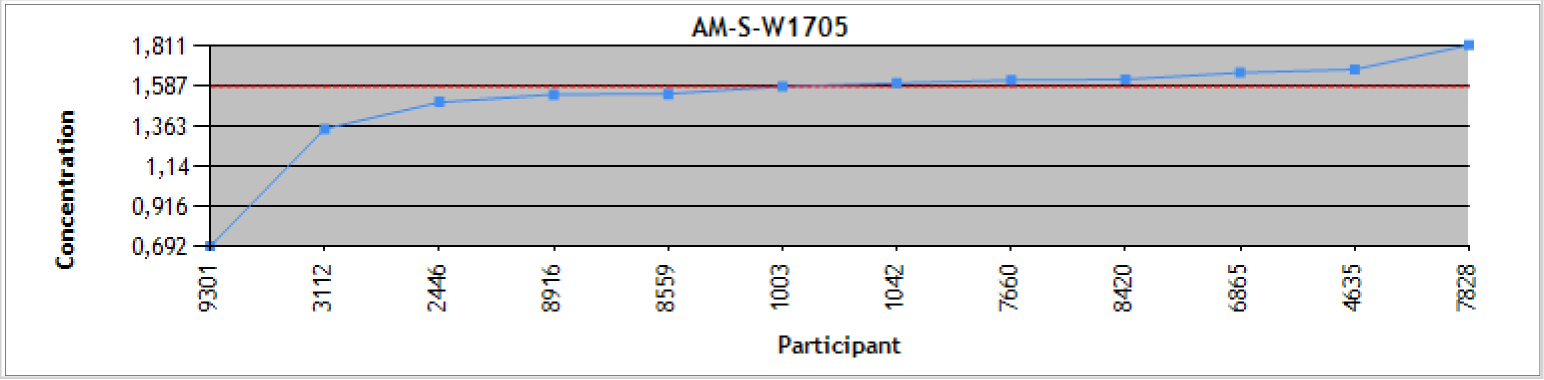

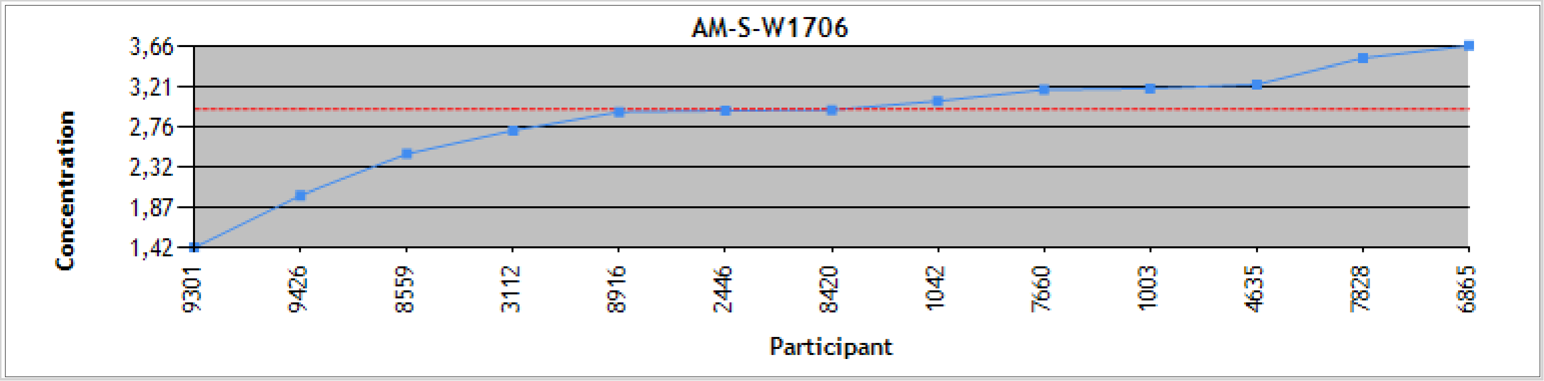

Individual results

Serum Triglycerides (g/L)

Round #2017-02

Participant	AM-S-W1704	z'-score	AM-S-W1705	z'-score	AM-S-W1706	z'-score	Method
**1042**	1.97	-0.19	2.38	-0.82	1.80	-0.35	ND
**2446**	1.81	**-2.12**	2.32	-1.33	1.62	**-2.23**	ND
**4635**	2.04	0.54	2.60	1.02	1.89	0.65	ND
**7336**	2.07	0.94	2.65	1.42	1.96	1.38	ND
**7660**	1.98	-0.08	2.44	-0.30	1.82	-0.06	ND

	Assigned value	Standard uncertainty	σ pt	Acceptable range	K-S (Lilliefors)	Species
**AM-S-W1704**	1.99	0.0494	0.0689	1.82 - 2.16	Accepted	---
**AM-S-W1705**	2.48	0.0898	0.0794	2.24 - 2.72	Accepted	---
**AM-S-W1706**	1.83	0.0678	0.0656	1.64 - 2.02	Accepted	---

Triglycerides is not included in the scope of our accreditation.

Statistics
Serum Triglycerides (g/L)

All methods	AM-S-W1704	AM-S-W1705	AM-S-W1706
N	5	5	5
Robust mean Algo A	1.99	2.48	1.83
Robust STDev	0.0884	0.161	0.121
Median	1.98	2.44	1.82
STDev from MAD	0.0786	0.184	0.0994
Arithmetic mean	1.97	2.48	1.82
STDev	0.100	0.142	0.128
CV or Variability	4.5%	6.5%	6.6%

When fewer than 20 results were considered for statistical treatment of all or a sub-sample of results, the accuracy of statistical data may be questionable.

Distribution
Serum Triglycerides (g/L)

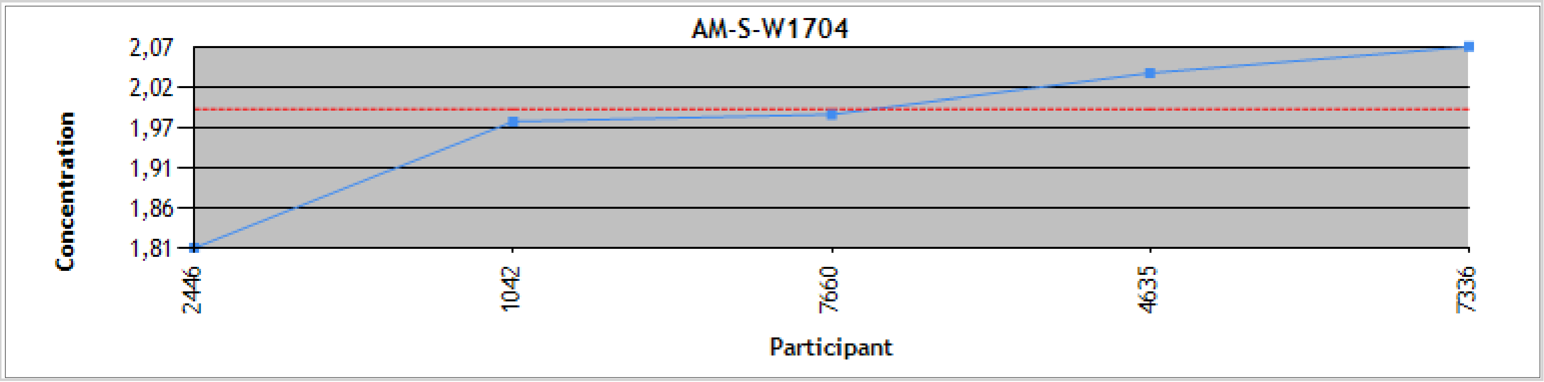

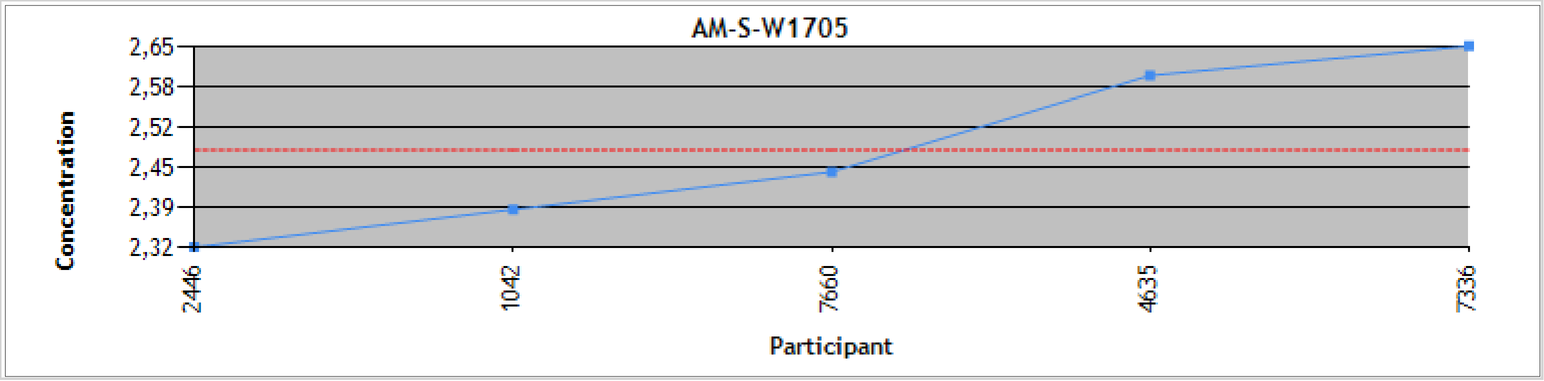

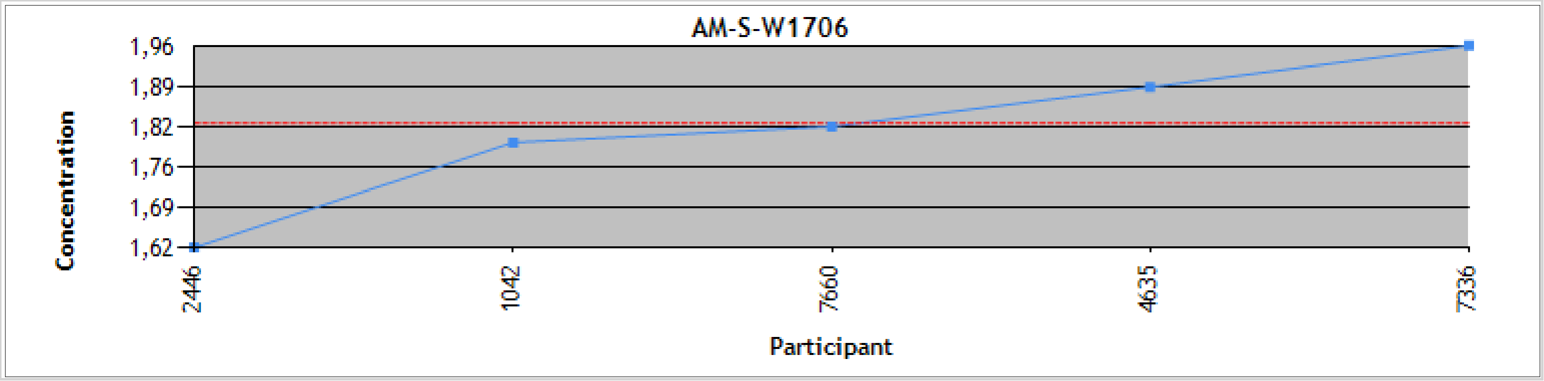

AMAP Ring Test
for Persistent Organic
Pollutants in Human Serum

ASSIGNED VALUES

ROUND: 2017-02

SHIPPED ON: 2017-05-01

DEADLINE: 2017-06-16

MATRIX	ANALYTE	UNIT	PTM	ASSIGNED VALUE	PTM	ASSIGNED VALUE	PTM	ASSIGNED VALUE
Serum	Dieldrin	µg/L	AM-S-W1704	0.131	AM-S-W1705	0.686	AM-S-W1706	1.58
	ß-HCH	µg/L	AM-S-W1704	0.266	AM-S-W1705	0.977	AM-S-W1706	0.534
	Heptachlor	µg/L	AM-S-W1704	0.0729	AM-S-W1705	0.633	AM-S-W1706	0.457
	Heptachlor epoxide	µg/L	AM-S-W1704	0.105	AM-S-W1705	1.19	AM-S-W1706	0.676
	Hexachlorobenzene	µg/L	AM-S-W1704	0.320	AM-S-W1705	1.27	AM-S-W1706	0.750
	Oxychlordane	µg/L	AM-S-W1704	0.200	AM-S-W1705	0.751	AM-S-W1706	1.52
	p,p'-DDE	µg/L	AM-S-W1704	1.57	AM-S-W1705	4.30	AM-S-W1706	8.49
	p,p'-DDT	µg/L	AM-S-W1704	0.197	AM-S-W1705	0.551	AM-S-W1706	0.968
	PBDE IUPAC # 28	µg/L	AM-S-W1704	0.0950	AM-S-W1705	0.428	AM-S-W1706	0.228
	PBDE IUPAC # 47	µg/L	AM-S-W1704	0.484	AM-S-W1705	1.12	AM-S-W1706	0.625
	PBDE IUPAC # 99	µg/L	AM-S-W1704	0.108	AM-S-W1705	0.443	AM-S-W1706	0.223
	PBDE IUPAC # 100	µg/L	AM-S-W1704	0.164	AM-S-W1705	0.325	AM-S-W1706	0.465
	PBDE IUPAC # 153	µg/L	AM-S-W1704	0.218	AM-S-W1705	0.518	AM-S-W1706	0.0831
	PBDE IUPAC # 154	µg/L	AM-S-W1704	0.0760	AM-S-W1705	0.571	AM-S-W1706	0.345
	PBDE IUPAC # 183	µg/L	AM-S-W1704	0.218	AM-S-W1705	0.354	AM-S-W1706	0.105
	PBDE IUPAC # 209	µg/L	AM-S-W1704	0.227	AM-S-W1705	1.04	AM-S-W1706	0.606
	PCB IUPAC # 28	µg/L	AM-S-W1704	0.203	AM-S-W1705	0.739	AM-S-W1706	0.399
	PCB IUPAC # 52	µg/L	AM-S-W1704	0.121	AM-S-W1705	0.283	AM-S-W1706	0.554
	PCB IUPAC # 74	µg/L	AM-S-W1704	0.160	AM-S-W1705	0.869	AM-S-W1706	0.283
	PCB IUPAC # 99	µg/L	AM-S-W1704	0.124	AM-S-W1705	0.684	AM-S-W1706	0.280
	PCB IUPAC # 101	µg/L	AM-S-W1704	0.245	AM-S-W1705	0.587	AM-S-W1706	1.17

Centre de toxicologie/INSPQ
945, Wolfe Avenue
Quebec, QC
Canada, G1V 5B3
Phone: (418) 650-5115, ext. 5254
Fax: (418) 654-2148
E-mail: ctq@inspq.qc.ca
http://www.inspq.qc.ca/ctq

Institut national de santé publique Québec
Centre de toxicologie

AMAP Ring Test for Persistent Organic Pollutants in Human Serum

ASSIGNED VALUES

ROUND: 2017-02

SHIPPED ON: 2017-05-01

DEADLINE: 2017-06-16

MATRIX	ANALYTE	UNIT	PTM	ASSIGNED VALUE	PTM	ASSIGNED VALUE	PTM	ASSIGNED VALUE
Serum	PCB IUPAC # 105	µg/L	AM-S-W1704	0.0709	AM-S-W1705	0.212	AM-S-W1706	0.122
	PCB IUPAC # 118	µg/L	AM-S-W1704	0.126	AM-S-W1705	0.431	AM-S-W1706	0.269
	PCB IUPAC # 138	µg/L	AM-S-W1704	0.332	AM-S-W1705	1.60	AM-S-W1706	0.765
	PCB IUPAC # 153	µg/L	AM-S-W1704	0.281	AM-S-W1705	0.958	AM-S-W1706	1.78
	PCB IUPAC # 170	µg/L	AM-S-W1704	0.181	AM-S-W1705	0.499	AM-S-W1706	0.296
	PCB IUPAC # 180	µg/L	AM-S-W1704	0.263	AM-S-W1705	0.586	AM-S-W1706	0.673
	PCB IUPAC # 183	µg/L	AM-S-W1704	0.250	AM-S-W1705	0.648	AM-S-W1706	1.11
	PCB IUPAC # 187	µg/L	AM-S-W1704	0.188	AM-S-W1705	0.860	AM-S-W1706	0.573
	PFHxA	µg/L	AM-S-Y1704	3.32	AM-S-Y1705	1.62	AM-S-Y1706	4.51
	PFHxS	µg/L	AM-S-Y1704	0.721	AM-S-Y1705	11.8	AM-S-Y1706	5.90
	PFNA	µg/L	AM-S-Y1704	0.684	AM-S-Y1705	2.59	AM-S-Y1706	1.36
	PFOA	µg/L	AM-S-Y1704	14.3	AM-S-Y1705	3.24	AM-S-Y1706	6.53
	PFOS	µg/L	AM-S-Y1704	96.1	AM-S-Y1705	34.3	AM-S-Y1706	177
	PFUdA	µg/L	AM-S-Y1704	0.644	AM-S-Y1705	2.31	AM-S-Y1706	1.50
	Total Cholesterol	g/L	AM-S-W1704	2.53	AM-S-W1705	1.82	AM-S-W1706	1.92
	Total lipids	g/L	AM-S-W1704	8.02	AM-S-W1705	7.11	AM-S-W1706	6.47
	Toxaphene Parlar # 26	µg/L	AM-S-W1704	0.0463	AM-S-W1705	0.377	AM-S-W1706	0.148
	Toxaphene Parlar # 50	µg/L	AM-S-W1704	0.0768	AM-S-W1705	0.256	AM-S-W1706	0.0990
	trans-nonachlor	µg/L	AM-S-W1704	0.534	AM-S-W1705	1.58	AM-S-W1706	2.96
	Triglycerides	g/L	AM-S-W1704	1.99	AM-S-W1705	2.48	AM-S-W1706	1.83

GROUPING OF ANALYTICAL METHODS FOR STATISTICS

METHODS GROUPING CODE	METHODS GROUPING	METHODS INCLUDED	METHODS CODE
GC	GC	GC-ECD	GC-ECD
		GC-FID	GC-FID
GC-MS EI	GC-MS EI	GC-HRMS	GC-HRMS
		GC-MS EI	GC-MS EI
GC-MS CI	GC-MS CI	GC-MS NCI	GC-MS NCI
GC-MS-MS	GC-MS-MS	GC-MS-MS EI	GC-MS-MS EI
		GC-MS-MS NCI	GC-MS-MS NCI
LC-MS	LC-MS	LC-MS APCI	LC-MS APCI
		LC-MS ESI	LC-MS ESI
LC-MS-MS	LC-MS-MS	LC-MS-MS APCI	LC-MS-MS APCI
		LC-MS-MS ESI	LC-MS-MS ESI

1. Kolmogorov-Smirnov (Lilliefors) test

The Kolmogorov-Smirnov (Lilliefors) statistical test is a tool used to evaluate the normality of a distribution of results. The test can be performed with N ≥ 4.

The power of the test increases rapidly with the number of observations. For N ≥ 30, the test will be sensitive to account for small deviations from normality even with the presence of only one outlier.

For a small sample size (N ≤ 10), the hypothesis that the observations follow a normal distribution will generally be accepted. However, if normality is rejected for N ≤ 10, this indicates that the observations are far from following a normal distribution and that one or more observations are most likely outliers.

When the normality hypothesis is rejected, the use of robust methods for the determination of the AV by consensus such as the Algorithm A allows to obtain a better estimation of the AV. Outliers are retained except if their deviation from the other observations can be clearly explained as indicated in section 1.1.

The normality hypothesis is rejected when the statistical value is too weak according to the Kolmogorov-Smirnov (Lilliefors) critical value table. The result of the normality of the distribution of each set of observations is provided in the report whether the test is "Accepted" or "Rejected" under the column labeled "KS (Lilliefors)".

***** END OF REPORT *****
